# HINT1 aggravates aortic aneurysm by targeting ITGA6/FAK axis in vascular smooth muscle cells

**DOI:** 10.1172/JCI186628

**Published:** 2025-04-08

**Authors:** Yan Zhang, Wencheng Wu, Xuehui Yang, Shanshan Luo, Xiaoqian Wang, Qiang Da, Ke Yan, Lulu Hu, Shixiu Sun, Xiaolong Du, Xiaoqiang Li, Zhijian Han, Feng Chen, Aihua Gu, Liansheng Wang, Zhiren Zhang, Bo Yu, Chenghui Yan, Yaling Han, Yi Han, Liping Xie, Yong Ji

**Affiliations:** 1 Key Laboratory of Drug Targets and Translational Medicine for Cardio-cerebrovascular Diseases, Key Laboratory of Targeted Intervention of Cardiovascular Disease, Collaborative Innovation Center for Cardiovascular Disease Translational Medicine, Medical Basic Research Innovation Center for Cardiovascular and Cerebrovascular Diseases, Ministry of Education, Nanjing Medical University, Nanjing, China.; 2 Department of Vascular Surgery, The Affiliated Nanjing Drum Tower Hospital, Nanjing University Medical School, Nanjing, China.; 3 Department of Urology, The First Affiliated Hospital of Nanjing Medical University, Nanjing, China.; 4 Department of Forensic Medicine and; 5 School of Public Health, Nanjing Medical University, Nanjing, China.; 6 Department of Cardiology, The First Affiliated Hospital of Nanjing Medical University, Nanjing, China.; 7 State Key Laboratory of Frigid Zone Cardiovascular Diseases, Harbin Medical University, Harbin, China.; 8 Department of Cardiology, The Second Affiliated Hospital of Harbin Medical University, Key Laboratory of Myocardial Ischemia, Ministry of Education, Harbin Medical University, Harbin, China.; 9 Cardiovascular Research Institute and Department of Cardiology, General Hospital of Northern Theater Command, Shenyang, China.; 10 Critical Care Department, The Second Affiliated Hospital of Harbin Medical University, Harbin, China.

**Keywords:** Cardiology, Vascular biology, Integrins

## Abstract

Aortic aneurysm is a high-risk cardiovascular disease without an effective cure. Vascular smooth muscle cell (VSMC) phenotypic switching is a key step in the pathogenesis of aortic aneurysm. Here, we revealed the role of histidine triad nucleotide-binding protein 1 (HINT1) in aortic aneurysm. HINT1 was upregulated both in aortic tissue from patients with aortic aneurysm and angiotensin II–induced aortic aneurysm mice. VSMC-specific HINT1 deletion alleviated aortic aneurysm via preventing VSMC phenotypic switching. With the stimulation of pathological factors, the increased nuclear translocation of HINT1 mediated by nucleoporin 98 promoted the interaction between HINT1 and transcription factor AP-2 α (TFAP2A), further triggered the transcription of integrin α6 (ITGA6) mediated by TFAP2A, and consequently activated the downstream focal adhesion kinase (FAK)/STAT3 signal pathway, leading to aggravation of VSMC phenotypic switching and aortic aneurysm. Importantly, defactinib treatment was demonstrated to limit aortic aneurysm development by inhibiting the FAK signal pathway. Thus, the HINT1/ITGA6/FAK axis emerges as a potential therapeutic strategy in aortic aneurysm.

## Abstract 2

HINT1 promotes VSMC phenotypic switching and aortic aneurysm by targeting ITGA6/FAK axis. Defactinib treatment was demonstrated to limit aortic aneurysm by inhibiting FAK signal pathway.

## Graphical abstract

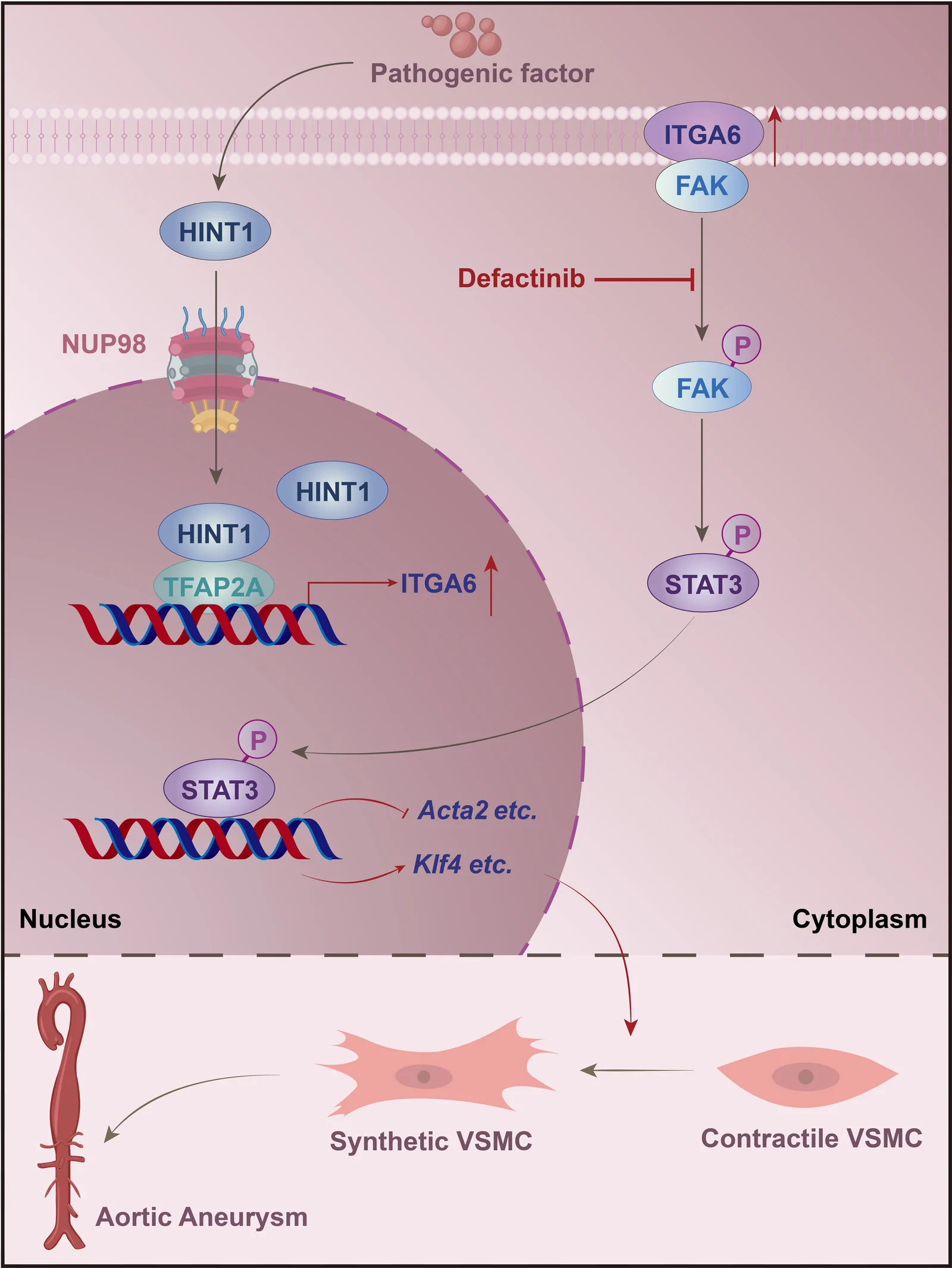

## Introduction

Aortic aneurysm is a life-threatening cardiovascular event that is characterized by permanent dilatation of aorta and an extremely high mortality rate in the event of rupture (1, 2). Aortic aneurysm is associated with high mortality, but neither predictive risk factors nor medical treatments have been established for it. Endovascular surgical repair remains the main treatment option for aortic aneurysm (3). Consequently, decoding the molecular mechanism involved in aortic aneurysm is critical for identifying potential pharmacological interventions.

Compelling evidence has shown that vascular smooth muscle cell (VSMC) phenotypic switching is a key step in the pathogenesis of aortic aneurysm (1, 4). Single-cell RNA-Seq and lineage tracing analyses revealed that VSMC phenotypic switching plays an essential role in the pathogenesis of aortic aneurysms (5–7). VSMCs are highly plastic, and they can exist in either contractile or synthetic phenotype in response to various stimuli, including microenvironmental cues (8, 9). The contractile phenotype of VSMCs is marked by high expression of calponin 1 (CNN1), α-smooth muscle actin (α-SMA), and smooth muscle protein 22 (SM22), which are essential for cellular contraction, modulation of vascular tone, blood pressure homeostasis, and blood flow redistribution. Synthetic VSMCs can markedly increase their capacity to proliferate, migrate, and promote the synthesis of elastolytic and proinflammatory factors (10). This phenotypic switching results in extracellular matrix degradation and aortic wall weakening, which ultimately render the aorta prone to rupture and aortic aneurysm progression (11, 12). However, molecular mechanisms governing VSMC phenotypic switching during the development of aortic aneurysm remain incompletely understood.

Histidine triad nucleotide-binding protein 1 (HINT1) is a highly conserved protein that belongs to the histidine triad superfamily, members of which contain the His-X-His-X-His-X motif (where X is a hydrophobic amino acid) (13). Recent studies suggest that HINT1 plays important roles in diverse neuropsychiatric diseases, including schizophrenia, mood disorders, drug addiction, and inherited peripheral neuropathies (14, 15). Loss of HINT1 increases susceptibility to carcinogenesis in mice, suggesting a role as a tumor suppressor (16, 17). HINT1 is located in both cytoplasm and the nucleus (18, 19). Our previous study demonstrated that HINT1 in cardiomyocytes protects from cardiohypertrophy as an important signal transduction molecule (20). However, the roles of HINT1 in VSMCs and aortic aneurysm have not been addressed.

In this study, by using animal and cell models, we found that HINT1 in VSMCs promoted VSMC phenotypic switching and aggravated aortic aneurysm induced by angiotensin II (Ang II) in mice. Further, we identified integrin α6 (ITGA6) as a target of HINT1 that contributed to VSMC phenotypic switching through activating the focal adhesion kinase (FAK)/STAT3 signal pathway. Mechanically, under the stimulation of pathological factors, the nuclear translocation of HINT1 mediated by nucleoporin 98 (Nup98) increased significantly and HINT1 directly bound to TFAP2A, upregulating the transcription of ITGA6 mediated by TFAP2A, leading to activating the FAK/STAT3 signal pathway, and further resulting in aggravating VSMC phenotypic switching. Defactinib, an inhibitor of FAK, significantly limited aortic aneurysm progression in mice by targeting the ITGA6/FAK axis.

## Results

### Increased HINT1 expression in the vascular smooth muscle is associated with the occurrence of aortic aneurysm.

**Figure 1:**
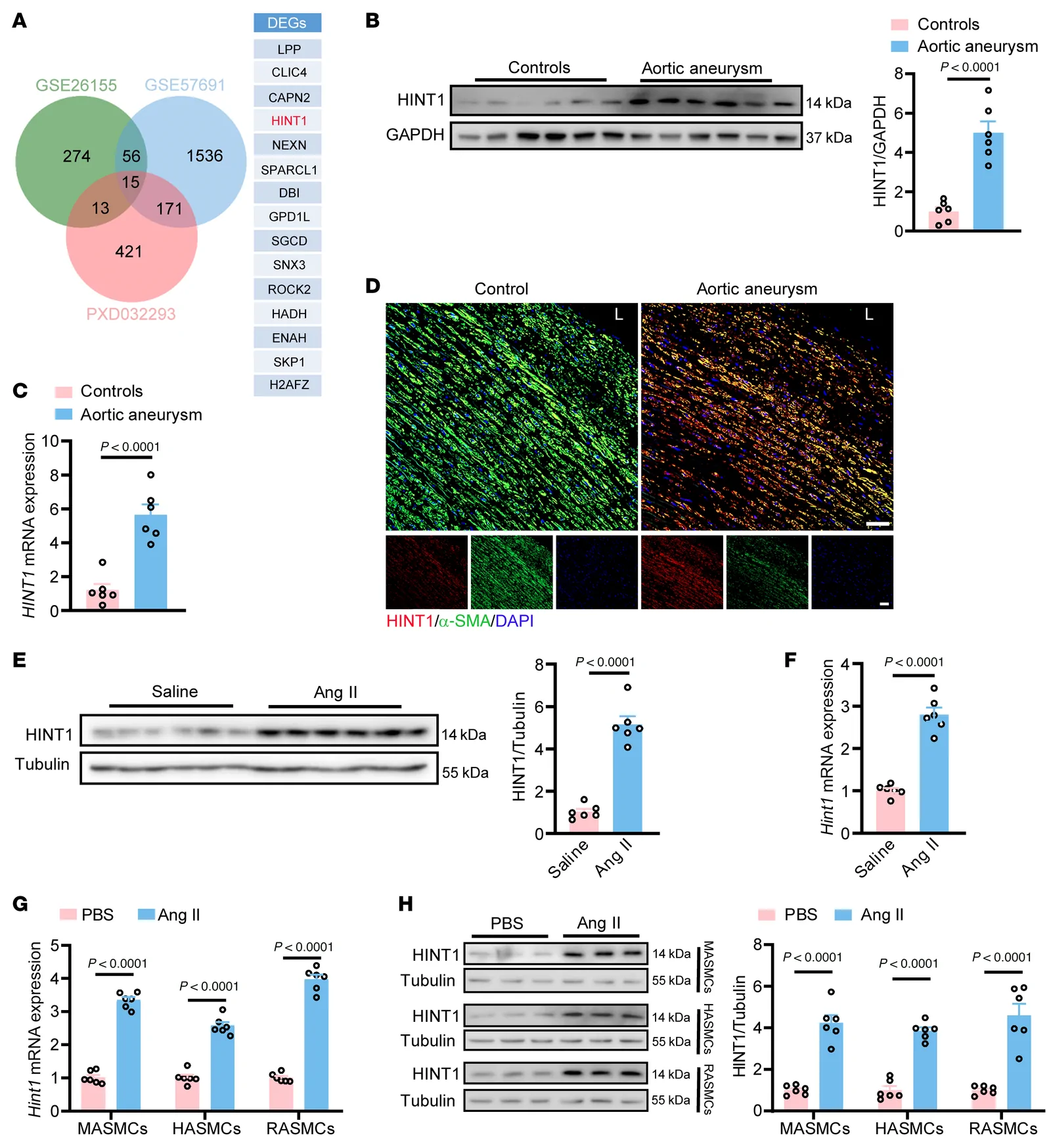
Upregulation of HINT1 correlates with aortic aneurysm. (**A**) Differentially expressed genes/proteins were identified from aortic tissue of aortic aneurysm patients and controls in 3 databases (green, GSE57691; blue, GSE26155; and red, PXD03229). Venn diagram showing the comparison among the 3 datasets identified 15 overlapping targets. (**B** and **C**) Western blotting (**B**) and q-PCR (**C**) analysis of HINT1 expression in aorta samples from aortic aneurysm patients and normal aorta samples from donors (*n* = 6 per group). (**D**) Representative immunofluorescence images of α-SMA and HINT1 in aortic samples from aortic aneurysm patients and nonaortic aneurysm controls. Red, HINT1; green, α-SMA; blue, DAPI; L, lumen. Scale bars: 50 μm. (**E** and **F**) Eight-week-old male *Apoe^–/–^* mice were infused with saline or Ang II (1,000 ng/kg/min) for 28 days. Western blotting (**E**) and q-PCR (**F**) analysis of HINT1 expression in mouse suprarenal abdominal aortas. *n* = 6 per group. (**G**) MASMCs were isolated from the whole aortas of mice. q-PCR analysis of the mRNA levels of *Hint1* in isolated MASMCs, HASMCs, and RASMCs stimulated with PBS or Ang II (10^–6^ M) (*n* = 6 per group). (**H**) MASMCs were isolated from the whole aortas of mice. Western blotting analysis of HINT1 in MASMCs, HASMCs, and RASMCs stimulated with PBS or Ang II (10^–6^ M) (*n* = 6 per group). Statistical analysis was performed by Student’s *t* test (**B**, **C**, and **E**–**H**). For all statistical plots, the data are presented as mean ± SEM.

To understand the mechanism of aortic aneurysms, we analyzed 2 RNA-Seq datasets (GEO, GSE26155 and GSE57691; https://www.ncbi.nlm.nih.gov/geo/) and 1 proteomics dataset (ProteomeXchange, PXD032293; https://www.proteomexchange.org/), in which differentially expressed genes/proteins were identified in aortic samples from patients with aortic aneurysm and nonaortic aneurysm controls. We identified 15 differentially expressed genes/proteins (fold change > 1.5, FDR < 0.05), which overlapped in the above 3 databases (Figure 1A). Importantly, we found HINT1, which was demonstrated to protect against cardiac hypertrophy in our previous study (20), as a member of the 15 identified genes/proteins, suggesting that it may be involved in aortic aneurysm. Next, we clarified the role of HINT1 in the aortic aneurysm. As shown in Figure 1, B and C, the protein and mRNA levels of *HINT1* were markedly higher in aorta samples from aortic aneurysm patients (Supplemental Table 1) than those in normal aorta samples from donors (Supplemental Table 2). Moreover, immunofluorescence staining also revealed increased HINT1 in aorta samples from aortic aneurysm patients than that from control subjects (Figure 1D). Meanwhile, we observed that HINT1 was colocalized with α–smooth muscle actin (α-SMA) in aorta samples (Figure 1D), which suggests that HINT1 is mainly located in VSMCs. Subcutaneous administration of Ang II by a mini pump was used to induce aortic aneurysm in *Apoe^–/–^* mice. Increases in protein (Figure 1E) and mRNA levels (Figure 1F) of *Hint1* were observed in the suprarenal abdominal aortas of *Apoe^–/–^* mice infused with Ang II compared with controls. Furthermore, we observed a marked increase of HINT1 at various time points (3, 7, and 28 days) after Ang II infusion (Supplemental Figure 1A; supplemental material available online with this article; https://doi.org/10.1172/JCI186628DS1). In addition, we found that Ang II treatment resulted in markedly increased HINT1 in mouse aortic smooth muscle cells (MASMCs), human aortic smooth muscle cells (HASMCs), and rat aortic smooth muscle cells (RASMCs) (Figure 1, G and H), while it did not affect HINT1 expression in mouse aortic endothelial cells (Supplemental Figure 1, B and C) and mouse bone marrow–derived macrophages (Supplemental Figure 1, D and E), implying an important role of HINT1 in VSMCs in aortic aneurysm. Taken together, upregulation of HINT1 expression in VSMCs is correlated with aortic aneurysm in both humans and mice, suggesting that HINT1 may play an important role in the progression of aortic aneurysm.

### VSMC-specific knockout of Hint1 alleviates Ang II–induced aortic aneurysms in vivo.

**Figure 2:**
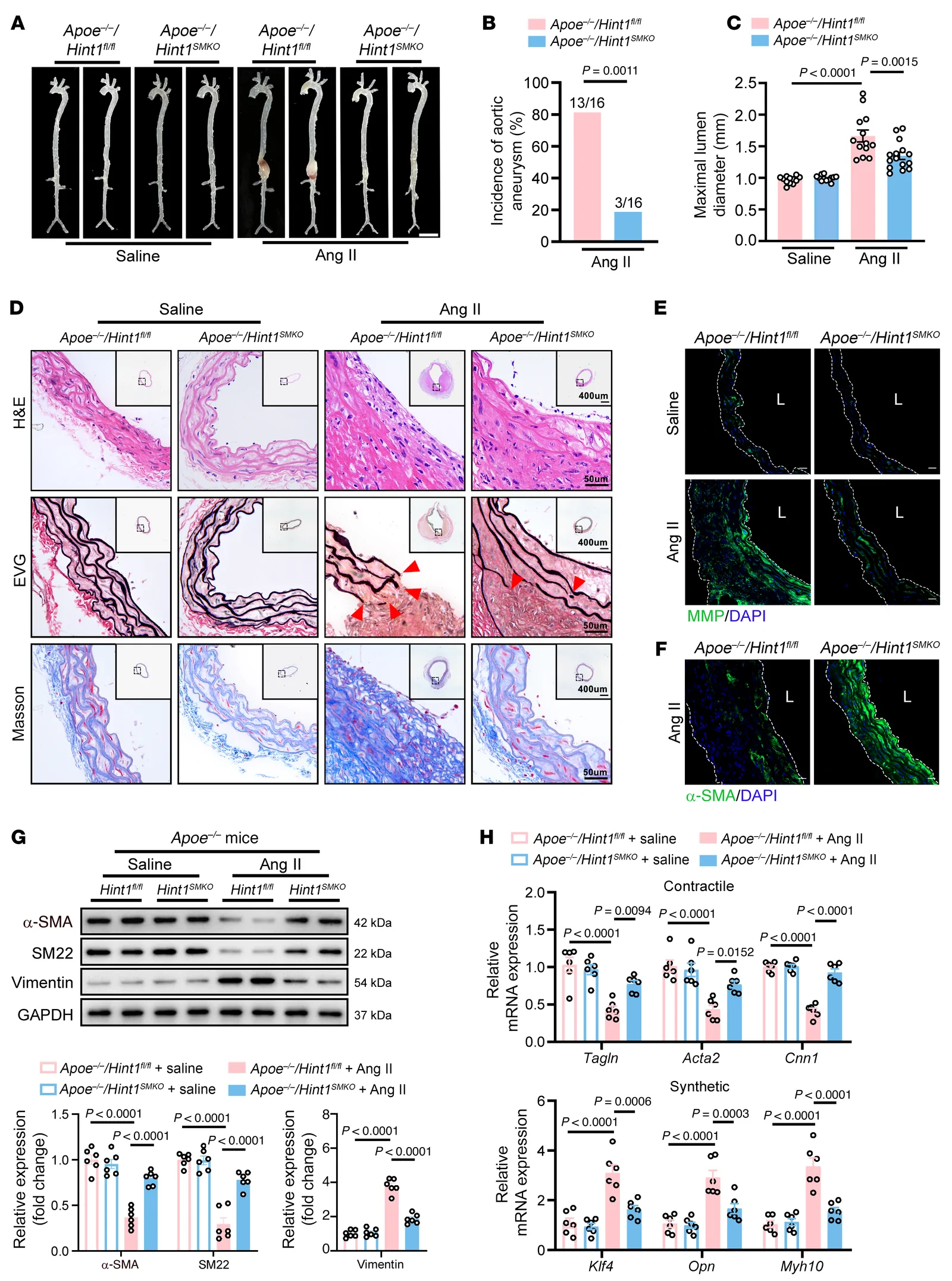
*Hint1* deficiency in VSMCs mitigates aortic aneurysm. Saline (*n* = 12 per group) or Ang II (1,000 ng/kg/min) (*n* = 16 per group) was infused subcutaneously in *Apoe^–/–^/Hint1^fl/fl^* and *Apoe^–/–^/Hint1^SMKO^* mice for 28 days. (**A**) Representative photograph of aortas from *Apoe^–/–^/Hint1^fl/fl^* and *Apoe^–/–^/Hint1^SMKO^* mice after saline or Ang II infusion. Scale bar: 2 mm. (**B**) Incidence of Ang II–induced aortic aneurysm. (**C**) Maximal abdominal lumen diameter in mice infused with saline (*n* = 12) or Ang II (*n* = 13–16), as measured by ultrasound. (**D**) Histopathological images of suprarenal abdominal aortas of *Apoe^–/–^/Hint1^fl/fl^* and *Apoe^–/–^/Hint1^SMKO^* mice after 28 days of saline or Ang II infusion. Scale bars: 50 μm and 400 μm (insets). EVG, elastic van Gieson staining. (**E**) Representative in situ zymography photomicrographs showing MMP activity of suprarenal abdominal aortas. Scale bars: 20 μm. L, lumen. (**F**) Representative immunofluorescence staining of α-SMA expression in abdominal aortas. Scale bars: 20 μm. L, lumen. (**G**) Western blotting analysis of the VSMC contractile markers (α-SMA and SM22) and synthetic marker (Vimentin) in suprarenal abdominal aortas from saline- or Ang II–infused *Apoe^–/–^/Hint1^fl/fl^* and *Apoe^–/–^/Hint1^SMKO^* mice (*n* = 6 per group). (**H**) q-PCR analysis of the mRNA levels of VSMC contractile markers (*Acta2*, *Cnn1*, and *Tagln*) and synthetic markers (*Klf4*, *Opn*, and *Myh10*) in suprarenal abdominal aortas from saline- or Ang II–infused *Apoe^–/–^/Hint1^fl/fl^* and *Apoe^–/–^/Hint1^SMKO^* mice (*n* = 6 per group). Statistical analysis was performed by Fisher’s exact test (**B**) or 1-way ANOVA (**C**, **G**, and **H**). For all statistical plots, data are shown as the mean ± SEM.

To determine the role of HINT1 in VSMCs in the progression of aortic aneurysm, we constructed VSMC-specific *Hint1*-knockout (*Hint1^SMKO^*) mice by crossing *Hint1^fl/fl^* mice with *Tagln*-Cre mice. Western blotting and quantitative PCR (q-PCR) confirmed the deletion of *Hint1* in VSMCs isolated from *Hint1^SMKO^* mice (Supplemental Figure 2, A and B). Next, *Hint1^SMKO^* mice were crossed with *Apoe^–/–^* mice. We then induced aortic aneurysm in *Apoe^–/–^*/*Hint1^SMKO^* mice and *Apoe^–/–^*/*Hint1^fl/fl^* littermates by subcutaneous infusion of Ang II for 4 weeks. Gross examination showed lower luminal expansion in the suprarenal region of the abdominal aorta of *Apoe^–/–^*/*Hint1^SMKO^* mice than in *Apoe^–/–^*/*Hint1^fl/fl^* mice induced with Ang II (Figure 2A). Notably, there were no blood pressure (BP) differences between the 2 groups, suggesting that the effects of HINT1 in VSMCs are not related to BP (Supplemental Figure 2C). The incidence of aortic aneurysm reduced from 81.2% in *Apoe^–/–^/Hint1^fl/fl^* mice to 18.7% in *Apoe^–/–^*/*Hint1^SMKO^* mice infused with Ang II (Figure 2B). The aortic rupture rate was 18.7% in *Apoe^–/–^/Hint1^fl/fl^* mice and 6.2% in *Apoe^–/–^*/*Hint1^SMKO^* mice infused with Ang II (Supplemental Figure 2D). As determined by transabdominal ultrasound imaging (Figure 2C) and postmortem measurement (Supplemental Figure 2E), *Hint1* deficiency in VSMCs markedly decreased aortic diameter in *Apoe^–/–^*/*Hint1^SMKO^* mice infused with Ang II. Morphologically, histological analysis results revealed that VSMC-specific *Hint1* knockout mitigated arterial wall thickening and reduced elastic fiber degradation and collagen deposition in Ang II–administered mouse suprarenal abdominal aortas (Figure 2D). Moreover, we found that the activity of matrix metalloproteinase decreased in the aortas of the *Apoe^–/–^*/*Hint1^SMKO^* mice by in situ zymography (Figure 2E). Furthermore, we observed markedly increased α-SMA in suprarenal abdominal aortas from *Apoe^–/–^*/*Hint1^SMKO^* mice infused with Ang II by immunofluorescence staining (Figure 2F). Consistently, we found increased contractile proteins (α-SMA and SM22) and decreased synthetic protein (Vimentin) in the suprarenal abdominal aortas from Ang II–infused *Apoe^–/–^*/*Hint1^SMKO^* mice (Figure 2G). Meanwhile, q-PCR of suprarenal abdominal aorta revealed that knockout of *Hint1* in VSMCs resulted in a marked increase of contractile genes, including *Tagln*, *Acta2*, and *Cnn1*, whereas the expression of synthetic genes, including *Klf4*, *Opn*, and *Myh10*, was reduced (Figure 2H), which suggests that HINT1 promotes VSMC phenotypic conversion.

### Hint1 deficiency represses the phenotypic switching of VSMCs.

Next, we investigated whether HINT1 triggers VSMC phenotypic switching in vitro. PDGF-BB is a recognized factor that induces VSMC phenotypic switching in vitro when Ang II is infused to induce aortic aneurysms in vivo (21, 22). We observed a marked upregulation of HINT1 in PDGF-BB–induced RASMCs, HASMCs, and MASMCs by q-PCR (Supplemental Figure 3A) and Western blot (Supplemental Figure 3B). As shown in Supplemental Figure 3C, under stimulation of PDGF-BB, primary aortic VSMCs from *Hint1*^–/–^ mice showed increased contractile proteins (α-SMA and SM22) and decreased synthetic protein (Vimentin) than controls, which suggests that deficiency of *Hint1* disrupts phenotypic transformation of VSMC in vitro. Meanwhile, q-PCR revealed that deficiency of *Hint1* in mouse VSMCs resulted in a marked increase of contractile genes, including *Tagln*, *Acta2*, and *Cnn1*, while the expression of synthetic genes, including *Klf4*, *Opn*, and *Myh10*, was decreased (Supplemental Figure 3D). Moreover, phalloidin staining revealed that *Hint1* deficiency inhibited microfilament remodeling and prevented morphological changes of VSMCs from a spindle-like contractile phenotype to a polygonal synthetic phenotype (Supplemental Figure 3E). Meanwhile, we demonstrated that knockdown of *HINT1* in HASMCs by siRNA reversed PDGF-BB–triggered VSMC phenotypic alteration from contractile to synthetic phenotypes (Supplemental Figure 3, F and G). A similar phenomenon was also observed by phalloidin staining (Supplemental Figure 3H). In addition, we also confirmed that HINT1 promoted Ang II–induced VSMC phenotypic switching in vitro (Supplemental Figure 4). Collectively, we demonstrated that *Hint1* deficiency maintains the contractile phenotype of VSMCs.

### HINT1 effects on phenotypic switching of VSMCs via targeting Itga6.

**Figure 3:**
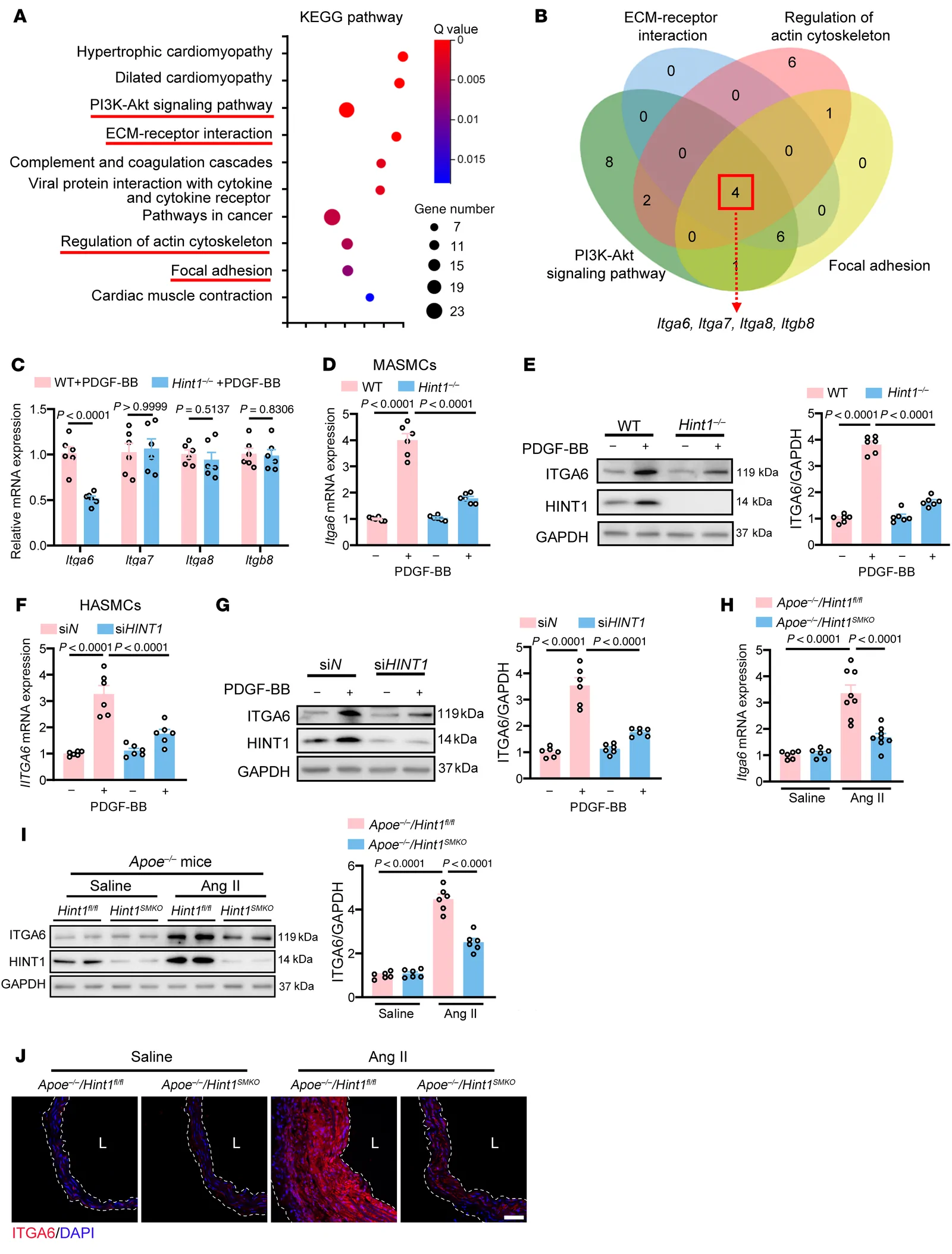
ITGA6 is the downstream target of HINT1. (**A**) Kyoto Encyclopedia of Genes and Genomes (KEGG) pathway analysis of differentially expressed genes in PDGF-BB–treated MASMCs isolated from the whole aorta of WT and *Hint1^–/–^* mice. (**B**) Venn diagram showing 4 overlapping targets that were all enriched in the related pathways (blue, ECM-receptor interaction; red, regulation of actin cytoskeleton; green, PI3K/AKT signaling pathway; and yellow, focal adhesion) in **A**. (**C**) q-PCR analysis of above 4 overlapping targets (*Itga6*, *Itga7*, *Itga8*, and *Itgb8*) in MASMCs isolated from the whole aortas of WT and *Hint1^–/–^* mice and treated with PDGF-BB (20 ng/mL) (*n* = 6 per group). (**D** and **E**) q-PCR (**D**) and Western blotting (**E**) analysis of *Itga6* in MASMCs isolated from the whole aortas of WT and *Hint1^–/–^* mice and treated with PBS or PDGF-BB (20 ng/mL) (*n* = 6 per group). (**F** and **G**) q-PCR (**F**) and Western blotting (**G**) analysis of *Itga6* in HASMCs transfected with si*N* or si*HINT1* followed by PBS or PDGF-BB (20 ng/mL) stimulation (*n* = 6 per group). (**H**–**J**) Eight-week-old male *Apoe^–/–^/Hint1^fl/fl^* and *Apoe^–/–^/Hint1^SMKO^* mice were infused with saline or Ang II (1,000 ng/kg/min) for 28 days. q-PCR (**H**) and Western blotting (**I**) analysis of the levels of *Itga6* in aortas. *n* = 6–8 per group. (**J**) Representative immunofluorescence staining of ITGA6 expression in abdominal aortas. Scale bar: 20 μm. L, lumen. Statistical analysis was performed by Student’s *t* test (**C**) or 1-way ANOVA (**D**–**I**). For all statistical plots, the data are presented as mean ± SEM.

To identify the downstream targets of HINT1 in phenotypic switching of VSMCs, we conducted RNA-Seq analysis (GEO GSE289426) to evaluate transcriptomic changes caused by deficiency of HINT1 in primary mouse aortic VSMCs stimulated with PDGF-BB. A total of 168 upregulated and 165 downregulated genes (fold change > 1.5, FDR < 0.05) were identified in VSMCs from *Hint1*^–/–^ mice stimulated with PDGF-BB compared with WT VSMCs. Kyoto Encyclopedia of Genes and Genomes pathway and enrichment analysis revealed that deficiency of HINT1 in VSMCs stimulated with PDGF-BB was related to 4 pathways, including PI3K/AKT signaling, ECM-receptor interaction, regulation of actin cytoskeleton, and focal adhesion pathways (Figure 3A). We screened 4 genes, including *Itga6*, *Itga7*, *Itga8*, and *Itgb8*, which were enriched in the above 4 pathways, suggesting that they may participate in phenotypic switching of VSMCs mediated by HINT1 (Figure 3B). Next, q-PCR was performed to verify the expression level of these genes in VSMCs from WT and *Hint1*^–/–^ mice treated with PDGF-BB. We demonstrated that *Hint1* deficiency caused reduced *Itga6* but had no effect on *Itga7*, *Itga8*, or *Itgb8*, with the treatment of PDGF-BB or Ang II (Figure 3C and Supplemental Figure 5A). Furthermore, we confirmed that either *Hint1* deficiency in MASMCs (Figure 3, D and E, and Supplemental Figure 5, B and C) or *HINT1* knockdown in HASMCs (Figure 3, F and G, and Supplemental Figure 5, D and E) resulted in a marked decrease of ITGA6, with the treatment of PDGF-BB or Ang II. Meanwhile, we observed reduced ITGA6 in the aortas from *Apoe^–/–^*/*Hint1^SMKO^* mice compared with that in the aortas from *Apoe^–/–^*/*Hint1^fl/fl^* mice infused with Ang II by q-PCR (Figure 3H), Western blot (Figure 3I), and immunofluorescence (Figure 3J). Collectively, these results indicate that HINT1 promotes phenotypic transformation of VSMCs via targeting ITGA6.

### ITGA6 promotes VSMC phenotypic switching.

**Figure 4:**
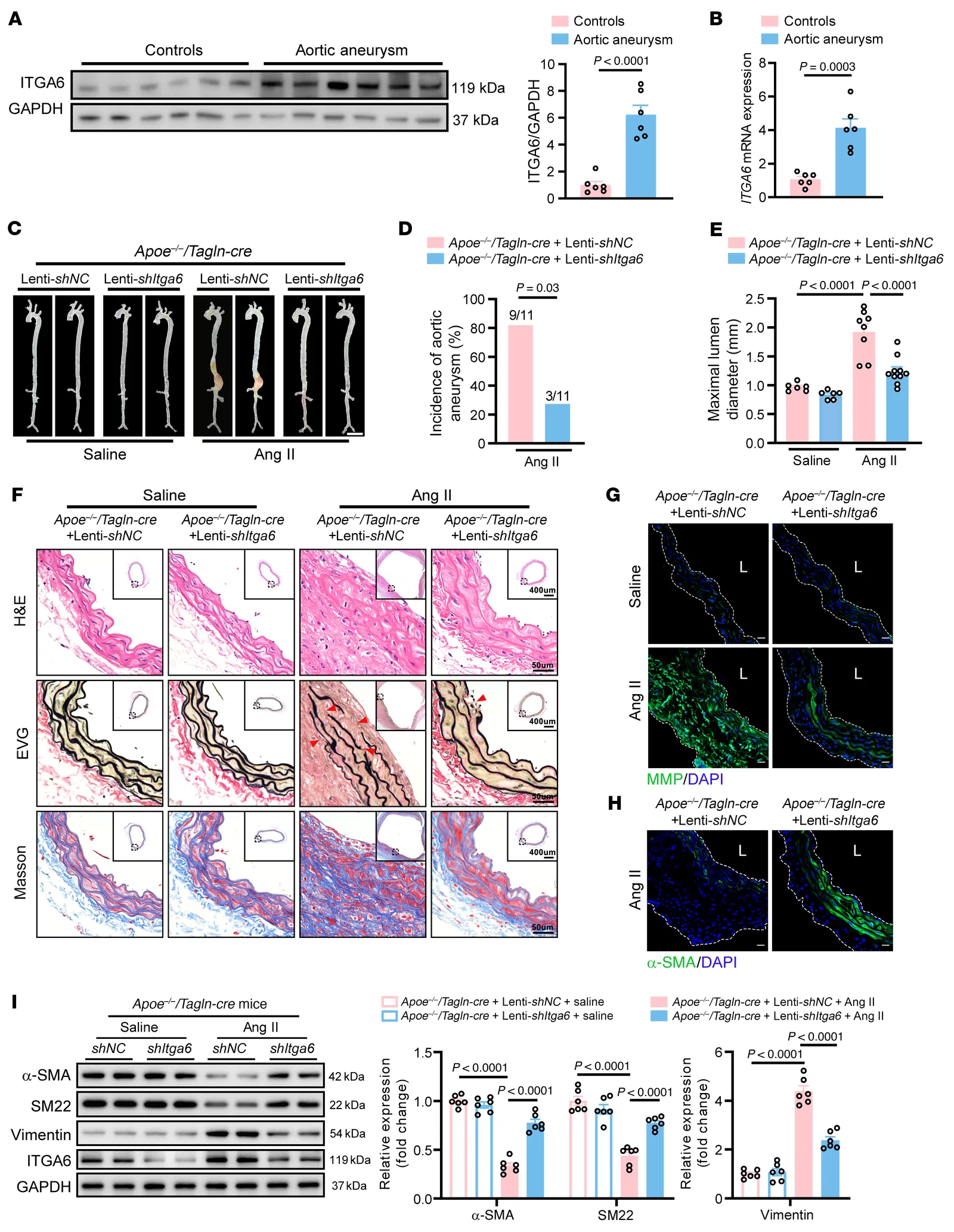
Knockdown of *Itga6* in VSMCs mitigates aortic aneurysm. (**A** and **B**) Western blotting (**A**) and q-PCR (**B**) analysis of ITGA6 in aorta samples from aortic aneurysm patients and normal aorta samples from donors (*n* = 6 per group). Six-week-old male *Apoe^–/–^/Tagln-cre* mice were injected with lentivirus vector encoding negative shRNA control (Lenti-sh*NC*) or lentivirus vector encoding shRNA targeting Itga6 (Lenti-*shItga6*) with reverse loxP sites, which can be recognized by Cre recombinase. After injection for 14 days, mice were infused with saline or Ang II (1,000 ng/kg/min) for 28 days. (**C**) Representative photograph of aortas from saline or Ang II–infused *Apoe^–/–^/Tagln-cre* mice infected with Lenti-sh*NC* or Lenti-sh*Itga6*. Scale bar: 2 mm. (**D**) Incidence of Ang II–induced aortic aneurysm. (**E**) Maximal abdominal lumen diameter in mice infused with saline (*n* = 6) or Ang II (*n* = 8–10), as measured by ultrasound. (**F**) Histopathological images of suprarenal abdominal aortas of saline- or Ang II–infused *Apoe^–/–^/Tagln-cre* mice infected with Lenti-sh*NC* or Lenti-sh*Itga6*. Scale bars: 50 μm and 400 μm (insets). EVG, elastic van Gieson staining. (**G**) Representative in situ zymography photomicrographs showing MMP activity of suprarenal abdominal aortas. Scale bars: 20 μm. L, lumen. (**H**) Representative immunofluorescence staining of α-SMA expression in suprarenal abdominal aortas. Scale bars: 20 μm. L, lumen. (**I**) Western blotting analysis of the VSMC contractile markers (α-SMA and SM22) and synthetic marker (Vimentin) in suprarenal abdominal aortas from saline- or Ang II–infused *Apoe^–/–^/Tagln-cre* mice infected with Lenti-sh*NC* or Lenti-sh*Itga6* (*n* = 6 per group). Statistical analysis was performed by Student’s *t* test (**A** and **B**), Fisher’s exact test (**D**), or 1-way ANOVA (**E** and **I**). For all statistical plots, the data are presented as mean ± SEM.

To explore the role of ITGA6 in phenotypic switching of VSMCs, we evaluated the expression of ITGA6 in VSMCs. We found increased ITGA6 in aorta samples from aortic aneurysm patients than that in normal aorta samples from donors by Western blot (Figure 4A) and q-PCR (Figure 4B). In addition, ITGA6 was upregulated in Ang II–infused *Apoe^–/–^* mice (Supplemental Figure 6, A–C). Besides, we also found an upregulation of ITGA6 in PDGF-BB–induced HASMCs, RASMCs, and MASMCs (Supplemental Figure 6, D and E). Moreover, we found that knockdown of *ITGA6* in VSMCs showed increased contractile proteins and decreased synthetic proteins (Supplemental Figure 6, F and G), which suggests that knockdown of *ITGA6* prevents phenotypic switching of VSMCs. In addition, phalloidin staining revealed that knockdown of *Itga6* inhibited microfilament remodeling and prevented morphological changes of VSMCs from a spindle-like contractile phenotype to a polygonal synthetic phenotype (Supplemental Figure 6H).

### ITGA6 in VSMCs aggravates Ang II–induced aortic aneurysm.

To clarify the role of ITGA6 in aortic aneurysm, we injected lentivirus vector encoding negative shRNA control (Lenti-sh*NC*) or lentivirus vector encoding shRNA targeting *Itga6* (Lenti-sh*Itga6*) with 2 reverse loxP sites, which can be recognized by Cre recombinase. These lentiviruses were injected into *Apoe^–/–^*/*Tagln-Cre* mice through the tail vein to specifically downregulate the *Itga6* level in VSMCs. Two weeks later, these mice were infused with Ang II or saline for 4 weeks (Supplemental Figure 7A), and ITGA6 level in the mouse aorta was confirmed to be downregulated (Supplemental Figure 7B). Gross examination showed lower luminal expansion in the suprarenal region of the abdominal aorta of Ang II–infused *Apoe^–/–^*/*Tagln-Cre* mice in which *Itga6* was reduced in VSMCs (Figure 4C). In addition, we showed that the knockdown of *Itga6* had no effect on BP, suggesting that the effects of ITGA6 on aortic aneurysm are not related to BP control (Supplemental Figure 7C). Silencing *Itga6* resulted in decreased incidence of aortic aneurysm (Figure 4D) and reduced aortic diameter (Figure 4E and Supplemental Figure 7D). The aortic rupture rate was 27.2% in Lenti-sh*NC* mice and 9.0% in Lenti-sh*Itga6* mice infused with Ang II (Supplemental Figure 7E). Morphologically, histological analysis results revealed that knockdown of *Itga6* in VSMCs mitigated arterial wall thickening and reduced elastic fiber degradation and collagen deposition in Ang II–administered mouse suprarenal abdominal aortas (Figure 4F). Moreover, we demonstrated that downregulation of *Itga6* decreased the activity of matrix metalloproteinase (Figure 4G) and increased α-SMA (Figure 4H) in aortic tissue. In addition, Western blot (Figure 4I) and q-PhCR (Supplemental Figure 7F) revealed that knockdown of *Itga6* led to an increase of contractile markers and a decrease of synthetic markers in the suprarenal abdominal aortas from Ang II–infused *Apoe^–/–^*/*Tagln-Cre* mice, which suggests that ITGA6 promotes VSMC phenotypic switching. Taken together, these results indicate that ITGA6 in VSMCs causes VSMC phenotypic switching and aggravates aortic aneurysms in vivo.

### Impact of HINT1 on aortic aneurysm relies on its regulation of ITGA6.

**Figure 5:**
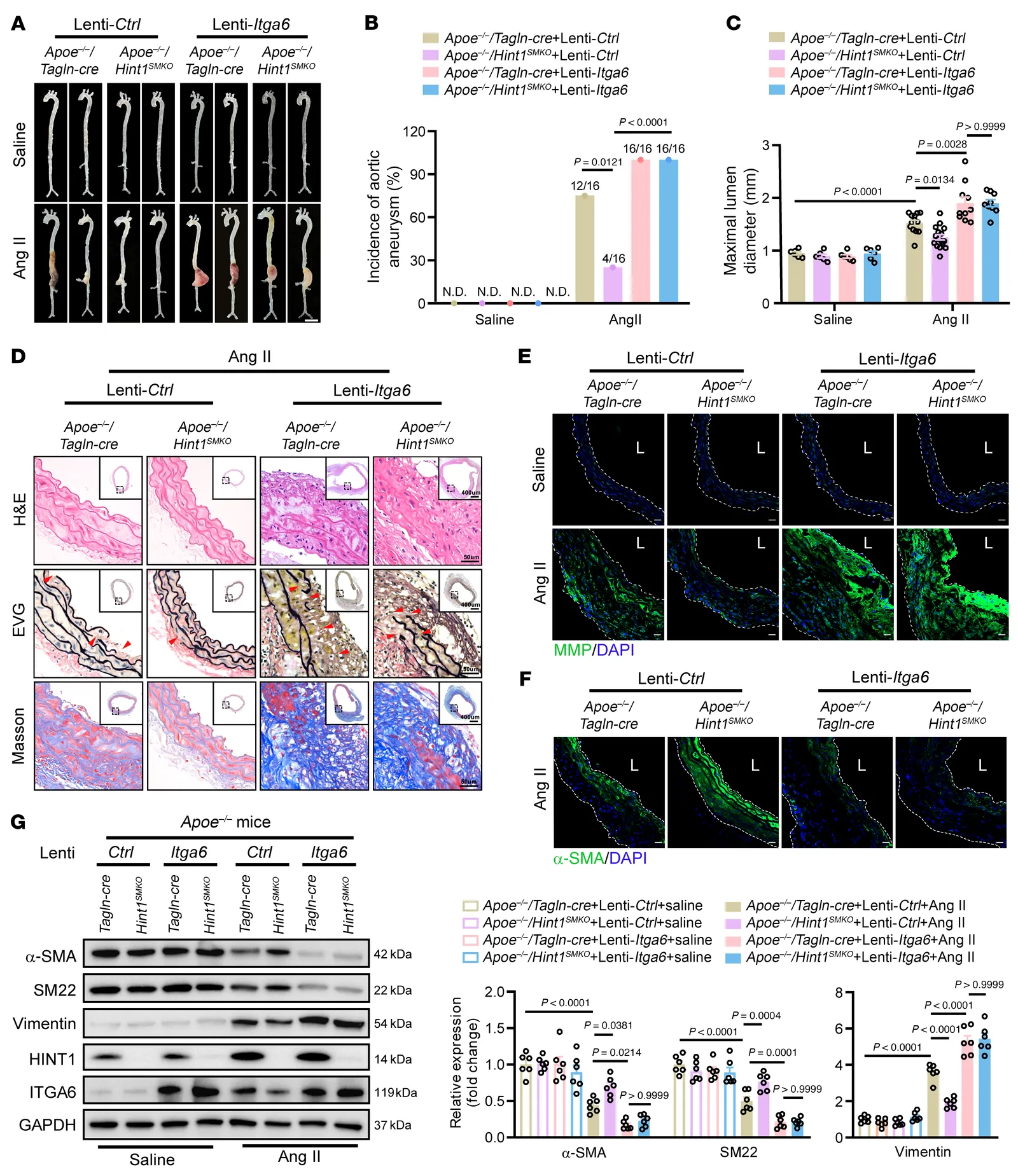
Impact of HINT1 on aortic aneurysm relies on its regulation of ITGA6 in vivo. Six-week-old male *Apoe^–/–^/Tagln-cre* and *Apoe^–/–^/Hint1^SMKO^* mice were injected with lentivirus vectors encoding control (Lenti-*Ctrl*) or *Itga6* (Lenti-*Itga6*) with reverse loxP sites, which can be recognized by Cre recombinase. After injection for 14 days, mice were infused with saline or Ang II (1,000 ng/kg/min) for 28 days. (**A**) Representative photograph of aortas from saline- or Ang II–infused *Apoe^–/–^/Tagln-cre or Apoe^–/–^/Hint1^SMKO^* mice infected with Lenti-*Ctrl* or Lenti-*Itga6*. Scale bar: 2 mm. (**B**) Incidence of Ang II–induced aortic aneurysm. N.D., not determined. (**C**) Maximal abdominal lumen diameter in mice infused with saline (*n* = 6) or Ang II (*n* = 9–16), as measured by ultrasound. (**D**) Histopathological images of suprarenal abdominal aortas of saline or Ang II–infused *Apoe^–/–^/Tagln-cre or Apoe^–/–^/Hint1^SMKO^* mice infected with Lenti-*Ctrl* or Lenti-*Itga6*. Scale bars: 50 μm; 400 μm (insets). EVG, elastic van Gieson staining. (**E**) Representative in situ zymography photomicrographs showing MMP activity of suprarenal abdominal aortas. Scale bars: 20 μm. L, lumen. (**F**) Representative immunofluorescence staining of α-SMA expression in suprarenal abdominal aortas. Scale bars: 20 μm. L, lumen. (**G**) Western blotting analysis of the VSMC contractile markers (α-SMA and SM22) and synthetic marker (Vimentin) in suprarenal abdominal aortas from saline- or Ang II–infused *Apoe^–/–^/Tagln-cre or Apoe^–/–^/Hint1^SMKO^* mice infected with Lenti-*Ctrl* or Lenti-*Itga6* (*n* = 6 per group). Statistical analysis was performed by Fisher’s exact test (**B**) or 2-way ANOVA (**C** and **G**). For all statistical plots, the data are presented as mean ± SEM.

To evaluate whether the effects of HINT1 on aortic aneurysm rely on its regulation of ITGA6 in vivo, we injected lentivirus vectors encoding control (Lenti-*Ctrl*) or *Itga6* (Lenti-*Itga6*) with reverse loxP sites, which can be recognized by Cre recombinase, into *Apoe^–/–^*/*Hint1^SMKO^* and *Apoe^–/–^*/*Hint1^fl/fl^* littermates through the tail vein to specifically overexpress *Itga6* in VSMCs. Two weeks later, these mice were infused with Ang II or saline for 4 weeks (Supplemental Figure 8A) and the BPs of mice were measured (Supplemental Figure 8B). As shown in Figure 5A, knockout of *Hint1* in VSMCs alleviated aortic aneurysm, and simultaneous overexpression of *Itga6* reversed this protective effect. Consistent with these findings, the incidence of aortic aneurysm (Figure 5B) and aortic diameter (Figure 5C and Supplemental Figure 8C) were substantially decreased in the *Apoe^–/–^*/*Hint1^SMKO^* mice, which were reversed by injection of Lenti-*Itga6*. We observed reduced aortic rupture rate caused by deficiency of *Hint1*; however, these beneficial effects were eliminated by overexpression of *Itga6* (Supplemental Figure 8D). Deficiency of *Hint1* in VSMCs reduced the elevation of aortic medial thickness, fragmentation of elastic fibers, and deposition of collagen in the suprarenal aorta; however, these beneficial effects were eliminated by overexpression of *Itga6* (Figure 5D). Meanwhile, VSMC-specific knockout of *Hint1* reduced MMP activity and increased α-SMA expression in the suprarenal aorta, while this phenomenon was diminished by overexpression of *Itga6* (Figure 5, E and F). Furthermore, overexpression of *Itga6* reversed the inhibition of deficiency of *Hint1* in VSMC phenotypic switching (Figure 5G and Supplemental Figure 8E).

To further confirm that the regulatory effects of HINT1 on VSMC phenotypic switching are ITGA6 dependent, *ITGA6* was overexpressed by lentivirus transfection when *HINT1* was silenced by siRNA in VSMCs. Western blotting and q-PCR showed that *ITGA6* overexpression reversed the reduction of phenotypic switching of VSMCs induced by *HINT1* knockdown (Supplemental Figure 8, F and G). We observed consistent results through phalloidin staining (Supplemental Figure 8H). These findings demonstrated that HINT1 promoted phenotypic switching of VSMCs via upregulating ITGA6 expression in vitro. Taken together, these results suggest that HINT1 in VSMC aggravates aortic aneurysm by increasing the expression of ITGA6.

### HINT1 regulates ITGA6 transcription via its interaction with TFAP2A.

**Figure 6:**
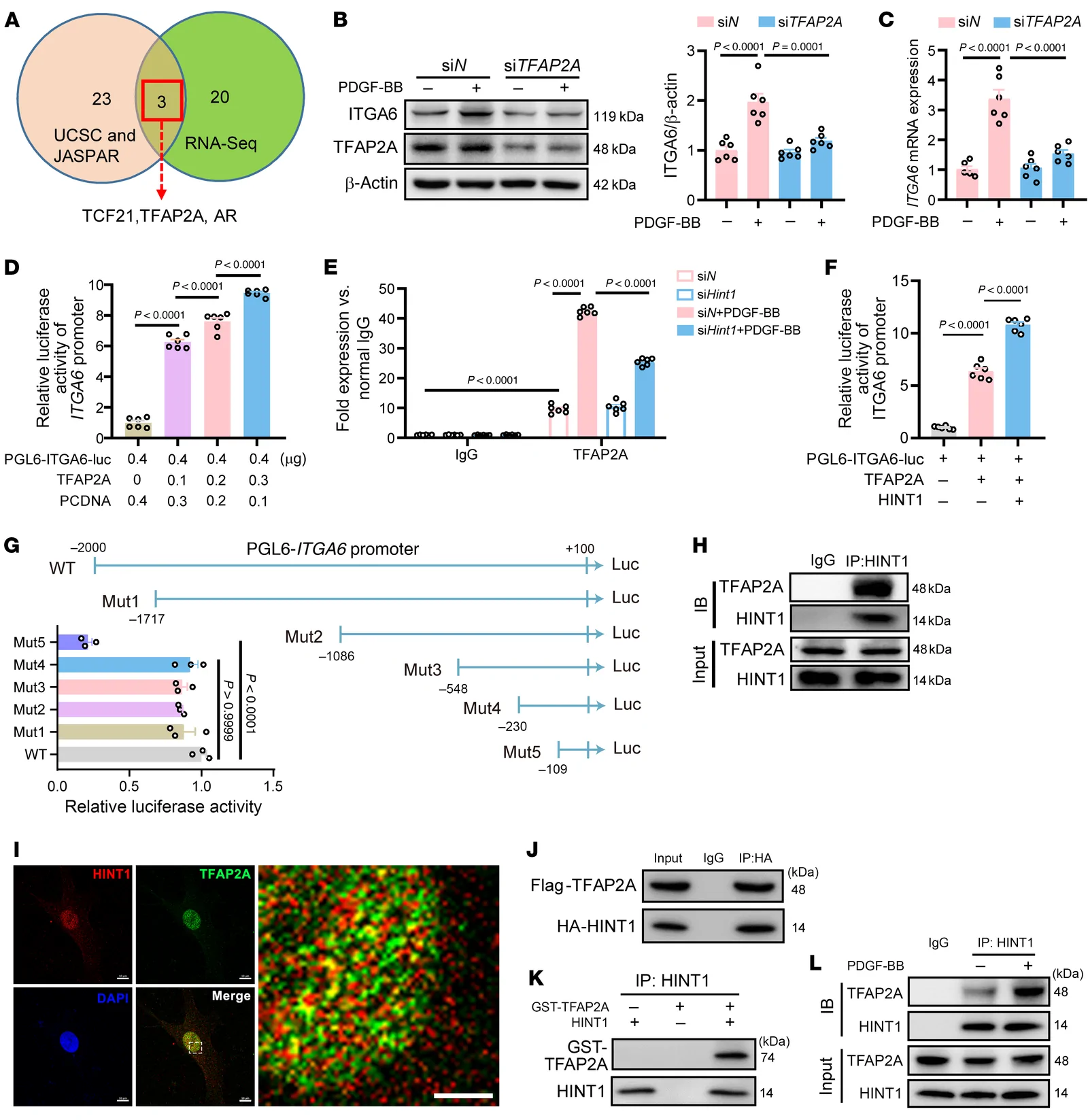
HINT1 regulates ITGA6 expression via its interaction with TFAP2A. (**A**) Venn diagram showing the potential transcription factors for ITGA6, which were regulated by HINT1. Pink, transcription factors for ITGA6 predicted by UCSC and JASPAR databases. Green, differentially expressed transcription factors in PDGF-BB–treated WT and *Hint1^–/–^* MASMCs detected by RNA-Seq mentioned in Figure 3A. (**B** and **C**) Western blotting (**B**) and q-PCR (**C**) analysis of ITGA6 expression in HASMCs that were transfected with si*N* or si*TFAP2A* followed by PBS or PDGF-BB stimulation (*n* = 6). (**D**) Luciferase reporter constructs with full-length *Itga6* promoter were cotransfected with TFAP2A plasmid or pcDNA into HEK293T cells, and luciferase activity was evaluated (*n* = 6). (**E**) ChIP assays of TFAP2A binding to the ITGA6 promoter in RASMCs transfected with si*N* or si*Hint1* and treated with PBS or PDGF-BB (*n* = 6). (**F**) Luciferase reporter constructs with full-length *Itga6* promoter were cotransfected with or without TFAP2A and HINT1 plasmid into HEK293T cells, and luciferase activity was evaluated (*n* = 6). (**G**) Luciferase activation driven by the WT *Itga6* promoter or mutant promoter (–1,717, –1,086, –548, –230, –109 up to +100) normalized to renilla luciferase in HEK293T cells (*n* = 3). (**H**) Identification of HINT1 and TFAP2A interaction in RASMCs by Co-IP (immunoprecipitated by HINT1 antibody). (**I**) Confocal fluorescence microscopy of HINT1 (red) and TFAP2A (green) in HASMCs. DAPI, blue. Scale bars: 10 μm (left); 5 μm (right). (**J**) HEK293T cells were cotransfected with Flag-TFAP2A and HA-HINT1 plasmids. Co-IP analysis of Flag-TFAP2A and HA-HINT1 interaction (immunoprecipitated by HA antibody). (**K**) In vitro binding assay of purified HINT1 and GST-TFAP2A protein (immunoprecipitated by HINT1 antibody). (**L**) Co-IP assay of HINT1 and TFAP2A interaction in HASMCs with PBS or PDGF-BB stimulation for 4 hours (immunoprecipitated by HINT1 antibody). Statistical analysis was performed by 1-way ANOVA (**B**–**D**, **F**, and **G**) or 2-way ANOVA (**E**). For all statistical plots, the data are presented as mean ± SEM.

To further address how HINT1 regulates ITGA6 transcription and expression, UCSC and JASPAR databases were combined and employed to identify transcription factors of *ITGA6*. Meanwhile, the transcription factors regulated by HINT1 were identified by RNA-Seq, as we performed above in Figure 3A. After cross-comparing these 2 datasets, we identified 3 transcription factors, including transcription factor 21 (TCF21), transcription factor AP-2 α (TFAP2A), and androgen receptor (AR), which may be the potential transcription factors for *ITGA6* and regulated by HINT1 (Figure 6A). Luciferase reporter assays showed that TFAP2A may be the transcription factor for *ITGA6* but not TCF21 or AR (Supplemental Figure 9A). Further, we observed reduced ITGA6 when *TFAP2A* was knocked down in HASMCs treated with PDGF-BB or Ang II (Figure 6, B and C, and Supplemental Figure 9, B and C). Luciferase reporter assays (Figure 6D) and ChIP assay (Supplemental Figure 9D) verified the binding of TFAP2A to the promoter of *ITGA6*, and PDGF-BB stimulation increhased this binding. The ChIP assay revealed that the knockdown of *Hint1* reduced PDGF-BB/Ang II–induced binding of TFAP2A to the *Itga6* promoter (Figure 6E and Supplemental Figure 9E). Luciferase reporter assays also demonstrated that overexpression of *HINT1* promoted *ITGA6* transcription mediated by TFAP2A in human embryonic kidney 293T (HEK293T) cells (Figure 6F). Next, we uncovered the motifs of the *ITGA6* promoter to which TFAP2A binds. According to the predicted binding sites of TFAP2A in the *ITGA6* promoter from the JASPAR library, WT and mutations (MUT1, MUT2, MUT3, MUT4, and MUT5) of *ITGA6* promoter-firefly luciferase reporter plasmids were constructed and transfected into HEK293T cells. Luciferase reporter assays showed that TFAP2A did not stimulate the activity of the MUT5-*ITGA6* promoter. These results indicate the promoter region between –230 to –109 bp is the TFAP2A binding site (Figure 6G).

Next, we aimed to investigate the mechanism by which HINT1 regulates *ITGA6* transcription via TFAP2A. HINT1, a transcription cofactor, has been reported to regulate a series of transcription factors by interacting with them, including USF2, MITF, and β-catenin (18, 19). We speculated that HINT1 can interact with TFAP2A and further regulate its activity as a transcription factor. To test our hypothesis, coimmunoprecipitation (Figure 6H) and immunofluorescence (Figure 6I) were performed. When both Flag-tagged TFAP2A and HA-tagged HINT1 were cotransfected into HEK293T cells, coimmunoprecipitation (Co-IP) with Flag or HA antibody showed that Flag-TFAP2A interacted with HA-HINT1 in cells (Figure 6J and Supplemental Figure 9F). Next, purified proteins of HINT1 and GST-TFAP2A were synthesized, and we observed that the 2 proteins could directly interact with each other in vitro (Figure 6K and Supplemental Figure 9G), which suggests that there is a direct interaction between HINT1 and TFAP2A. Furthermore, we found that PDGF-BB/Ang II stimulation increased the interaction between TFAP2A and HINT1 in HASMCs by Co-IP (Figure 6L and Supplemental Figure 9H) and immunofluorescence colocalization (Supplemental Figure 9I). Taken together, we demonstrated that HINT1 promotes ITGA6 transcription and expression via its enhanced interaction with TFAP2A.

### HINT1 enhances the interaction with TFAP2A by its increased nuclear translocation mediated by Nup98 under stimulation of PDGF-BB.

Next, we wondered why the interaction between HINT1 and TFAP2A was increased under pathological stimulus, which led to upregulated ITGA6 transcription mediated by TFAP2A. Given that TFAP2A is mostly located in the nucleus, as we observed and previously reported (23), we speculated that nuclear translocation of HINT1 is increased under stimulation of PDGF-BB, which leads to more interaction and colocalization of HINT1 and TFAP2A. As expected, we identified more nuclear translocation of HINT1 in PDGF-BB–treated HASMCs by Western blot (Supplemental Figure 10A) and immunofluorescence (Supplemental Figure 10B). To clarify why the nuclear translocation of HINT1 was increased under stimulation of PDGF-BB, the interacting proteins of HINT1 were pulled down by Co-IP using the antibody of HINT1 and identified by mass spectrometry (MS). The MS results showed that NUP98, an important component of the nuclear pore complex, could interact with HINT1 (Supplemental Figure 10C). The nuclear pore complex is a large protein channel in the nuclear membrane of cells that is responsible for the transport of substances between the nucleus and cytoplasm (24, 25). The interaction of endogenous HINT1 and NUP98 was confirmed by Co-IP in RASMCs (Supplemental Figure 10D) and HASMCs (Supplemental Figure 10E). Furthermore, we found that the knockdown of *NUP98* by siRNA reduced the nuclear translocation of HINT1 in PDGF-BB–treated HASMCs by Western blot (Supplemental Figure 10F). In addition, we observed less interaction between HINT1 and TFAP2A (Supplemental Figure 10G), along with decreased ITGA6 expression (Supplemental Figure 10, H and I) and attenuated phenotypic switching of VSMCs when *NUP98* was knocked down in PDGF-BB–treated HASMCs (Supplemental Figure 10, J and K). Taken together, we demonstrated that HINT1 enhances the interaction with TFAP2A by its increased nuclear translocation mediated by Nup98 under stimulation of PDGF-BB.

### ITGA6 exacerbates VSMC phenotypic switching via activating the FAK/STAT3 signal pathway.

**Figure 7:**
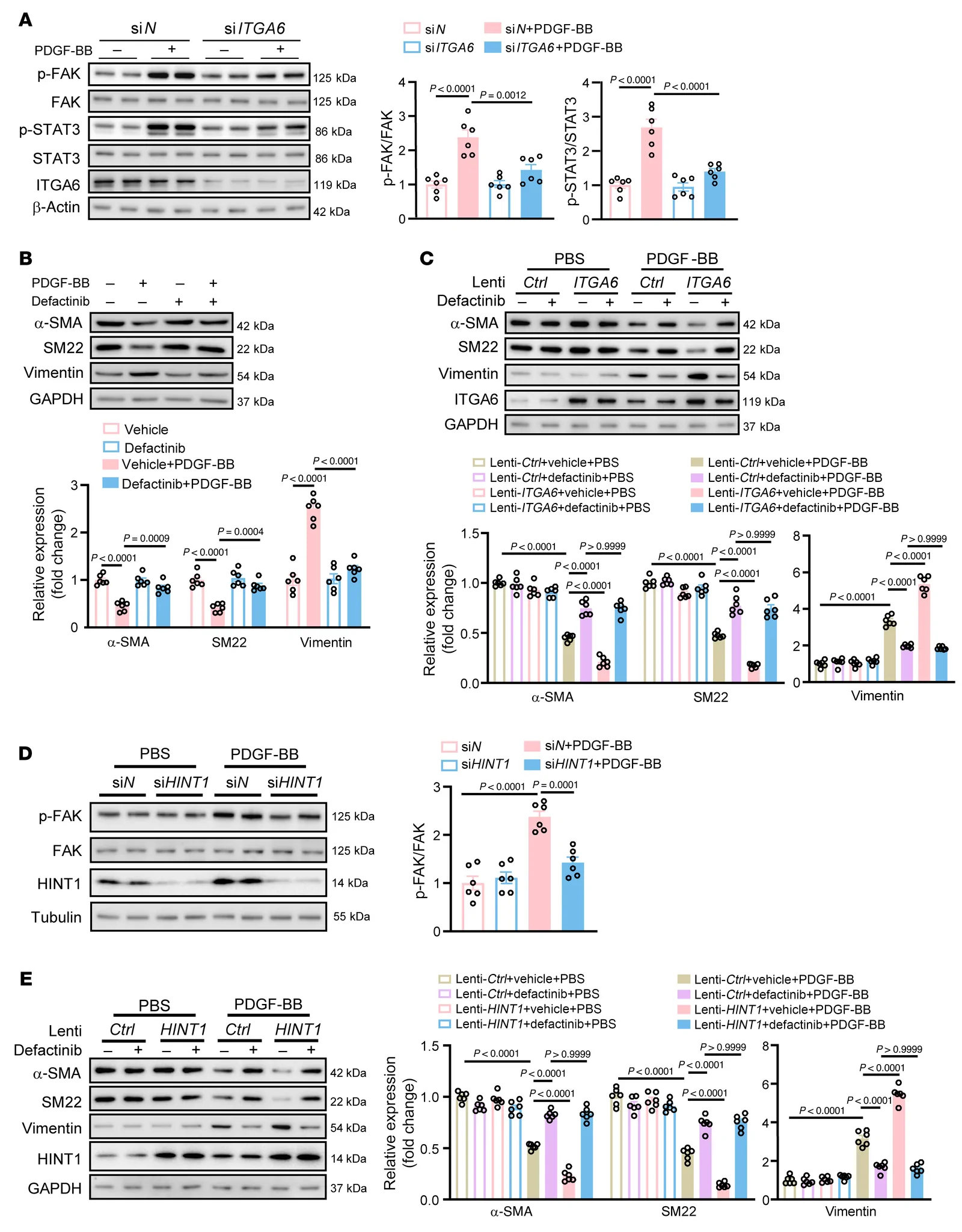
ITGA6 aggravates VSMC phenotypic switching via activating the FAK/STAT3 signal pathway. (**A**) Western blotting analysis of phosphorylation levels of FAK and STAT3 in HASMCs transfected with si*N* or si*ITGA6* followed by PBS or PDGF-BB (20 ng/mL) stimulation for 30 minutes (*n* = 6 per group). (**B**) Western blotting analysis of VSMC contractile markers (α-SMA and SM22) and synthetic marker (Vimentin) in HASMCs pretreated with or without defactinib (2.5 M) followed by PBS or PDGF-BB (20 ng/mL) stimulation (*n* = 6 per group). (**C**) Western blotting analysis of VSMC contractile marker (α-SMA and SM22) and synthetic marker (Vimentin) expression in HASMCs infected with Lenti-*Ctrl* or Lenti-*ITGA6* and pretreated with or without defactinib (2.5 M) followed by PBS or PDGF-BB (20 ng/mL) stimulation (*n* = 6 per group). (**D**) Western blotting analysis of phosphorylation levels of FAK in HASMCs transfected with si*N* or si*HINT1* followed by PBS or PDGF-BB (20 ng/mL) stimulation (*n* = 6 per group). (**E**) Western blotting analysis of VSMC contractile marker (α-SMA and SM22) and synthetic marker (Vimentin) expression in HASMCs infected with Lenti-*Ctrl* or Lenti-*HINT1* and pretreated with or without defactinib (2.5 M) followed by PBS or PDGF-BB (20 ng/mL) stimulation (*n* = 6 per group). Statistical analysis was performed by 1-way ANOVA (**A**, **B**, and **D**) or 2-way ANOVA (**C** and **E**). For all statistical plots, the data are presented as mean ± SEM.

Next, to investigate the mechanism underlying how ITGA6 impacts VSMC phenotype switching, a protein–protein interaction network of ITGA6-interacting proteins was constructed using the STRING database, and PTK2 was identified as a target involved with ITGA6 (Supplemental Figure 11A). PTK2 is the gene name of FAK. Previous studies reported that activated FAK could trigger the downstream STAT3 signaling pathway (26, 27), which is recognized to promote VSMC phenotype switching (28). Therefore, we speculated that ITGA6 affected VSMC phenotype switching via the FAK/STAT3 signal pathway. As expected, we observed reduced p-FAK and p-STAT3 levels in *ITGA6*-knockdown HASMCs (Figure 7A). In addition, a reduced nuclear translocation of STAT3 was confirmed when *ITGA6* was knocked down (Supplemental Figure 11B). Defactinib, an inhibitor of FAK (29), reduced FAK activation, downstream phosphorylation of STAT3 (Supplemental Figure 11C), and nuclear translocation of STAT3 (Supplemental Figure 11, D and E). Furthermore, we found that defactinib could inhibit VSMC phenotypic switching by Western blot (Figure 7B) and q-PCR (Supplemental Figure 11F). We further detected that defactinib treatment inhibited the FAK/STAT3 signal pathway (Supplemental Figure 11G) and nuclear translocation of STAT3 (Supplemental Figure 11H) induced by overexpression of ITGA6. Besides, we observed that overexpression of *ITGA6* promoted VSMC phenotypic switching, and defactinib treatment attenuated this effect, indicating that ITGA6 aggravates VSMC phenotypic switching via activating the FAK signal pathway (Figure 7C and Supplemental Figure 11, I and J). Furthermore, we observed reduced p-FAK in *Hint1*-silenced HASMCs (Figure 7D) and defactinib treatment–attenuated VSMC phenotypic switching induced by overexpression of *HINT1* (Figure 7E and Supplemental Figure 11K). Taken together, our findings suggest that ITGA6 aggravates VSMC phenotypic switching via activating the FAK/STAT3 signal pathway.

### Pharmacological blockade of FAK activation by defactinib protects against aortic aneurysm.

**Figure 8:**
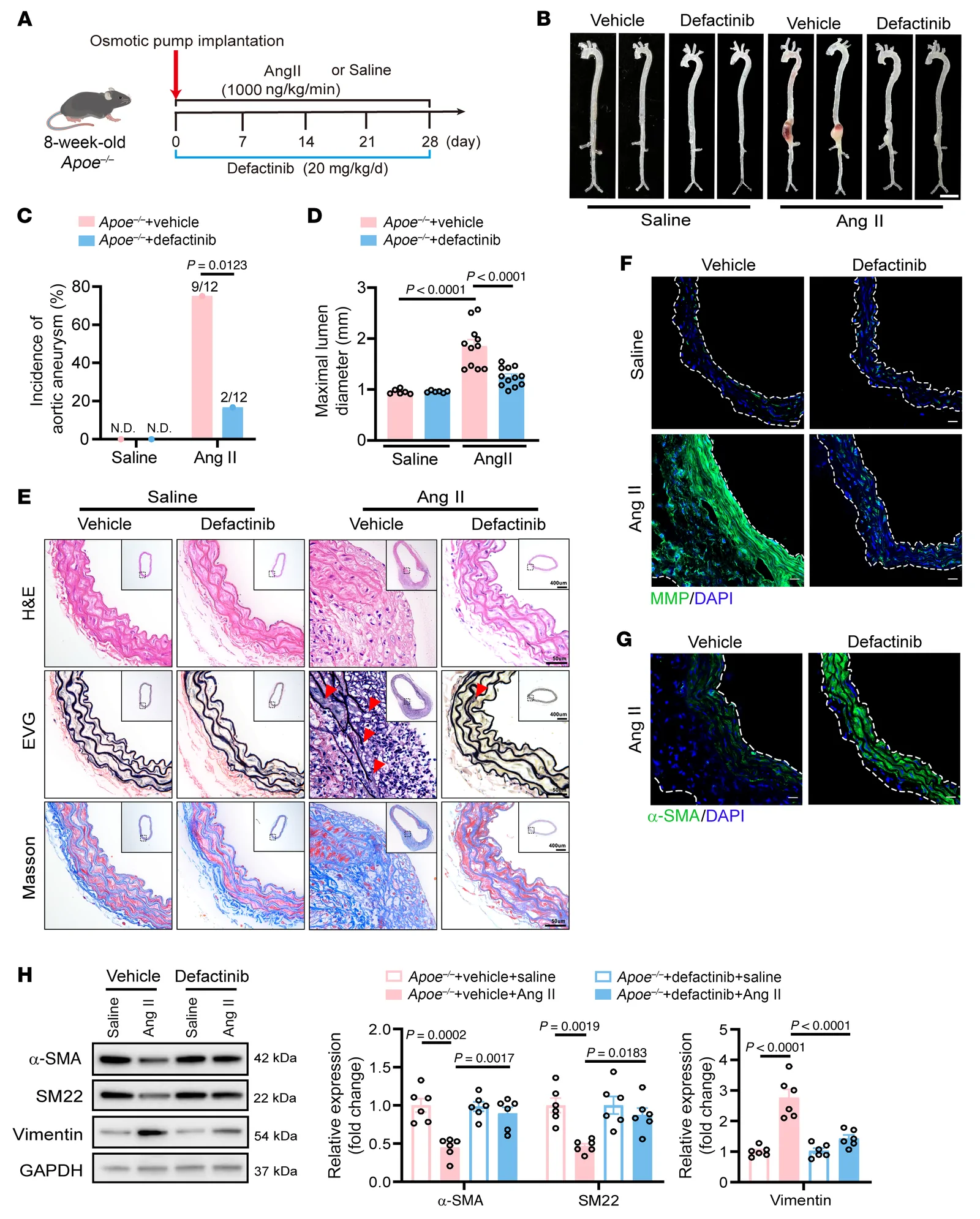
Defactinib protects against aortic aneurysm. (**A**) Eight-week-old male *Apoe^–/–^* mice were treated with defactinib (20 mg/kg/d) daily via intragastric administration, starting at the first day of Ang II infusion and continuing for 28 days. (**B**) Representative photograph of aortas from saline- or Ang II–infused *Apoe^–/–^* mice. Scale bar: 2 mm. (**C**) Incidence of saline- or Ang II–induced aortic aneurysm. N.D., not determined. (**D**) Maximal abdominal lumen diameter in mice infused with saline (*n* = 6) or Ang II (*n* = 11–12), as measured by ultrasound. (**E**) Histopathological images of suprarenal abdominal aortas of saline- or Ang II–infused *Apoe^–/–^* mice. Scale bars: 50 μm; 400 μm (insets). EVG, elastic van Gieson staining. (**F**) Representative in situ zymography photomicrographs showing MMP activity of suprarenal abdominal aortas. Scale bars: 20 μm. (**G**) Representative immunofluorescence staining of α-SMA expression in suprarenal abdominal aortas. Scale bars: 20 μm. (**H**) Western blotting analysis of the VSMC contractile markers (α-SMA and SM22) and synthetic markers (Vimentin) in suprarenal abdominal aortas from saline- or Ang II–infused *Apoe^–/–^* mice (*n* = 6 per group). Statistical analysis was performed by Fisher’s exact test (**C**) or 1-way ANOVA (**D** and **H**). For all statistical plots, the data are presented as mean ± SEM.

Eight-week-old male *Apoe^–/–^* mice were treated with defactinib (20 mg/kg/d) daily via intragastric administration, starting at the first day of Ang II infusion and continuing for 28 days (Figure 8A). Treatment with defactinib resulted in a marked reduction in the aortic diameter (Figure 8, B and D, and Supplemental Figure 12A) and decreased incidence (Figure 8C) of aortic aneurysm compared with controls. Treating *Apoe^–/–^* mice with defactinib did not cause aortic rupture after the Ang II infusion compared with controls (Supplemental Figure 12B). BP and body weight did not change in *Apoe^–/–^* mice treated with defactinib (Supplemental Figure 12, C and D). Hematoxylin and eosin staining revealed a marked reduction in the aortic medial thickness, and elastic van Gieson staining showed lower severe fragmentation of elastic fibers in the *Apoe^–/–^* mice that received defactinib (Figure 8E). Defactinib treatment caused decreased global MMP activity (Figure 8F) and increased α-SMA expression (Figure 8G) in suprarenal abdominal aortas. We observed alleviated VSMC phenotypic transformation in the suprarenal abdominal aortas of *Apoe^–/–^* mice that received defactinib by q-PCR (Supplemental Figure 12E) and Western blotting (Figure 8H). Overall, these data indicate the protective effects of defactinib against the progression of aortic aneurysm.

## Discussion

VSMCs show high plasticity and thus undergo phenotypic switching in response to various pathological stimuli. Recent studies have demonstrated that VSMC phenotypic switching is important in the pathogenesis of a variety of cardiovascular diseases, such as atherosclerosis, postinjury restenosis, and aortic aneurysm/dissection (30–32). In contrast, our understanding of the key factors that regulate VSMC phenotypic switching is limited.

In the present work, we identified the role of HINT1 in VSMC phenotypic switching and the pathogenesis of aortic aneurysms. The contributions included the following: (a) Hint1 deficiency in VSMCs alleviates aortic aneurysm formation and progression; (b) the driving role of HINT1 in aortic aneurysm is dependent on ITGA6; (c) ITGA6 promotes aortic aneurysm by activating the downstream FAK/STAT3 signal pathway; and (d) HINT1 upregulates ITGA6 transcription by interacting with and activating TFAP2A, which is the transcription factor of ITGA6. Taken together, these findings demonstrated that HINT1 promotes VSMC phenotypic switching and aggravates aortic aneurysm in an ITGA6-dependent manner through direct interaction with TFAP2A, which is responsible for the transcriptional activation of ITGA6.

Our previous study identified the role of HINT1 in cardiovascular diseases, in which HINT1 in cardiomyocytes protects from cardiac hypertrophy as an important signal transduction player in cytoplasm (20). Mechanically, HINT1 inhibits PKCb1 activation by interacting with PKCb1 and suppresses the downstream HOXA5 expression through the MEK/ERK/YY1 signal pathway. Other studies have reported that as a transcription cofactor, HINT1 can regulate the activity of a variety of transcription factors, including MITF, NF-KB, and USF2 (18, 19). Here, we determined that HINT1 undergoes nuclear translocation as a transcription cofactor of TFAP2A, directly interacts with TFAP2A, and increases the transcriptional activity of ITGA6, resulting in the activation of the downstream FAK/STAT3 signal pathway and promoting VSMC phenotypic switching and aortic aneurysms (Supplemental Figure 13).

As a member of the integrin family, ITGA6 plays important roles in proliferation, migration, and drug resistance. Studies found that downregulation of ITGA6 or specific ITGA6-neutralizing antibody treatment inhibits acute lymphoblastic leukemia invasion to the central nervous system (33). Increased ITGA6 expression in human endothelial progenitor cells contributes to angiogenesis (34). The ITGA6 splice variant regulates proliferation and the Wnt/β-catenin pathway (35). However, the role of ITGA6 in VSMC phenotypic switching or aortic aneurysms has not been elucidated. Our study demonstrated that ITGA6 is a target of HINT1 and promotes VSMC phenotypic switching by coupling with the downstream FAK/STAT3 pathway.

FAK is a nonreceptor tyrosine kinase that classically transduces signaling from cell adhesions to regulate multiple biological cellular functions, including cell survival, migration, and invasion of cancer cells (36, 37). FAK is indeed critical in development, tissue regeneration, and wound healing (38). In the vascular system, FAK plays pivotal roles in the vasculature development through the regulation of SMC recruitment and endothelial vascular network formation (39, 40). Recent studies have revealed that FAK plays a key role in angiogenesis and vascular development (41). FAK signal activation has been proved to be involved with VSMC phenotypic switching (28, 42).

Our current study found that FAK activation mediated by ITGA6 could promote VSMC phenotypic switching. To confirm our findings, defactinib, an effective inhibitor of FAK, was used. We observed markedly blunt VSMC phenotypic switching and limited aortic aneurysm under treatment of defactinib. Defactinib is currently being investigated in combination with the PD-1 receptor inhibitor pembrolizumab in patients with pancreatic, non–small cell lung, and mesothelioma cancer (29, 43). Its safety has been confirmed. Based on the effective intervention effect on aortic aneurysms we observed in animal models, defactinib may have potential for the treatment of aortic aneurysms in the near future.

## Methods

### Sex as a biological variable.

Our study exclusively examined male mice because aortic aneurysm is a sex dimorphic disease and aortic aneurysm exhibits lower female prevalence (44, 45). It is unknown whether the findings are relevant for female mice.

### Animals.

*Hint1^fl/fl^* mice were generated using the CRISPR/Cas9 system at the Model Animal Research Center of Nanjing Medical University. *Tagln-Cre* mice were purchased from the Model Animal Research Center of Nanjing University. *Hint1^fl/fl^* mice were crossed with *Tagln-Cre* mice to generate *Hint1^SMKO^* mice. *Apoe^–/–^* mice were purchased from GemPharmatech. *Apoe^–/–^* mice were crossed with *Tagln-Cre* mice to generate *Apoe^–/–^/Tagln-cre* mice. *Hint1^–/–^* mice were acquired from the Shanghai Key Laboratory of Regulatory Biology, Institute of Biomedical Sciences and School of Life Sciences, East China Normal University. C57BL/6J mice were purchased from the Animal Core Facility of Nanjing Medical University. Mice were housed in a pathogen-free and temperature-controlled environment under a 12-hour light/dark cycle. Comparisons were made between littermates.

### Cell culture.

RASMCs were isolated from the aortas of male Sprague-Dawley rats (weight 150–180 g) by collagenase digestion as previously described (30). MASMCs were isolated from the thoracic aortas of 4- to 6-week-old male WT mice and *Hint1^–/–^* mice as previously described (46). HASMCs (CTCC-001-0065) were purchased from MEISEN CELL. HEK293T cells were from Cell Bank/Stem Cell Bank, Chinese Academy of Sciences.

### Western blot analysis.

Cells or tissues were lysed in RIPA lysis buffer (Thermo Fisher Scientific) with the addition of protease inhibitor (Roche) and phosphatase inhibitor (4906845001; Roche) cocktails. Protein samples were subjected to SDS-PAGE and transferred onto PVDF membranes. After blocking with 5% fat-free milk, the membrane was incubated with indicated primary antibodies at 4°C overnight. After TBST washing, membranes were incubated with secondary antibody for 1 hour at room temperature, and the bands were obtained by chemiluminescence (GE HealthCare).

### Immunofluorescence staining.

OCT-embedded aortic sections or cultured HASMCs or RASMCs were fixed with 4% paraformaldehyde for 20 minutes at room temperature. The samples were permeabilized with 0.1% Triton X-100 for 10 minutes, then blocked with 10% BSA for 1 hour. Next, anti-HINT1, anti–α-SMA, anti-ITGA6, anti-TFAP2A, or anti-STAT3 antibody was added to the sections or cells overnight at 4°C. After rinsing with PBS 3 times, the samples were incubated with Alexa Fluor 594 or 488 (1:500; Thermo Fisher Scientific) for 2 hours in darkness at room temperature. Nuclei were counterstained with DAPI (0100-20; SouthernBiotech). Staining was visualized using a confocal microscope (LSM800; Carl Zeiss).

### Lentivirus-mediated overexpression.

Mouse Itga6 cDNA was amplified by PCR and cloned into the pLVX-FLEX-EF1a-ZsGreen lentiviral vector. The correct sequence of the Itga6 gene in this construct was verified by sequencing. We cloned the expression cassette in an inverse, antisense orientation between 2 different loxP sites. The construct was designed so that Cre induction could be used to mediate the inversion of the Itga6 cassette into a sense orientation. Control or Itga6 lentivirus (10^9^ transducing units per mouse) was injected into *Apoe^–/–^/Tagln-Cre* or *Apoe^–/–^/Hint1^SMKO^* mice via the tail vein, in which Cre induction mediated an initial flipping based on orientation and location of 2 loxP sites.

### ChIP.

ChIP was performed using a ChIP Assay Kit (Beyotime) according to the manufacturer’s protocol. Briefly, 1% formaldehyde was added to fix cells at 37°C for 10 minutes, followed by neutralization using 125 mmol/L glycine. The cells were then washed with cold PBS and lysed on ice in SDS lysis buffer supplemented with proteinase inhibitor. The lysates were sonicated on ice. After centrifugation, the supernatant was collected and the chromatin in the supernatant was immunoprecipitated with anti-TFAP2A antibody (PA5-17359; Invitrogen) or IgG (control) incubation at 4°C overnight. Protein A/G beads were added and incubated for 2 hours at 4°C. After reversing the cross-links, DNA was isolated and used for PCR reactions. The primers used for ChIP assay are listed in Supplemental Table 3.

### Statistics.

All values are presented in the figures as the mean ± SEM, with *P* < 0.05 considered statistically significant. Fisher’s exact test was used for the incidence statistics of aortic aneurysms. For the comparison of the means between 2 groups, the Levene test was applied to evaluate the homogeneity of variance. An unpaired 2-tailed Student’s *t* test was used when data showed equal variance; otherwise, a *t* test assuming unequal variance was applied. For comparisons among more than 2 groups, a Brown-Forsythe test was used to evaluate homogeneity of variance. If the data showed equal variance, 1-way ANOVA was used followed by a post hoc analysis using the Bonferroni method to adjust for multiple comparisons; otherwise, a Welch’s 1-way ANOVA test was performed followed by a post hoc analysis using the Tamhane T2 method. Two-way ANOVA followed by Tukey’s multiple-comparison test for post hoc comparisons was used when appropriate. Two-way ANOVA with mixed effects was used for comparing the BP of mice that were repeatedly measured over time, at 0–28 days after osmotic pump implantation. Statistical results and the corresponding methods are presented in figure legends. All statistical analyses were performed and graphs were generated using GraphPad Prism 9.

Further information can be found in Supplemental Methods.

### Study approval.

The use of human aortic tissue was approved by the medical ethics committee of Nanjing Drum Tower Hospital following the Declaration of Helsinki. Written informed consent was provided by all participants or the organ donors’ legal representatives before enrollment. All animal experiments were conducted in accordance with the ARRIVE guidelines for the care and use of laboratory animals and with approval of the Nanjing Medical University Animal Care and Use Committee.

### Data availability.

Values for all data points in graphs are reported in the Supporting Data Values file. The RNA-Seq data reported in this study have been deposited in the GEO database (GSE289426).

## Author contributions

YJ, LX, Yi Han, and YZ developed the concept, designed the study, and revised the manuscript. YZ and WW analyzed the data and drafted the manuscript. YZ, WW, XY, SL, XW, QD, KY, LH, and SS performed the experiments. XD, ZH, and XL provided clinical samples. YJ, LX, Yaling Han, FC, AG, LW, ZZ, BY, CY, and Yi Han supervised the study. The order of co–first authors was determined by the volume of work each contributed to the study.

## Supplementary Material

Supplemental data

Unedited blot and gel images

Supporting data values

## Version 1 04/08/2025

In-Press Preview

## Version 2 06/02/2025

Electronic publication

## Version 3 11/13/2026

Corrected errors in the quantitative data and supporting data values for Supplemental Figures 3G, 6F, and 11H.
