# Corrigendum to HINT1 aggravates aortic aneurysm by targeting ITGA6/FAK axis in vascular smooth muscle cells

**DOI:** 10.1172/JCI213937

**Published:** 2026-11-16

**Authors:** Yan Zhang, Wencheng Wu, Xuehui Yang, Shanshan Luo, Xiaoqian Wang, Qiang Da, Ke Yan, Lulu Hu, Shixiu Sun, Xiaolong Du, Xiaoqiang Li, Zhijian Han, Feng Chen, Aihua Gu, Liansheng Wang, Zhiren Zhang, Bo Yu, Chenghui Yan, Yaling Han, Yi Han, Liping Xie, Yong Ji

Original citation: *J Clin Invest*. 2025;135(11):e186628. https://doi.org/10.1172/JCI186628

Citation for this corrigendum: *J Clin Invest*. 2026;136(22):e213937. https://doi.org/10.1172/JCI213937

The authors recently identified errors in the quantitative data and supporting data values for Supplemental Figures 3G, 6F, and 11H, and in the statistical analysis for Supplemental Figures 3G, 6F, and 11H. The original blot data underlying Supplemental Figures 6F and 11H were correct; the error was confined to quantitative data for SM22 in Supplemental Figure 6F and the cytoplasm data in Supplemental Figure 11H. In Supplemental Figure 3G, the original CT data for CNN1 were correct but the quantitative data for CNN1 were incorrect. The online version of the supplemental materials and the Supporting Data Values file has been corrected based on the original source data.

The authors regret the errors.

## Supplementary Material

Supplemental data

Supporting data values

## Version 1 11/16/2026

Electronic publication

