# Supplementary material for: HINT1 aggravates aortic aneurysm by targeting ITGA6/FAK axis in vascular smooth muscle cells
Source: J Clin Invest. 2025 Apr 8;135(11):e186628. doi: 10.1172/JCI186628 (PMC12126226; doi:10.1172/JCI186628)
Supplement: Unedited blot and gel images [file jci-135-186628-s297.pdf]

Full unedited gel for Figure 1B n=6

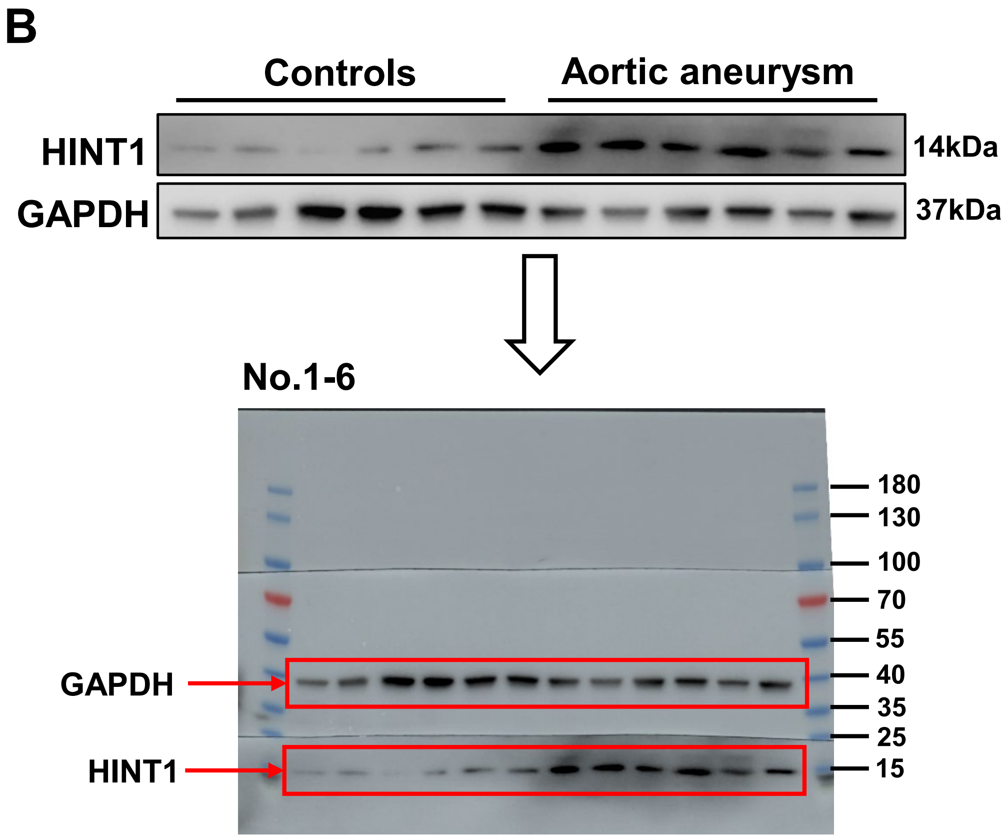

Full unedited gel for Figure 1E n=6

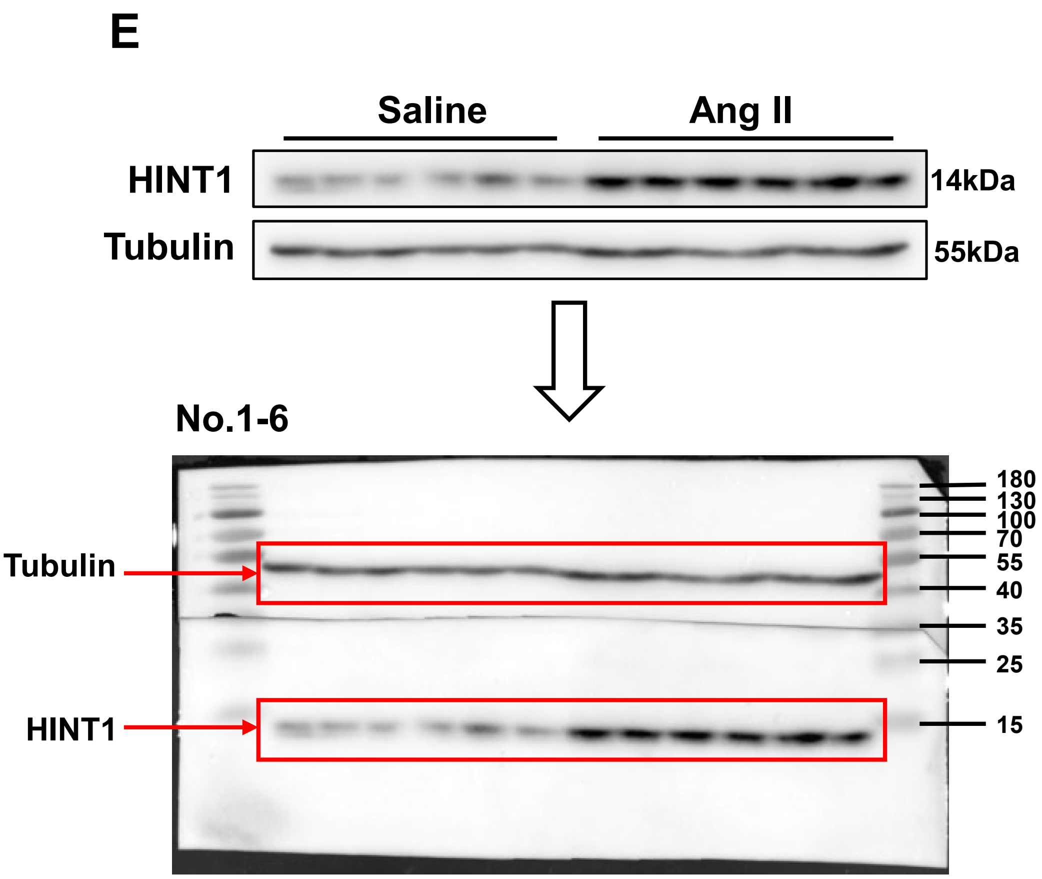

Full unedited gel for Figure 1H n=6

MASMCs

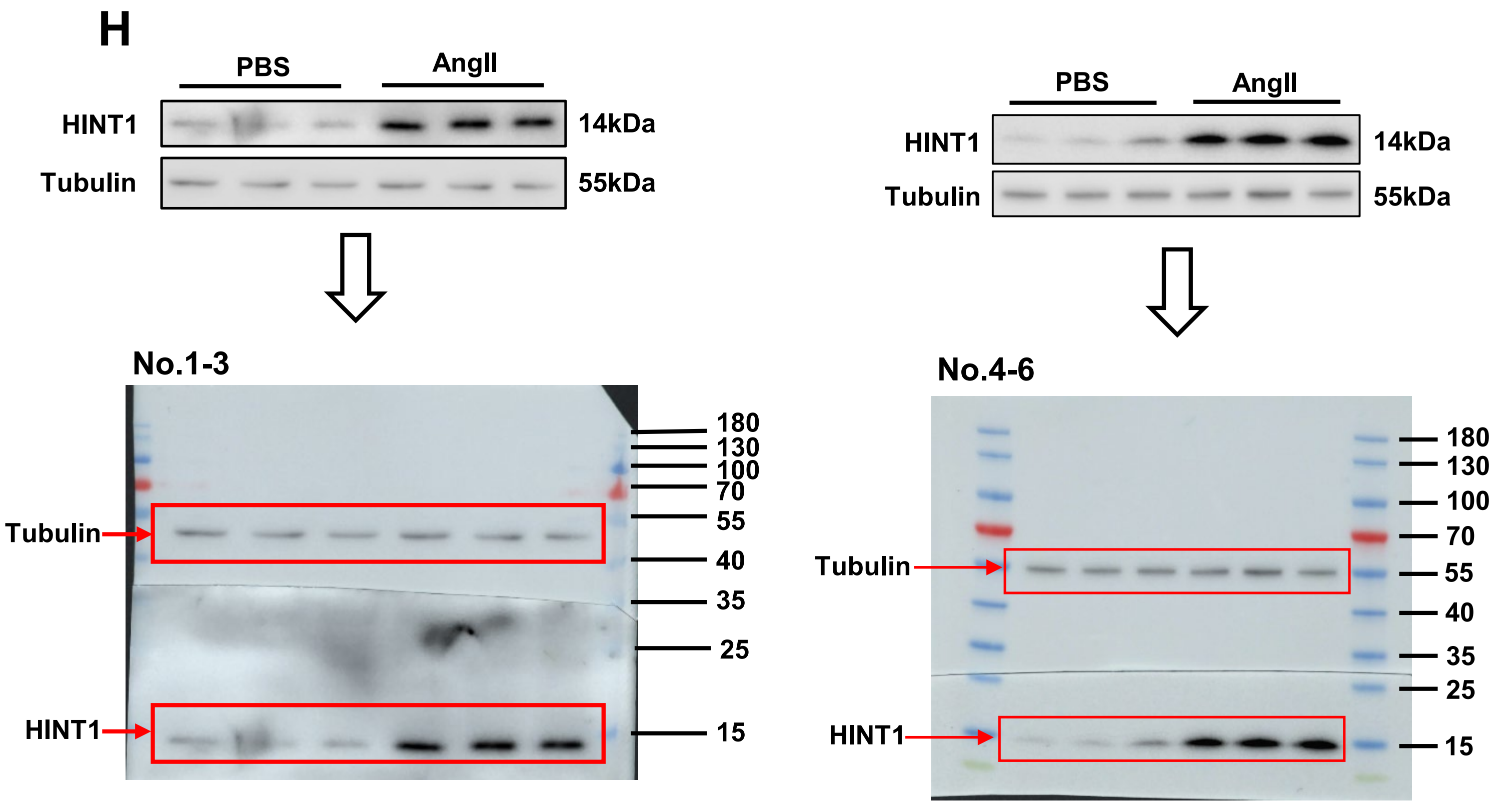

HASMCs

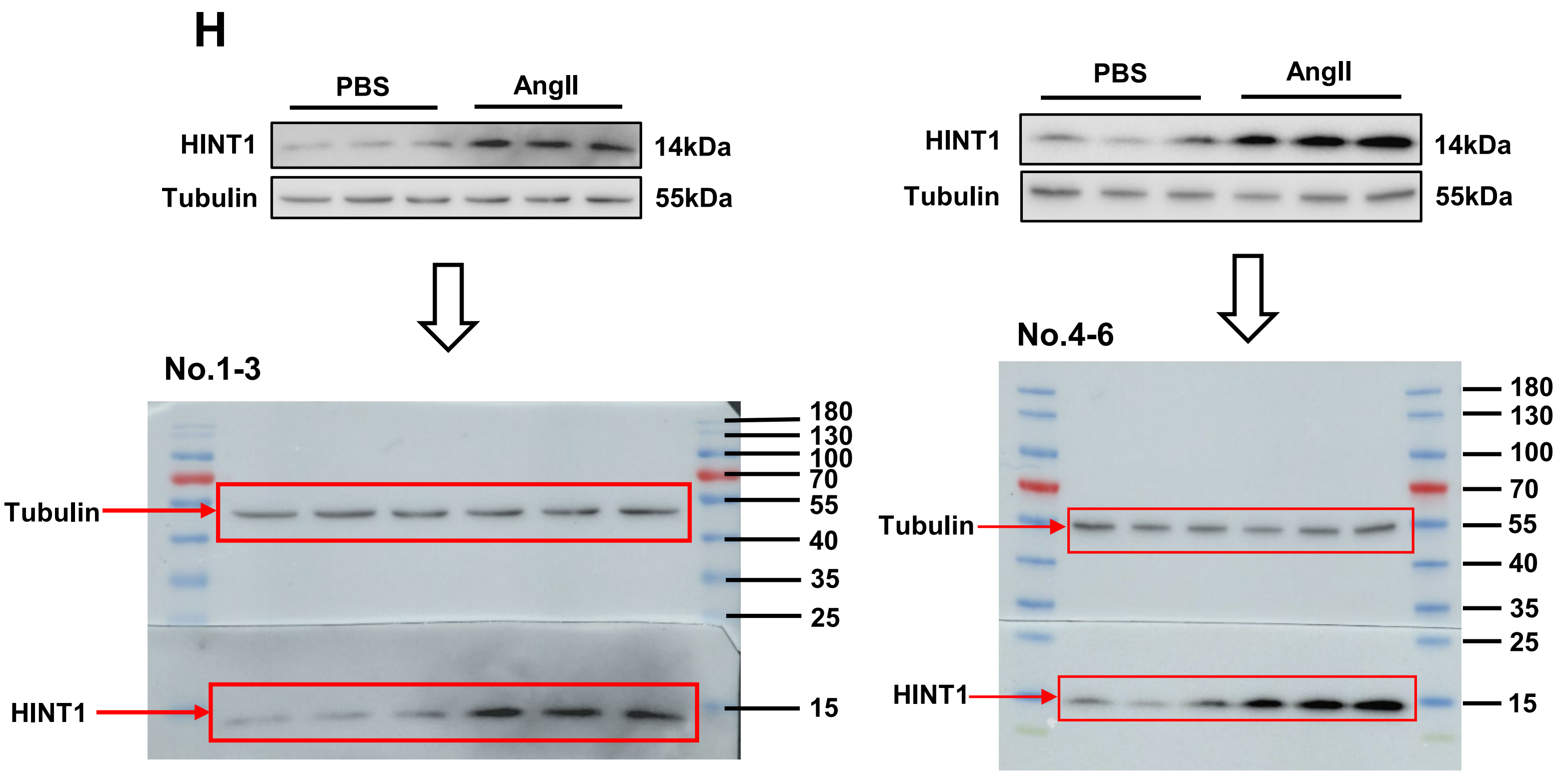

RASMCs

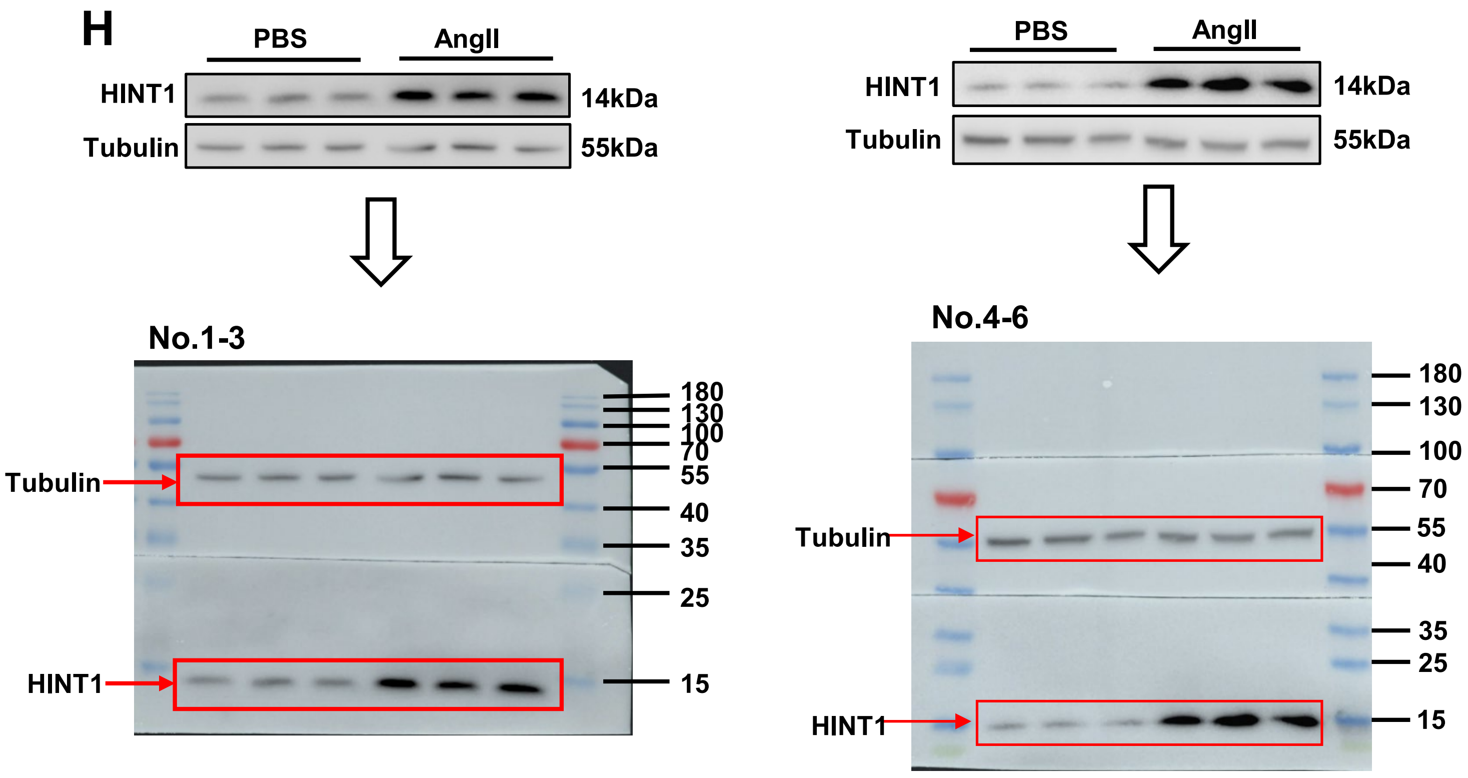
H
PBS
AngII
HINT1
Tubulin
14kDa
55kDa
No.1-3
Tubulin
HINT1
180
130
100
70
55
40
35
25
15
No.4-6
Tubulin
HINT1
180
130
100
70
55
40
35
25
15

Full unedited gel for Figure 2G n=6

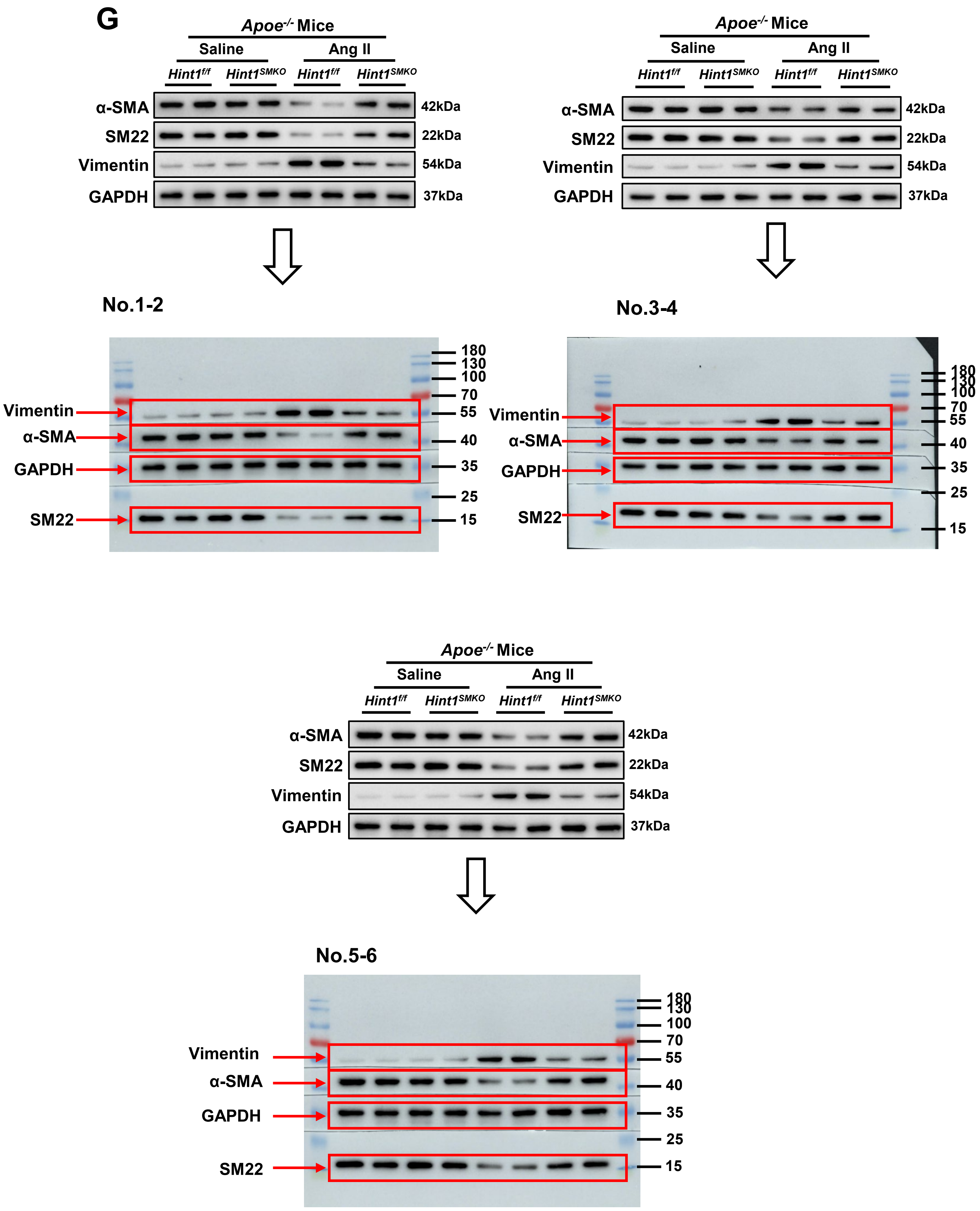

Full unedited gel for Figure 3E n=6

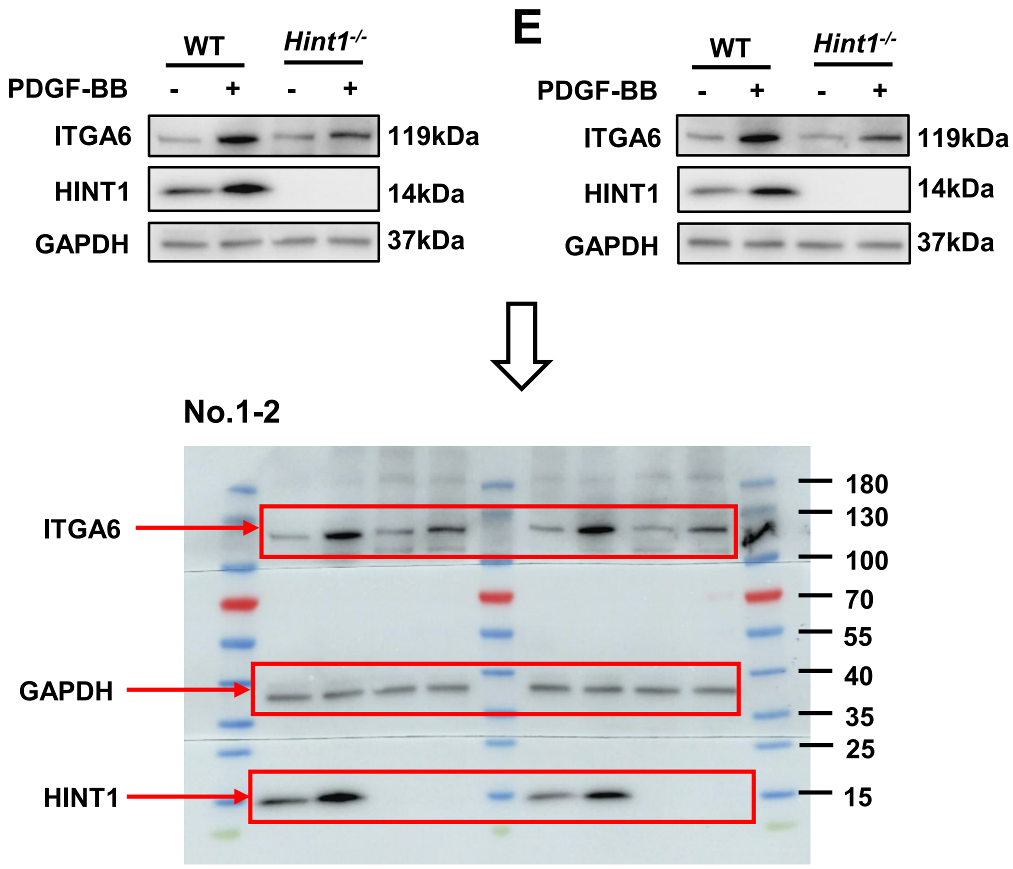

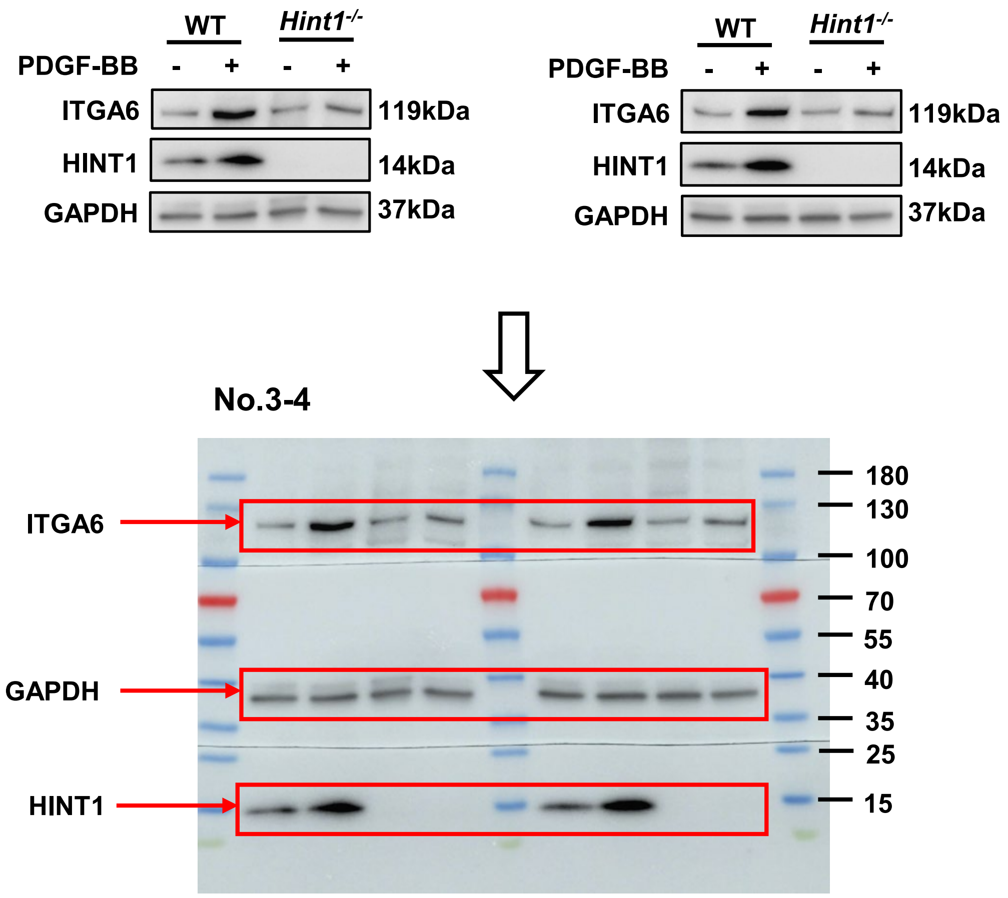

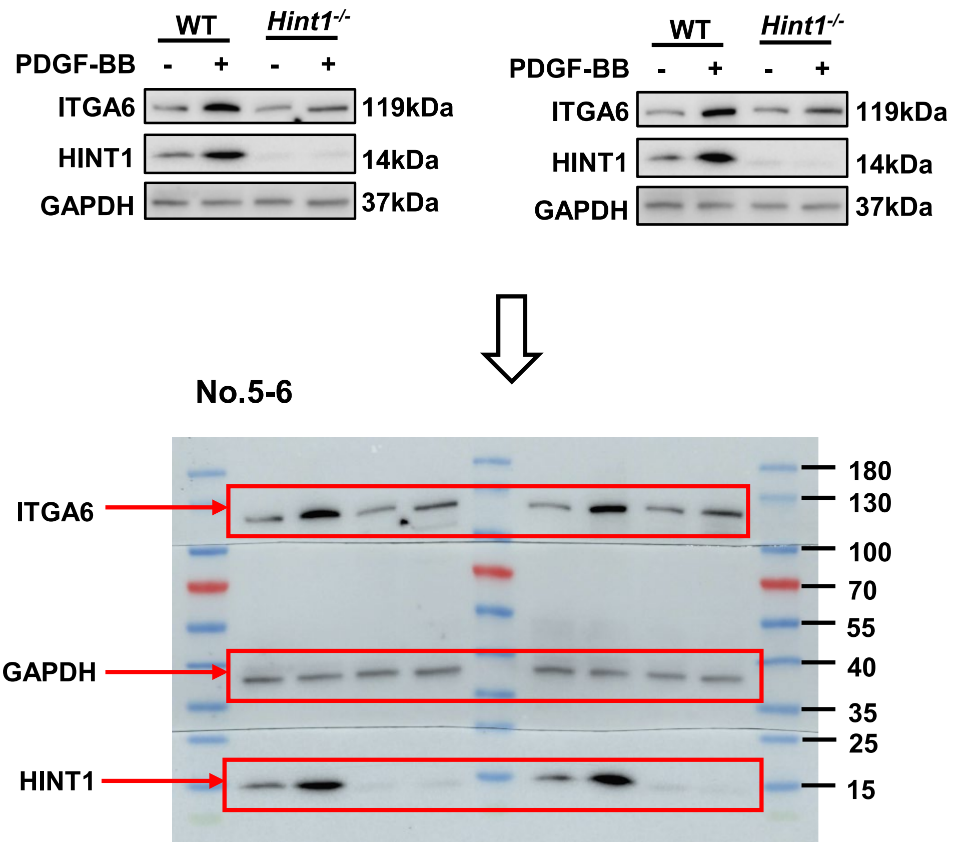
WT
Hint1-/-
PDGF-BB - + - +
ITGA6
119kDa
HINT1
14kDa
GAPDH
37kDa
WT
Hint1-/-
PDGF-BB - + - +
ITGA6
119kDa
HINT1
14kDa
GAPDH
37kDa
No.5-6
ITGA6
GAPDH
HINT1
180
130
100
70
55
40
35
25
15

Full unedited gel for Figure 3G n=6

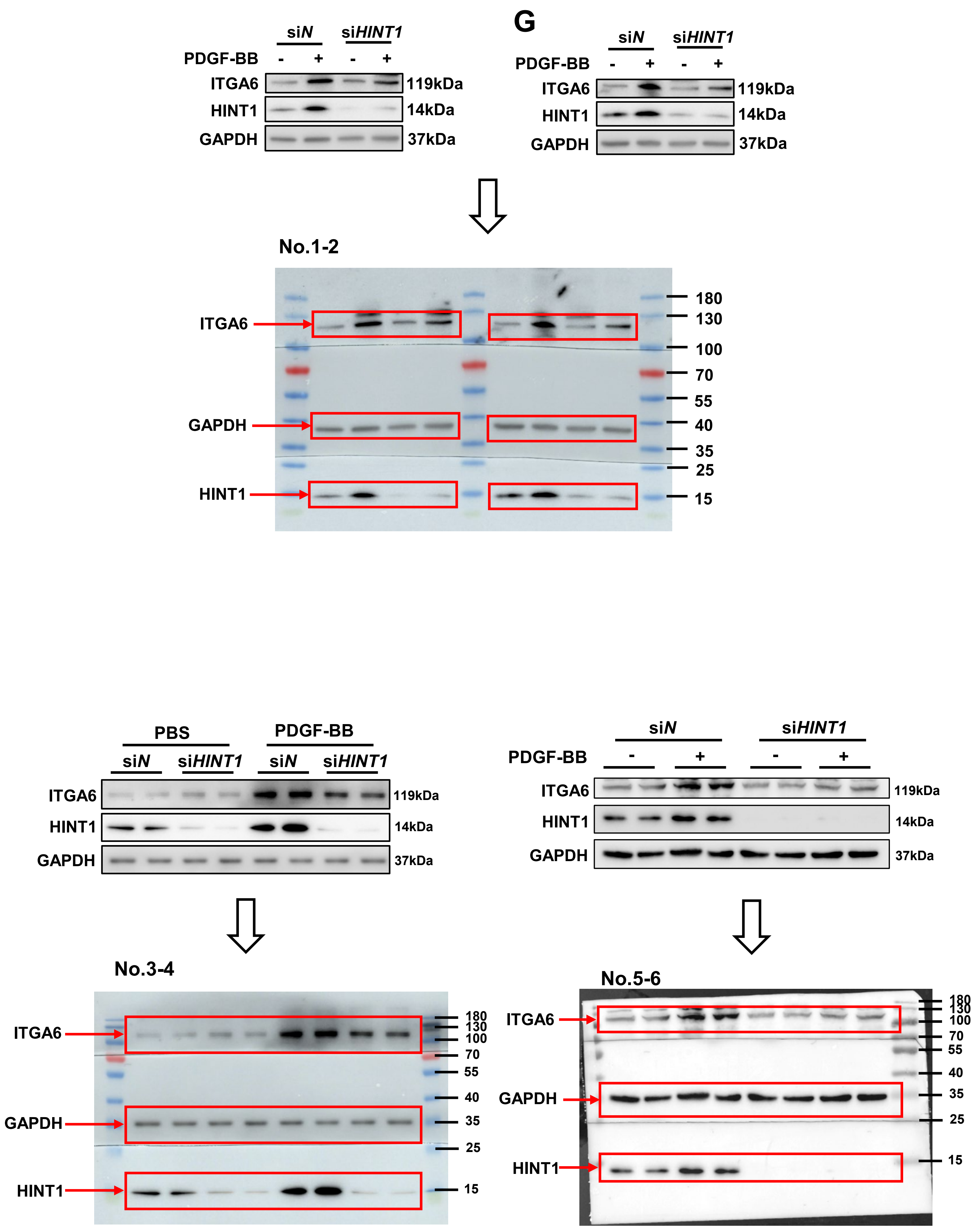

Full unedited gel for Figure 3I n=6

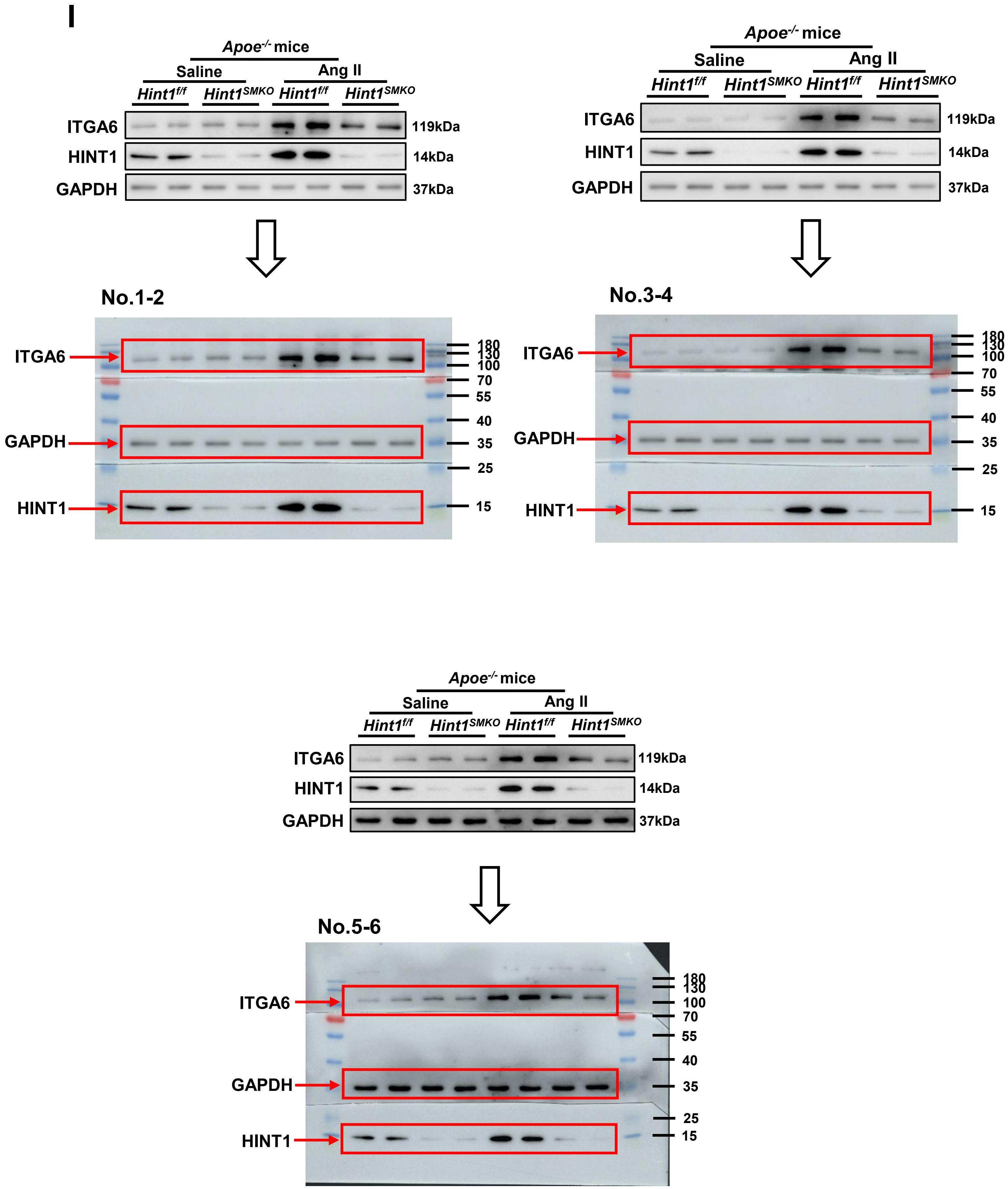

Full unedited gel for Figure 4A n=6

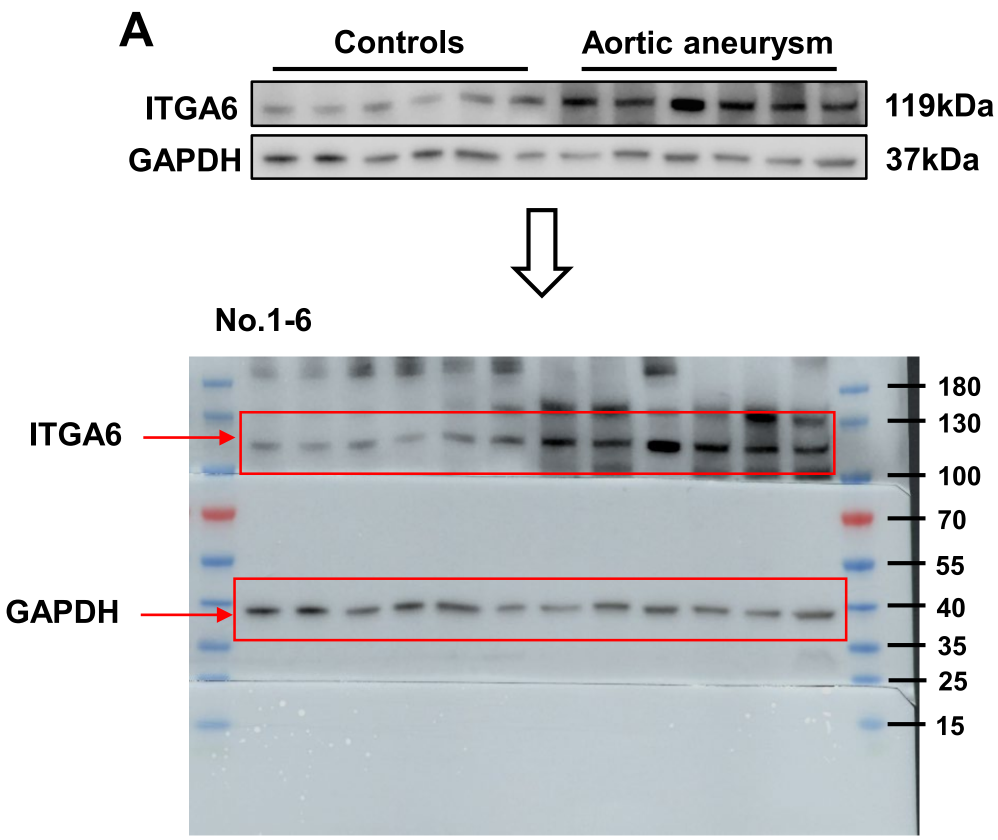

Full unedited gel for Figure 4I n=6

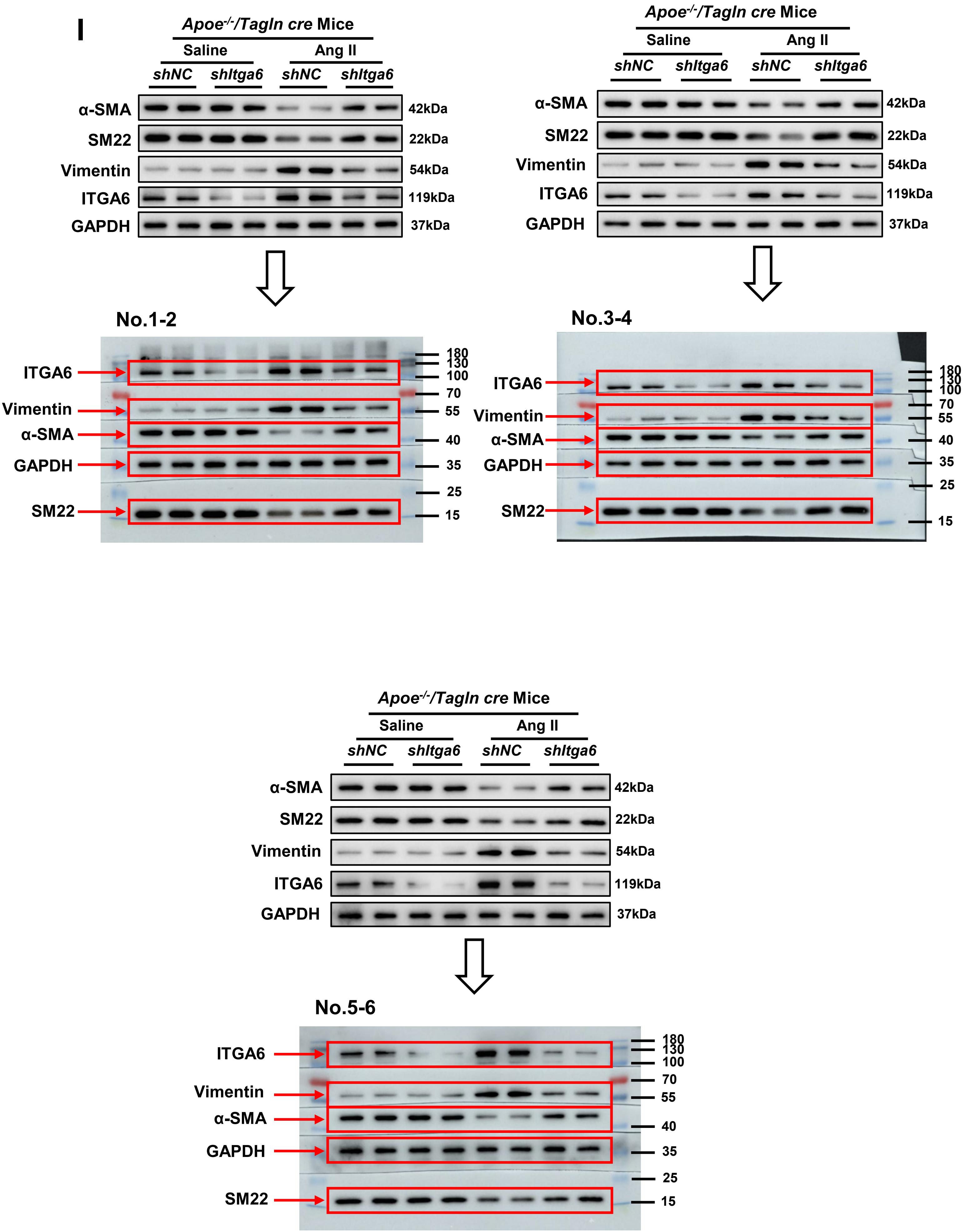

Full unedited gel for Figure 5G n=6

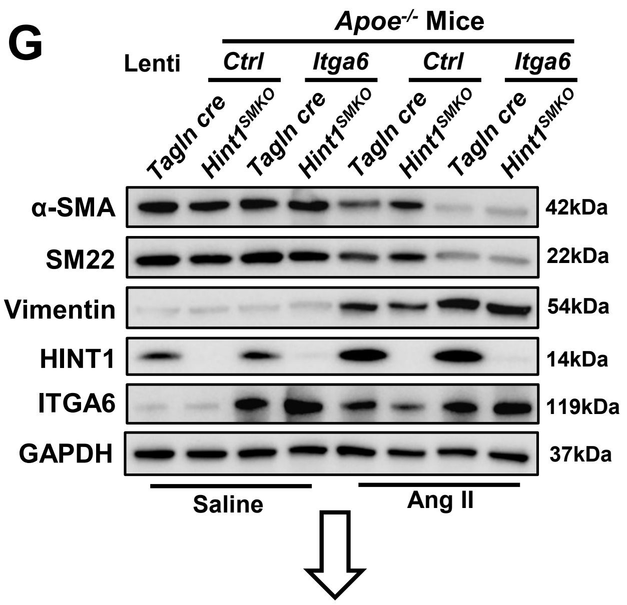

No.1

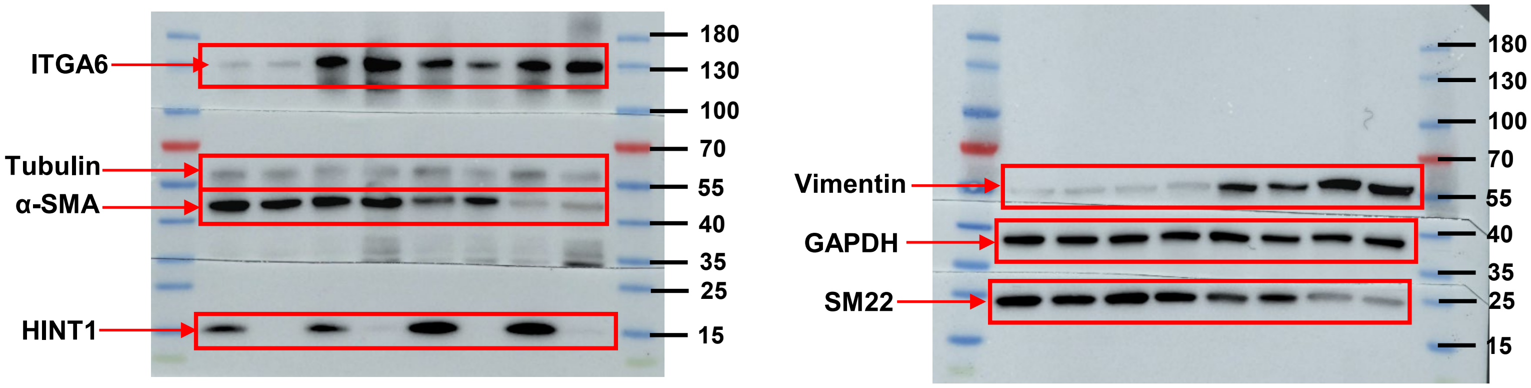

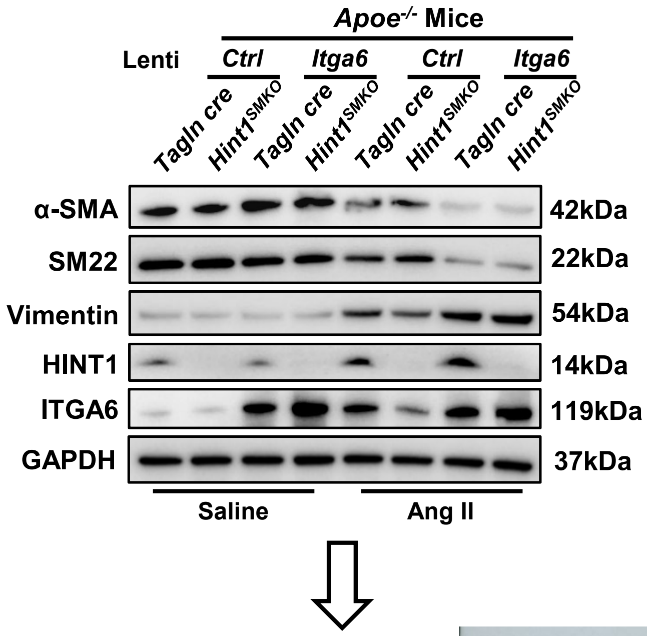

No.2

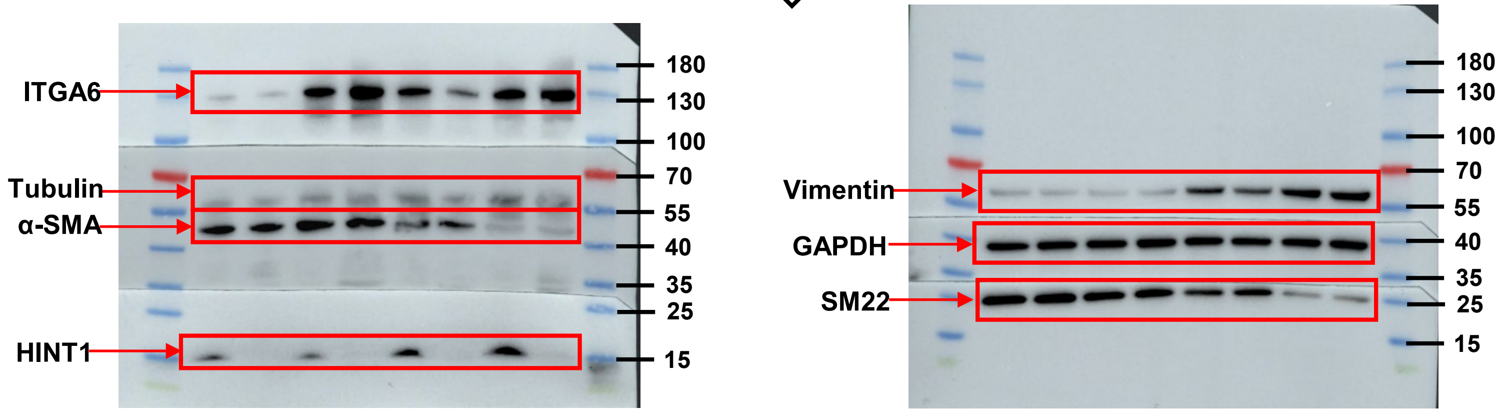

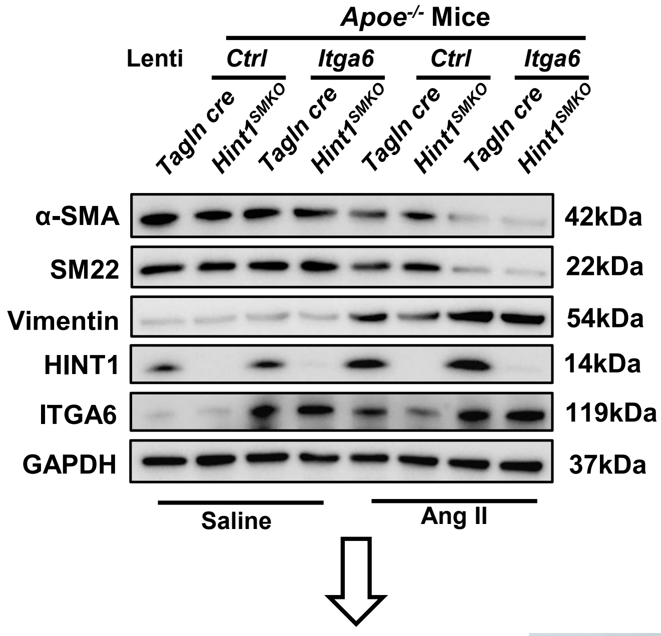
Apoe-/- Mice
Lenti
Ctrl
Itga6
Ctrl
Itga6
Tagln cre
Hint1SMKO
Tagln cre
Hint1SMKO
Tagln cre
Hint1SMKO
Tagln cre
Hint1SMKO
α-SMA
42kDa
SM22
22kDa
Vimentin
54kDa
HINT1
14kDa
ITGA6
119kDa
GAPDH
37kDa
Saline
Ang II

No.3

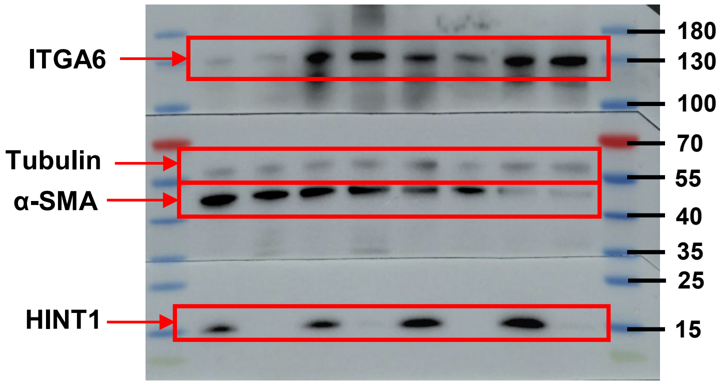
ITGA6
Tubulin
α-SMA
HINT1
180
130
100
70
55
40
35
25
15

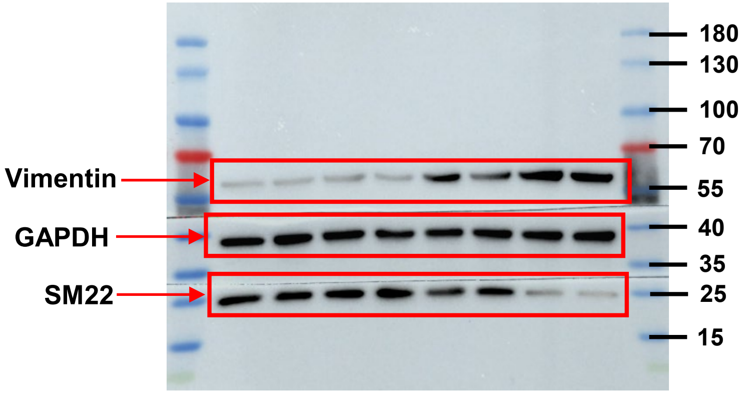
Vimentin
GAPDH
SM22
180
130
100
70
55
40
35
25
15

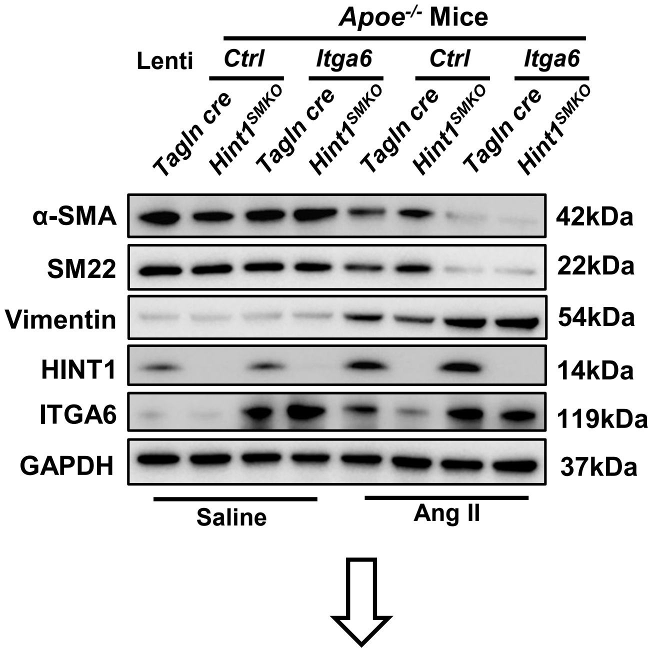
Apoe-/- Mice
Lenti
Ctrl
Itga6
Ctrl
Itga6
Tagln cre
Hint1SMKO
Tagln cre
Hint1SMKO
Tagln cre
Hint1SMKO
Tagln cre
Hint1SMKO
α-SMA
42kDa
SM22
22kDa
Vimentin
54kDa
HINT1
14kDa
ITGA6
119kDa
GAPDH
37kDa
Saline
Ang II

No.4

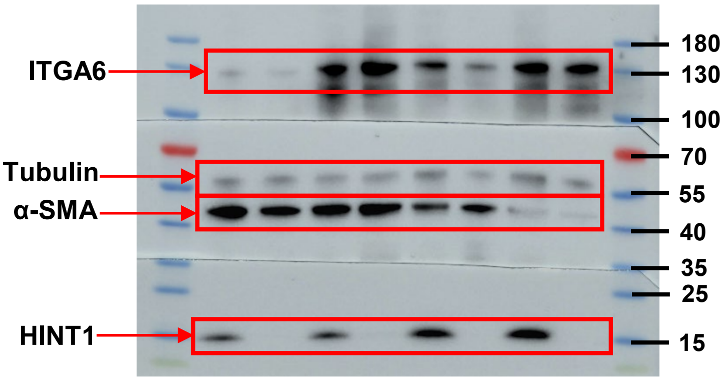
ITGA6
Tubulin
α-SMA
HINT1
180
130
100
70
55
40
35
25
15

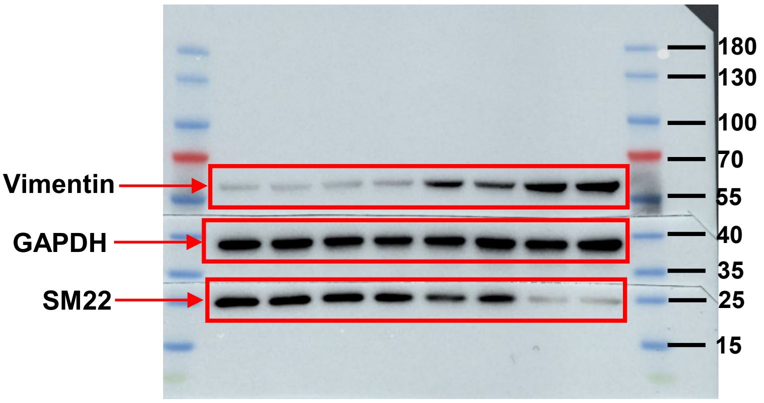
Vimentin
GAPDH
SM22
180
130
100
70
55
40
35
25
15

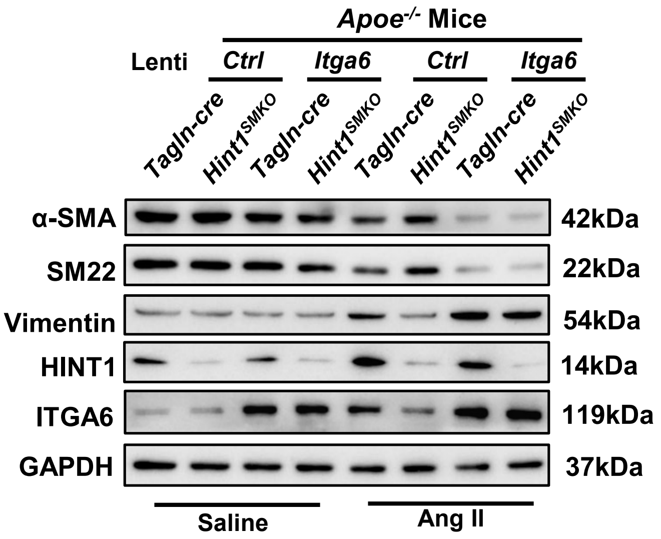
Apoe-/- Mice
Lenti
Ctrl
Itga6
Ctrl
Itga6
Tagln-cre
Hint1SMKO
Tagln-cre
Hint1SMKO
Tagln-cre
Hint1SMKO
Tagln-cre
Hint1SMKO
α-SMA
42kDa
SM22
22kDa
Vimentin
54kDa
HINT1
14kDa
ITGA6
119kDa
GAPDH
37kDa
Saline
Ang II

No.5

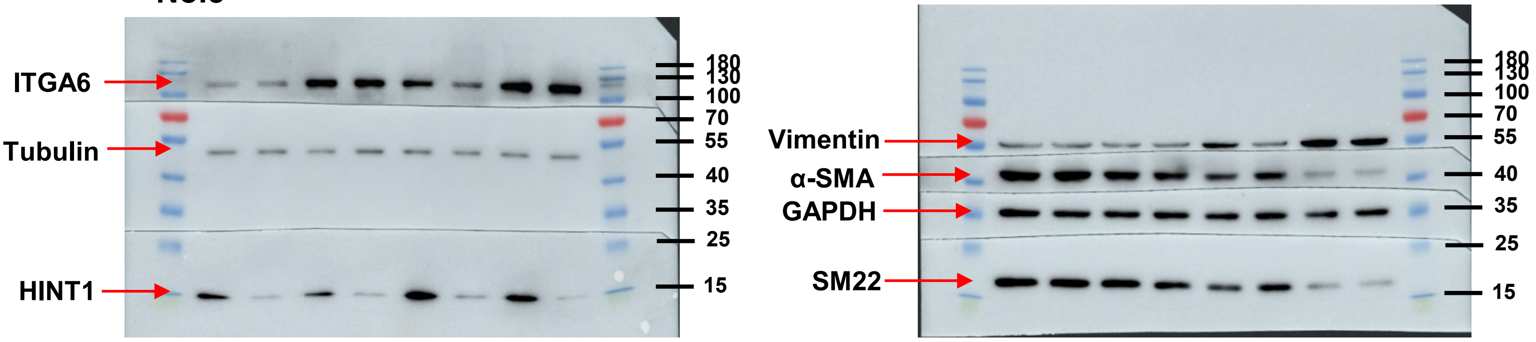
ITGA6
Tubulin
HINT1
180
130
100
70
55
40
35
25
15
Vimentin
α-SMA
GAPDH
SM22
180
130
100
70
55
40
35
25
15

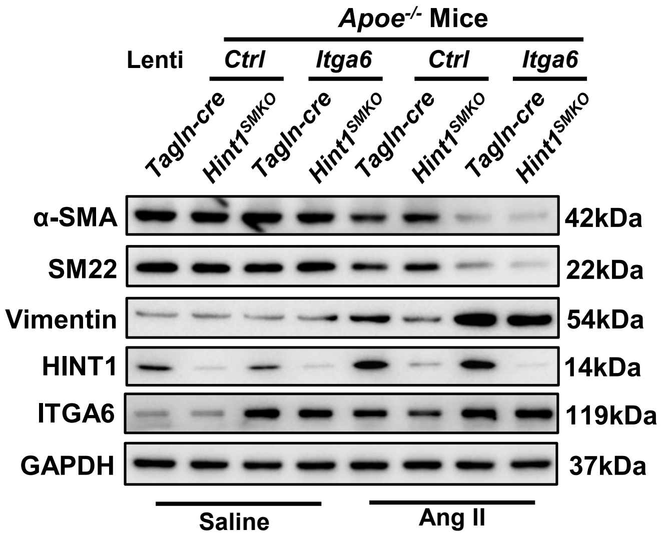
Apoe-/- Mice
Lenti
Ctrl
Itga6
Ctrl
Itga6
Tagln-cre
Hint1SMKO
Tagln-cre
Hint1SMKO
Tagln-cre
Hint1SMKO
Tagln-cre
Hint1SMKO
α-SMA
42kDa
SM22
22kDa
Vimentin
54kDa
HINT1
14kDa
ITGA6
119kDa
GAPDH
37kDa
Saline
Ang II

No.6

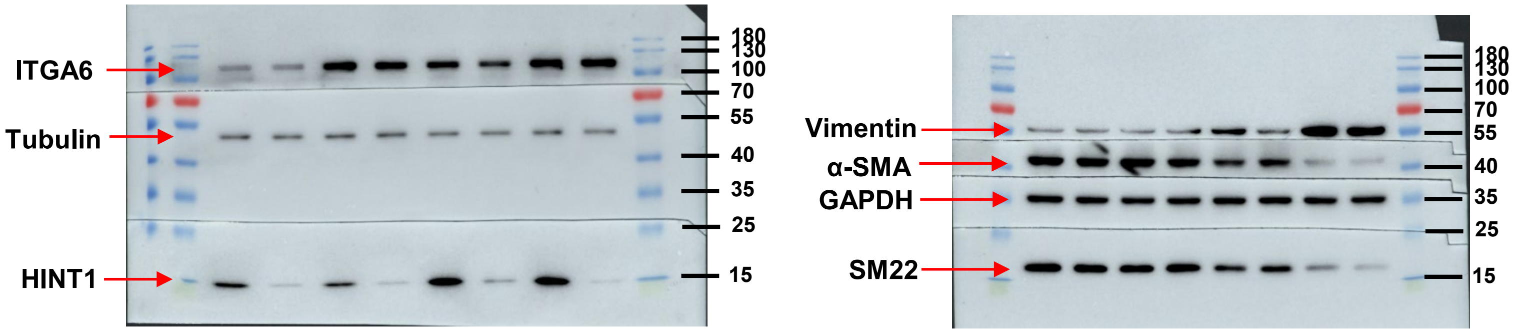
ITGA6
Tubulin
HINT1
180
130
100
70
55
40
35
25
15
Vimentin
α-SMA
GAPDH
SM22
180
130
100
70
55
40
35
25
15

Full unedited gel for Figure 6B n=6

B

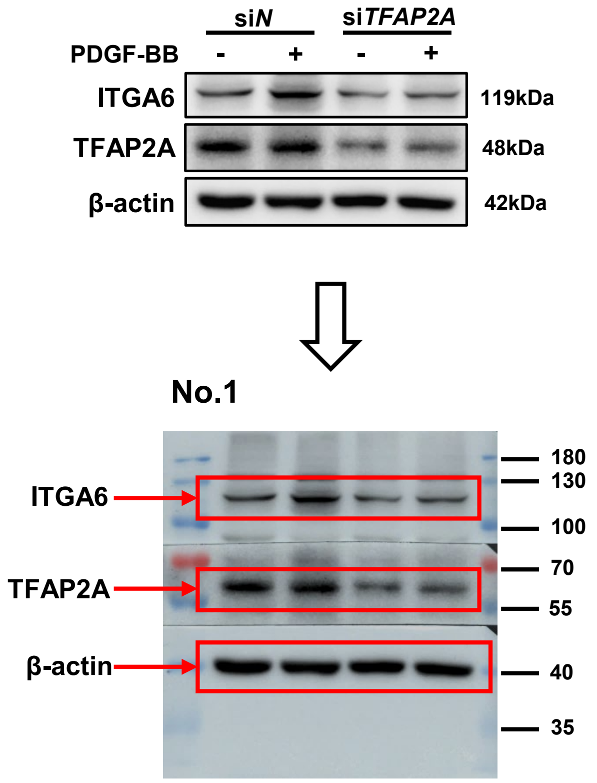

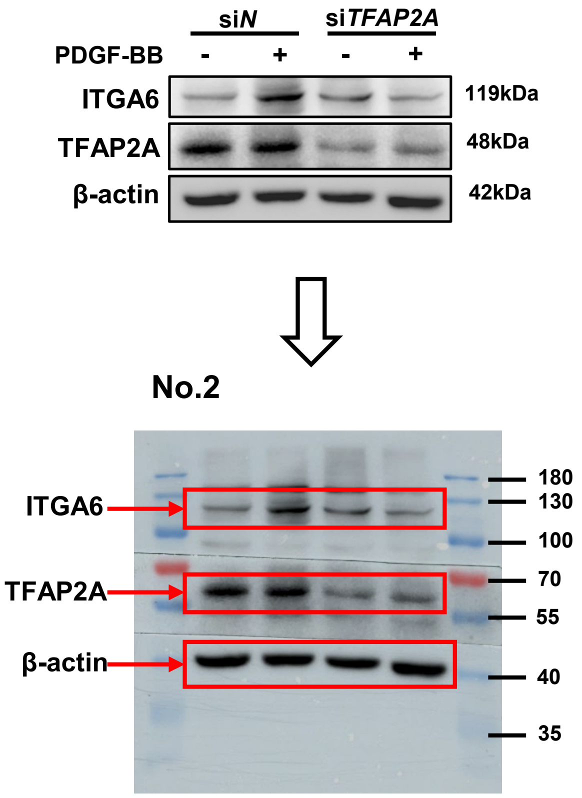

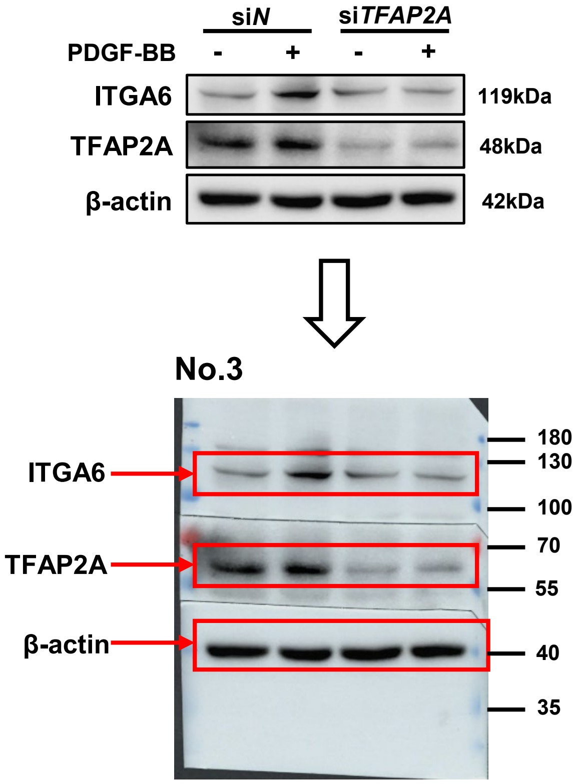

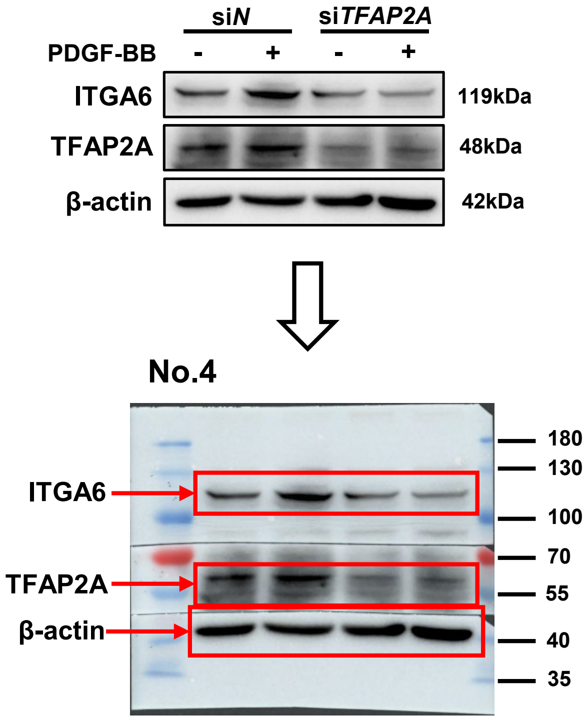

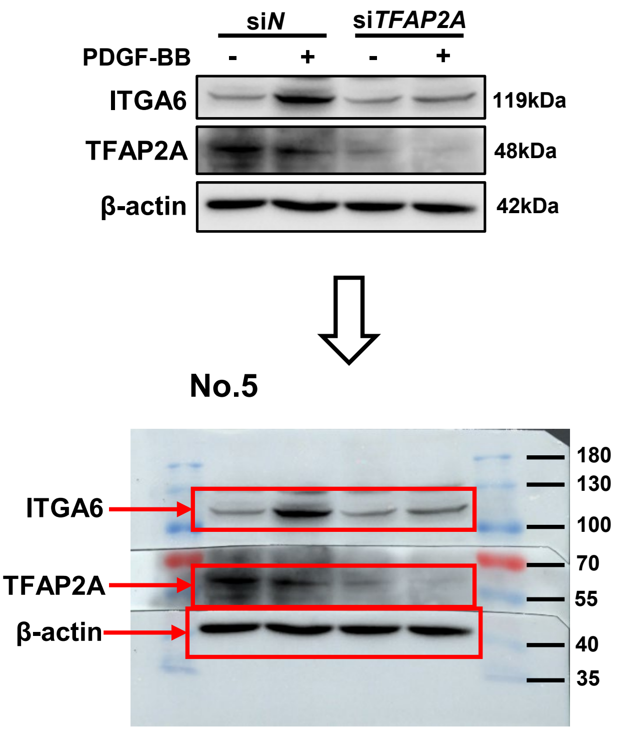

siN
siTFAP2A
PDGF-BB
- + - +
ITGA6
119kDa
TFAP2A
48kDa
β-actin
42kDa
No.5
ITGA6
TFAP2A
β-actin
180
130
100
70
55
40
35

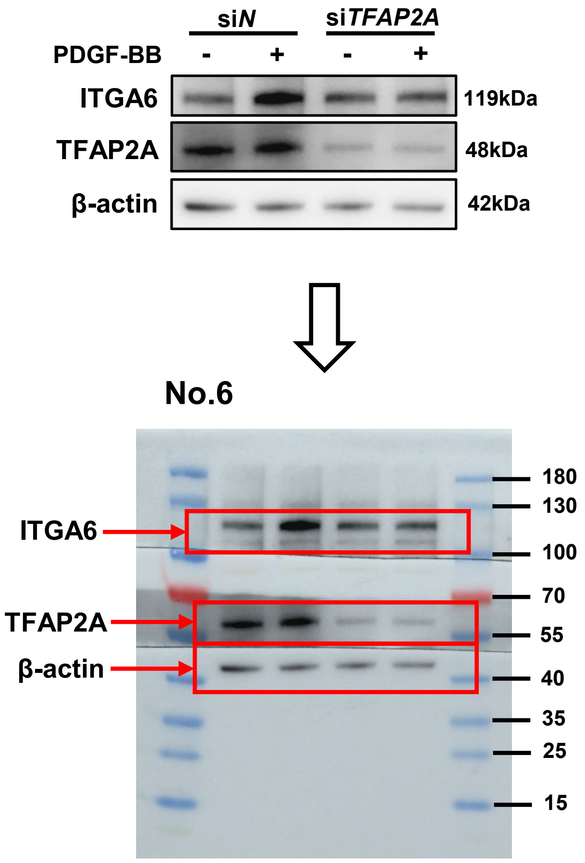

siN
siTFAP2A
PDGF-BB
- + - +
ITGA6
119kDa
TFAP2A
48kDa
β-actin
42kDa
No.6
ITGA6
TFAP2A
β-actin
180
130
100
70
55
40
35
25
15

Full unedited gel for Figure 6H n=1

H

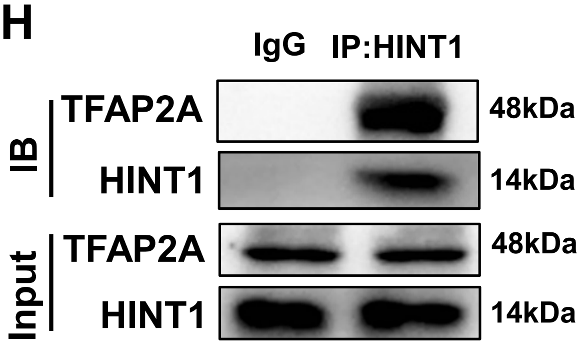

No.1

IB

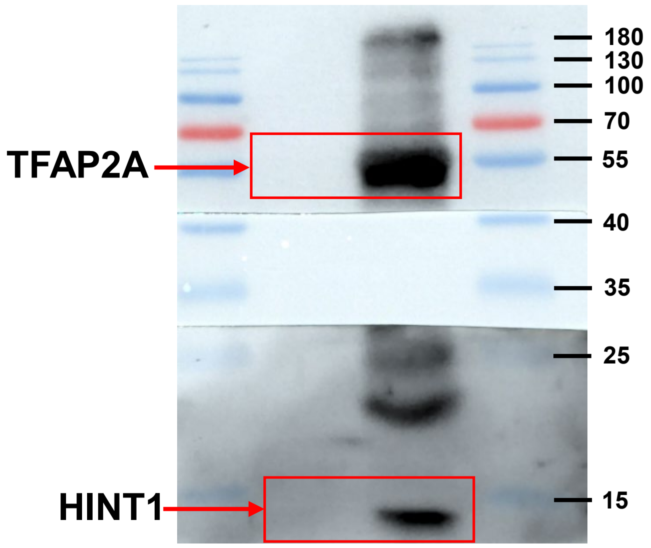

Input

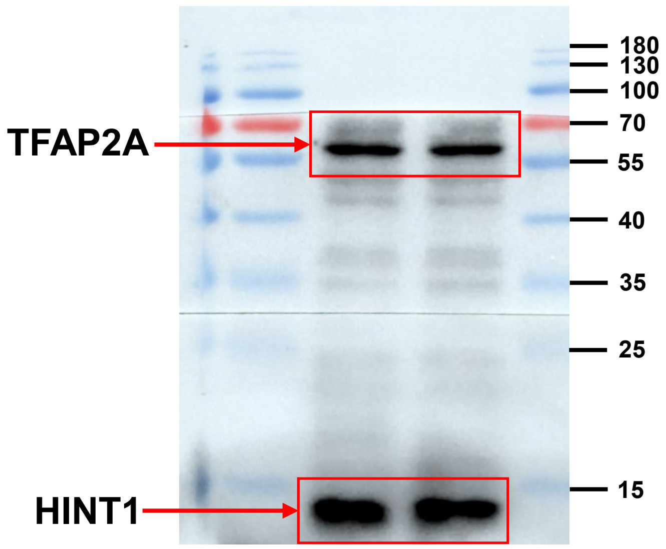

Full unedited gel for Figure 6J n=1

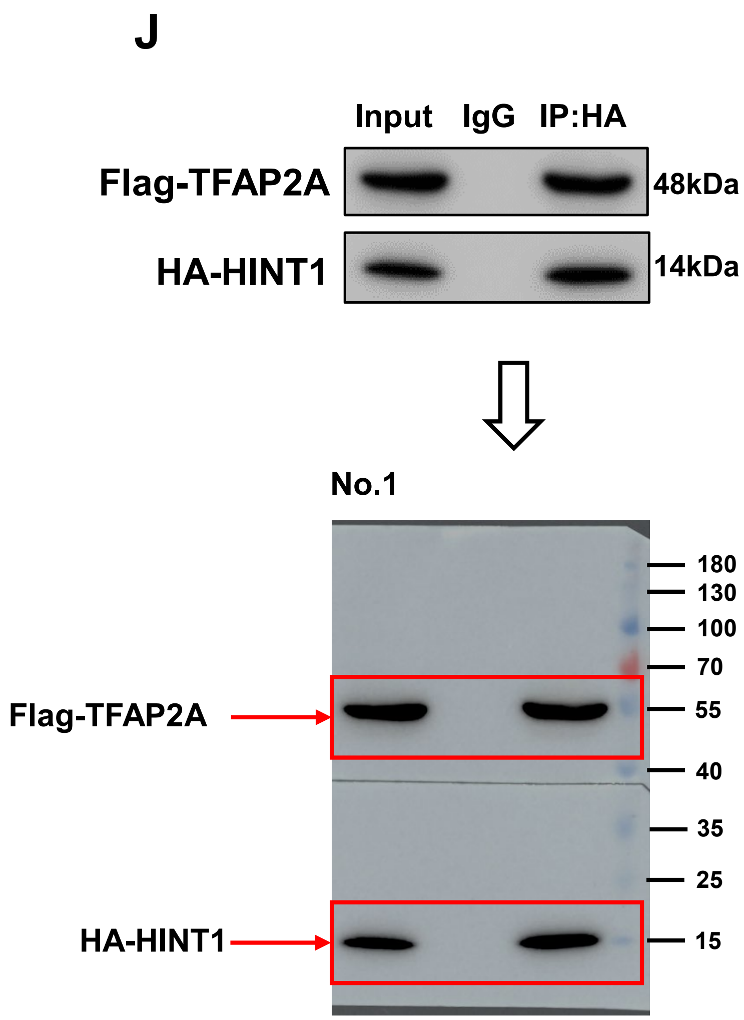

Full unedited gel for Figure 6K n=1

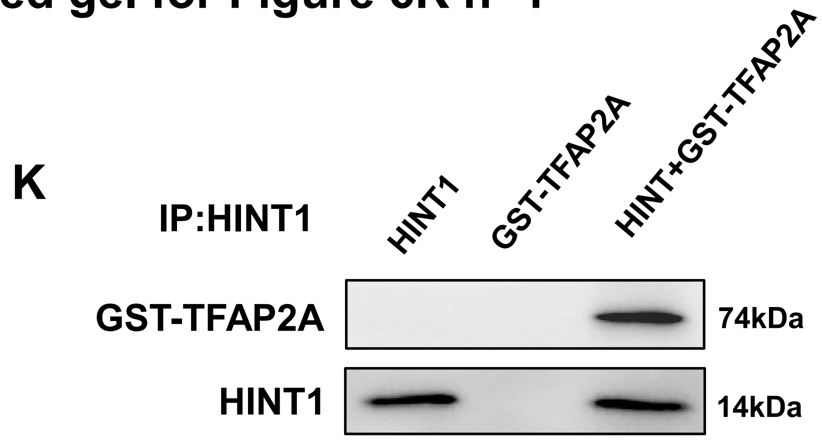

No.1

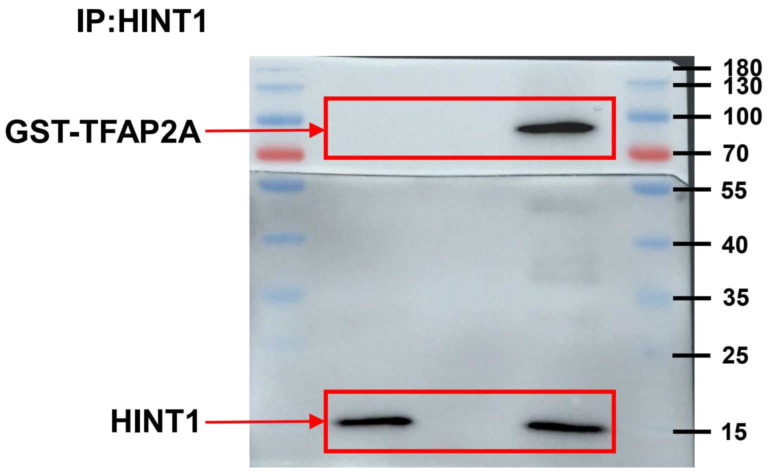

Full unedited gel for Figure 6L n=1

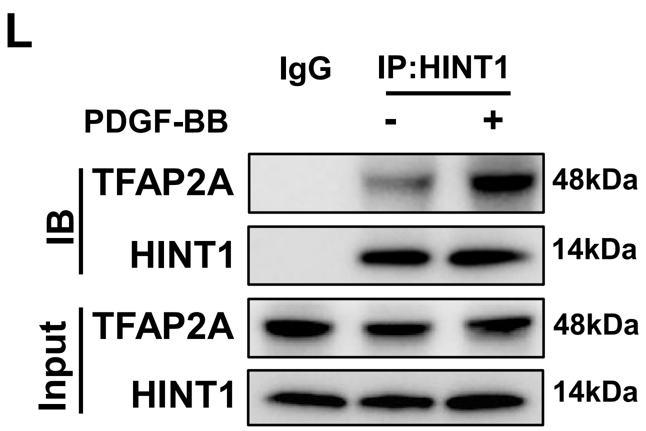

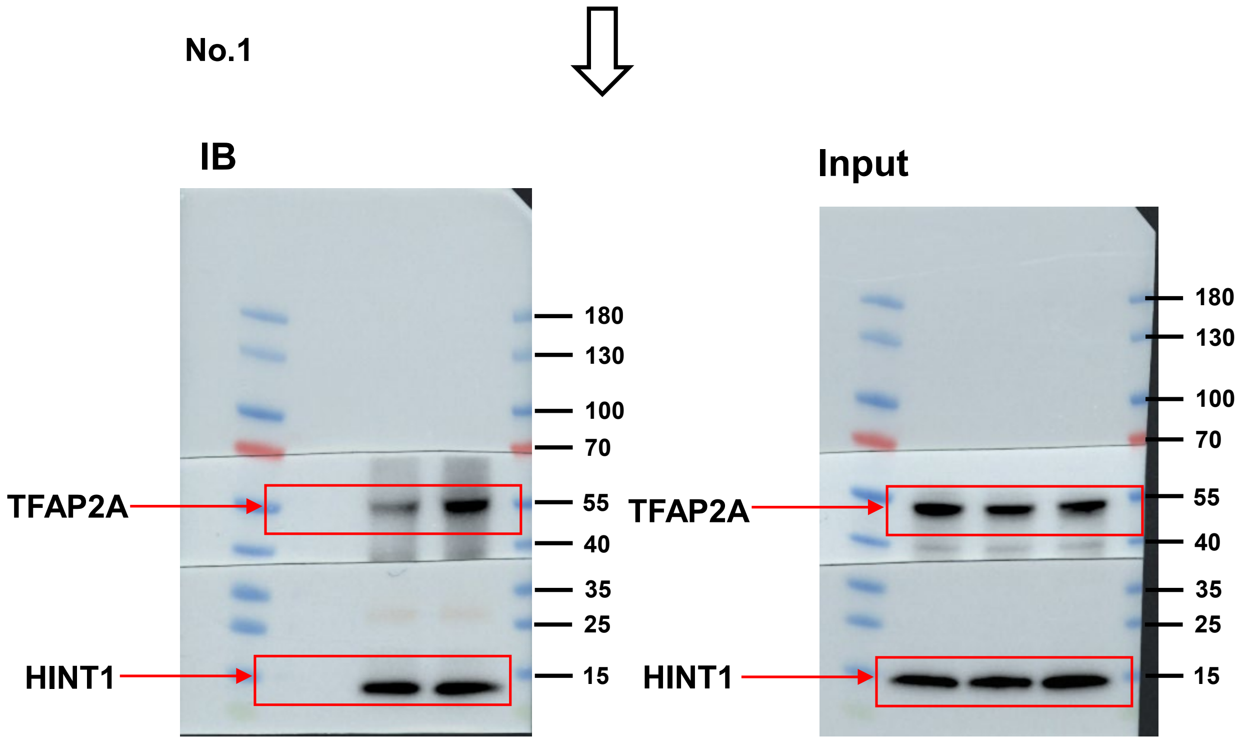

Full unedited gel for Figure 7A n=6

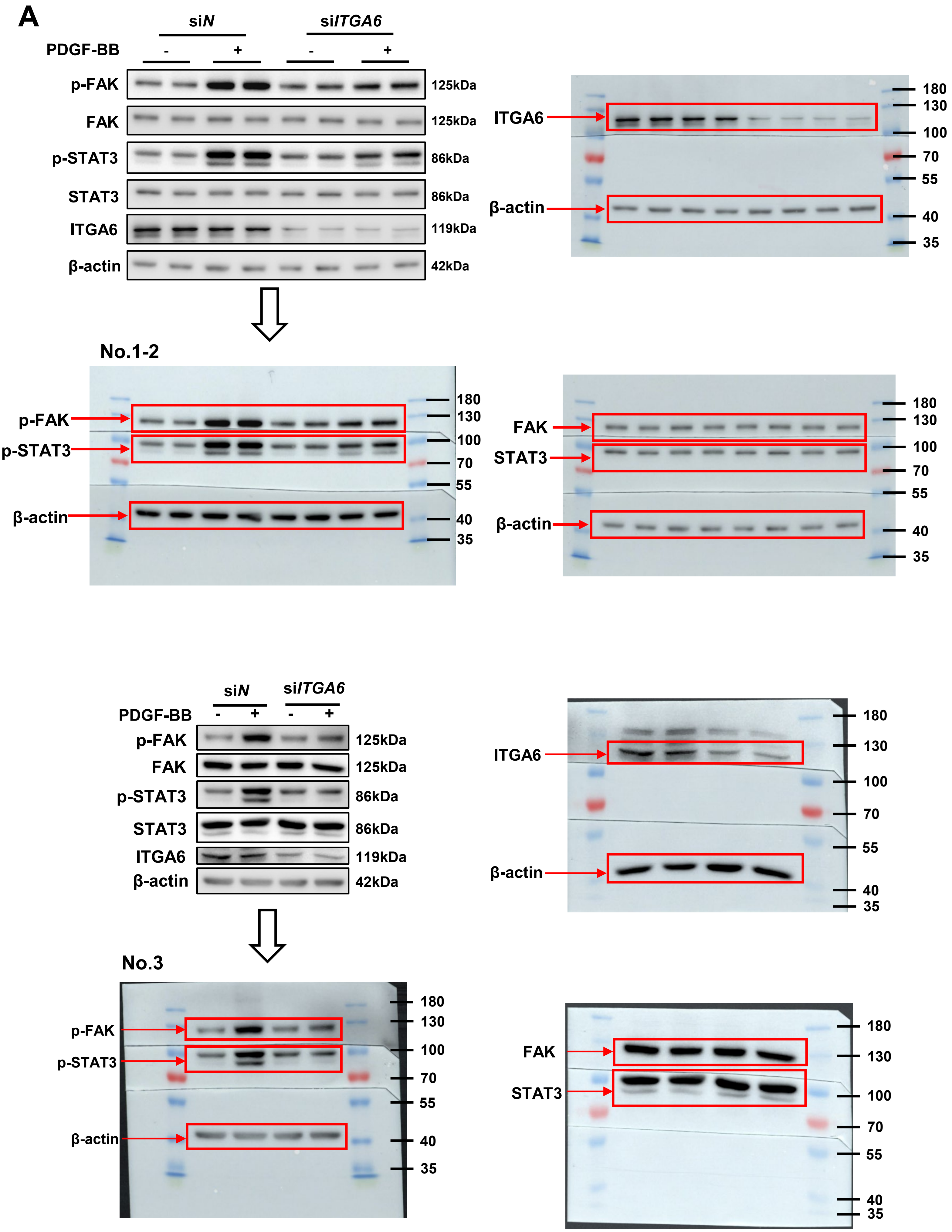

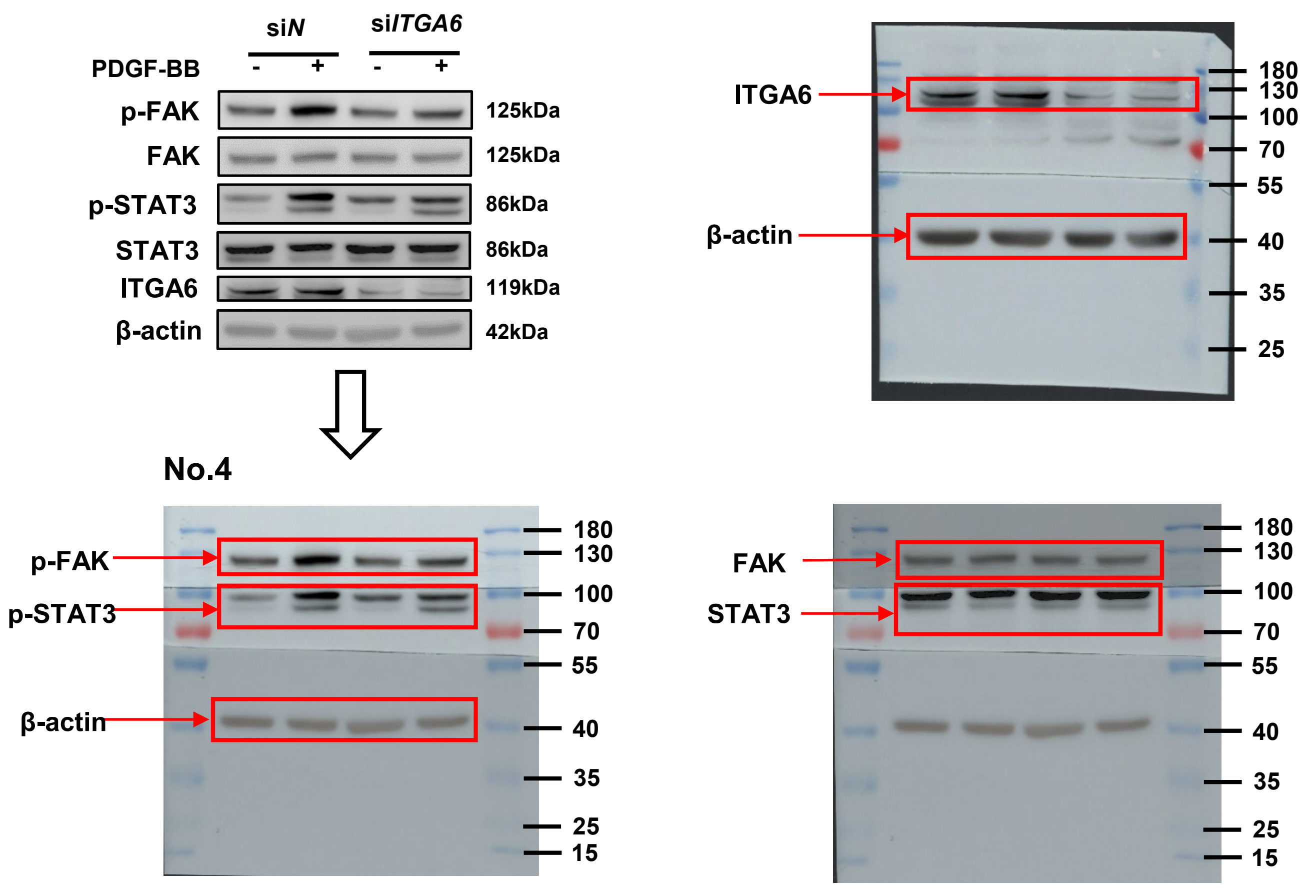
siN
siITGA6
PDGF-BB
- + - +
p-FAK
FAK
p-STAT3
STAT3
ITGA6
β-actin
125kDa
125kDa
86kDa
86kDa
119kDa
42kDa
ITGA6
β-actin
180
130
100
70
55
40
35
25
No.4
p-FAK
p-STAT3
β-actin
FAK
STAT3
15

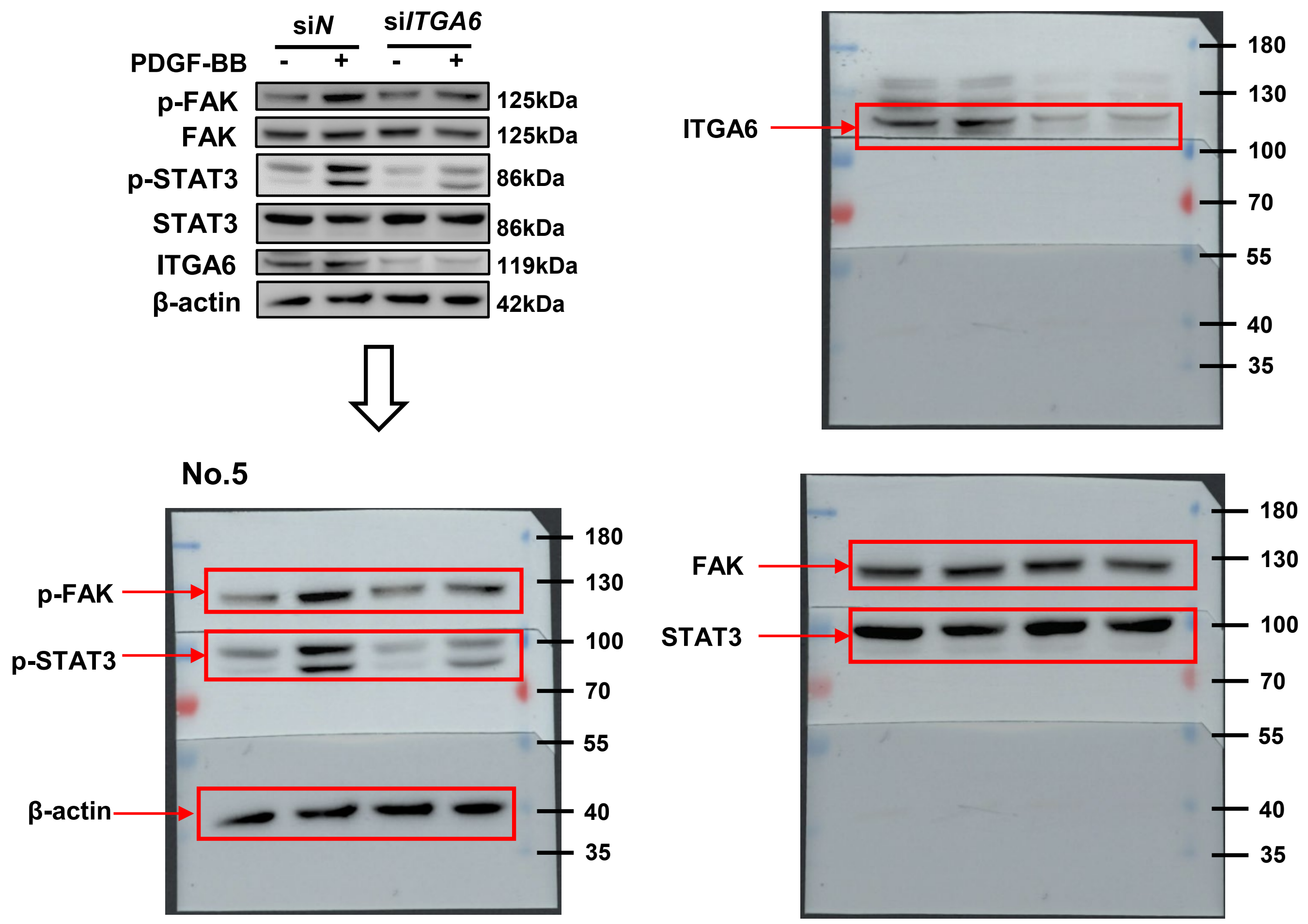
siN
siITGA6
PDGF-BB
- + - +
p-FAK
FAK
p-STAT3
STAT3
ITGA6
β-actin
125kDa
125kDa
86kDa
86kDa
119kDa
42kDa
ITGA6
180
130
100
70
55
40
35
No.5
p-FAK
p-STAT3
β-actin
FAK
STAT3

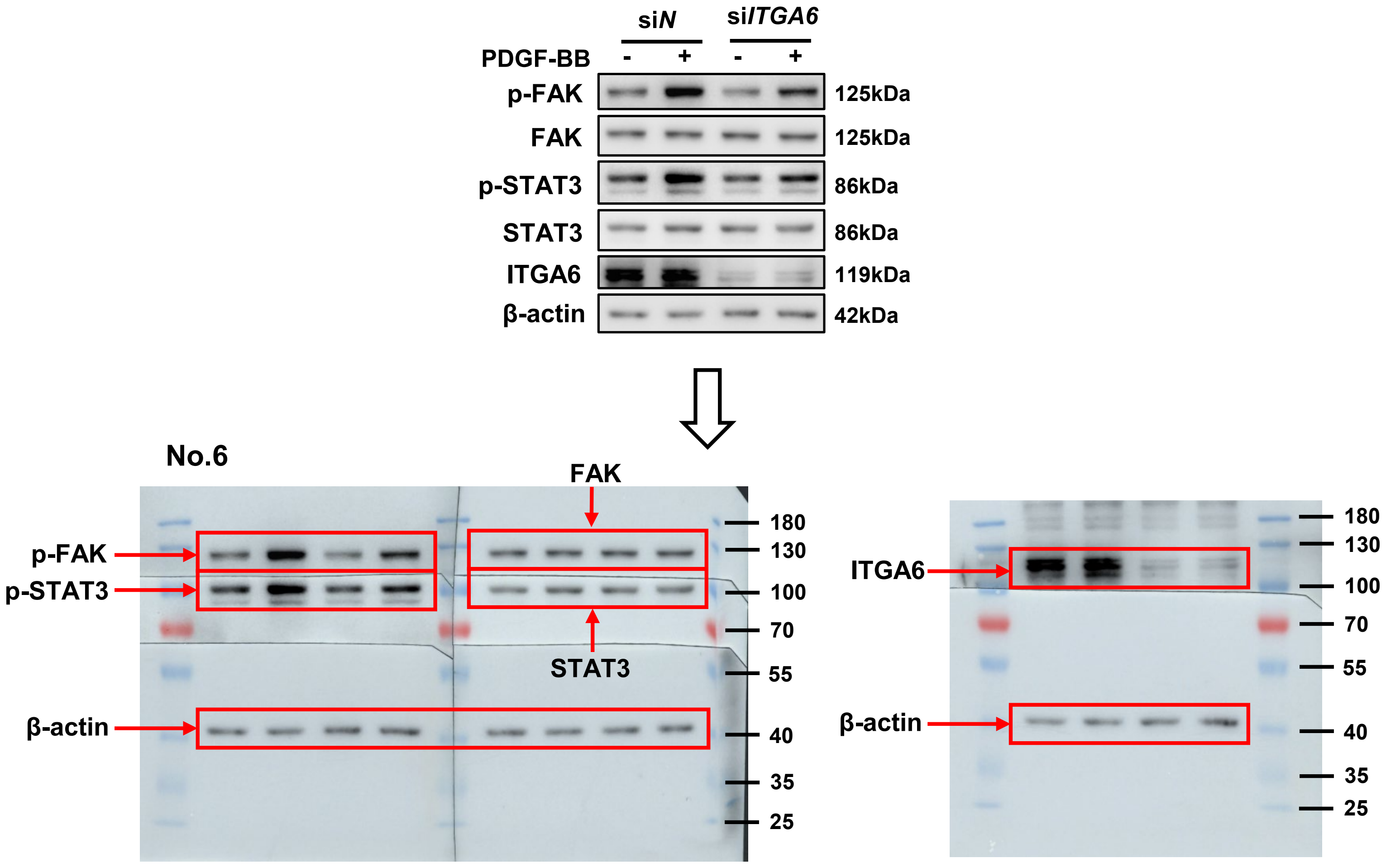

siN
siITGA6
PDGF-BB
-
+
-
+
p-FAK
FAK
p-STAT3
STAT3
ITGA6
β-actin
125kDa
125kDa
86kDa
86kDa
119kDa
42kDa
No.6
p-FAK
p-STAT3
β-actin
FAK
STAT3
180
130
100
70
55
40
35
25
ITGA6
β-actin
180
130
100
70
55
40
35
25

Full unedited gel for Figure 7B n=6

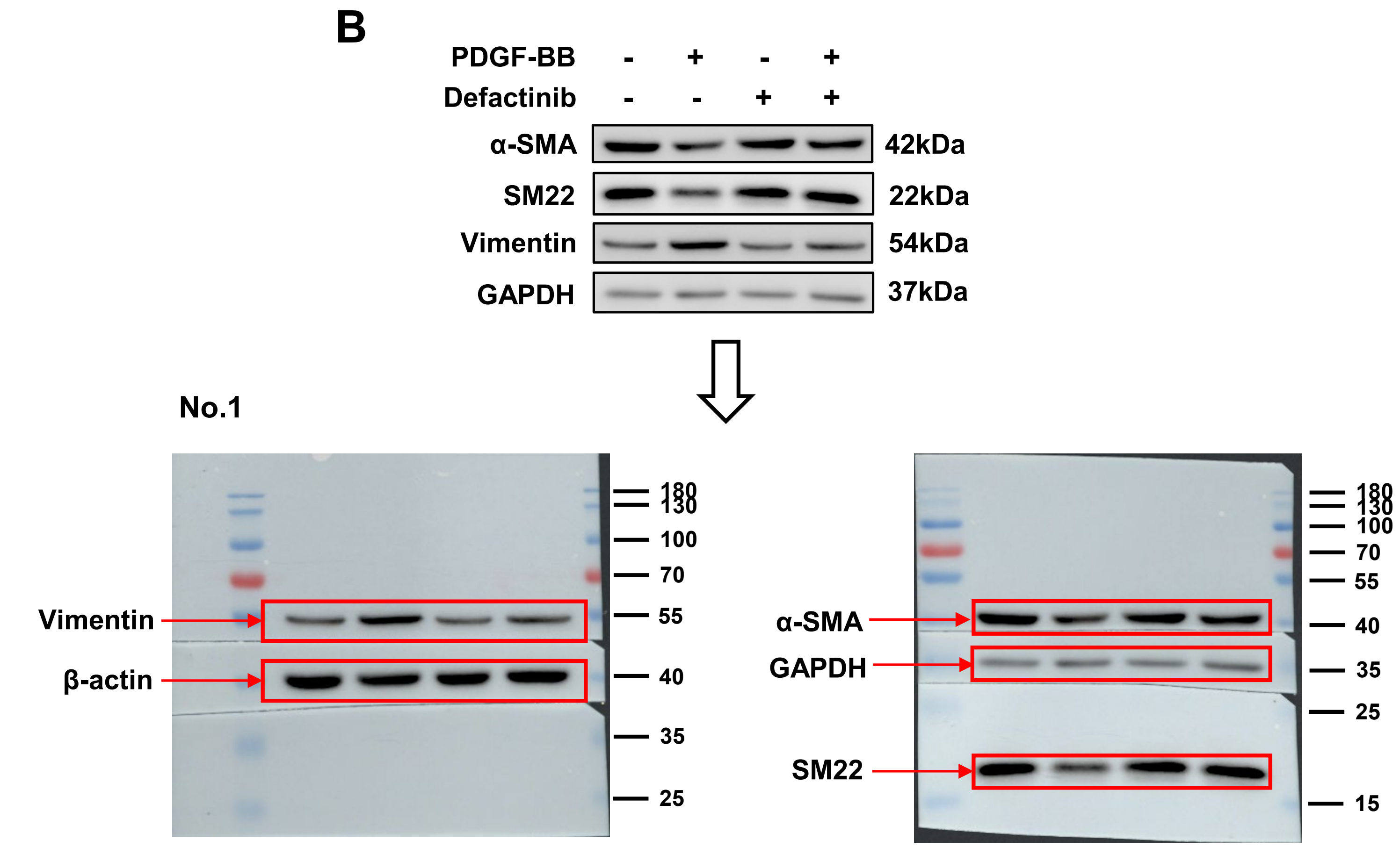

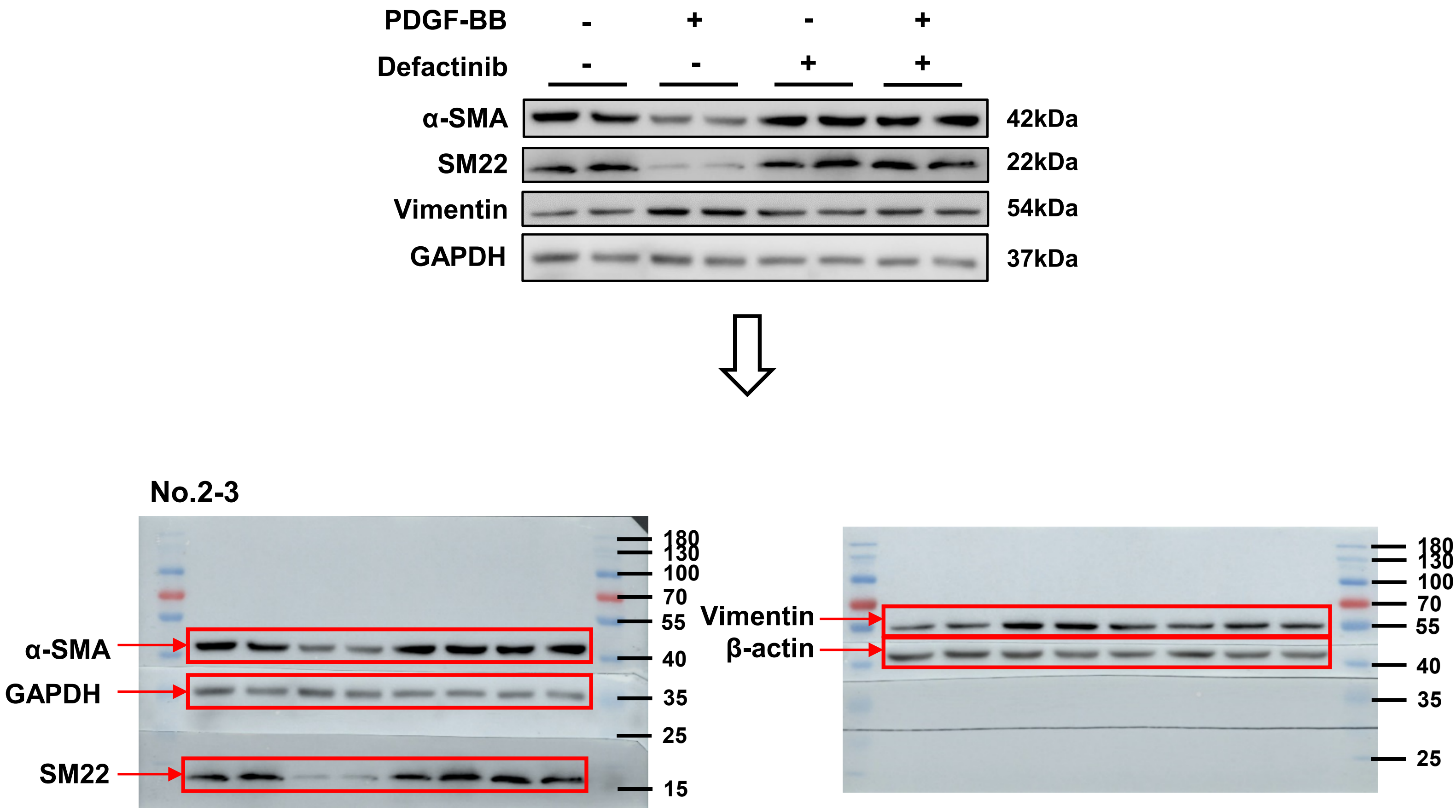

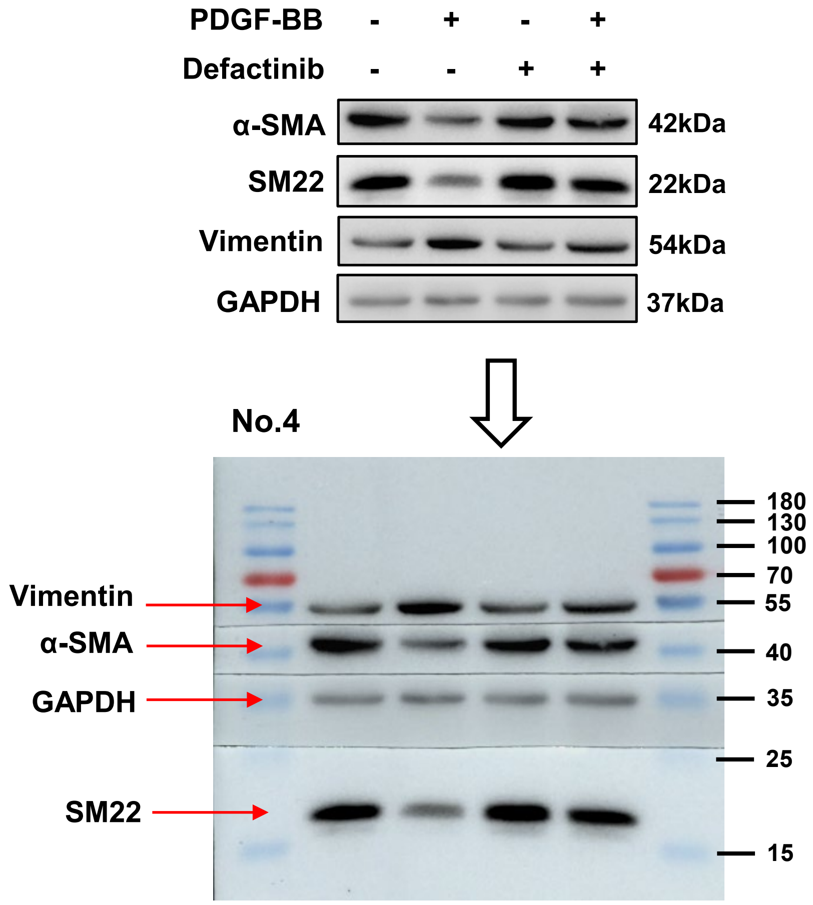
PDGF-BB - + - +
Defactinib - - + +
α-SMA
42kDa
SM22
22kDa
Vimentin
54kDa
GAPDH
37kDa
No.4
Vimentin
α-SMA
GAPDH
SM22
180
130
100
70
55
40
35
25
15

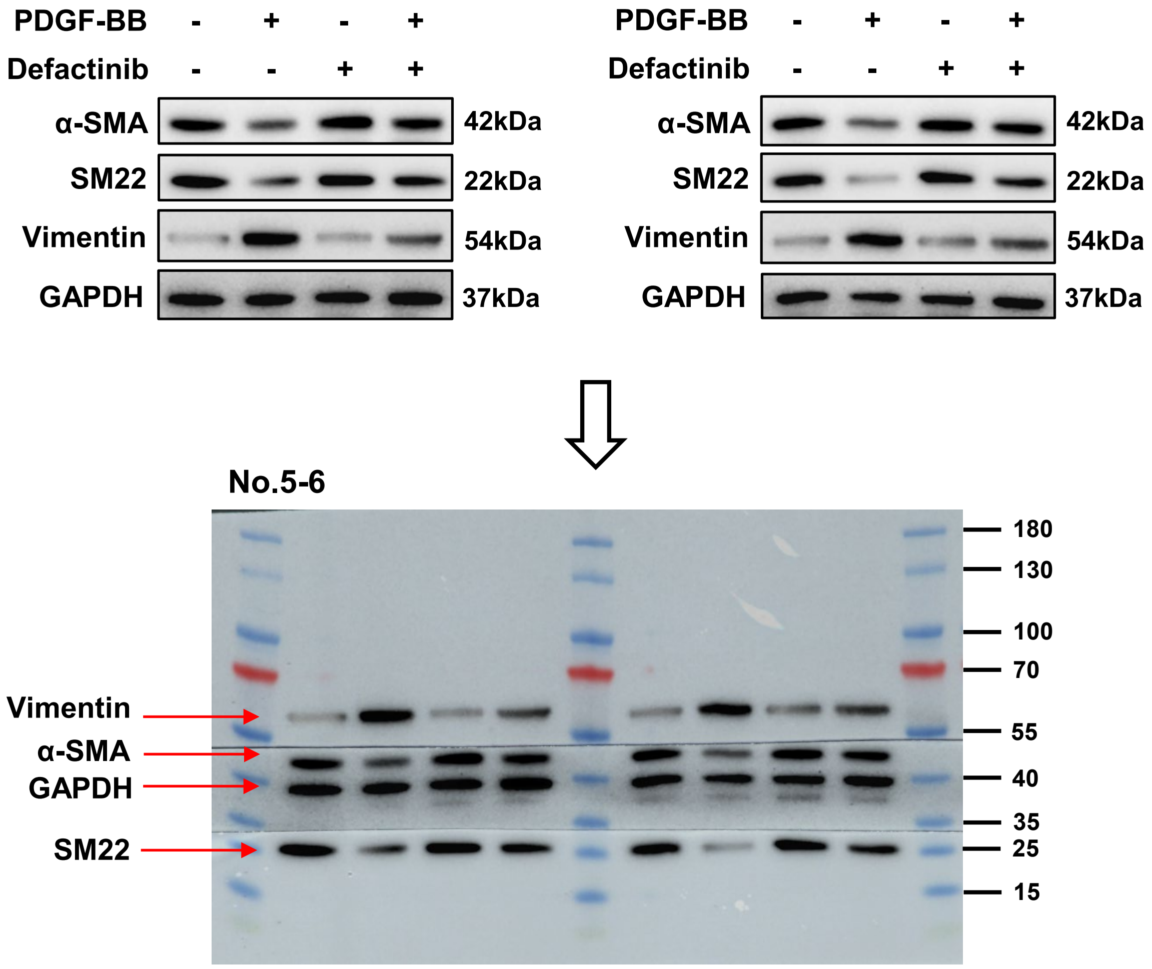
PDGF-BB - + - +
Defactinib - - + +
α-SMA
42kDa
SM22
22kDa
Vimentin
54kDa
GAPDH
37kDa
PDGF-BB - + - +
Defactinib - - + +
α-SMA
42kDa
SM22
22kDa
Vimentin
54kDa
GAPDH
37kDa
No.5-6
Vimentin
α-SMA
GAPDH
SM22
180
130
100
70
55
40
35
25
15

Full unedited gel for Figure 7C n=6

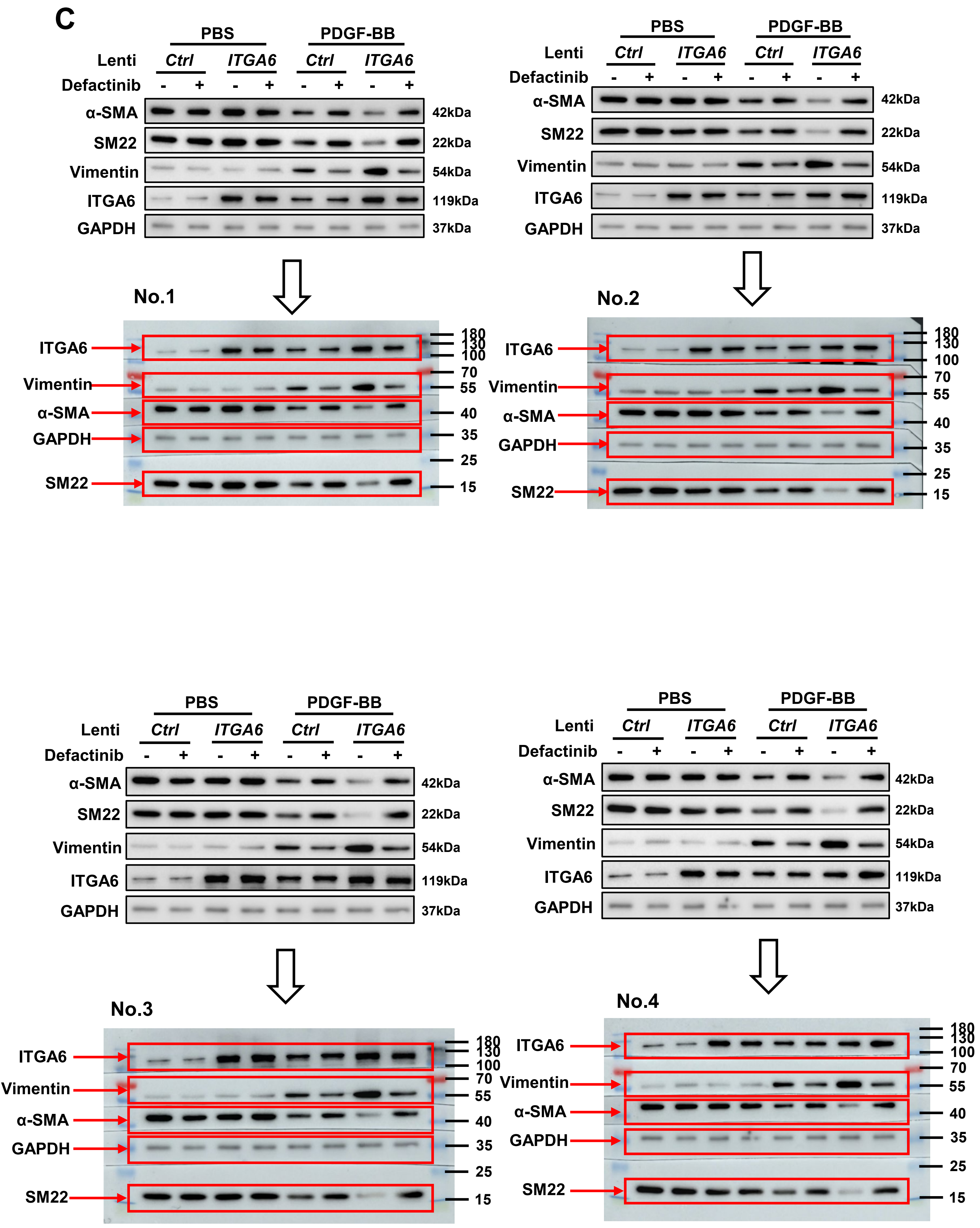

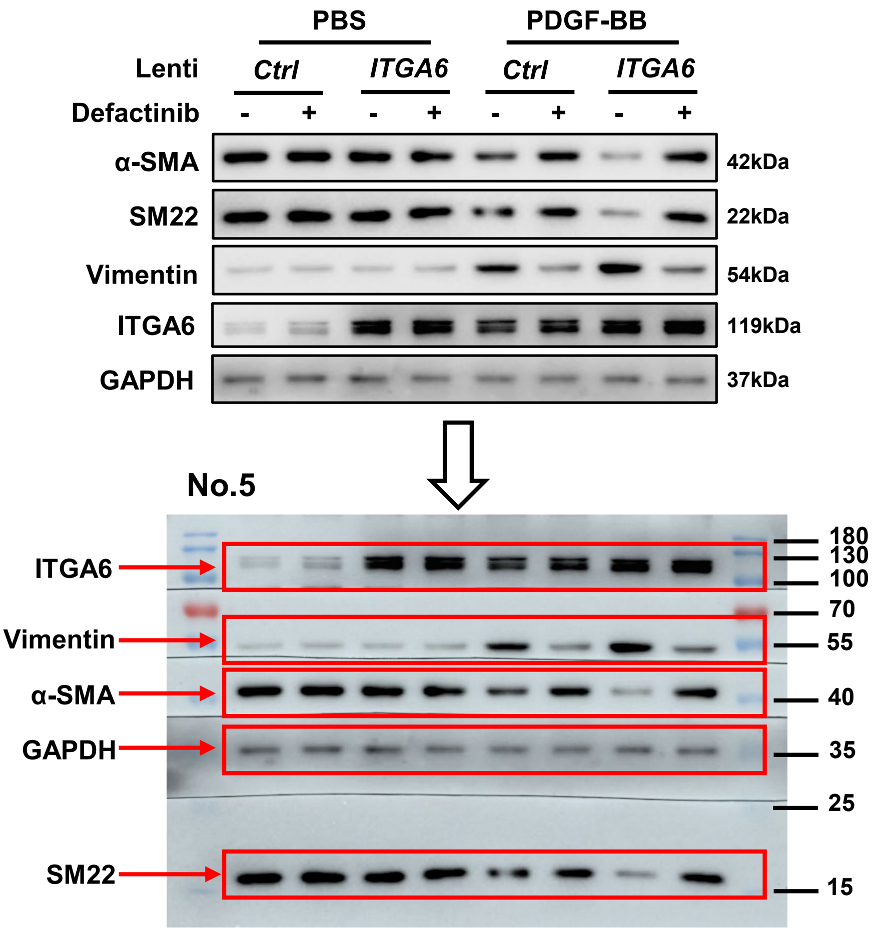
PBS
PDGF-BB
Lenti
Ctrl
ITGA6
Ctrl
ITGA6
Defactinib
- + - + - + - +
α-SMA
42kDa
SM22
22kDa
Vimentin
54kDa
ITGA6
119kDa
GAPDH
37kDa
No.5
ITGA6
Vimentin
α-SMA
GAPDH
SM22
180
130
100
70
55
40
35
25
15

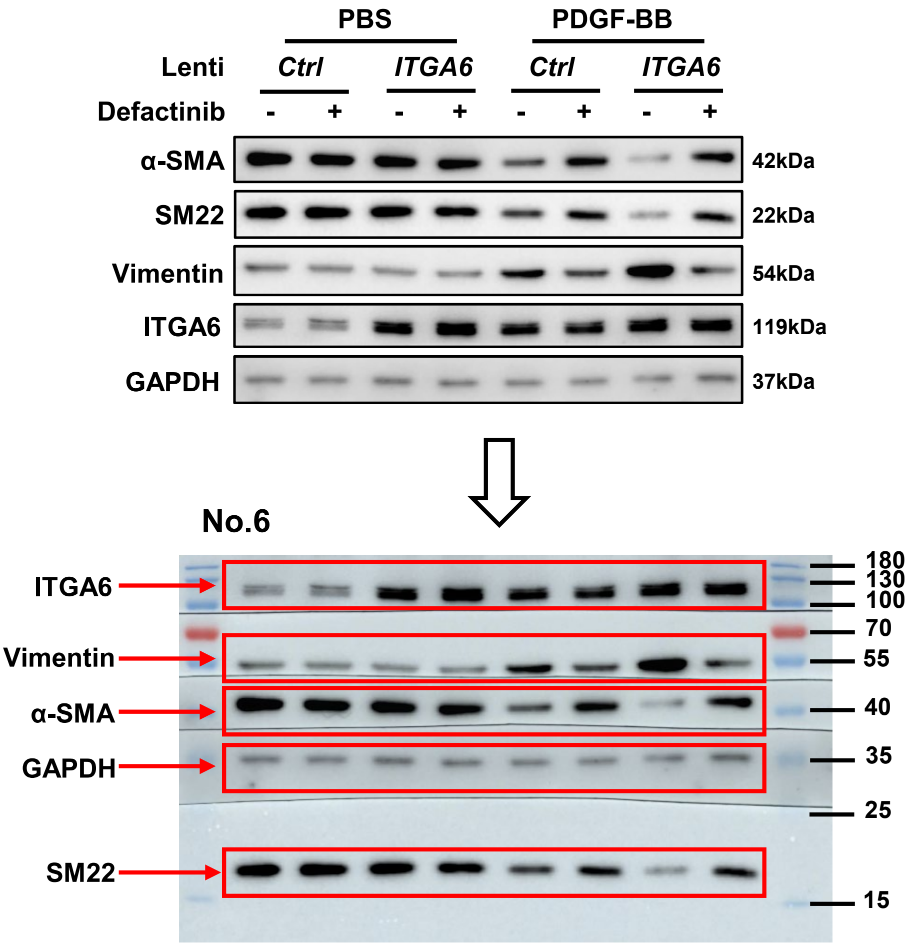
PBS
PDGF-BB
Lenti
Ctrl
ITGA6
Ctrl
ITGA6
Defactinib
- + - + - + - +
α-SMA
42kDa
SM22
22kDa
Vimentin
54kDa
ITGA6
119kDa
GAPDH
37kDa
No.6
ITGA6
Vimentin
α-SMA
GAPDH
SM22
180
130
100
70
55
40
35
25
15

Full unedited gel for Figure 7D n=6

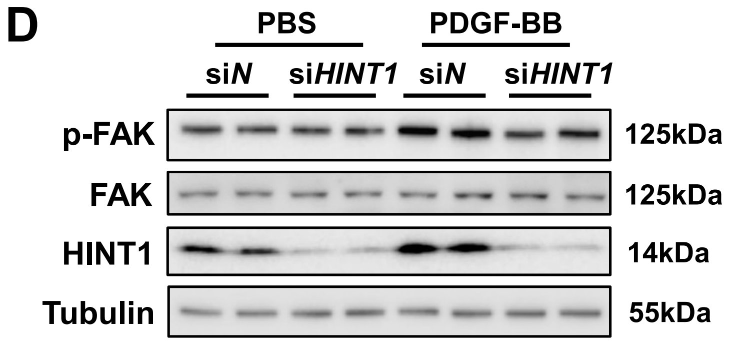

No.1-2

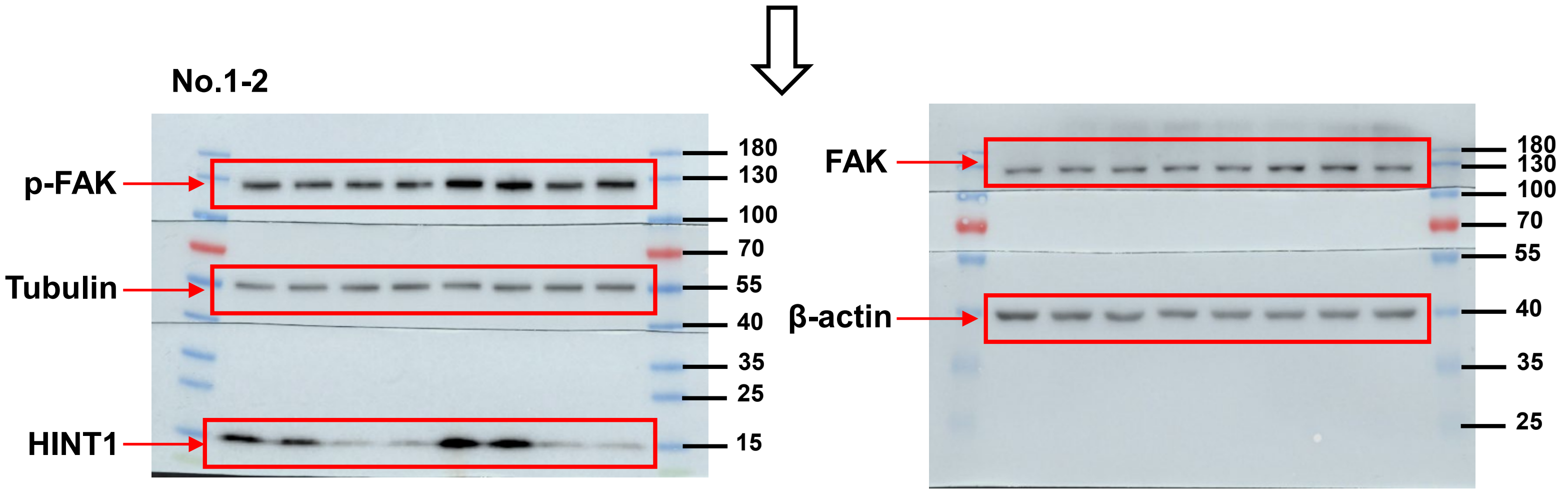

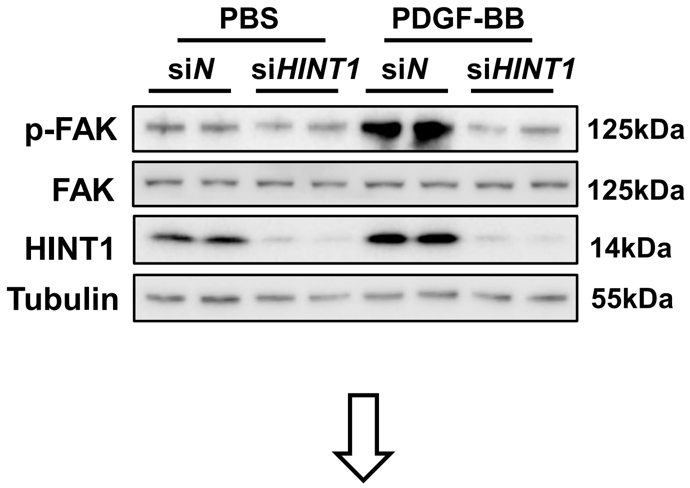

No.3-4

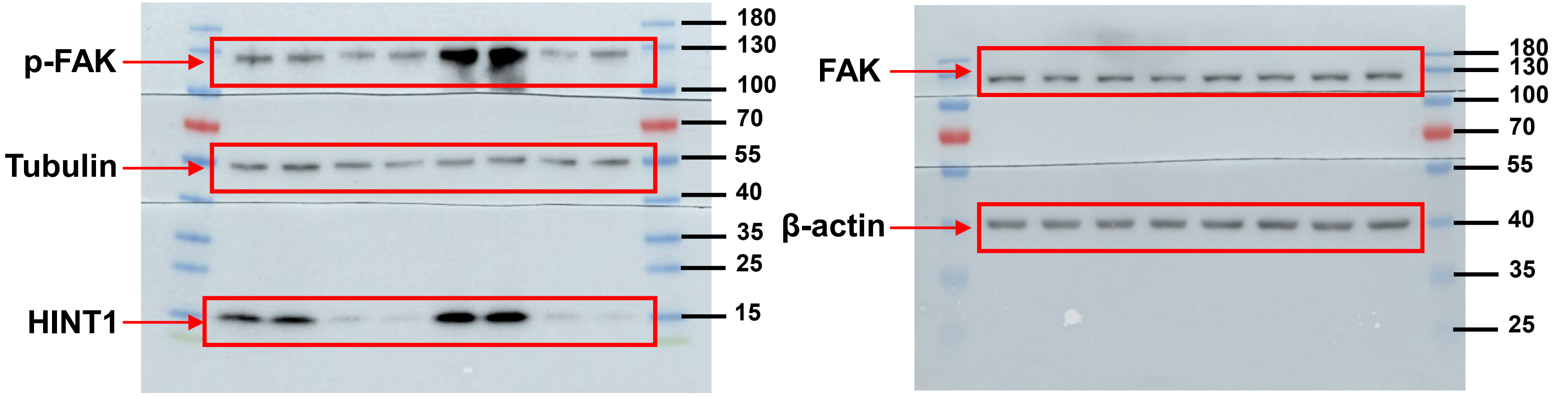

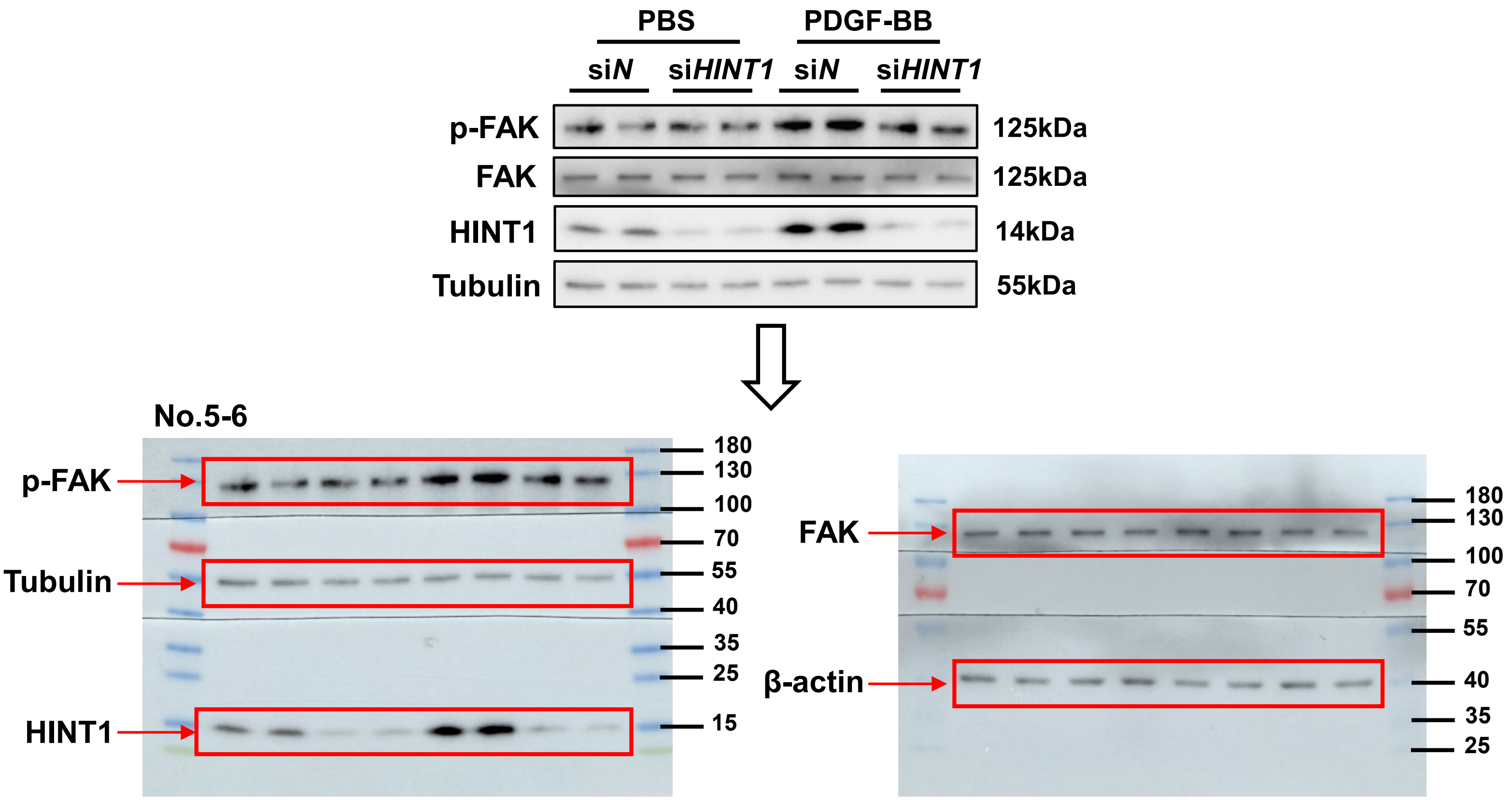

PBS
PDGF-BB
siN
siHINT1
siN
siHINT1
p-FAK
FAK
HINT1
Tubulin
125kDa
125kDa
14kDa
55kDa
No.5-6
p-FAK
Tubulin
HINT1
180
130
100
70
55
40
35
25
15
FAK
β-actin
180
130
100
70
55
40
35
25

Full unedited gel for Figure 7E n=6

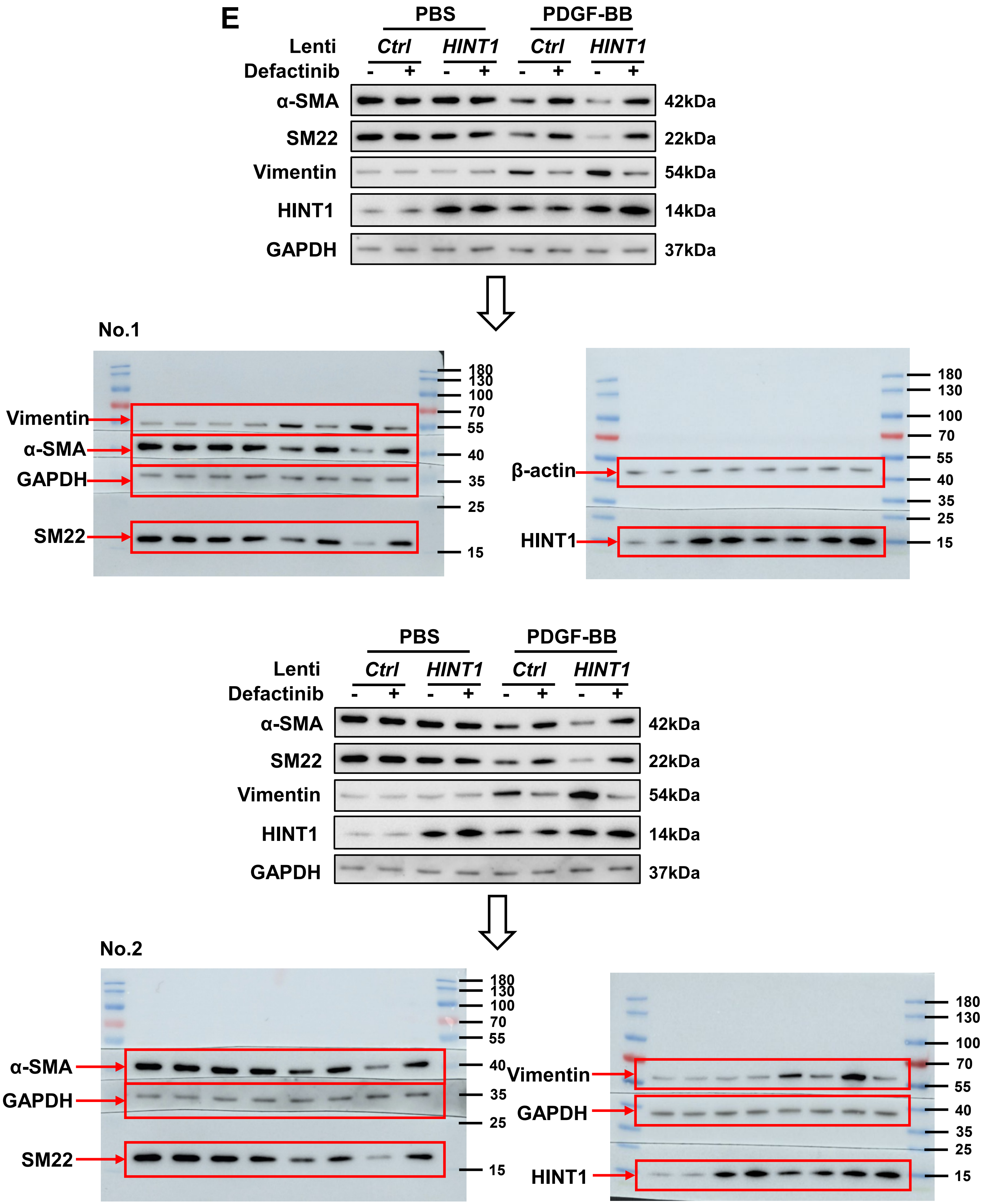

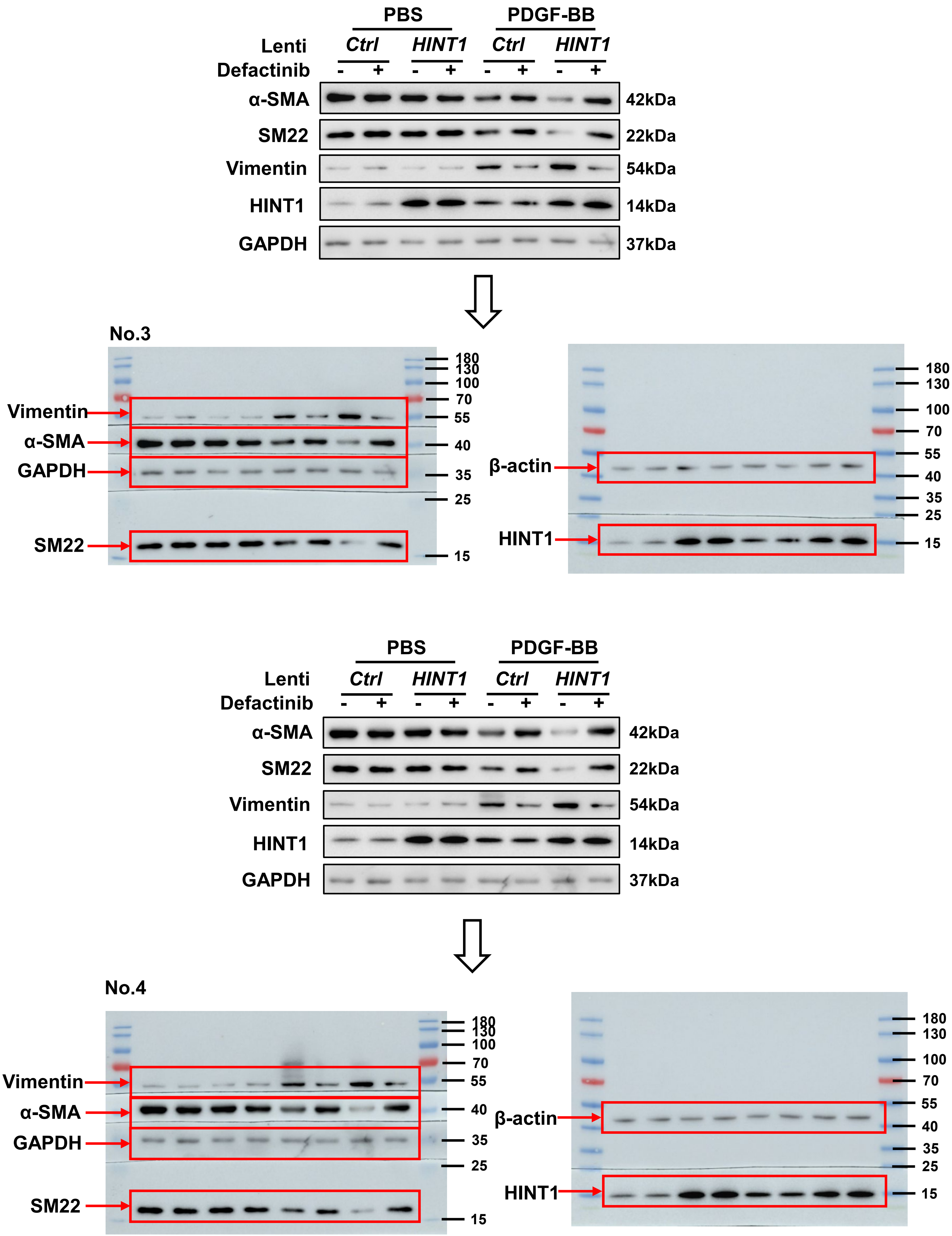
PBS
PDGF-BB
Lenti
Ctrl
HINT1
Ctrl
HINT1
Defactinib
- + - + - + - +
α-SMA
42kDa
SM22
22kDa
Vimentin
54kDa
HINT1
14kDa
GAPDH
37kDa
No.3
Vimentin
α-SMA
GAPDH
SM22
180
130
100
70
55
40
35
25
15
β-actin
HINT1
180
130
100
70
55
40
35
25
15
PBS
PDGF-BB
Lenti
Ctrl
HINT1
Ctrl
HINT1
Defactinib
- + - + - + - +
α-SMA
42kDa
SM22
22kDa
Vimentin
54kDa
HINT1
14kDa
GAPDH
37kDa
No.4
Vimentin
α-SMA
GAPDH
SM22
180
130
100
70
55
40
35
25
15
β-actin
HINT1
180
130
100
70
55
40
35
25
15

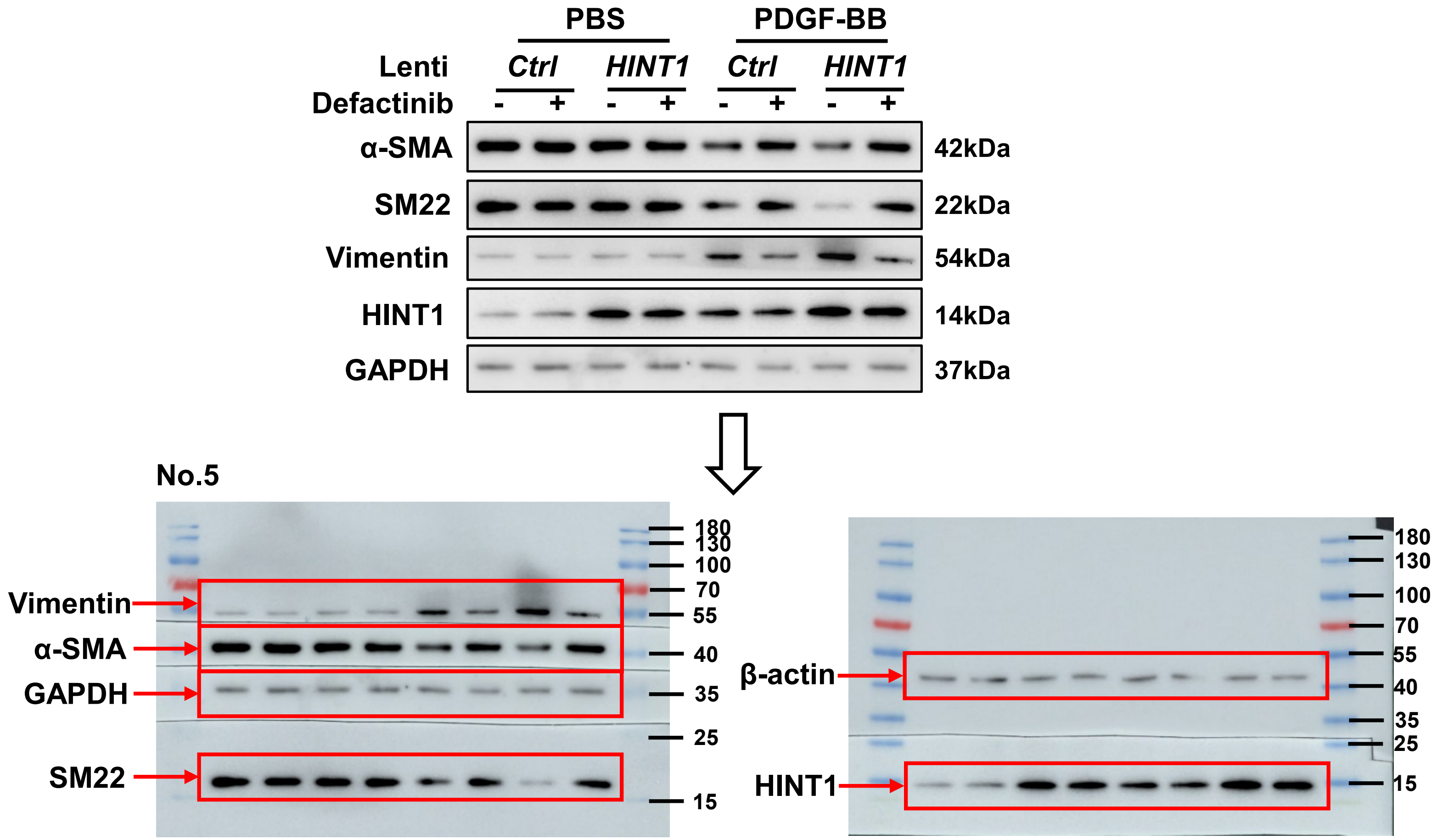
PBS
PDGF-BB
Lenti
Ctrl
HINT1
Ctrl
HINT1
Defactinib
- + - + - + - +
α-SMA
42kDa
SM22
22kDa
Vimentin
54kDa
HINT1
14kDa
GAPDH
37kDa
No.5
Vimentin
α-SMA
GAPDH
SM22
180
130
100
70
55
40
35
25
15
β-actin
HINT1
180
130
100
70
55
40
35
25
15

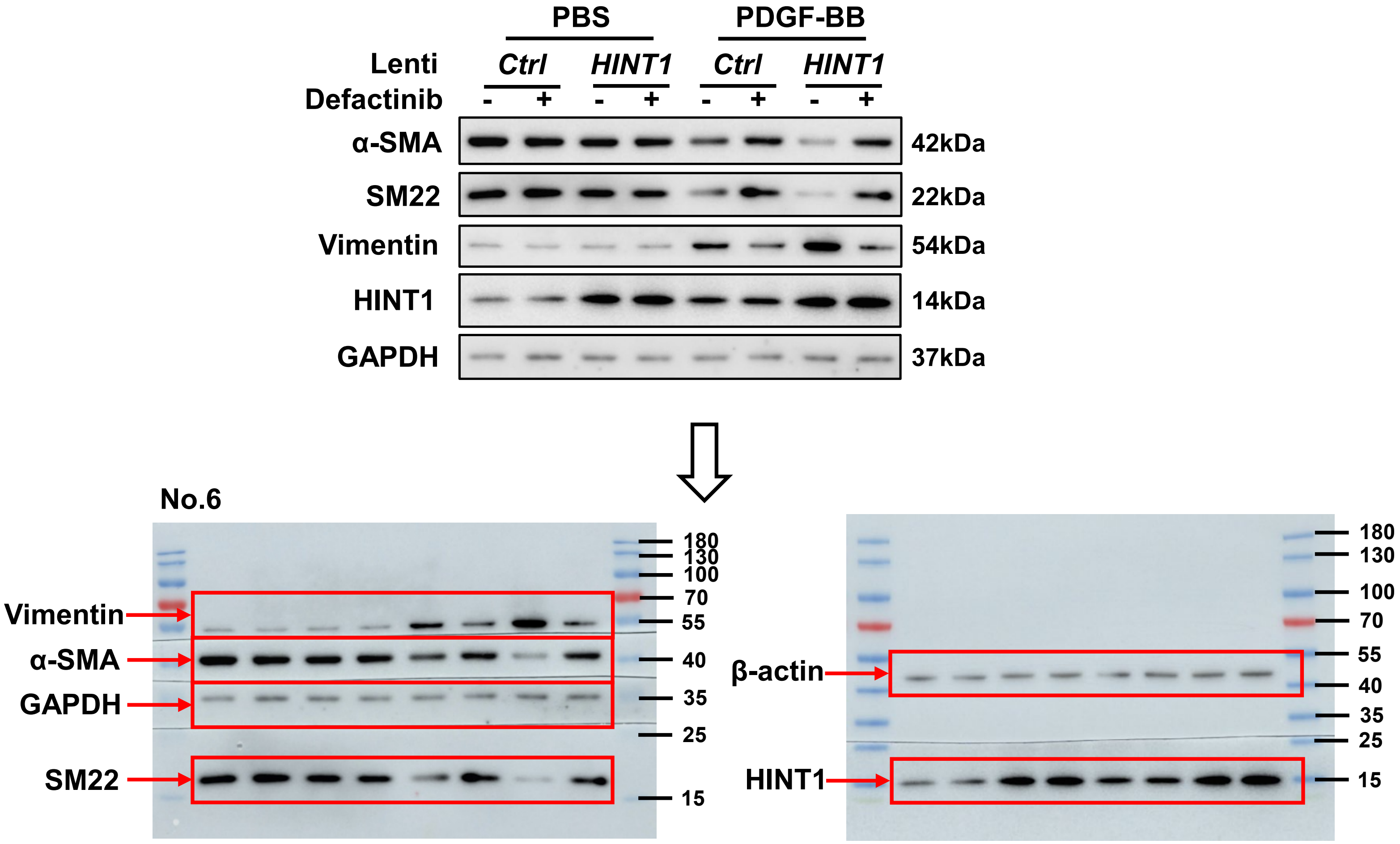
PBS
PDGF-BB
Lenti
Ctrl
HINT1
Ctrl
HINT1
Defactinib
- + - + - + - +
α-SMA
42kDa
SM22
22kDa
Vimentin
54kDa
HINT1
14kDa
GAPDH
37kDa
No.6
Vimentin
α-SMA
GAPDH
SM22
180
130
100
70
55
40
35
25
15
β-actin
HINT1
180
130
100
70
55
40
35
25
15

Full unedited gel for Figure 8H n=6

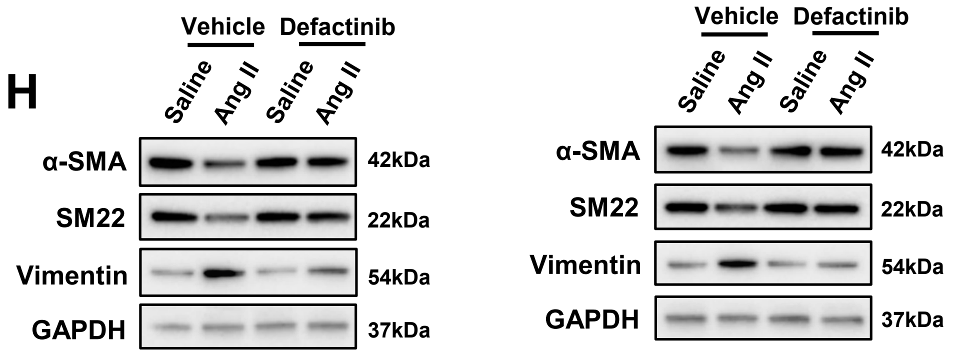

No.1-2

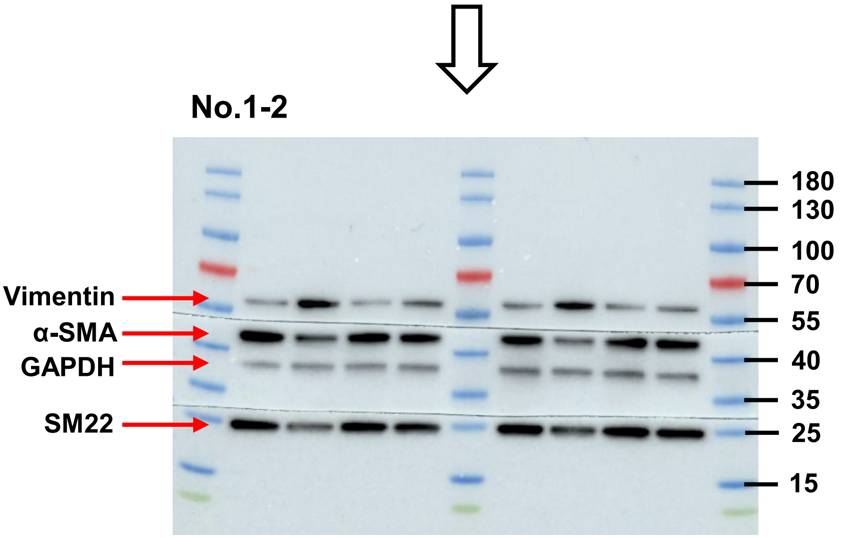

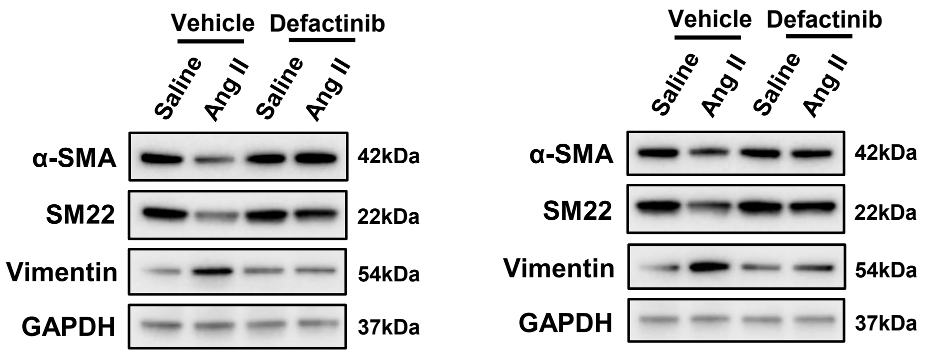

No.3-4

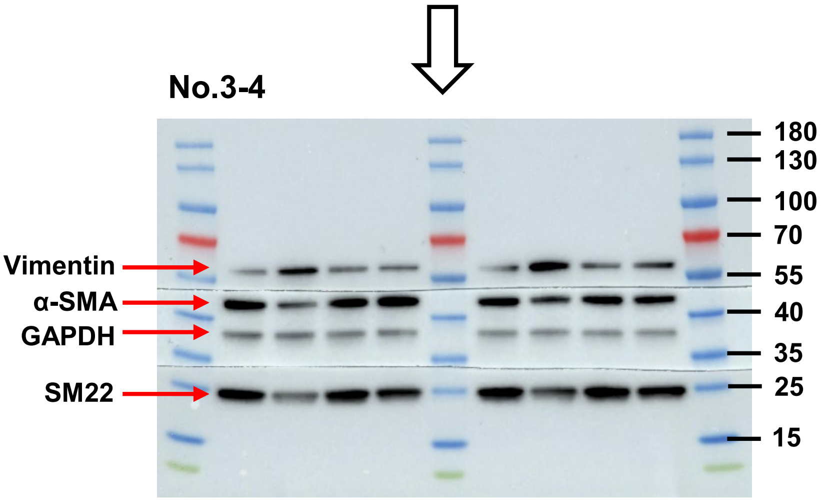

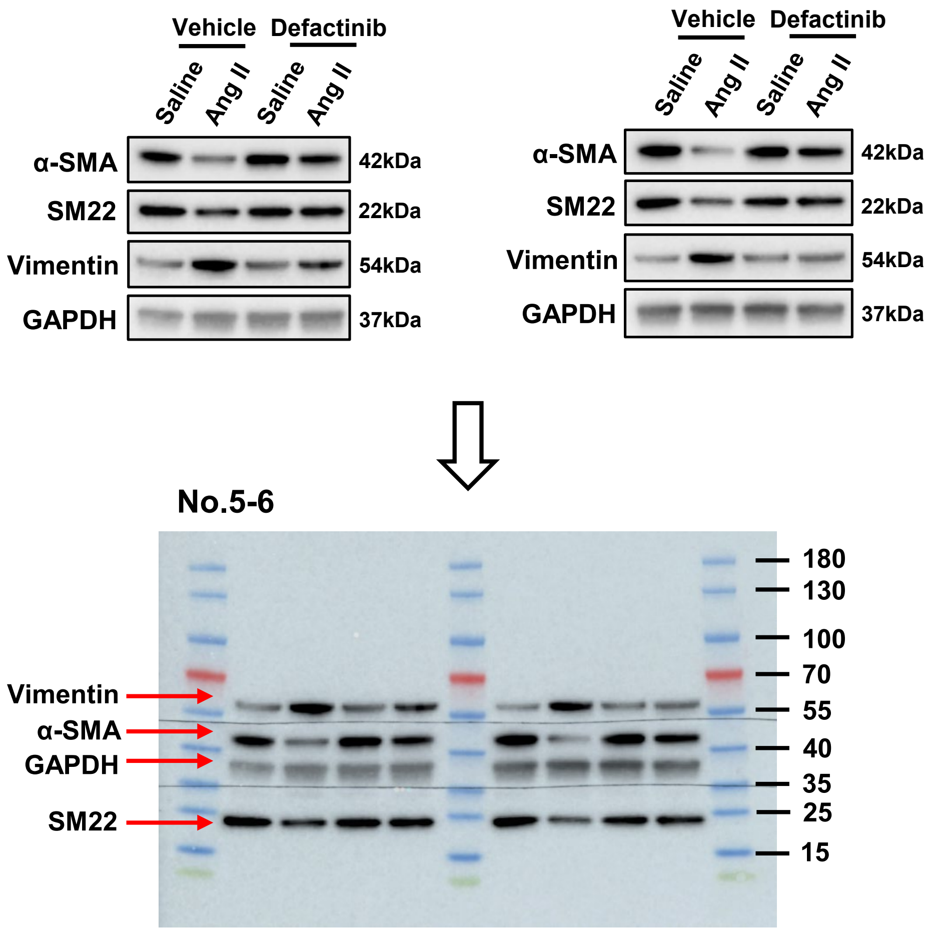

Vehicle
Defactinib
Saline
Ang II
Saline
Ang II
α-SMA
42kDa
SM22
22kDa
Vimentin
54kDa
GAPDH
37kDa
Vehicle
Defactinib
Saline
Ang II
Saline
Ang II
α-SMA
42kDa
SM22
22kDa
Vimentin
54kDa
GAPDH
37kDa
No.5-6
Vimentin
α-SMA
GAPDH
SM22
180
130
100
70
55
40
35
25
15

Full unedited gel for Supplemental Figure1A n=6

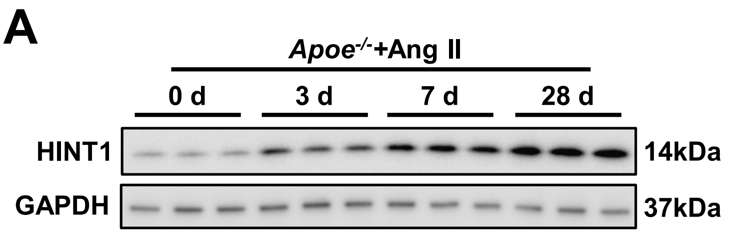

No.1-3

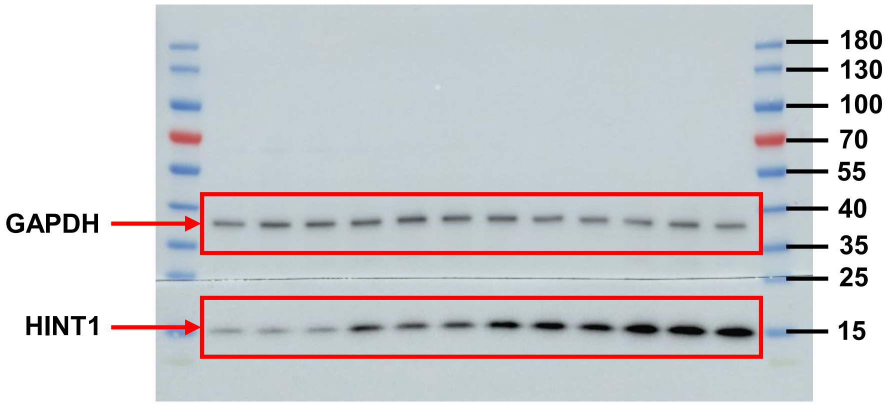

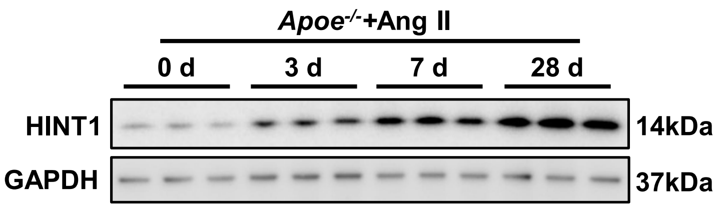

No.4-6

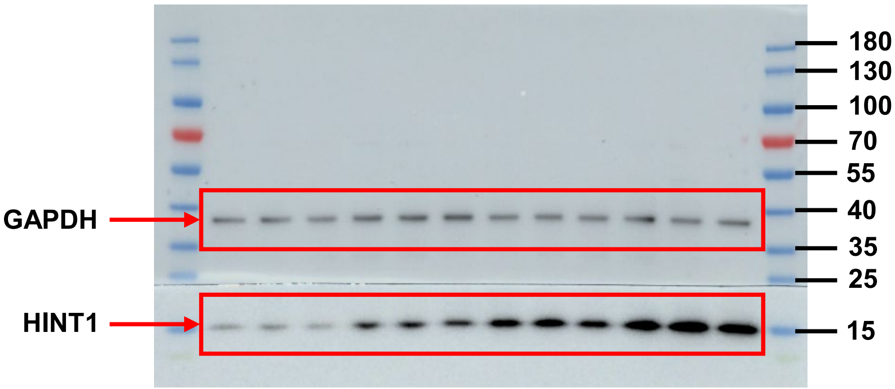

Full unedited gel for Supplemental Figure 1C n=3

C

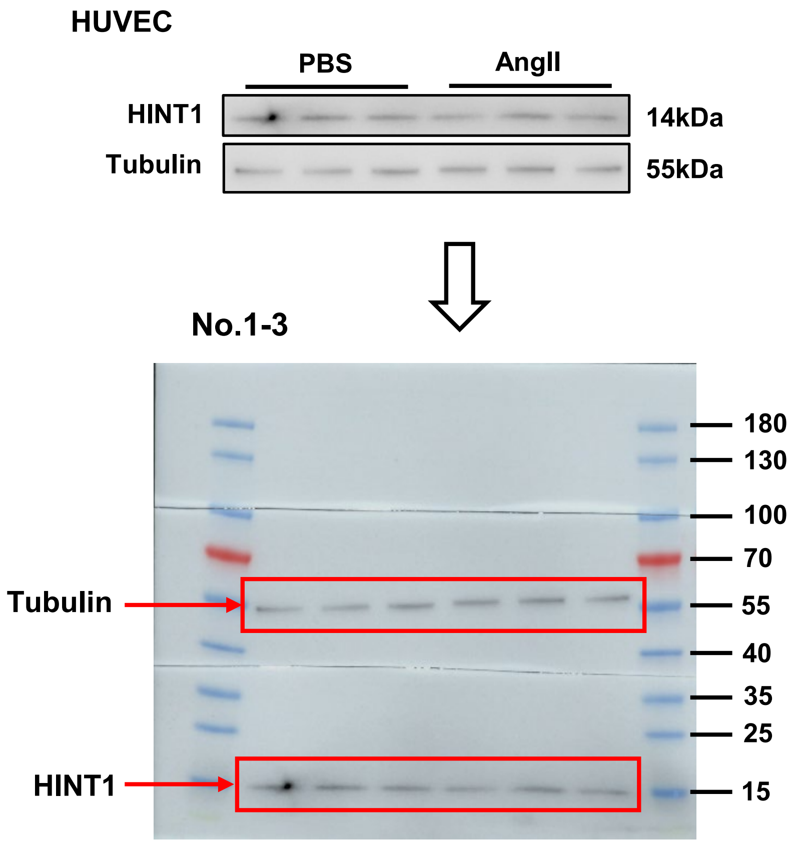

Full unedited gel for Supplemental Figure 1E n=3

E

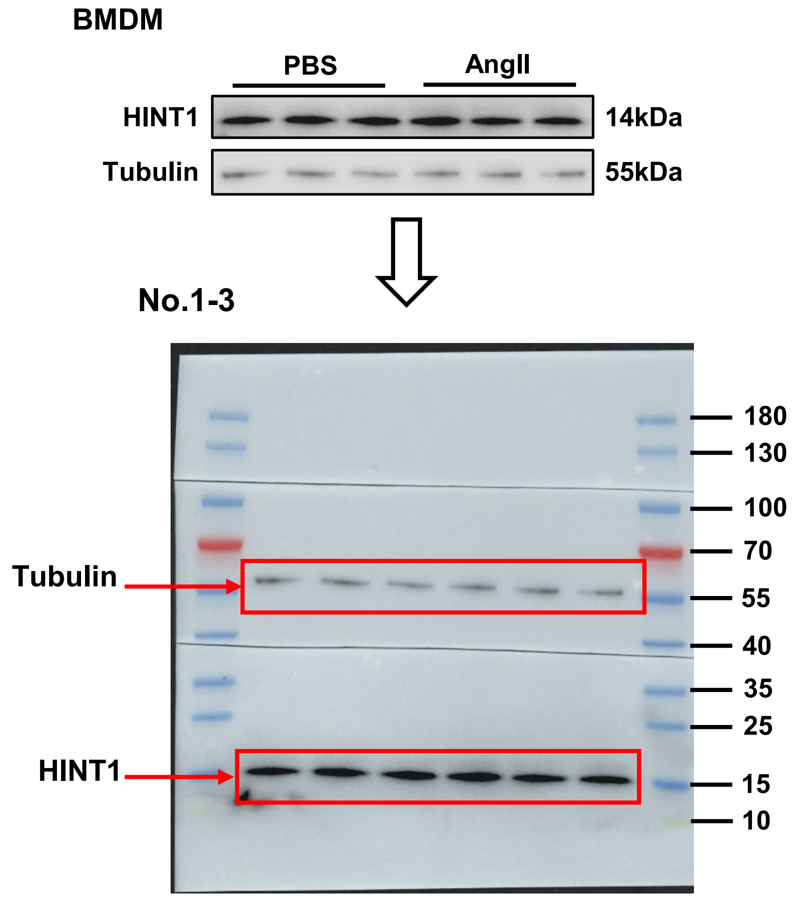

Full unedited gel for Supplemental Figure 2A n=3

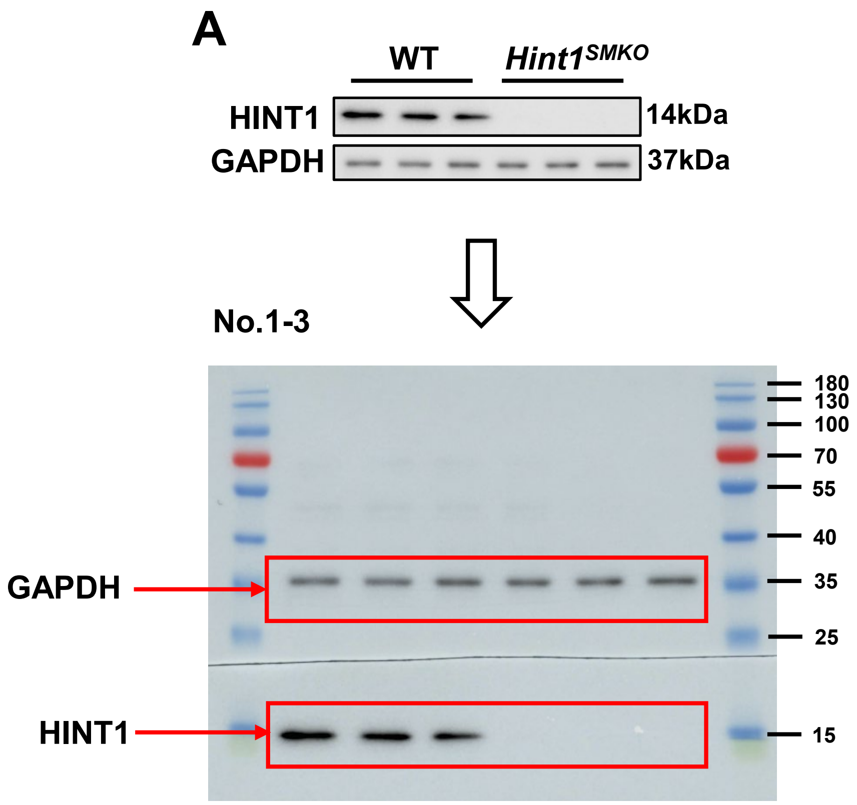

Full unedited gel for Supplemental Figure 3B n=6

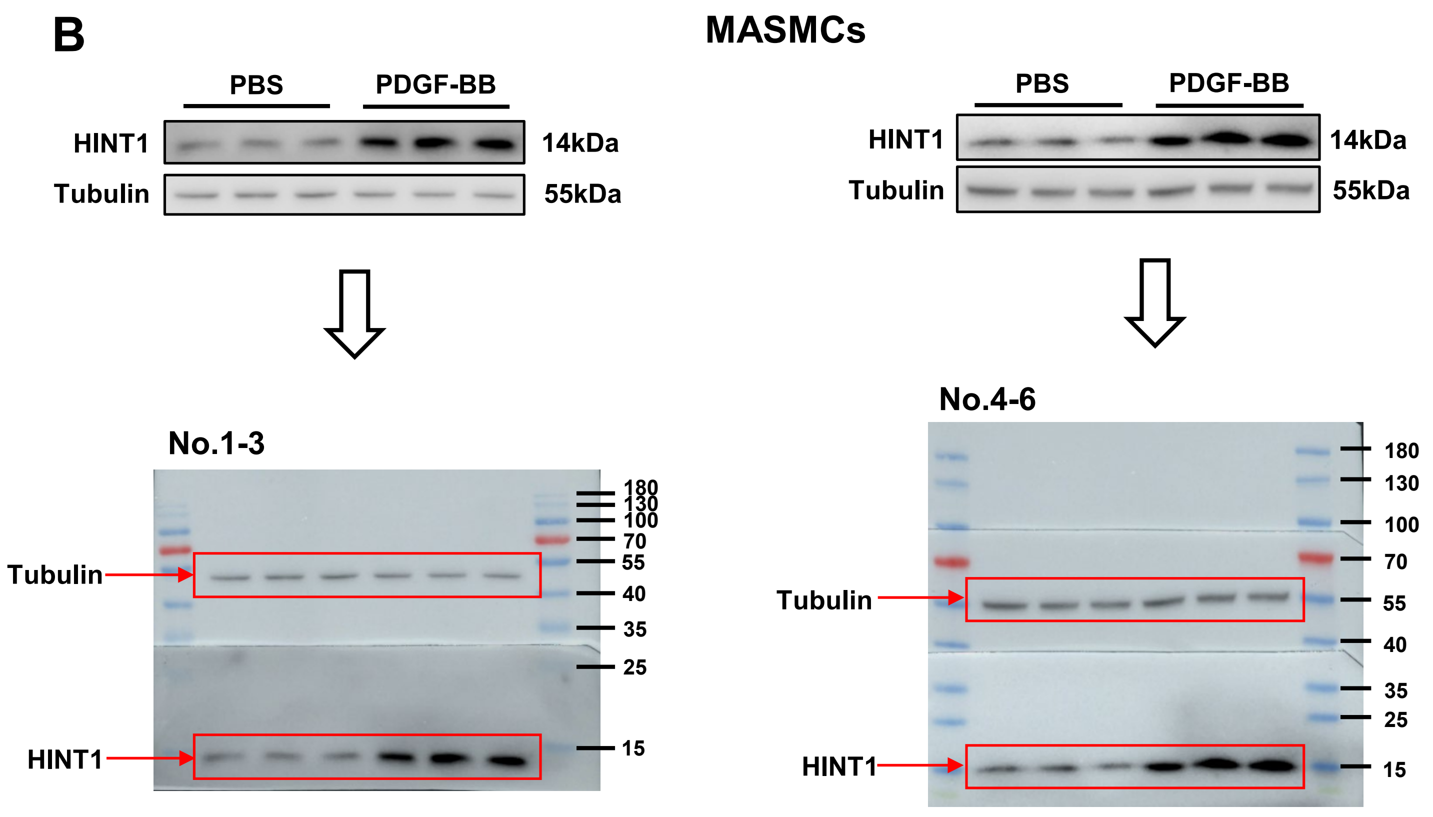

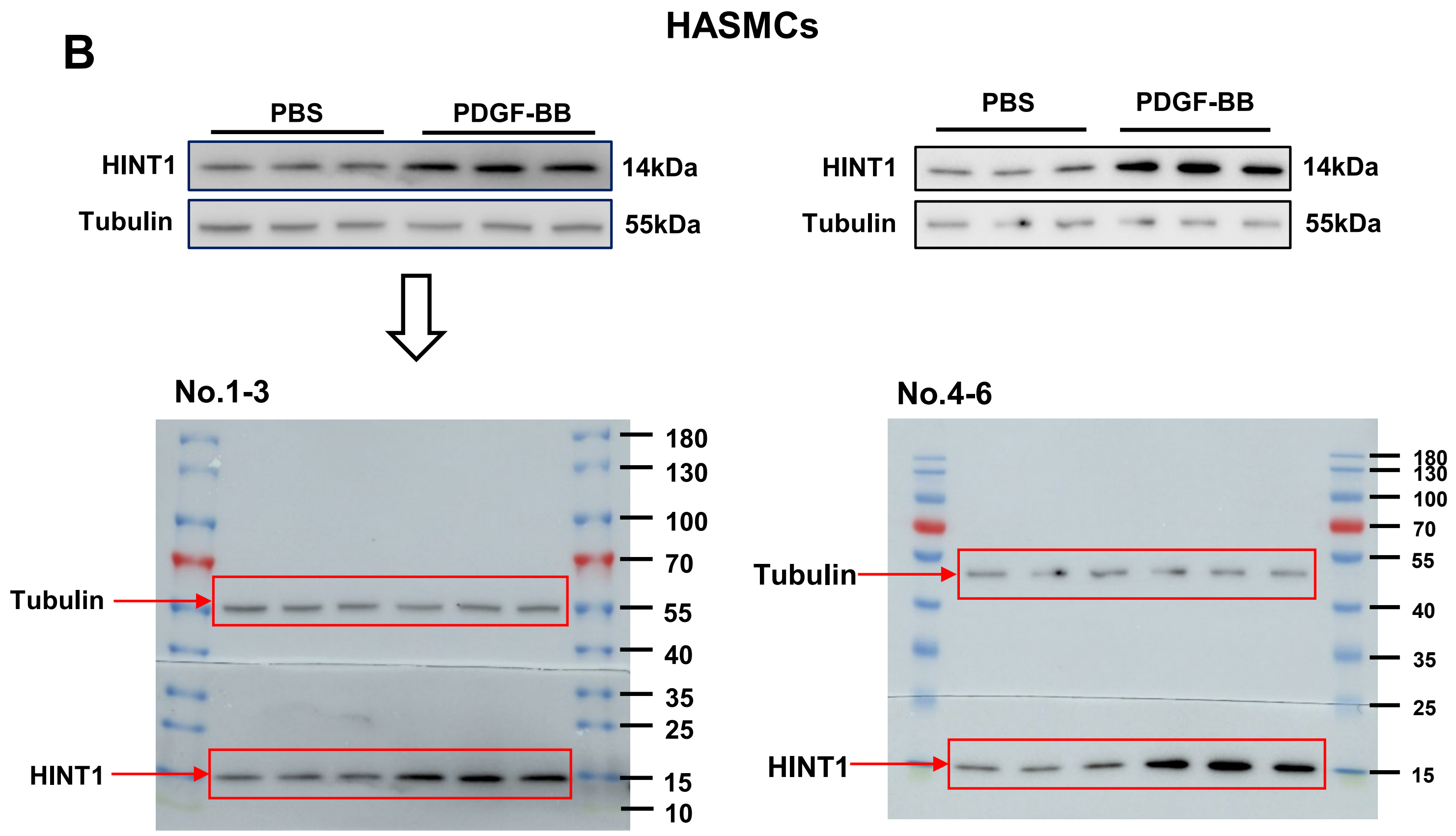

B

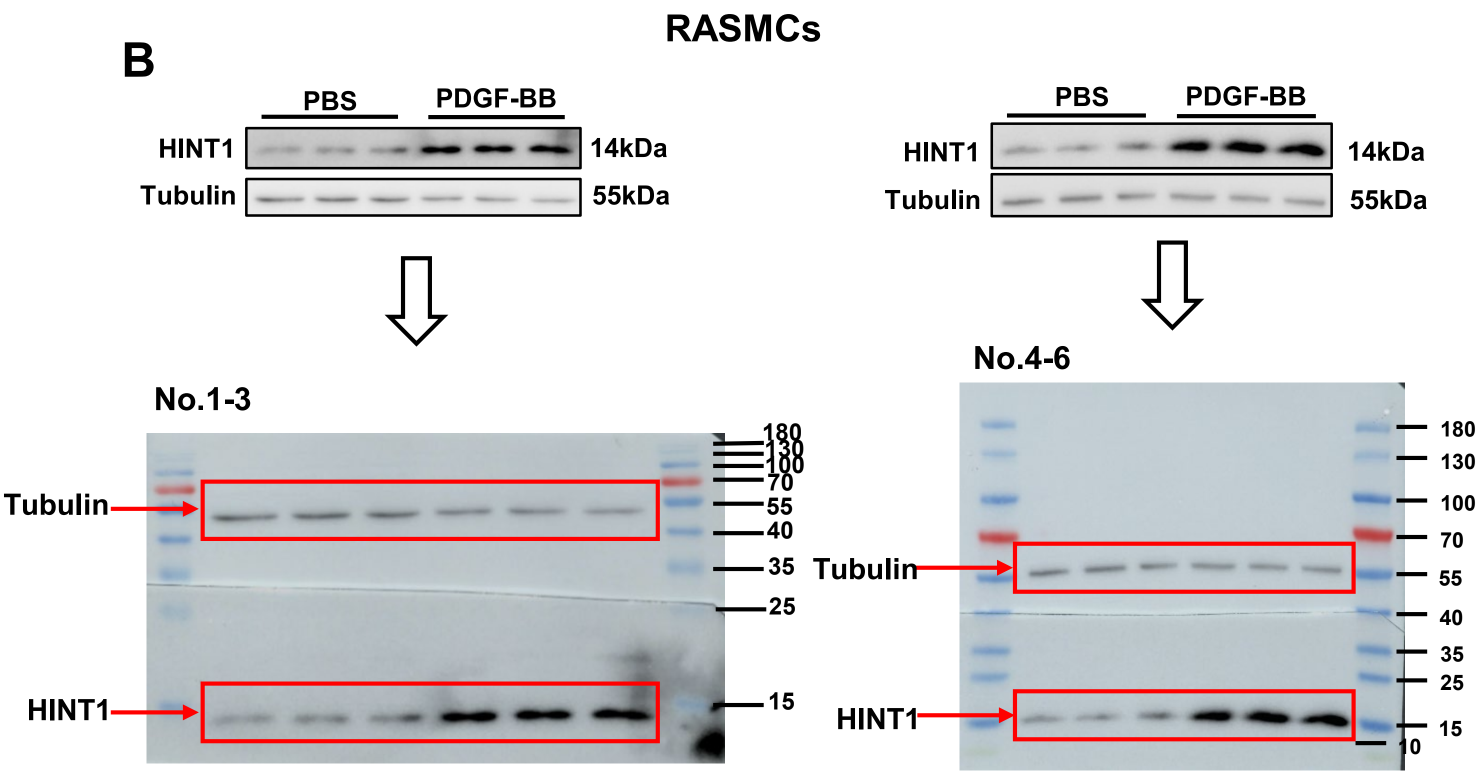

RASMCs
PBS
PDGF-BB
HINT1
14kDa
Tubulin
55kDa
No.1-3
180
130
100
70
55
40
35
25
15
Tubulin
HINT1
No.4-6
180
130
100
70
55
40
35
25
15
10

Full unedited gel for Supplemental Figure 3C n=6

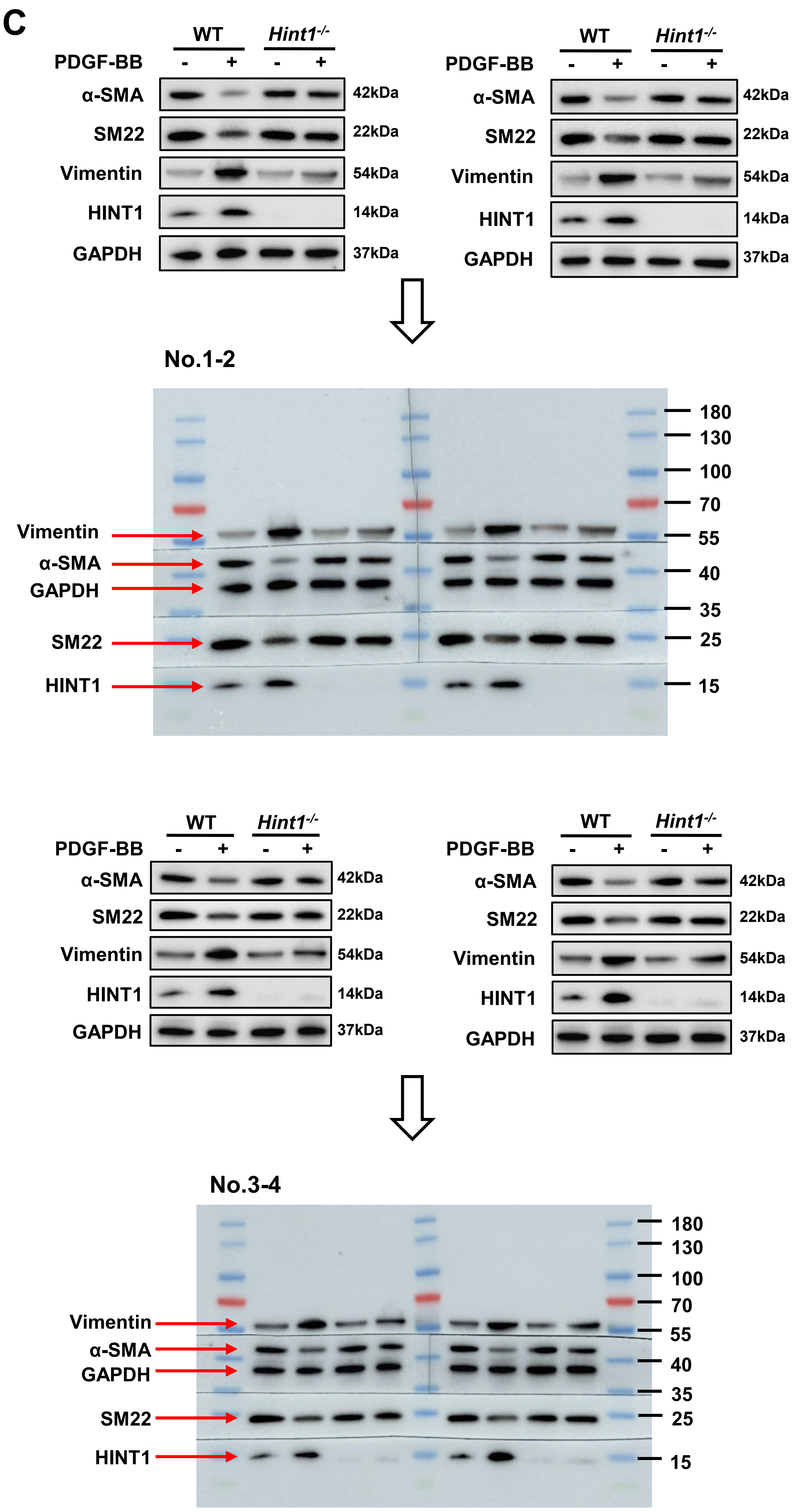

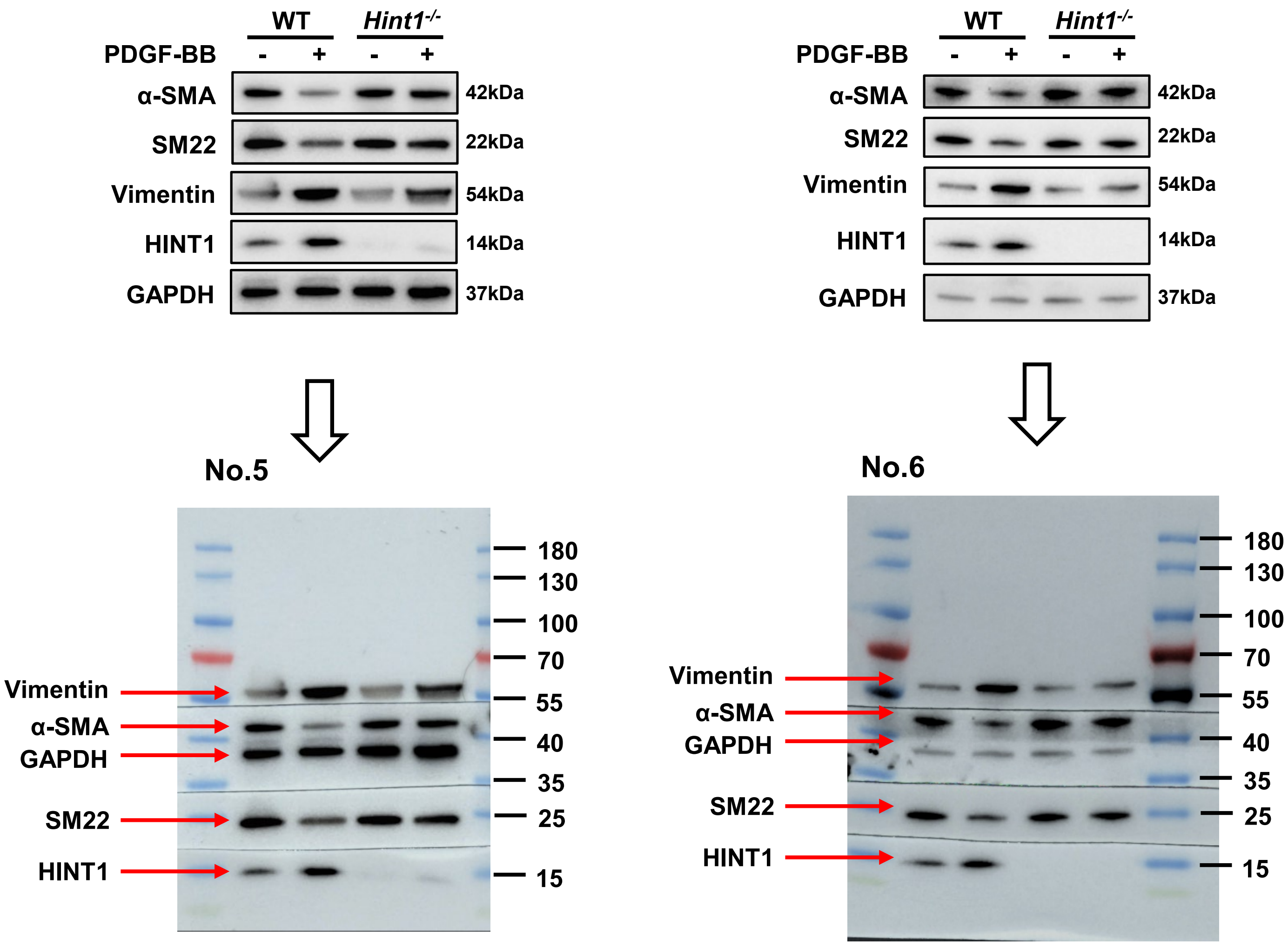

WT
Hint1-/-
PDGF-BB
- + - +
α-SMA
SM22
Vimentin
HINT1
GAPDH
42kDa
22kDa
54kDa
14kDa
37kDa
No.5
No.6
180
130
100
70
55
40
35
25
15

Full unedited gel for Supplemental Figure 3F n=6

F

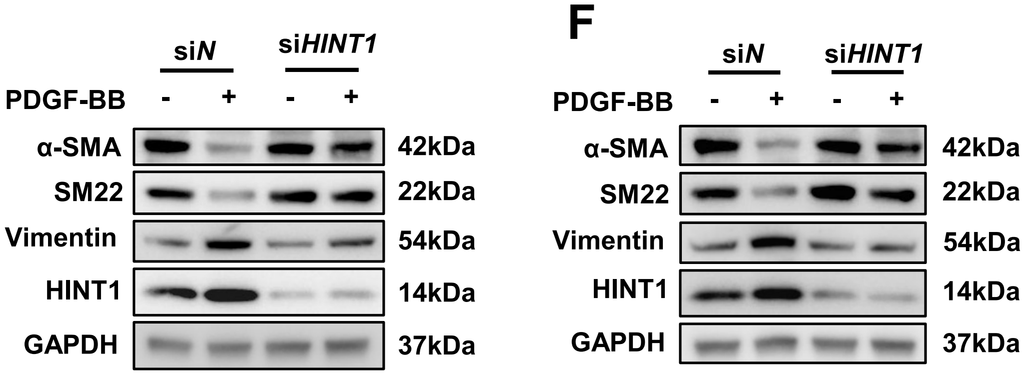

No.1-2

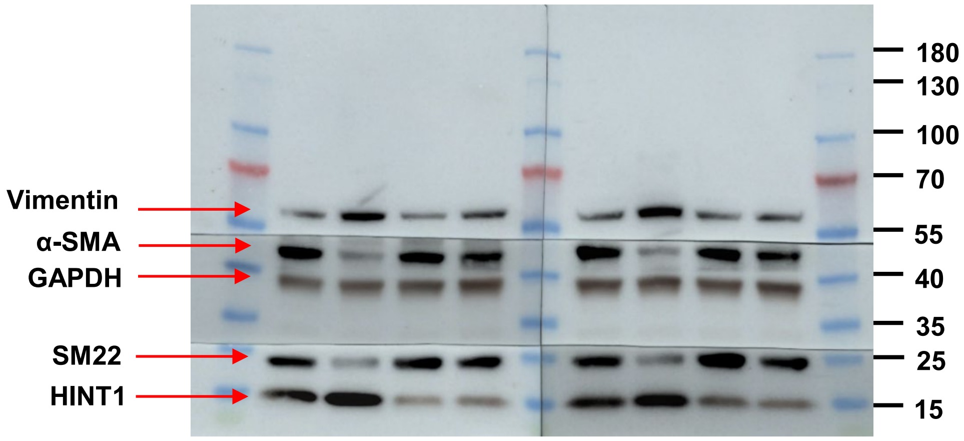

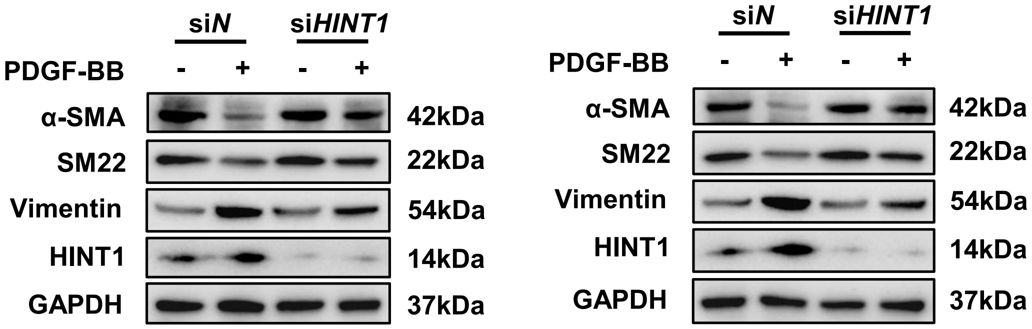

No.3-4

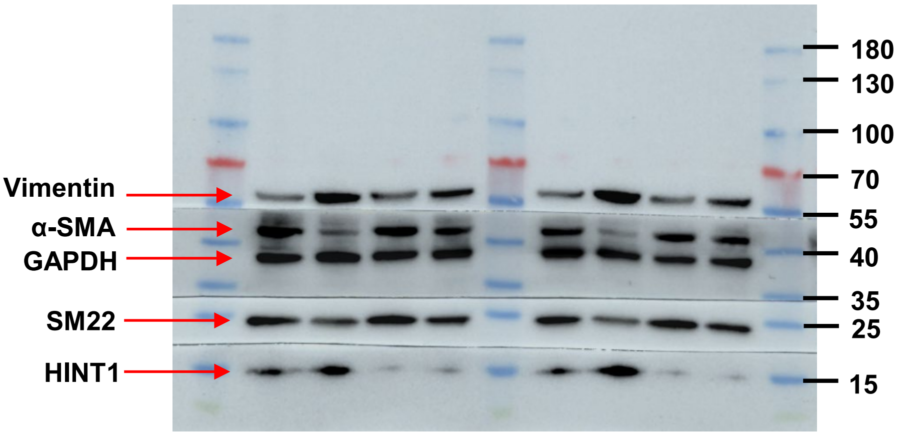

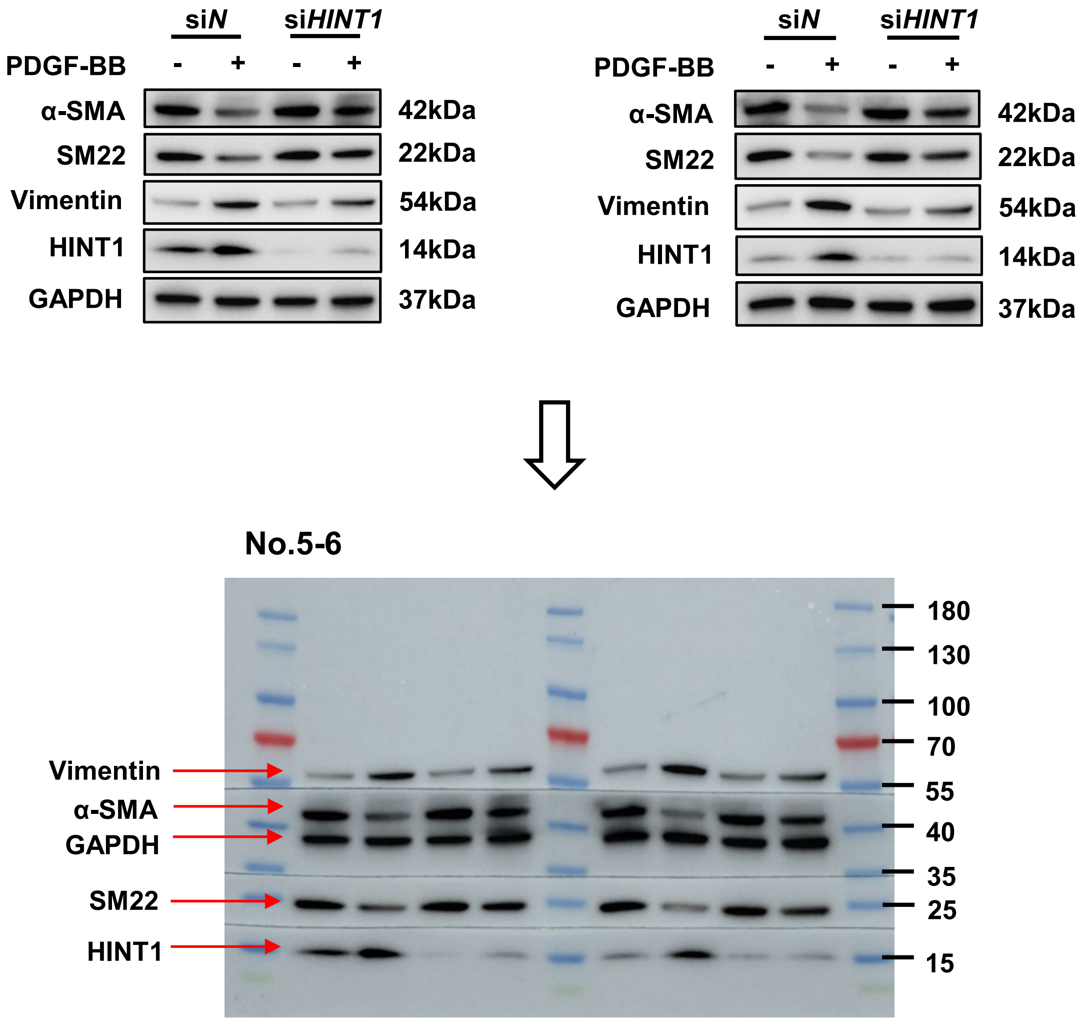
siN
siHINT1
PDGF-BB
- + - +
α-SMA
SM22
Vimentin
HINT1
GAPDH
42kDa
22kDa
54kDa
14kDa
37kDa
siN
siHINT1
PDGF-BB
- + - +
α-SMA
SM22
Vimentin
HINT1
GAPDH
42kDa
22kDa
54kDa
14kDa
37kDa
No.5-6
Vimentin
α-SMA
GAPDH
SM22
HINT1
180
130
100
70
55
40
35
25
15

Full unedited gel for Supplemental Figure 4A n=6

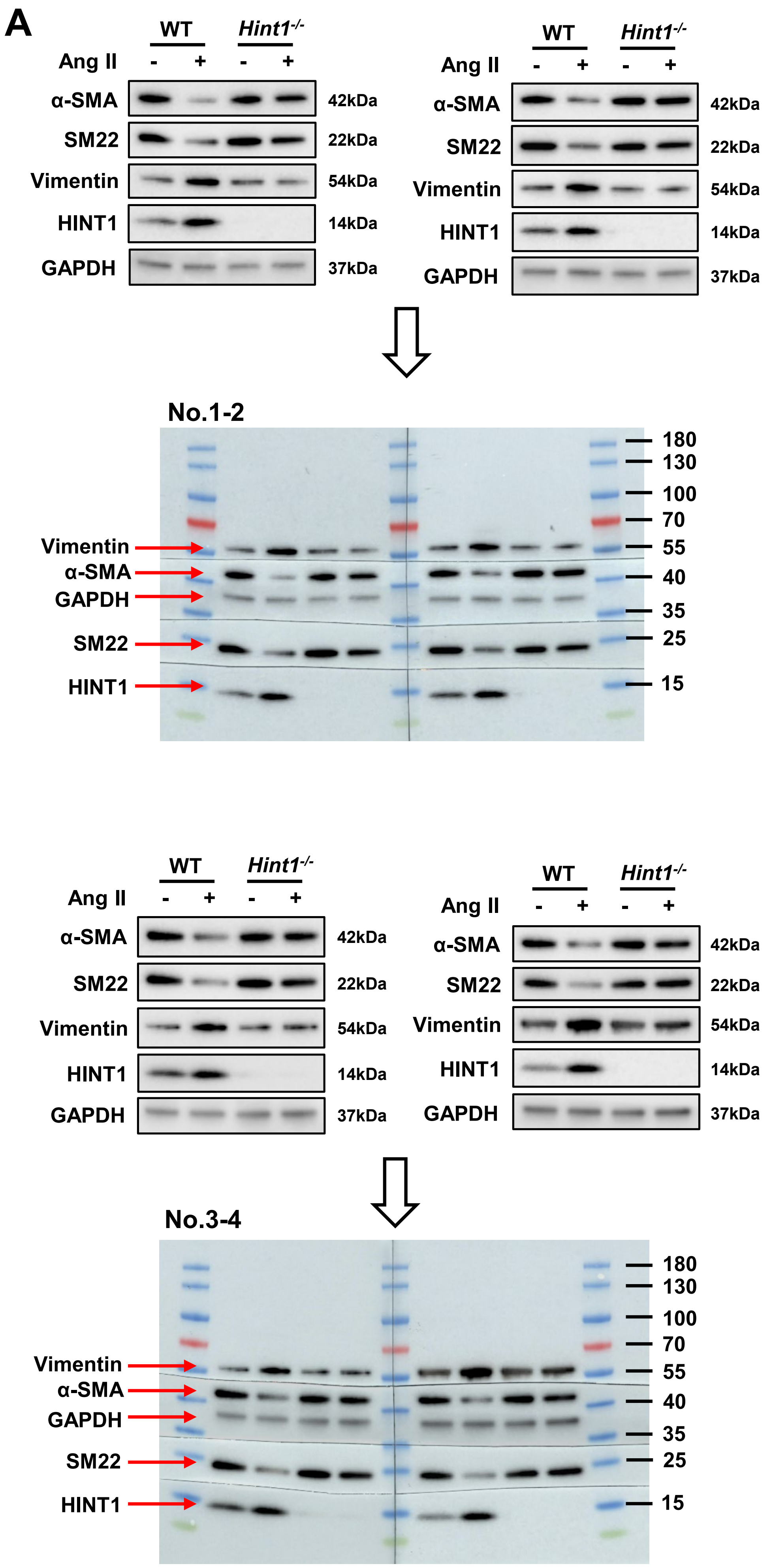

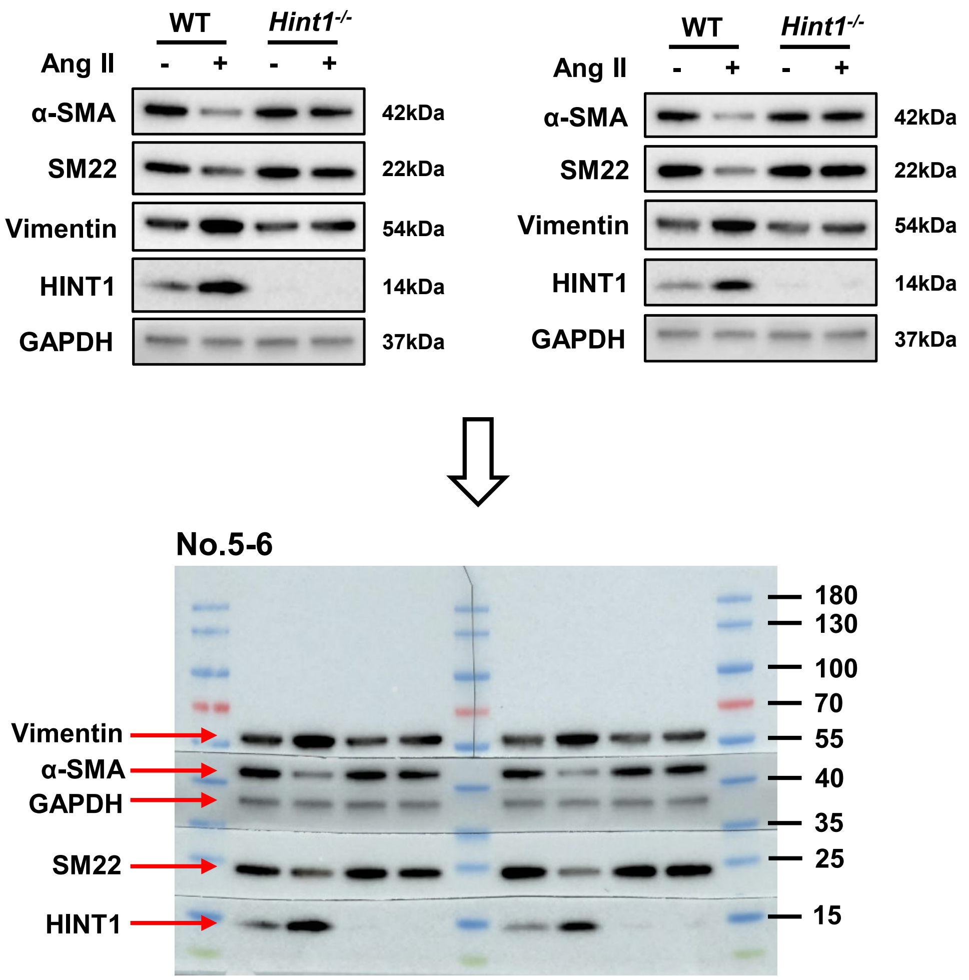
WT
Hint1-/-
Ang II
- + - +
α-SMA
SM22
Vimentin
HINT1
GAPDH
42kDa
22kDa
54kDa
14kDa
37kDa
No.5-6
180
130
100
70
55
40
35
25
15

Full unedited gel for Supplemental Figure 4D n=6

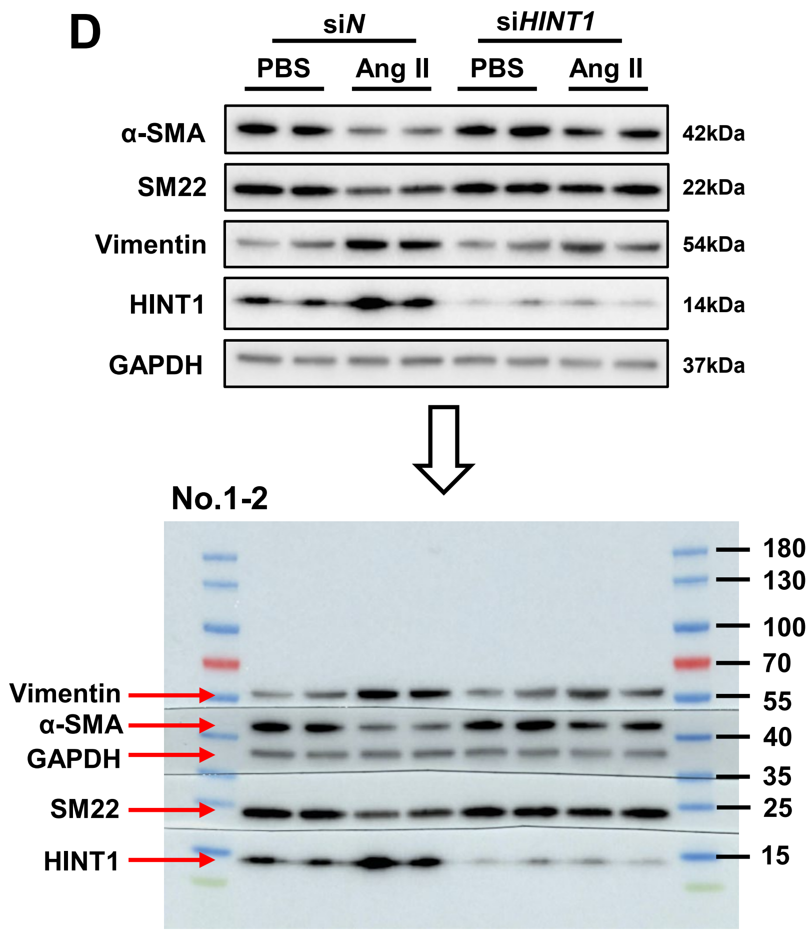

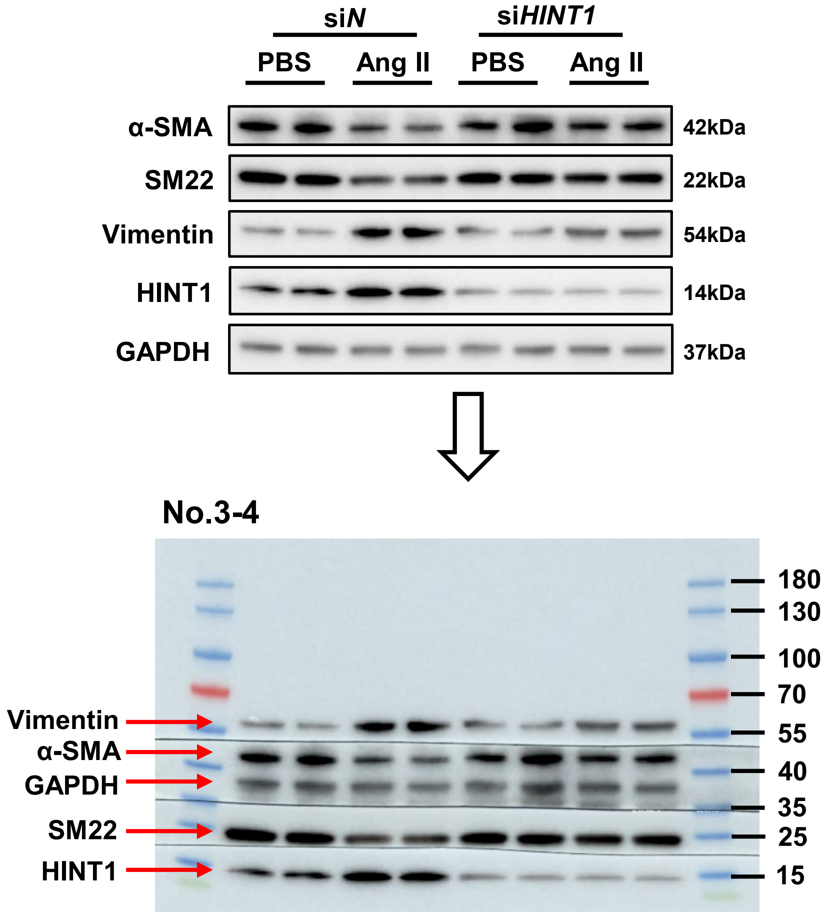

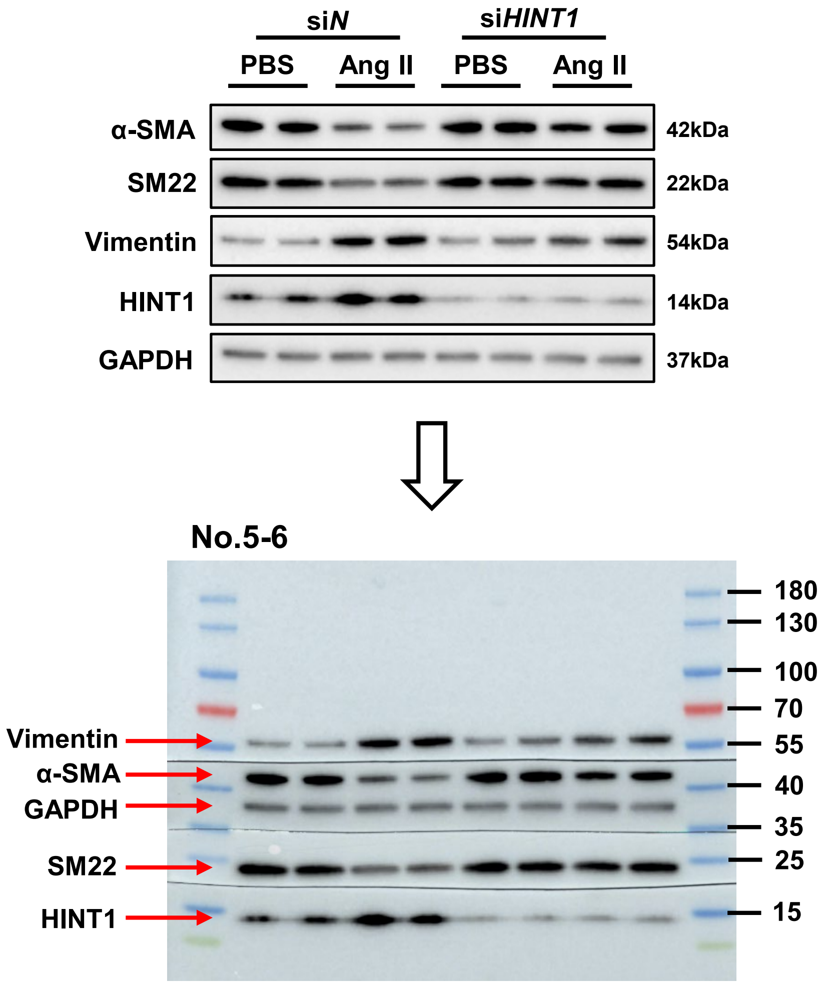
siN
siHINT1
PBS
Ang II
PBS
Ang II
α-SMA
SM22
Vimentin
HINT1
GAPDH
42kDa
22kDa
54kDa
14kDa
37kDa
No.5-6
Vimentin
α-SMA
GAPDH
SM22
HINT1
180
130
100
70
55
40
35
25
15

Full unedited gel for Supplemental Figure 5C n=6

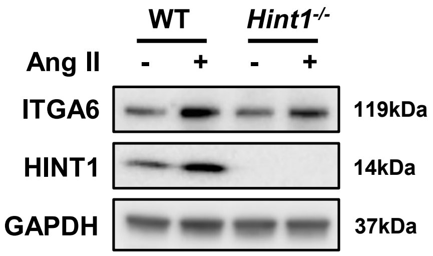

C

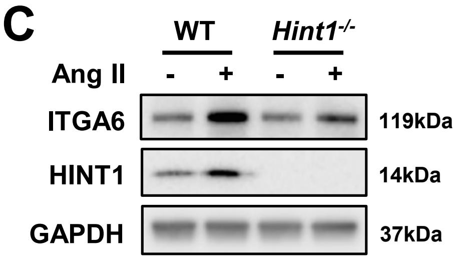

No.1-2

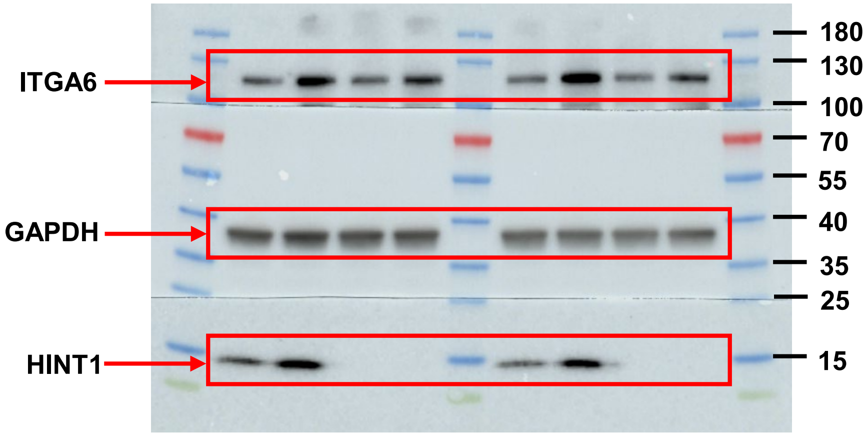

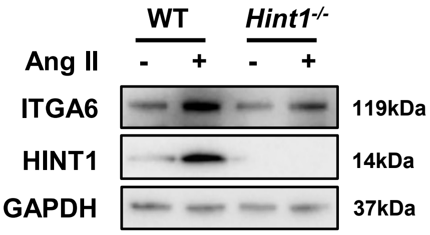

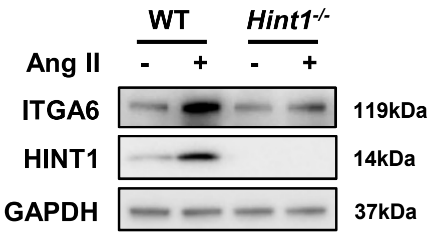

No.3-4

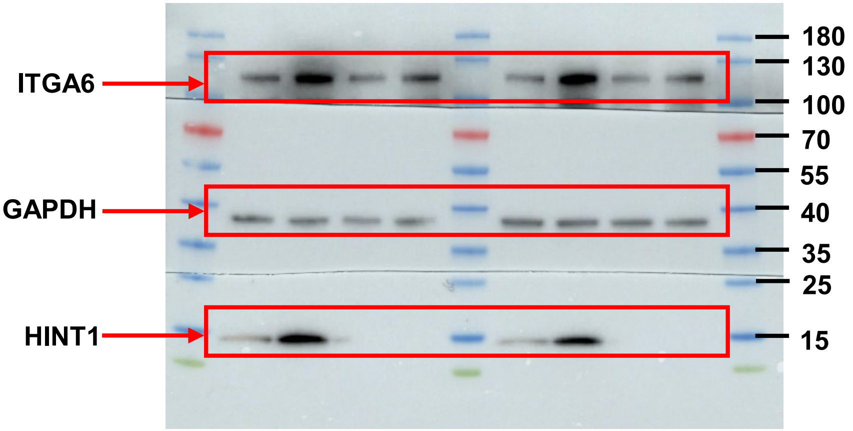

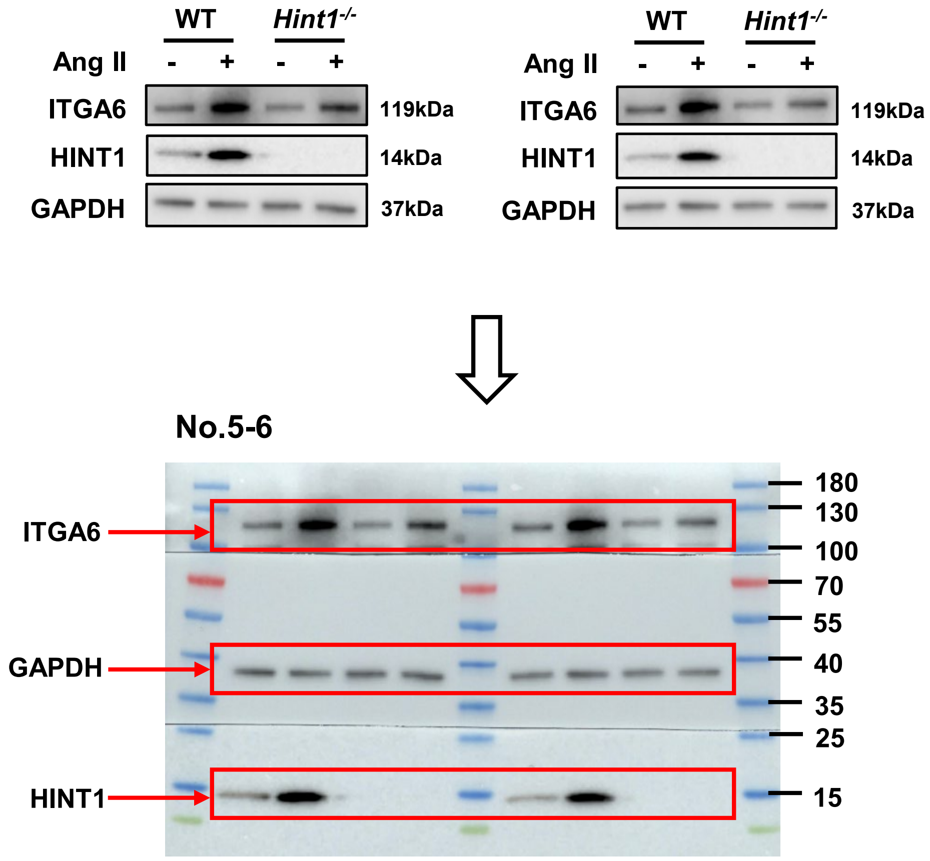
WT
Hint1-/-
Ang II
-
+
-
+
ITGA6
119kDa
HINT1
14kDa
GAPDH
37kDa
WT
Hint1-/-
Ang II
-
+
-
+
ITGA6
119kDa
HINT1
14kDa
GAPDH
37kDa
No.5-6
ITGA6
GAPDH
HINT1
180
130
100
70
55
40
35
25
15

Full unedited gel for Supplemental Figure 5E n=6

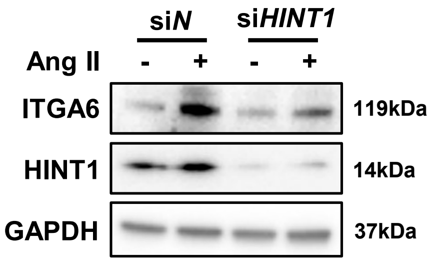

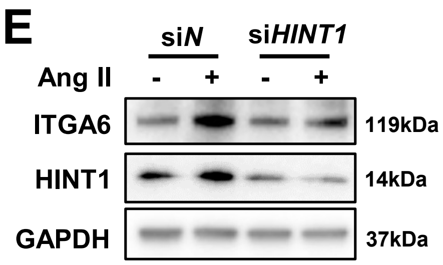

No.1-2

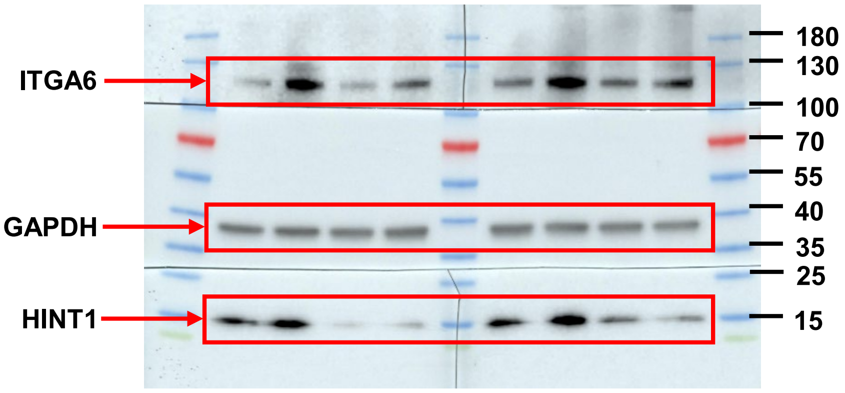

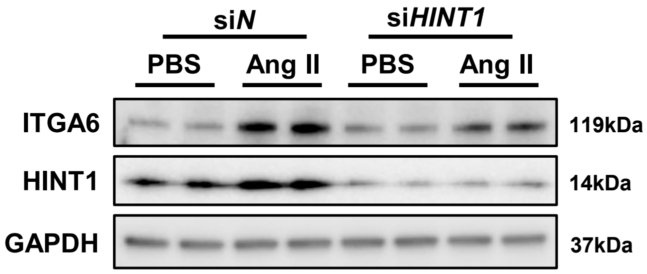

No.3-4

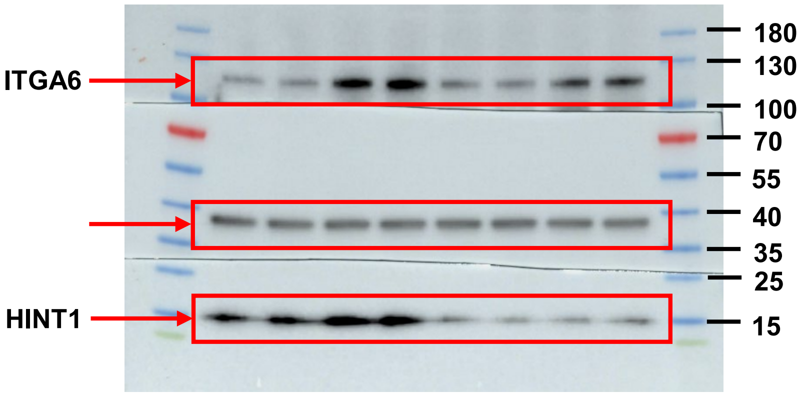

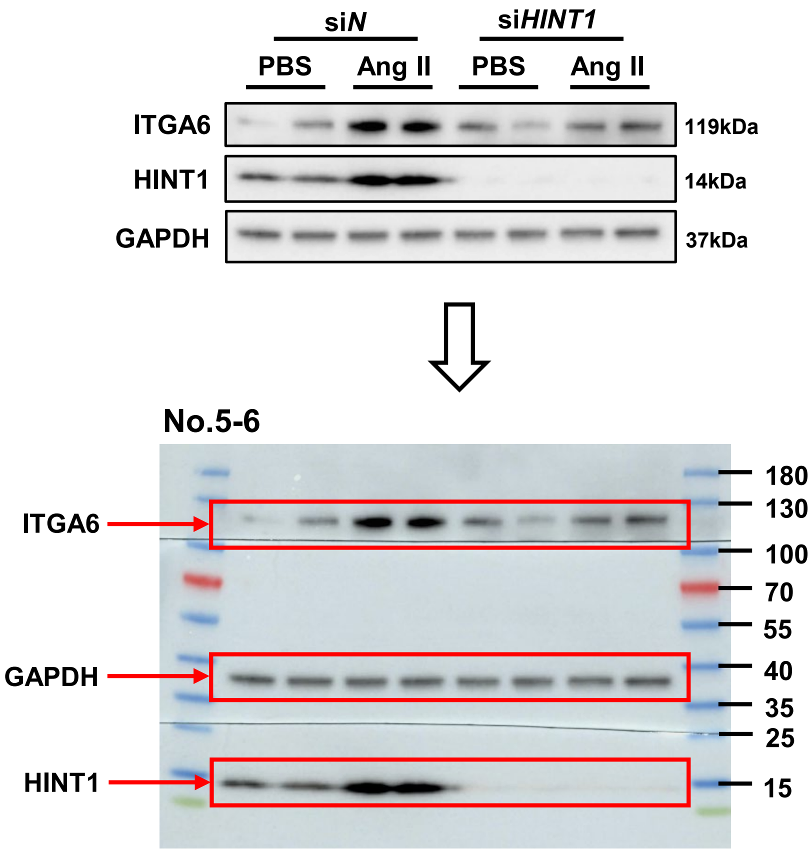

siN
siHINT1
PBS
Ang II
PBS
Ang II
ITGA6
HINT1
GAPDH
119kDa
14kDa
37kDa
No.5-6
ITGA6
GAPDH
HINT1
180
130
100
70
55
40
35
25
15

Full unedited gel for Supplemental Figure 6A n=6

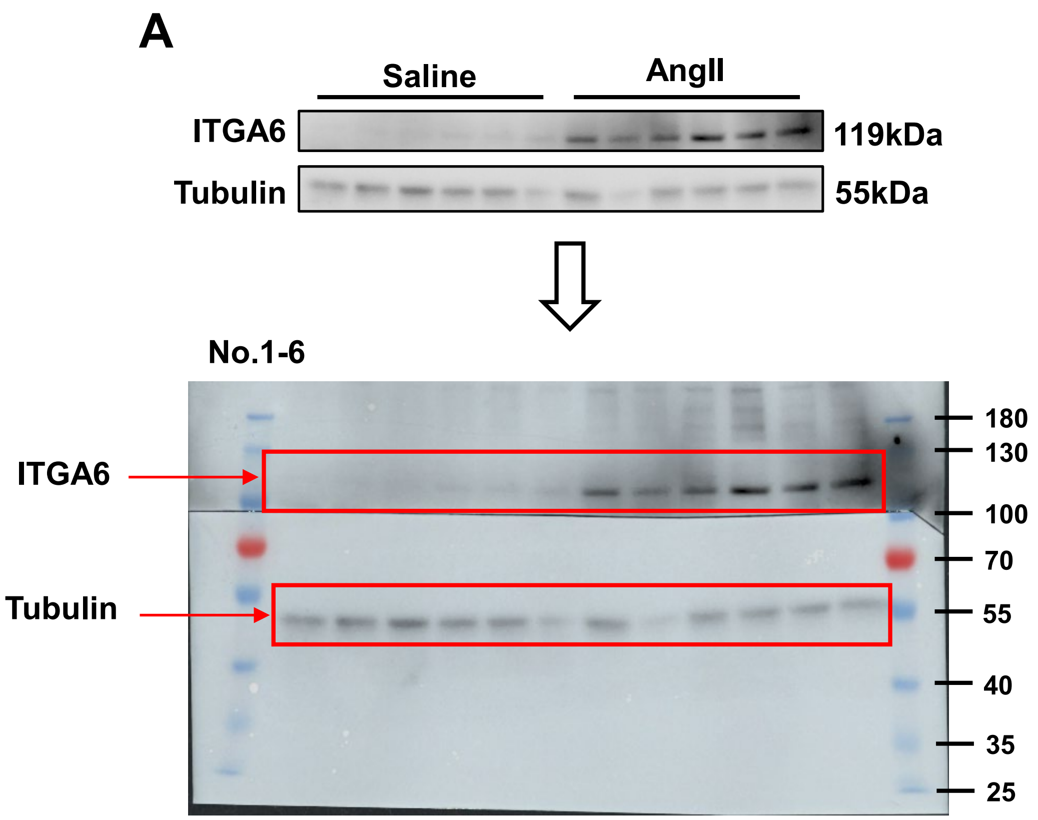

Full unedited gel for Supplemental Figure 6E n=7

MASMCs

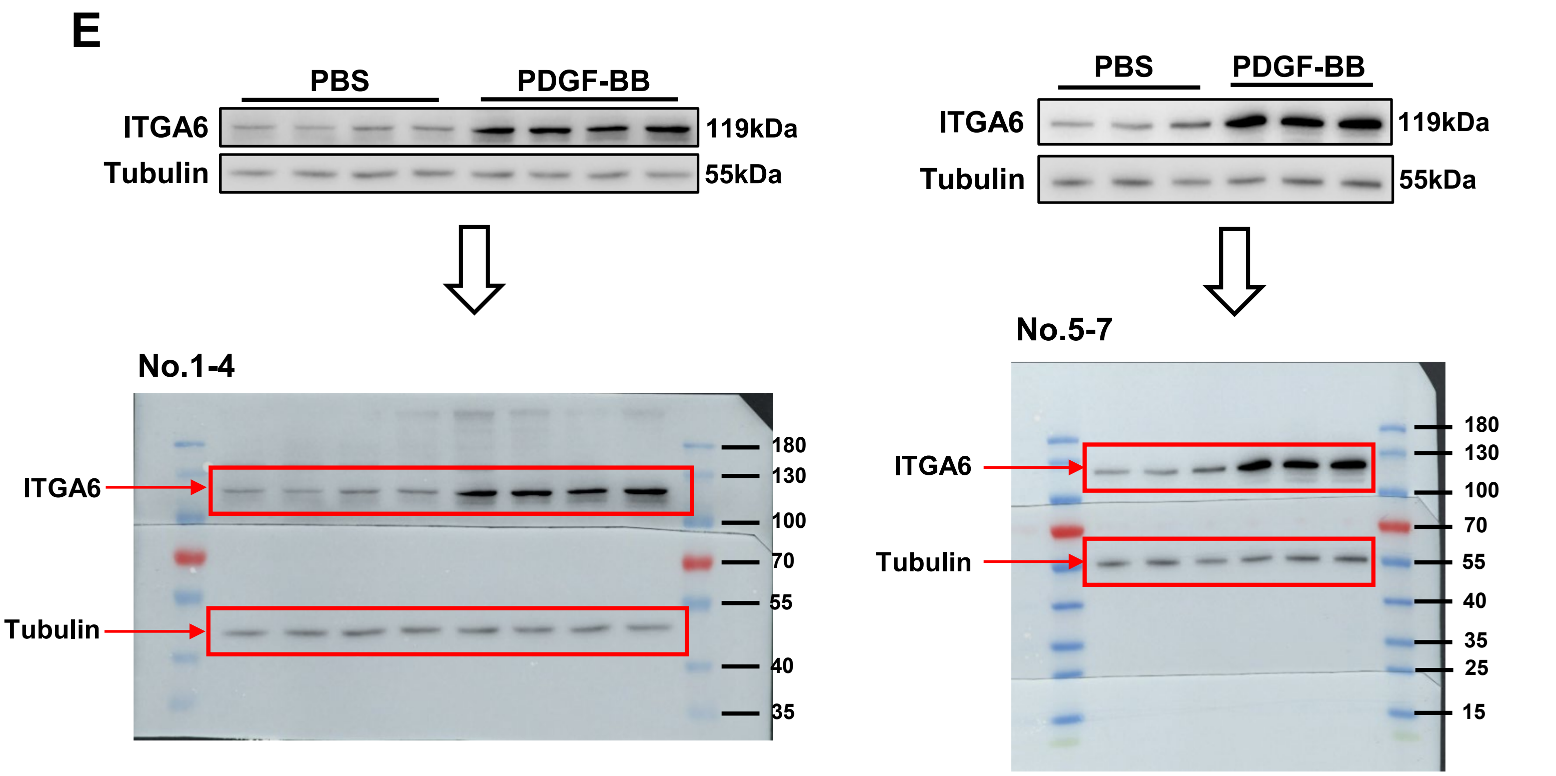

HASMCs

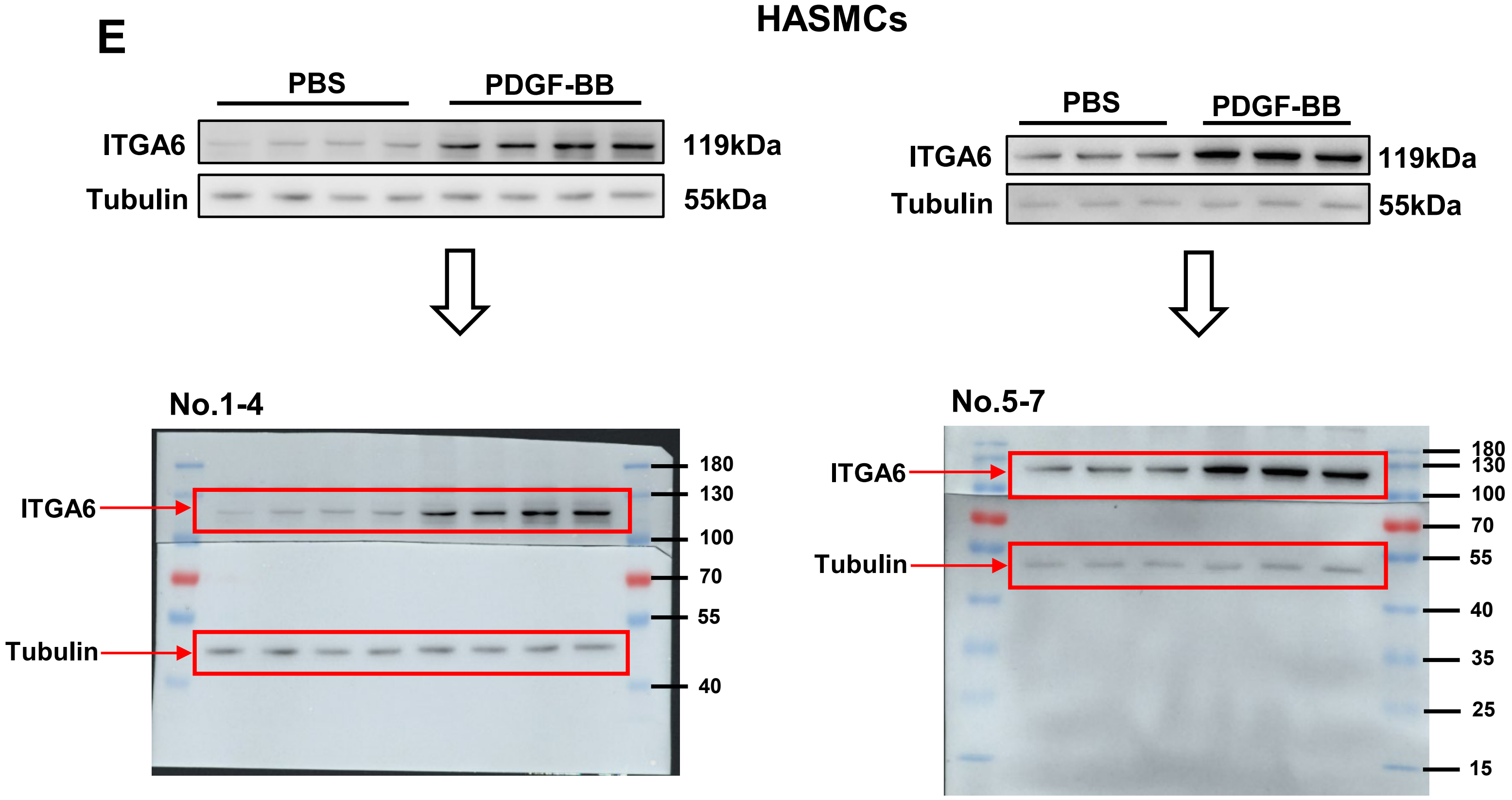

RASMCs

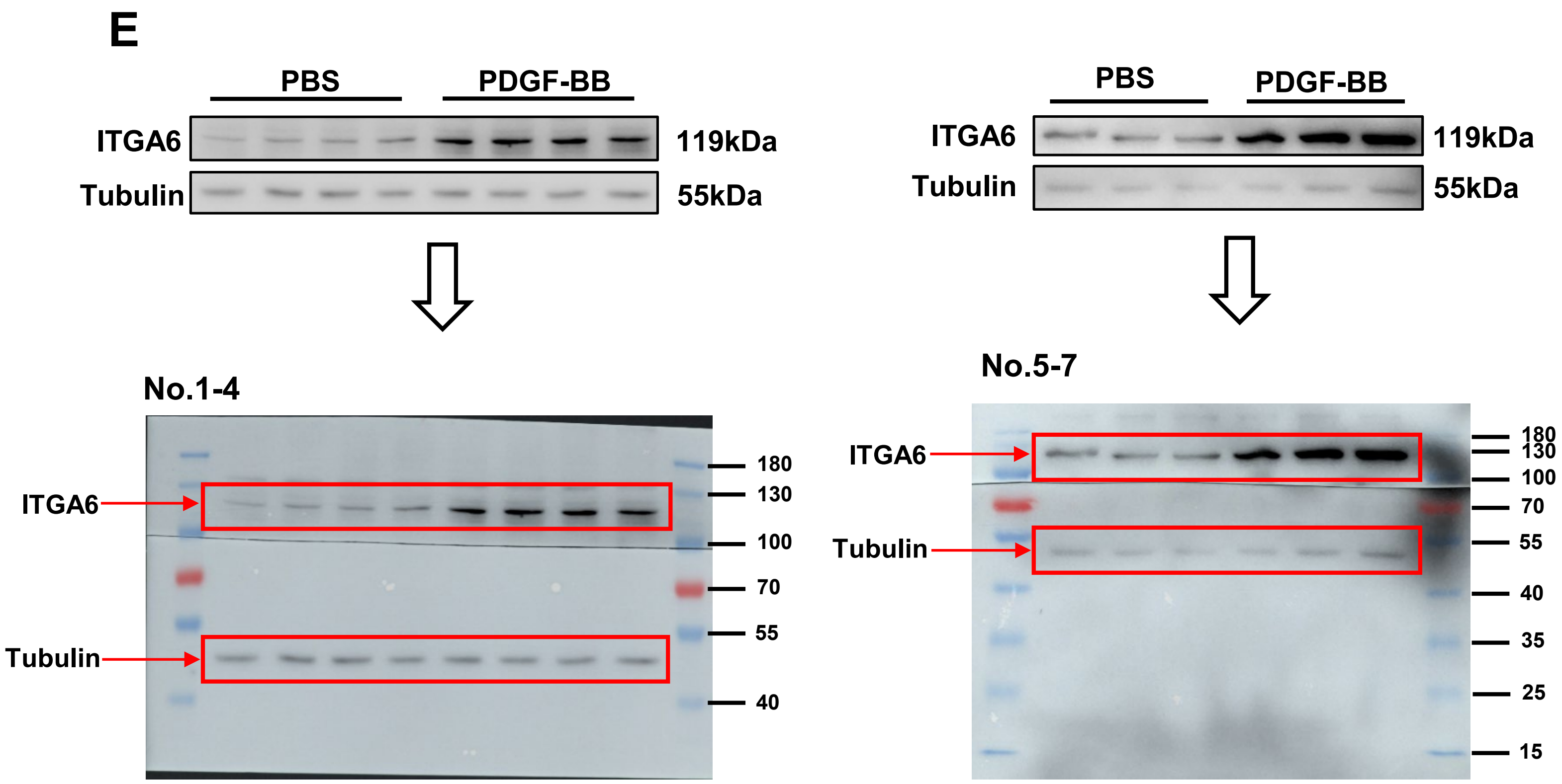

E
PBS
PDGF-BB
ITGA6
Tubulin
119kDa
55kDa
No.1-4
ITGA6
Tubulin
180
130
100
70
55
40
PBS
PDGF-BB
ITGA6
Tubulin
119kDa
55kDa
No.5-7
ITGA6
Tubulin
180
130
100
70
55
40
35
25
15

Full unedited gel for Supplemental Figure 6F n=6

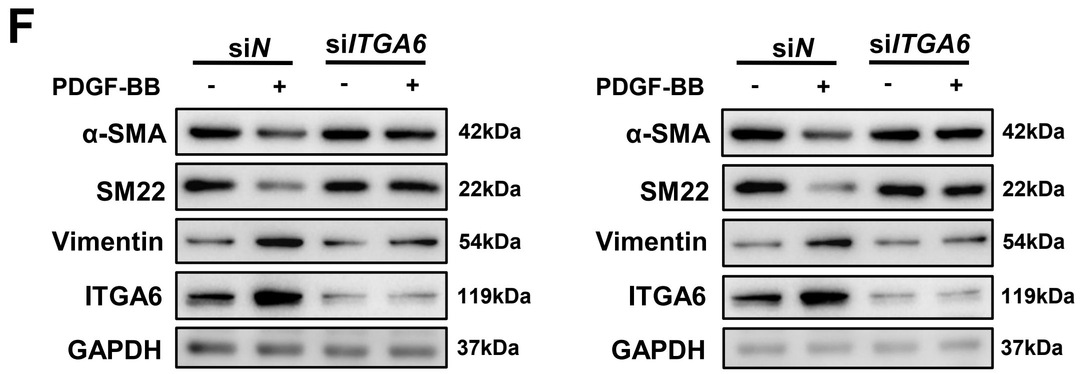

No.1-2

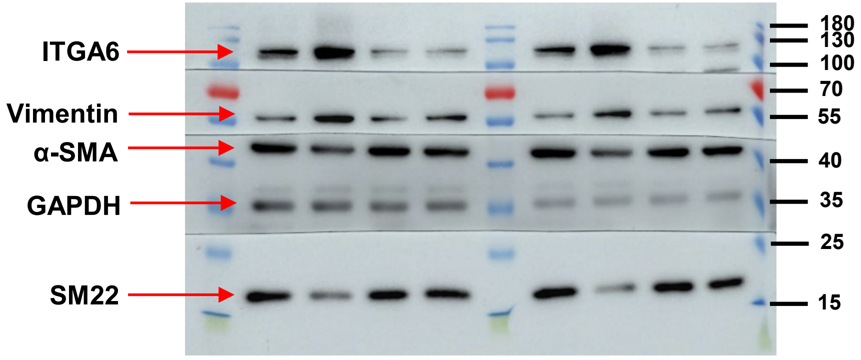

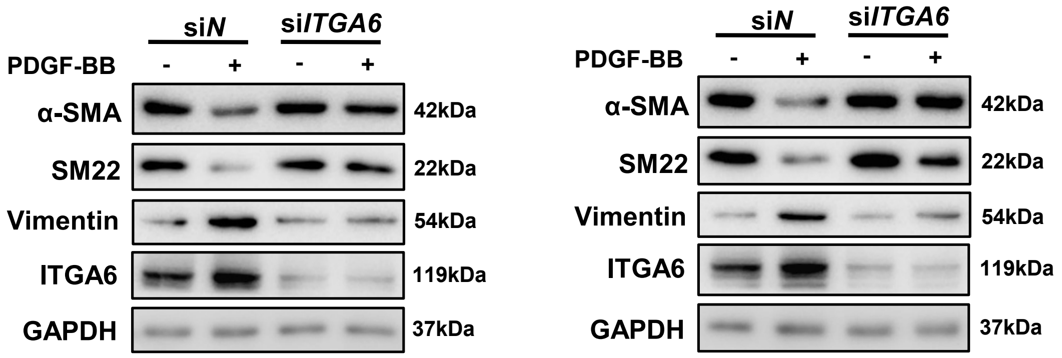

No.3-4

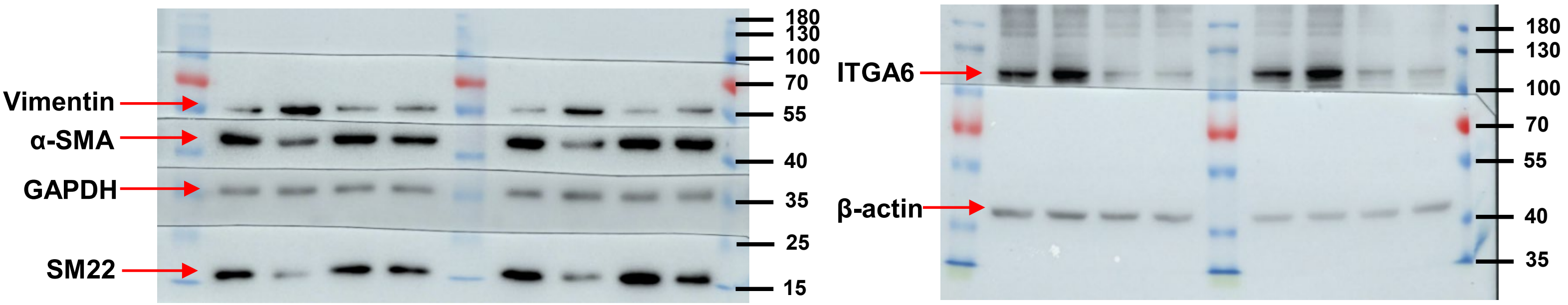

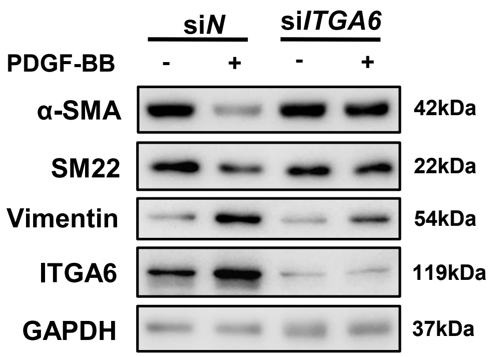

siN
siITGA6
PDGF-BB - + - +
α-SMA 42kDa
SM22 22kDa
Vimentin 54kDa
ITGA6 119kDa
GAPDH 37kDa

No.5

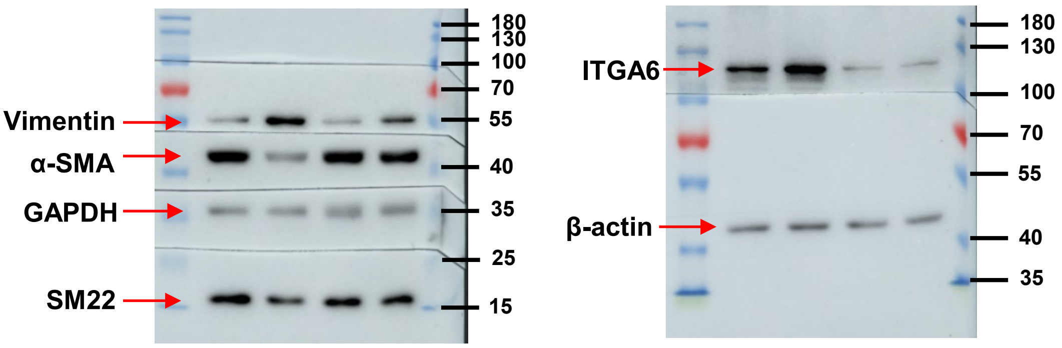

Vimentin
α-SMA
GAPDH
SM22
180
130
100
70
55
40
35
25
15
ITGA6
β-actin
180
130
100
70
55
40
35

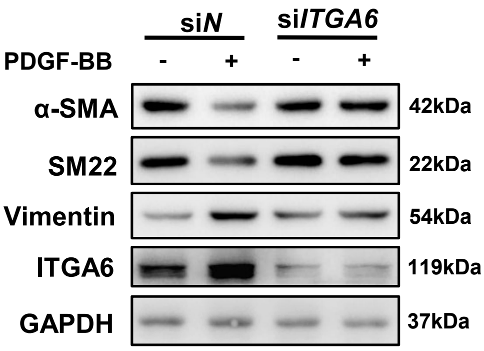

siN
siITGA6
PDGF-BB - + - +
α-SMA 42kDa
SM22 22kDa
Vimentin 54kDa
ITGA6 119kDa
GAPDH 37kDa

No.6

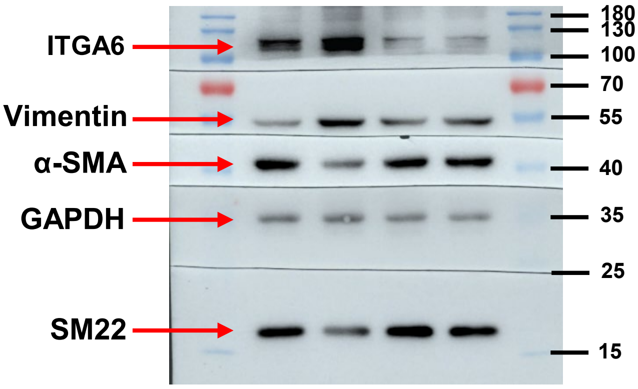

ITGA6
Vimentin
α-SMA
GAPDH
SM22
180
130
100
70
55
40
35
25
15

Full unedited gel for Supplemental Figure 7B n=6

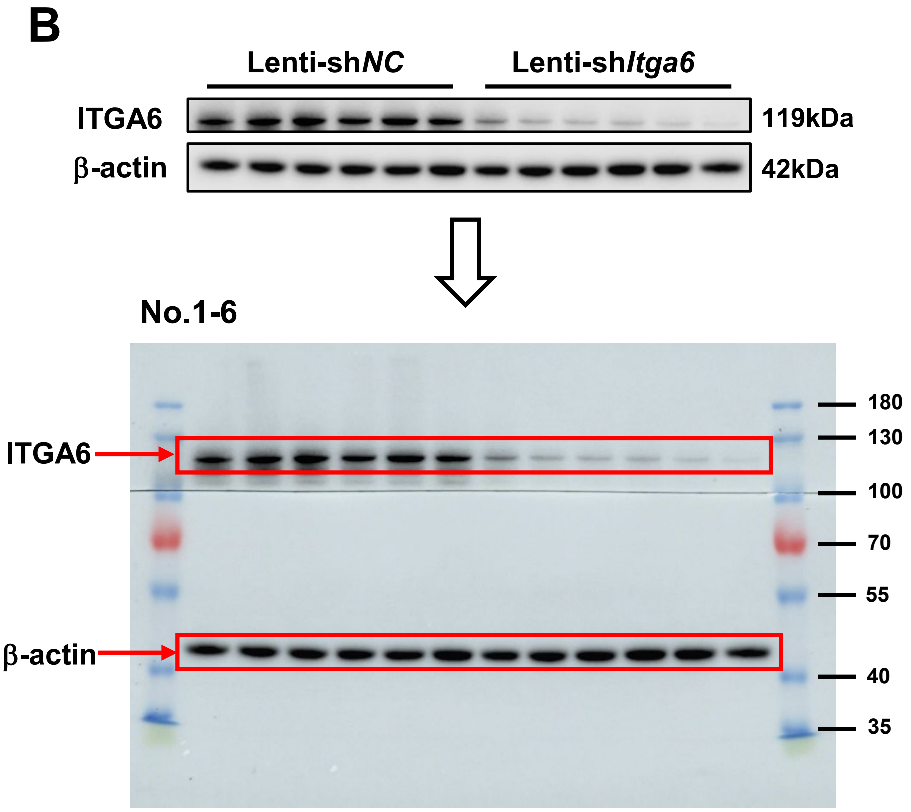

Full unedited gel for Supplemental Figure 8F n=6

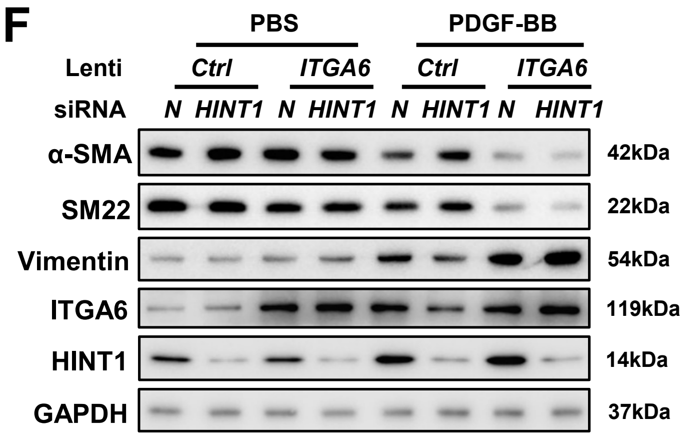

No.1

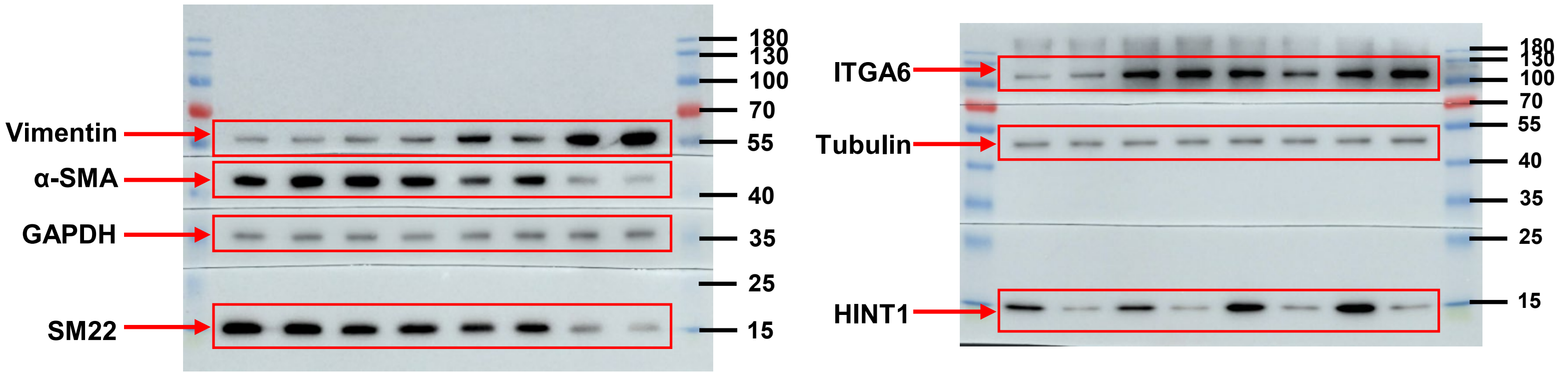

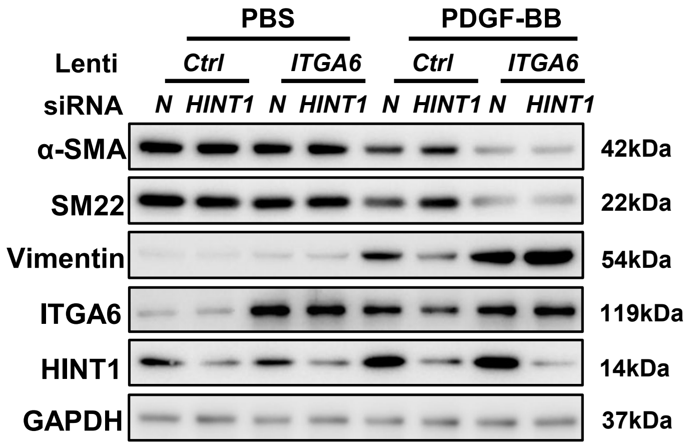

No.2

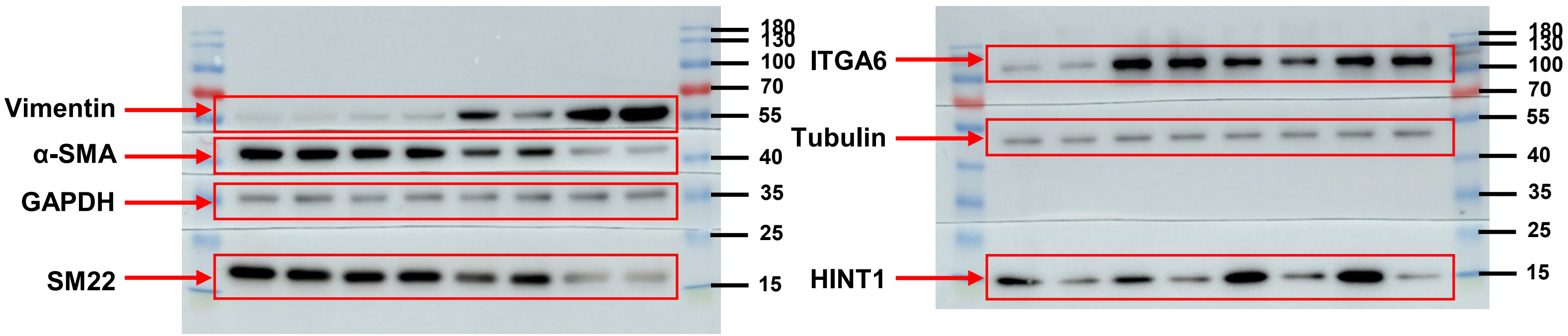

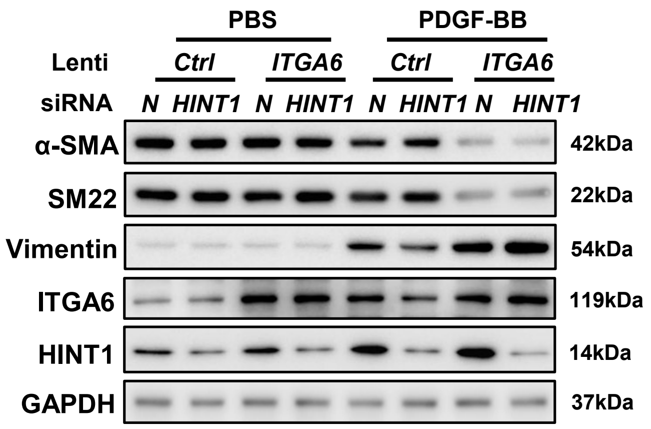
PBS
PDGF-BB
Lenti
Ctrl
ITGA6
Ctrl
ITGA6
siRNA
N HINT1 N HINT1 N HINT1 N HINT1
α-SMA
42kDa
SM22
22kDa
Vimentin
54kDa
ITGA6
119kDa
HINT1
14kDa
GAPDH
37kDa

No.3

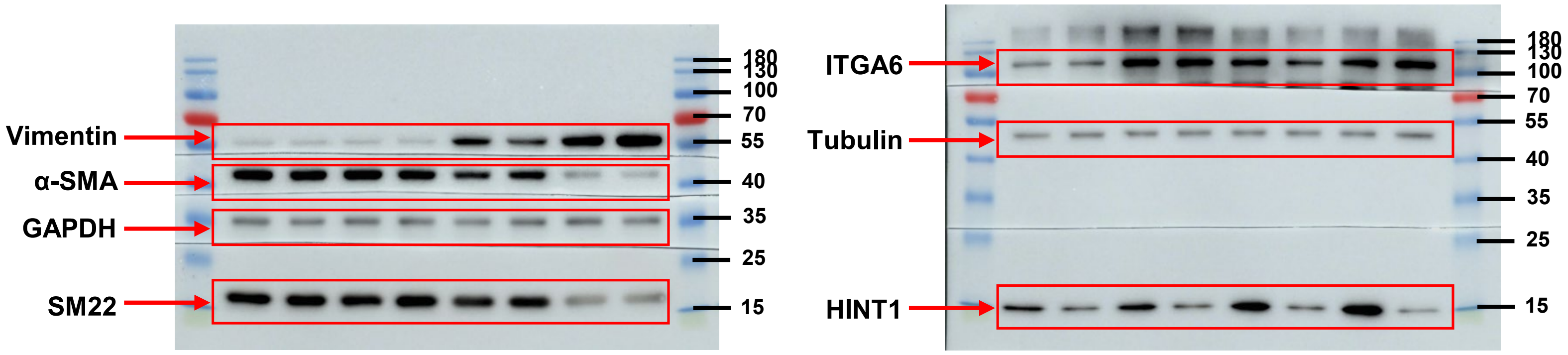
Vimentin
α-SMA
GAPDH
SM22
180
130
100
70
55
40
35
25
15
ITGA6
Tubulin
HINT1
180
130
100
70
55
40
35
25
15

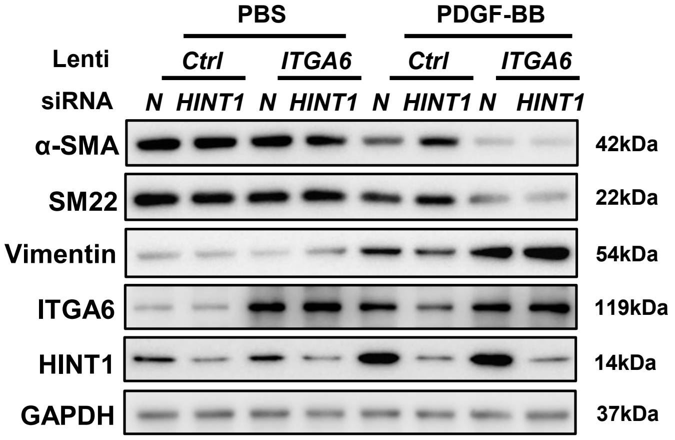
PBS
PDGF-BB
Lenti
Ctrl
ITGA6
Ctrl
ITGA6
siRNA
N HINT1 N HINT1 N HINT1 N HINT1
α-SMA
42kDa
SM22
22kDa
Vimentin
54kDa
ITGA6
119kDa
HINT1
14kDa
GAPDH
37kDa

No.4

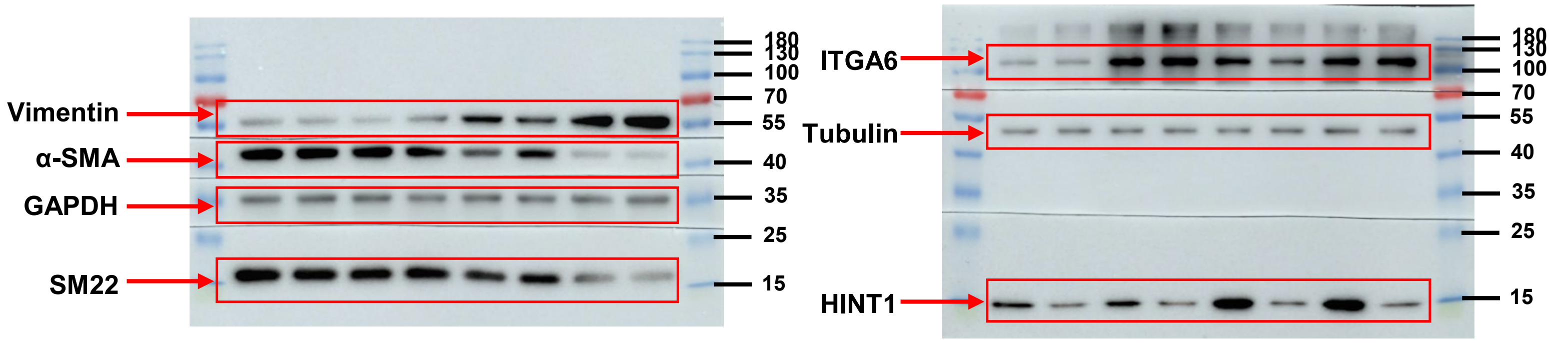
Vimentin
α-SMA
GAPDH
SM22
180
130
100
70
55
40
35
25
15
ITGA6
Tubulin
HINT1
180
130
100
70
55
40
35
25
15

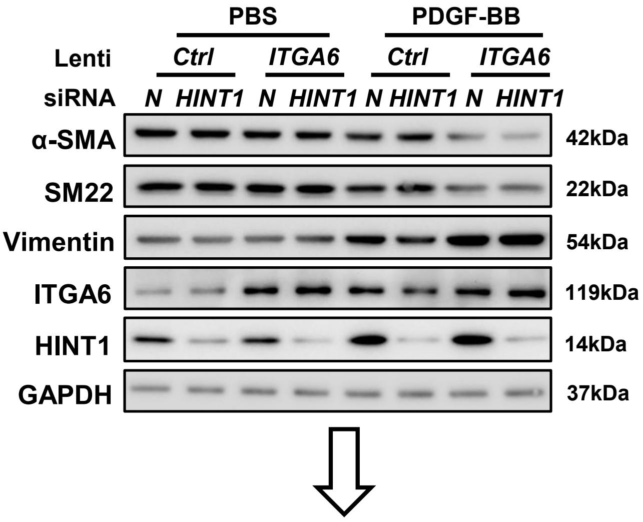
PBS
PDGF-BB
Lenti
Ctrl
ITGA6
Ctrl
ITGA6
siRNA
N HINT1 N HINT1 N HINT1 N HINT1
α-SMA 42kDa
SM22 22kDa
Vimentin 54kDa
ITGA6 119kDa
HINT1 14kDa
GAPDH 37kDa

No.5

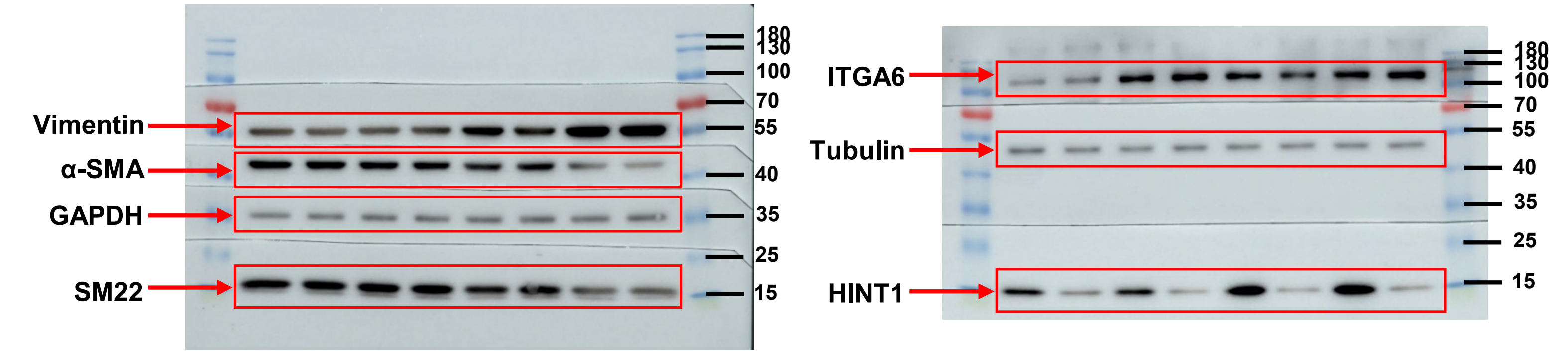
Vimentin
α-SMA
GAPDH
SM22
180
130
100
70
55
40
35
25
15
ITGA6
Tubulin
HINT1

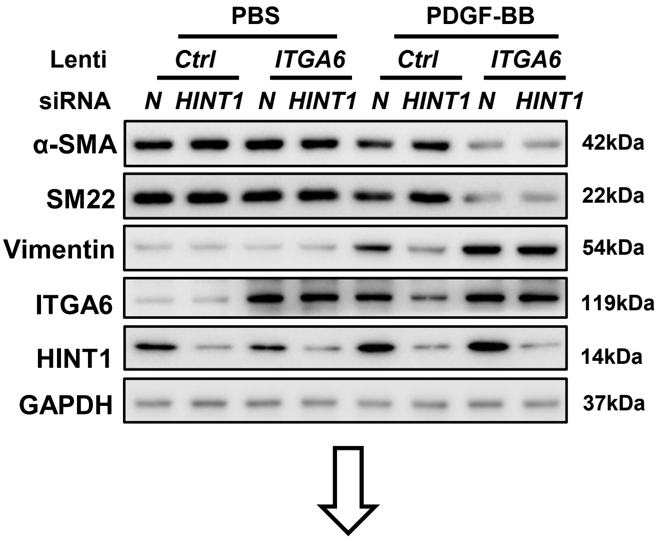
PBS
PDGF-BB
Lenti
Ctrl
ITGA6
Ctrl
ITGA6
siRNA
N HINT1 N HINT1 N HINT1 N HINT1
α-SMA 42kDa
SM22 22kDa
Vimentin 54kDa
ITGA6 119kDa
HINT1 14kDa
GAPDH 37kDa

No.6

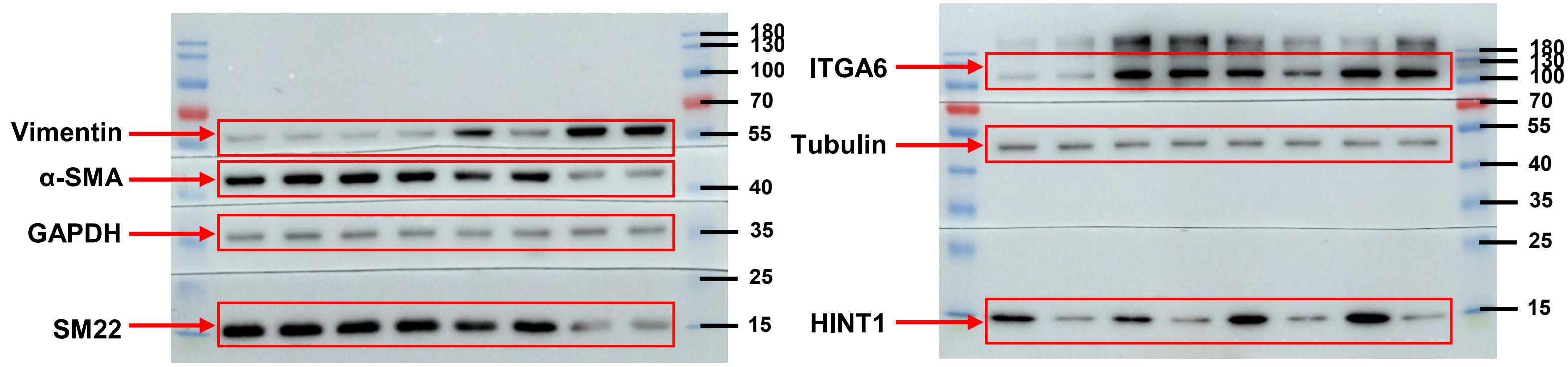
Vimentin
α-SMA
GAPDH
SM22
180
130
100
70
55
40
35
25
15
ITGA6
Tubulin
HINT1

Full unedited gel for Supplemental Figure 9B n=6

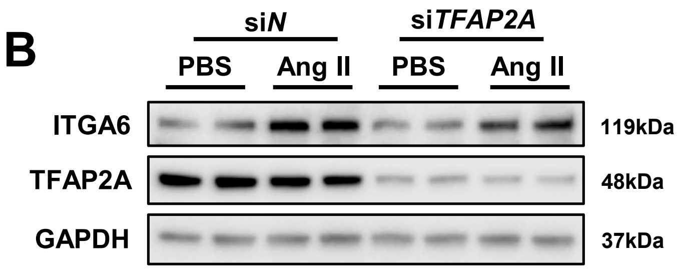

No.1-2

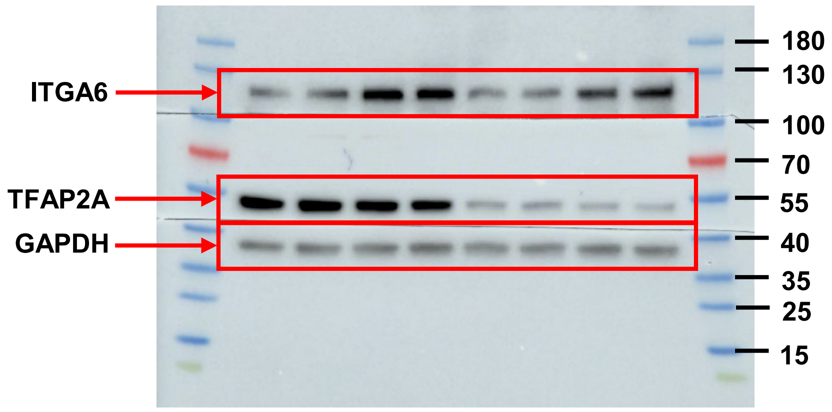

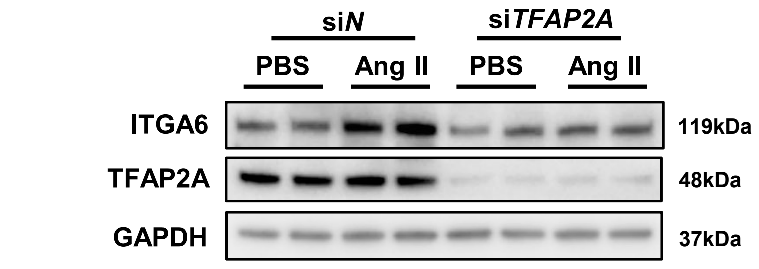

No.3-4

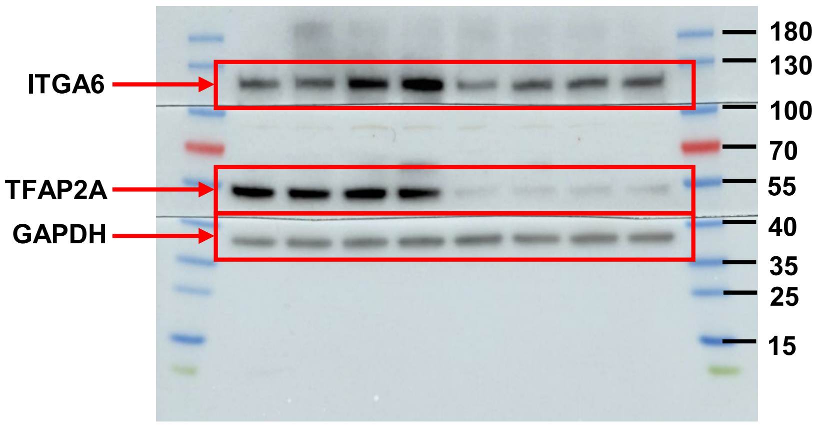

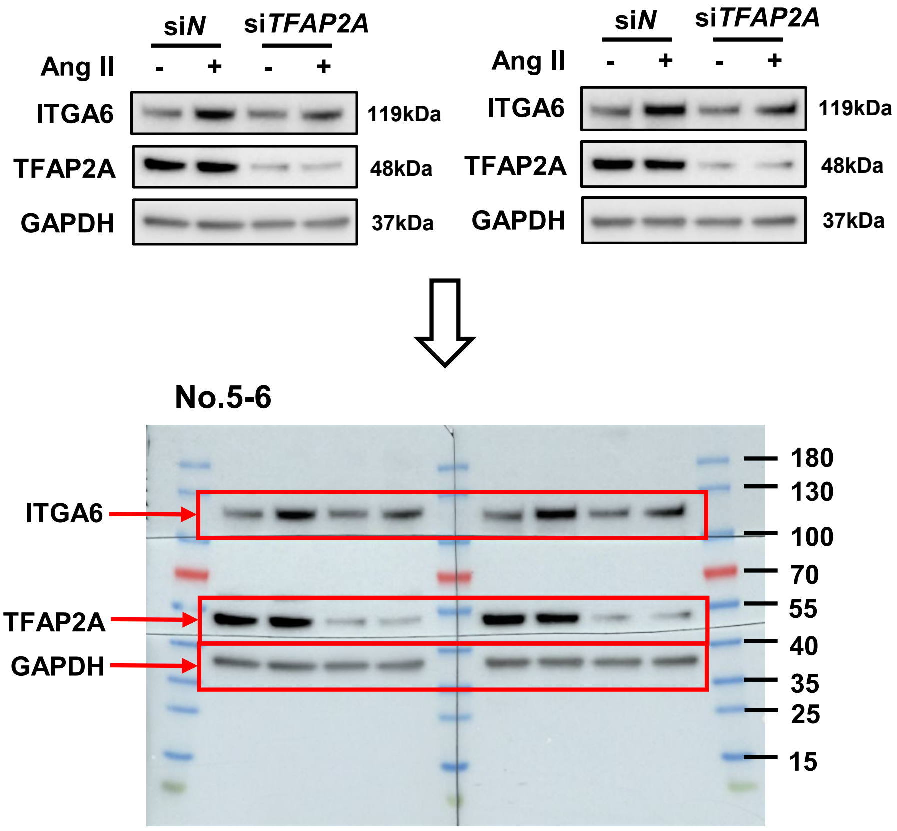
siN
siTFAP2A
Ang II
- + - +
ITGA6
119kDa
TFAP2A
48kDa
GAPDH
37kDa
No.5-6
180
130
100
70
55
40
35
25
15

Full unedited gel for Supplemental Figure 9F n=1

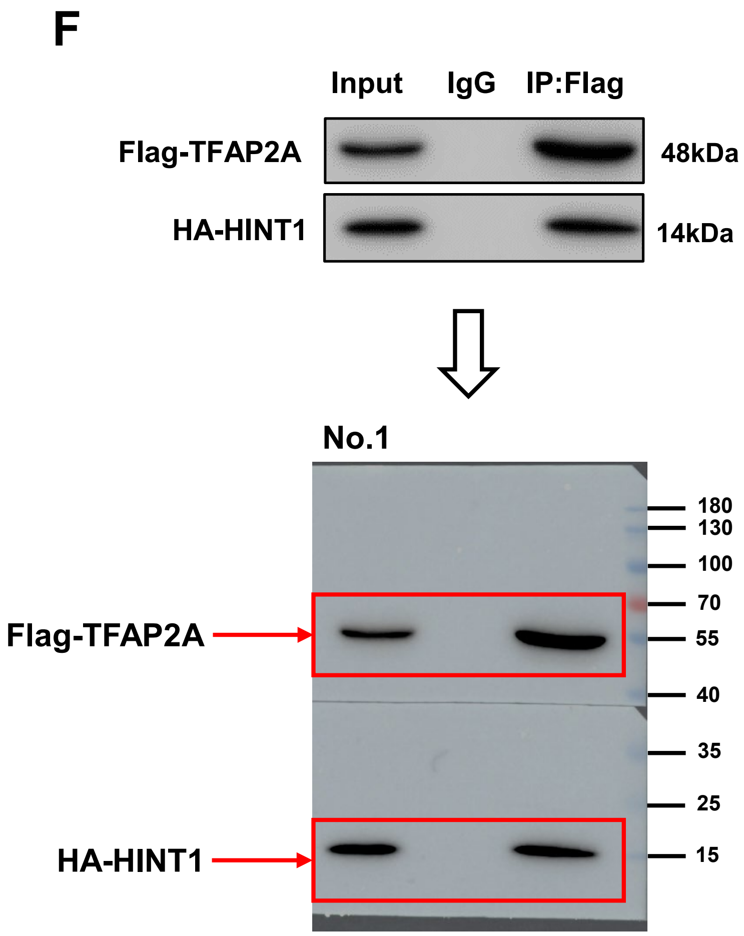

Full unedited gel for Supplemental Figure 9G n=1

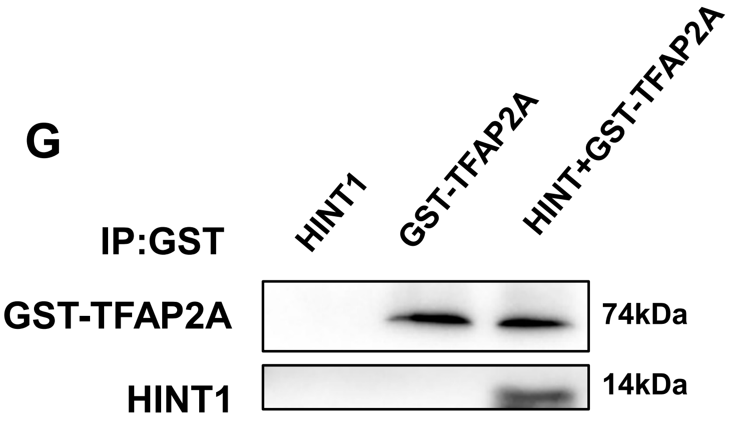

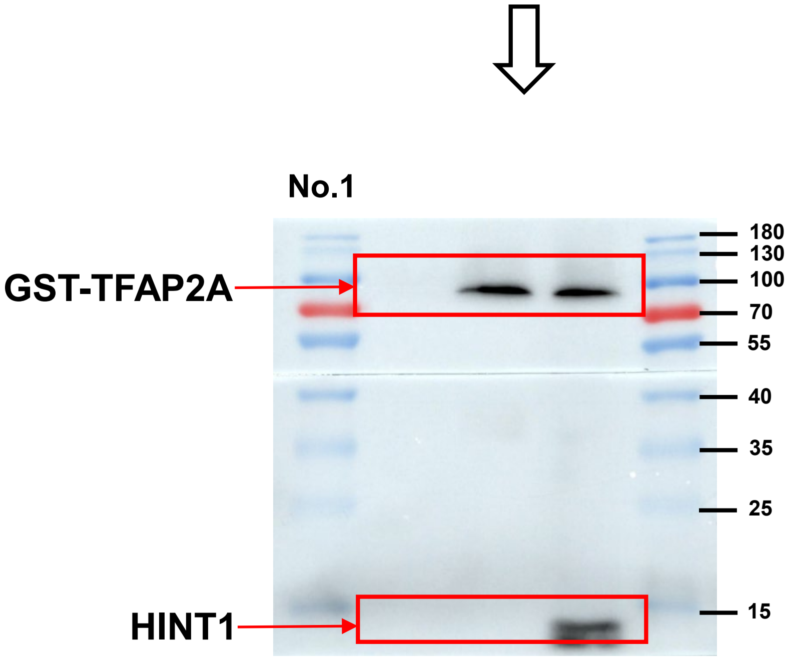

Full unedited gel for Supplemental Figure 9H n=1

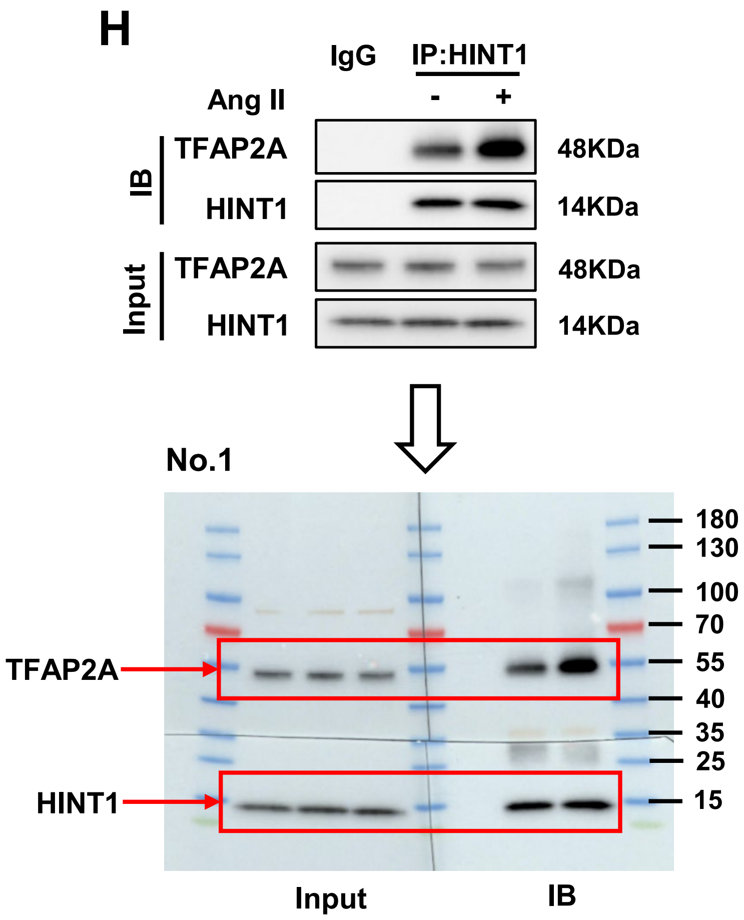

Full unedited gel for Supplemental Figure 10A n=6

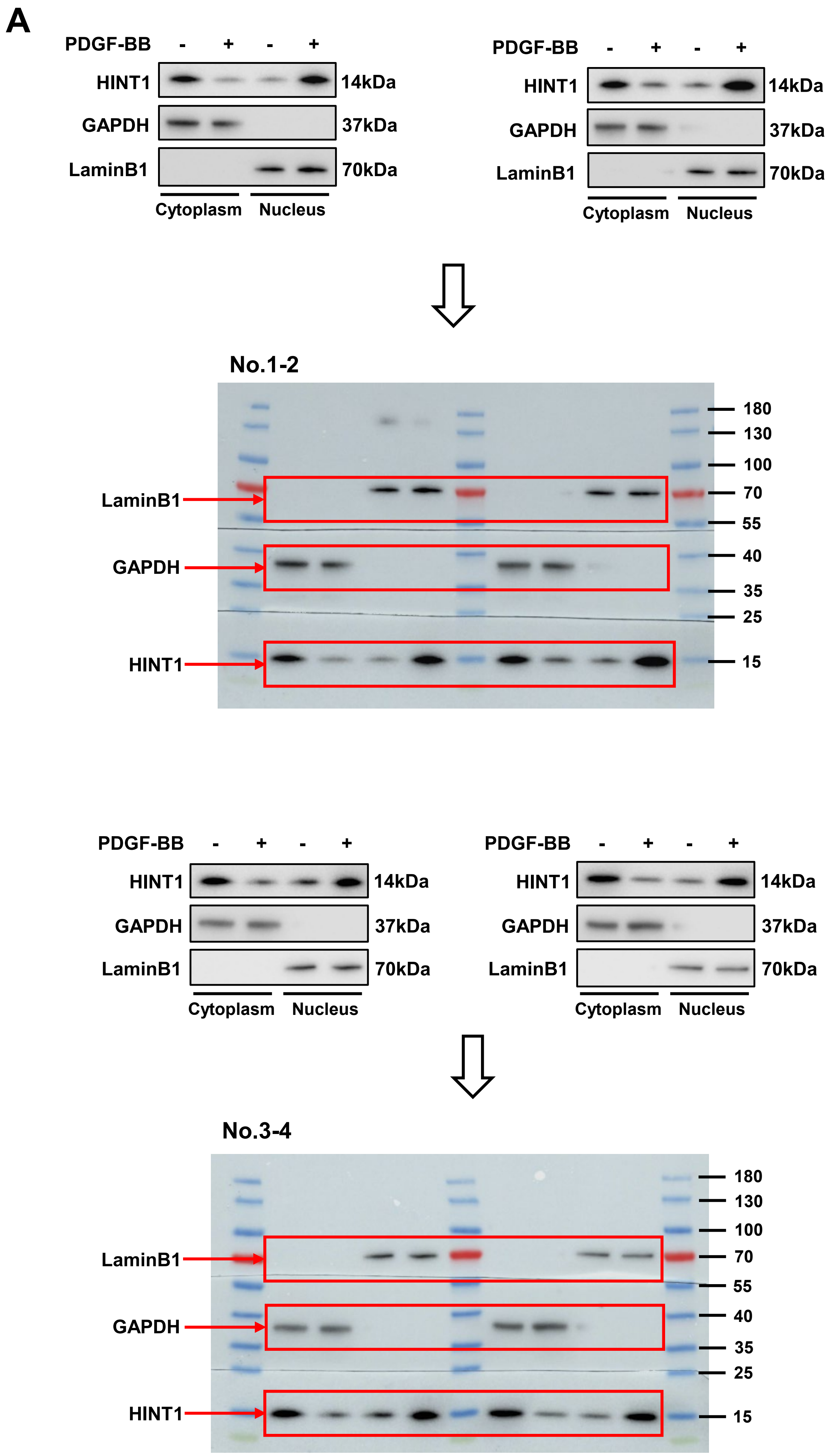

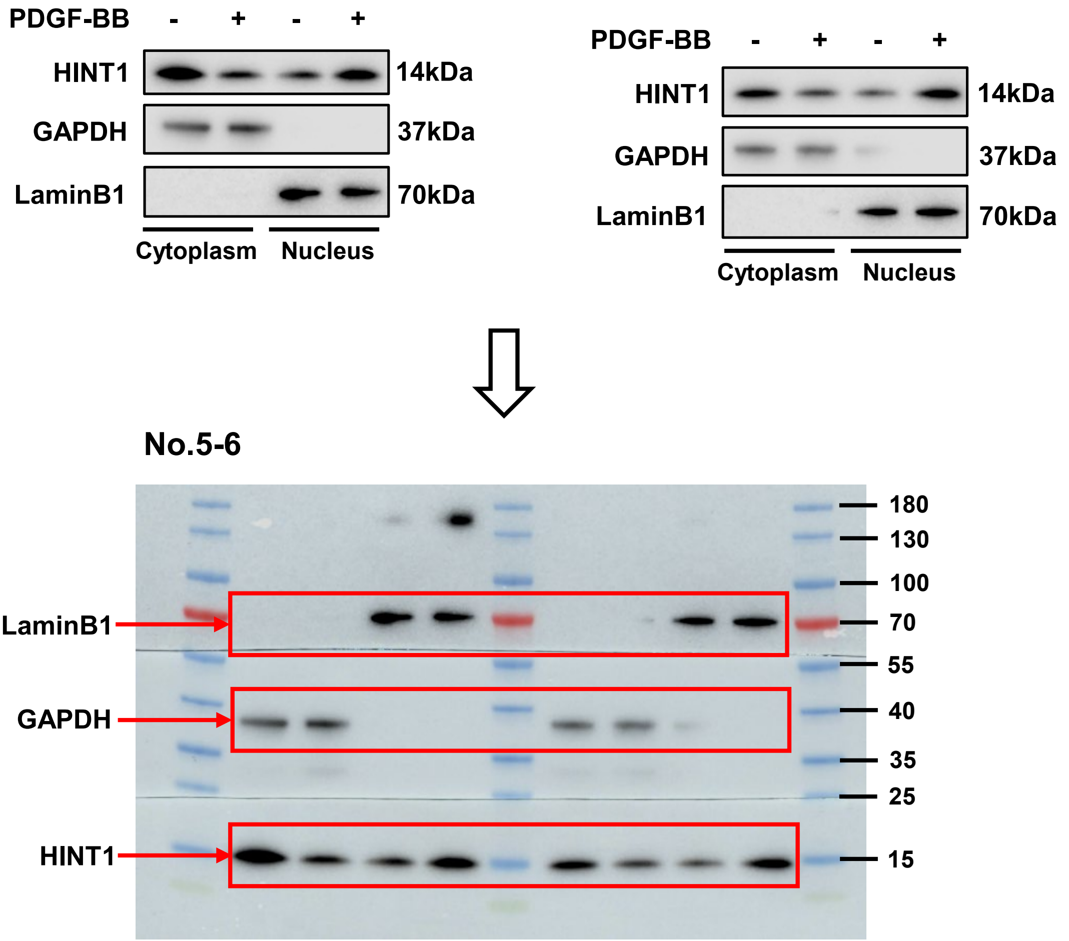
PDGF-BB - + - +
HINT1
14kDa
GAPDH
37kDa
LaminB1
70kDa
Cytoplasm
Nucleus
No.5-6
LaminB1
GAPDH
HINT1
180
130
100
70
55
40
35
25
15

Full unedited gel for Supplemental Figure 10D n=1

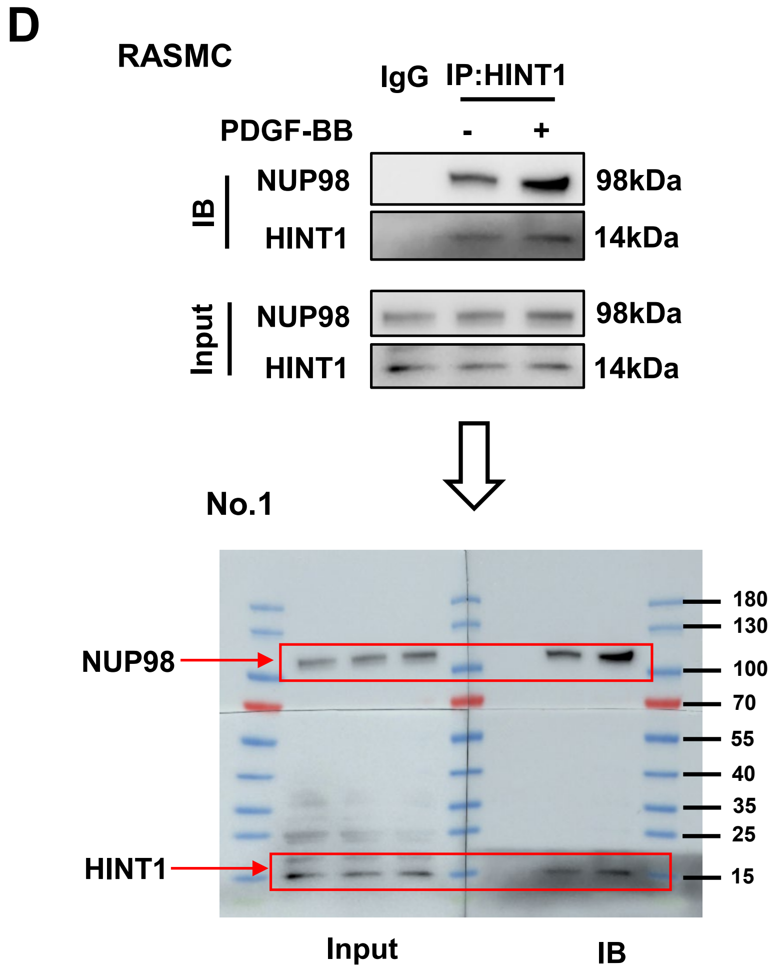

Full unedited gel for Supplemental Figure 10E n=1

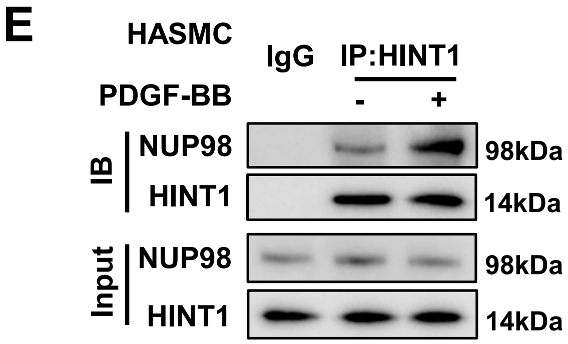

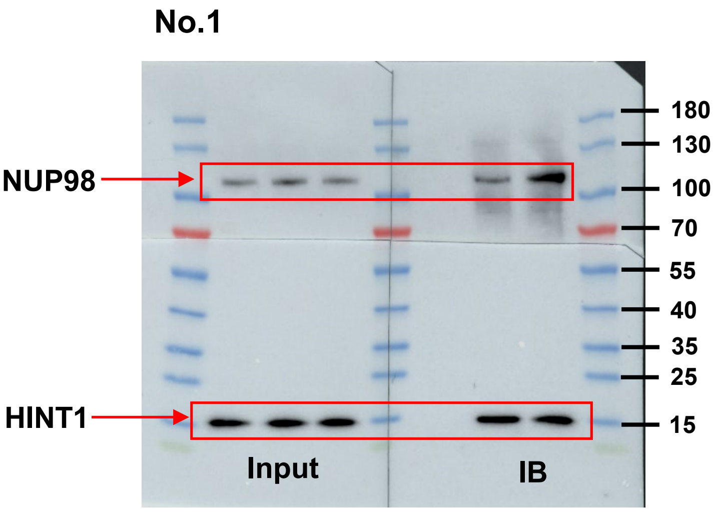

Full unedited gel for Supplemental Figure 10F n=6

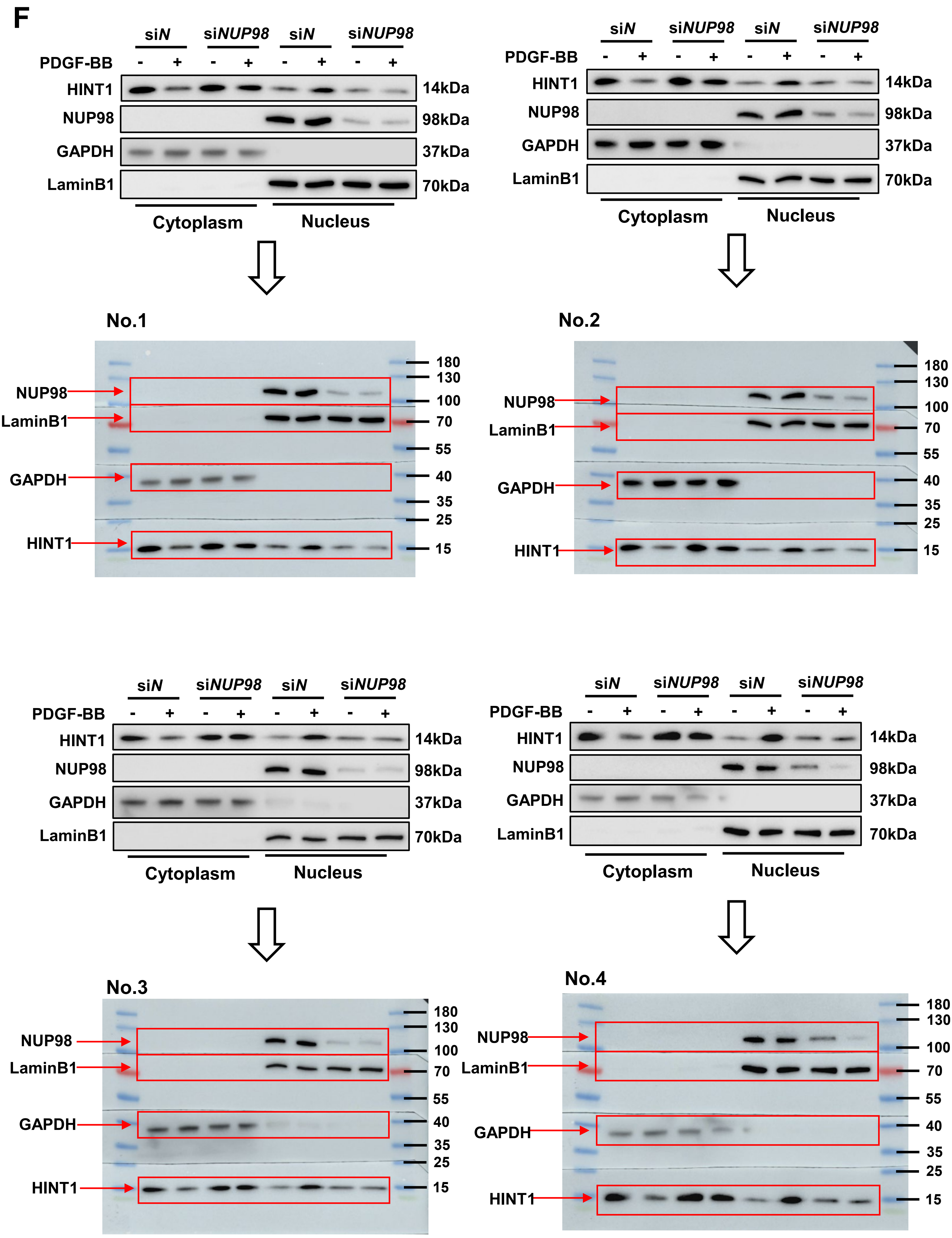

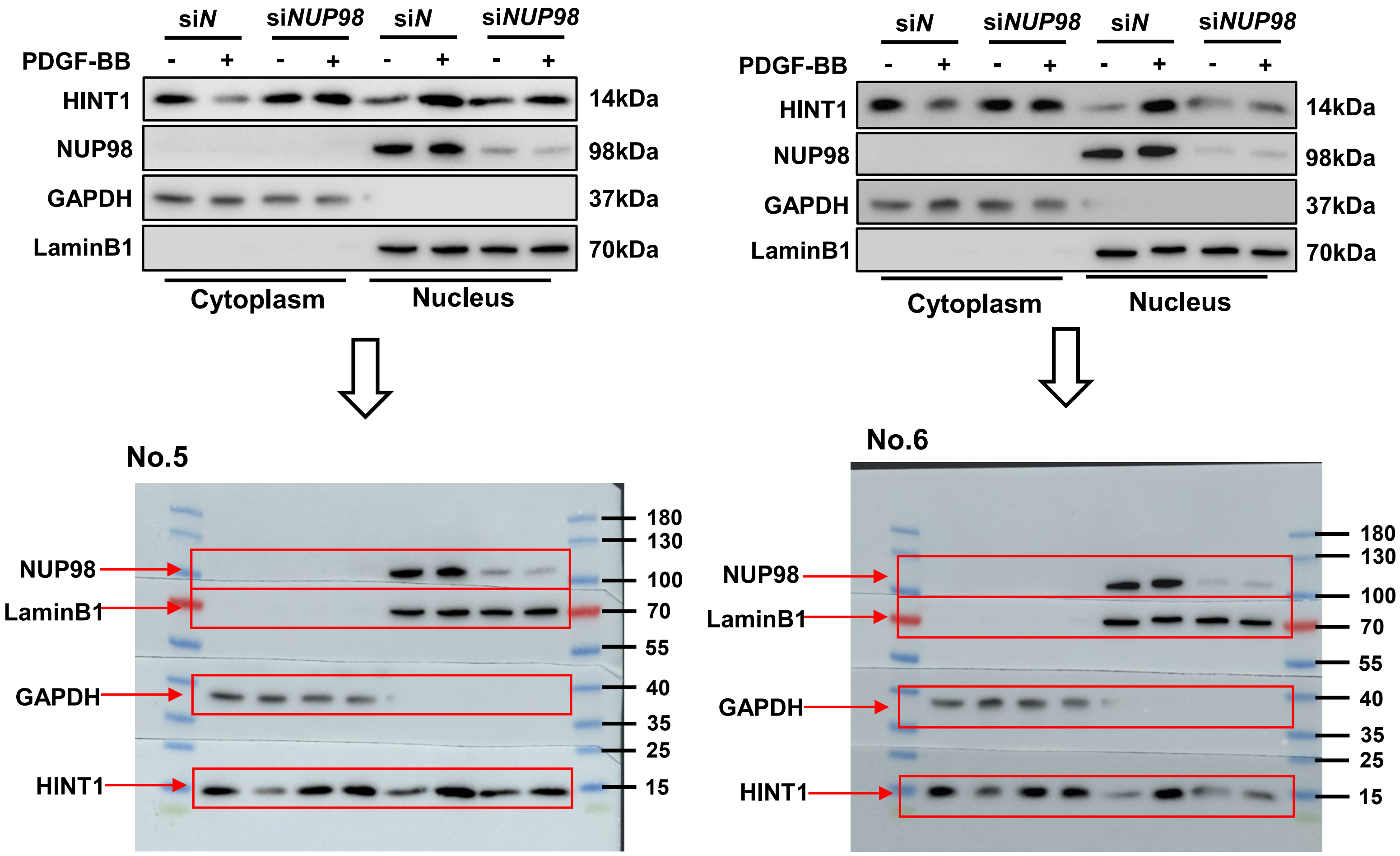

siN
siNUP98
siN
siNUP98
PDGF-BB
-
+
HINT1
NUP98
GAPDH
LaminB1
14kDa
98kDa
37kDa
70kDa
Cytoplasm
Nucleus
No.5
No.6
180
130
100
70
55
40
35
25
15

Full unedited gel for Supplemental Figure 10G n=3

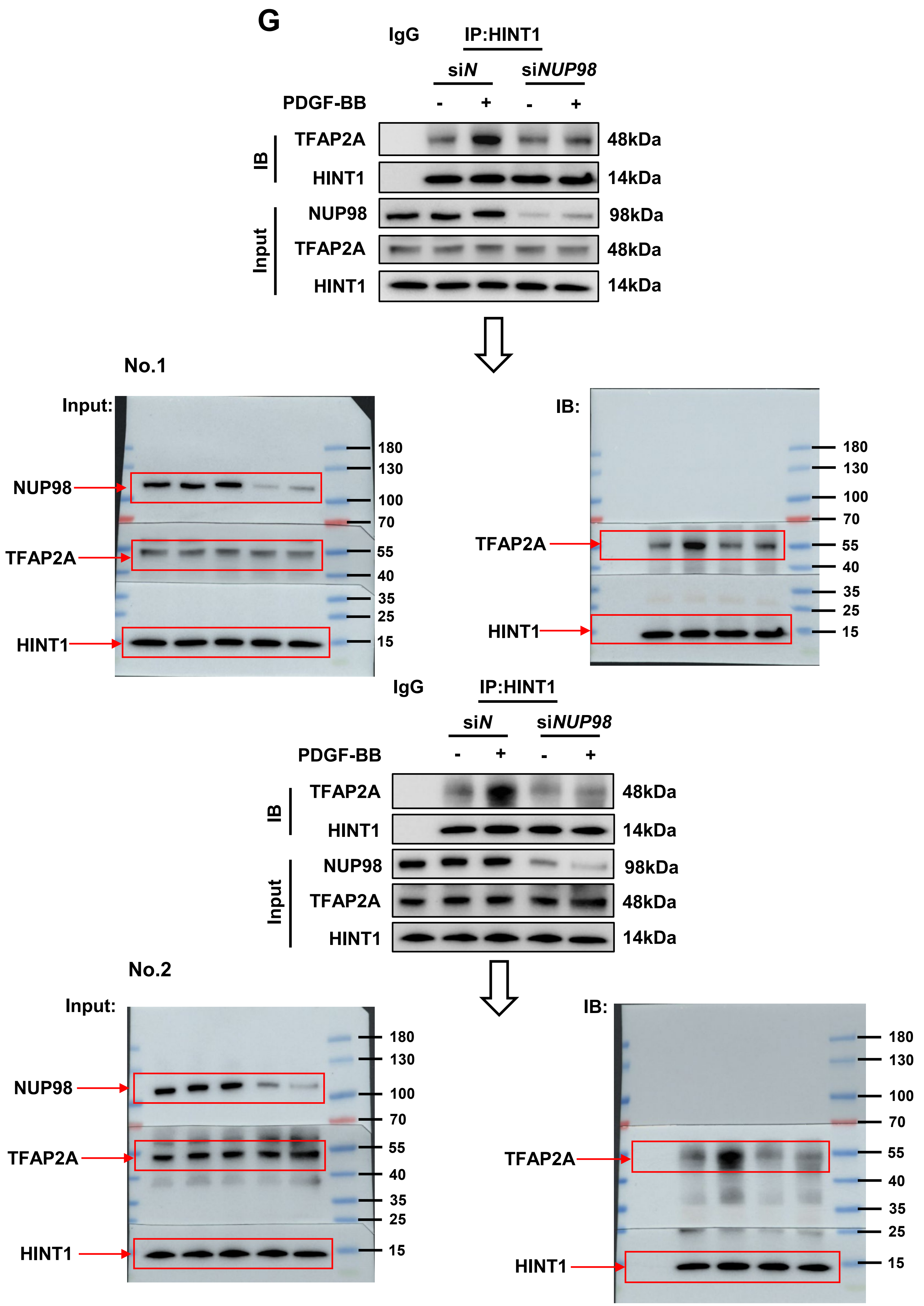

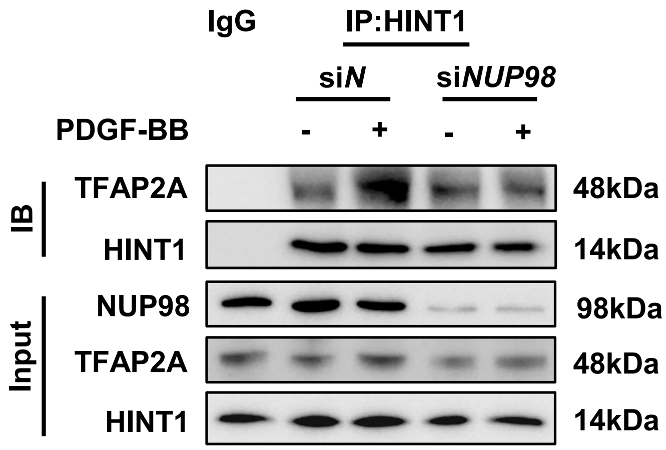
IgG
IP:HINT1
siN
siNUP98
PDGF-BB
-
+
-
+
IB
TFAP2A
HINT1
Input
NUP98
TFAP2A
HINT1
48kDa
14kDa
98kDa
48kDa
14kDa

No.3

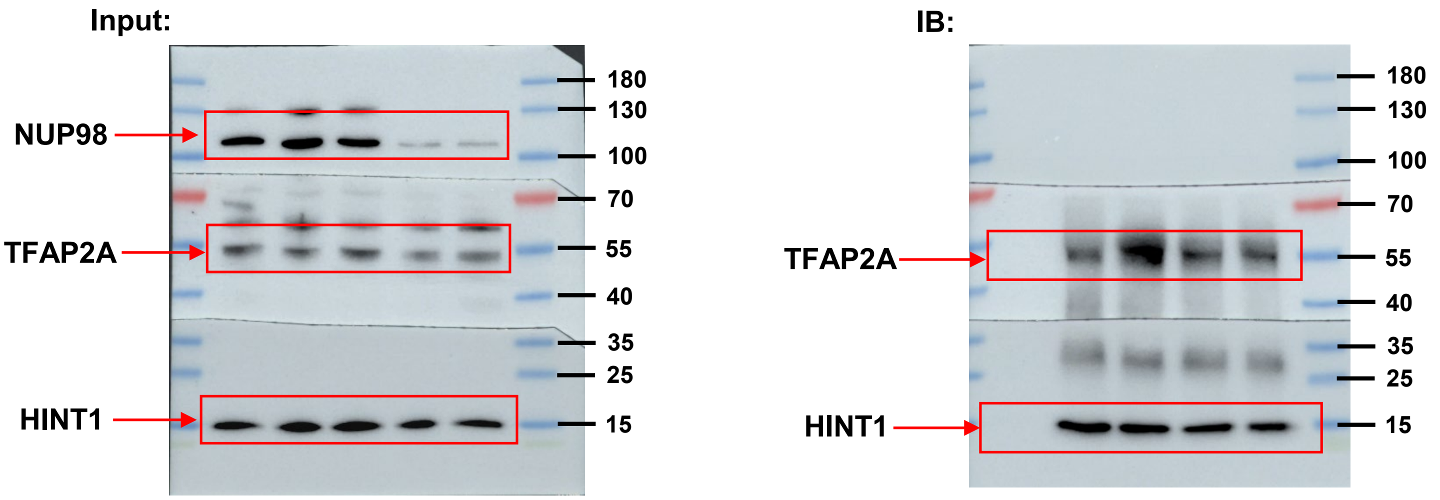
Input:
NUP98
TFAP2A
HINT1
180
130
100
70
55
40
35
25
15
IB:
TFAP2A
HINT1
180
130
100
70
55
40
35
25
15

Full unedited gel for Supplemental Figure 10I n=6

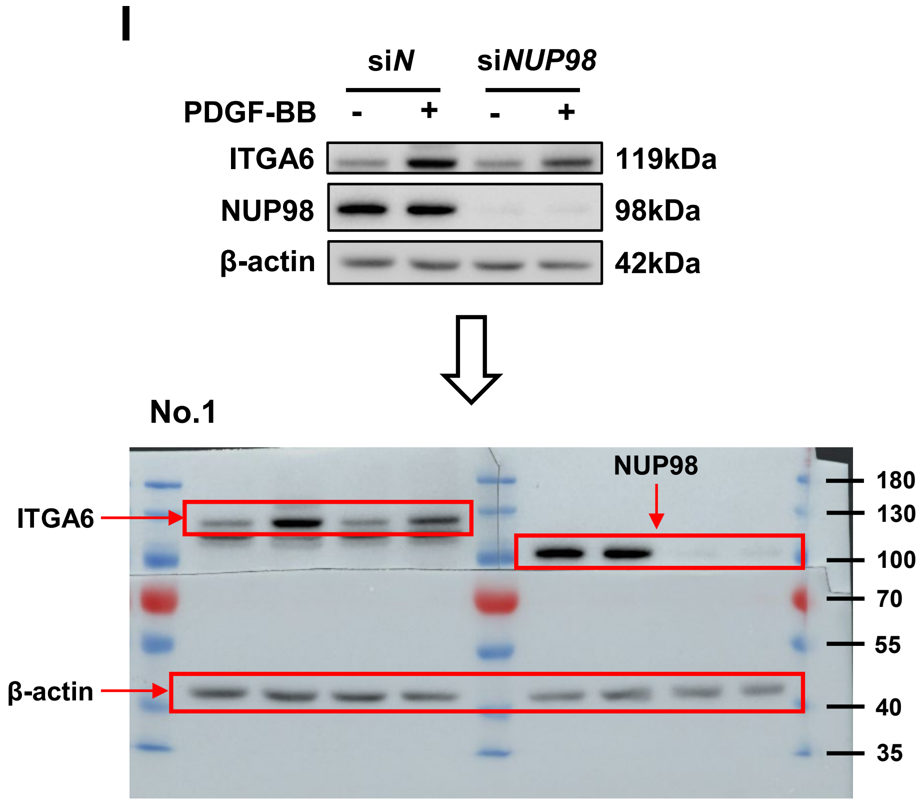

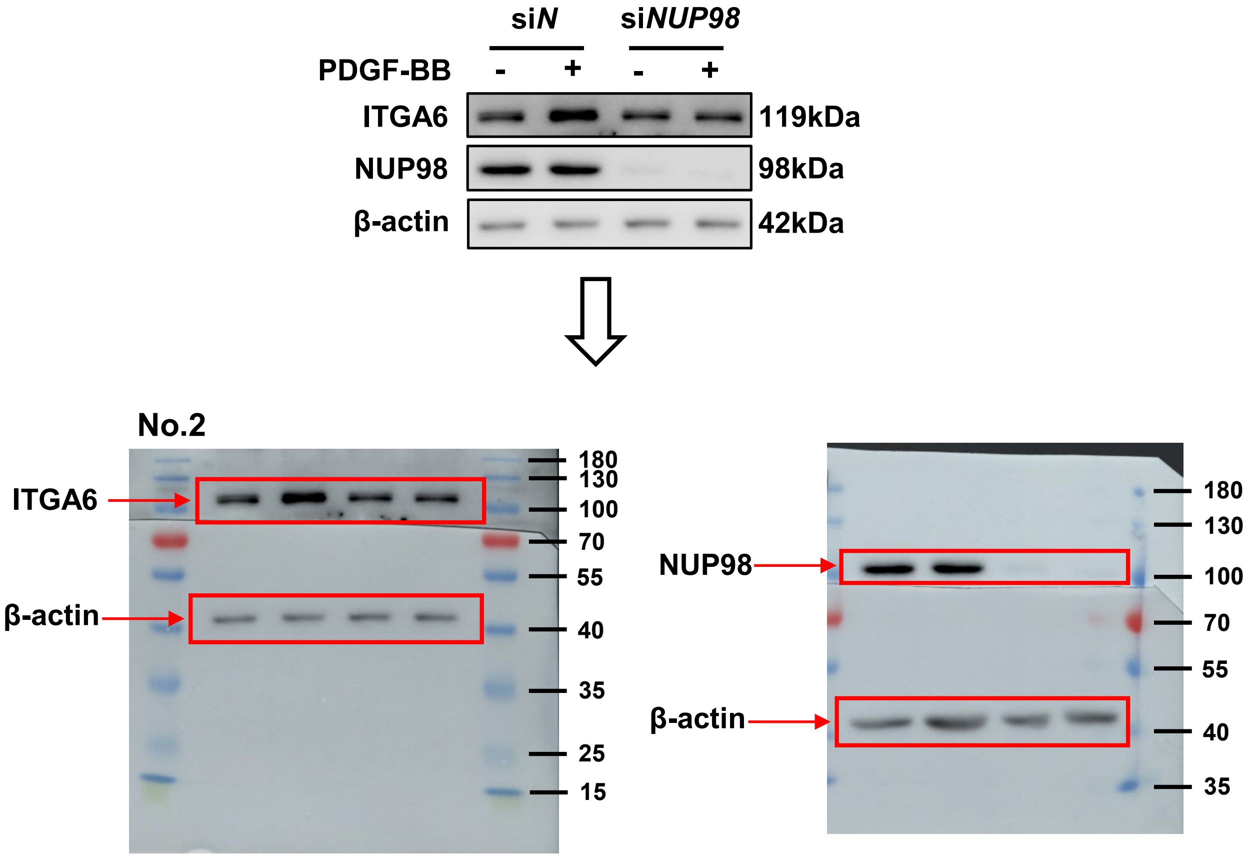

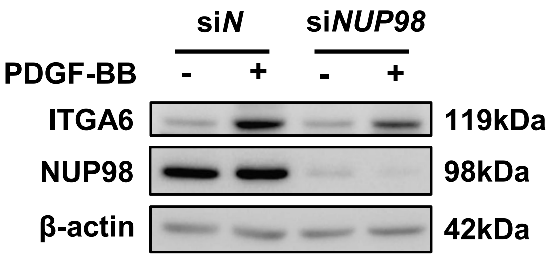
siN
siNUP98
PDGF-BB - + - +
ITGA6 119kDa
NUP98 98kDa
β-actin 42kDa

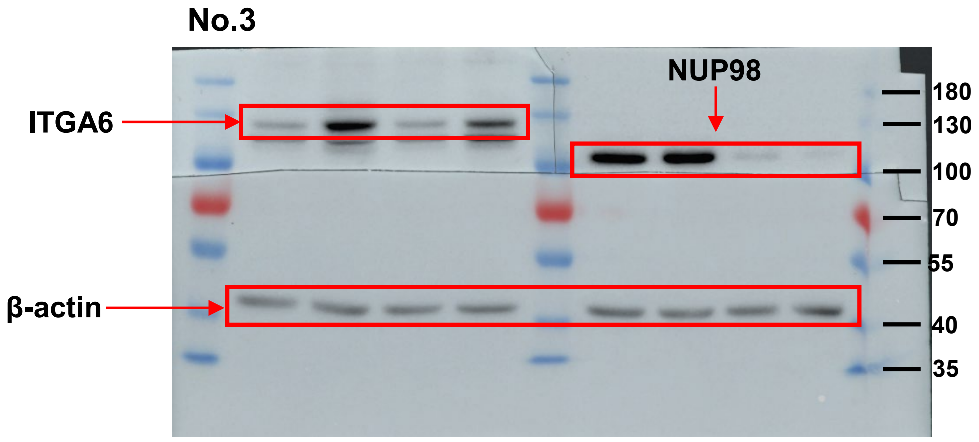
No.3
ITGA6
β-actin
NUP98
180
130
100
70
55
40
35

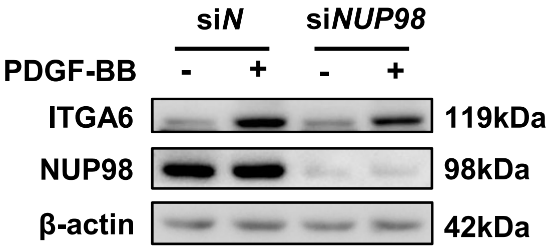
siN
siNUP98
PDGF-BB - + - +
ITGA6 119kDa
NUP98 98kDa
β-actin 42kDa

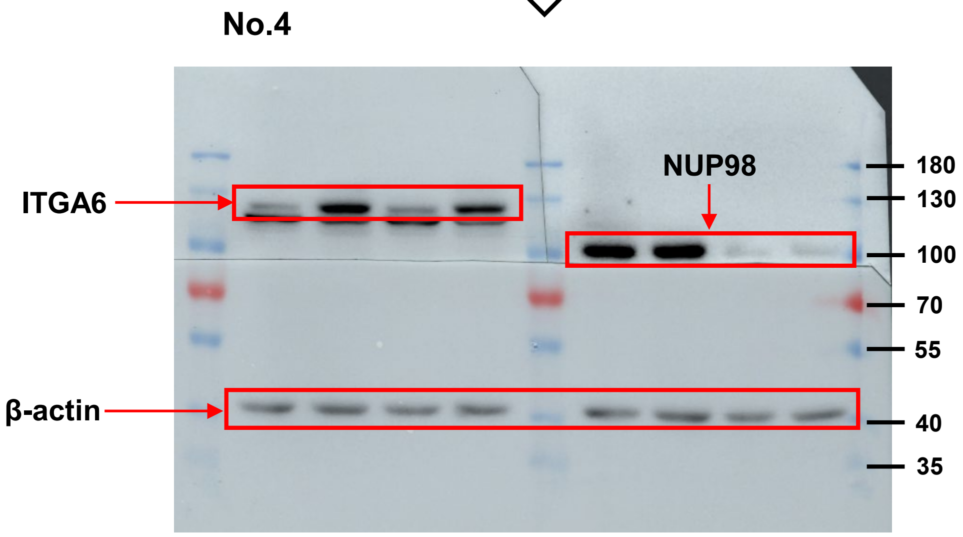
No.4
ITGA6
β-actin
NUP98
180
130
100
70
55
40
35

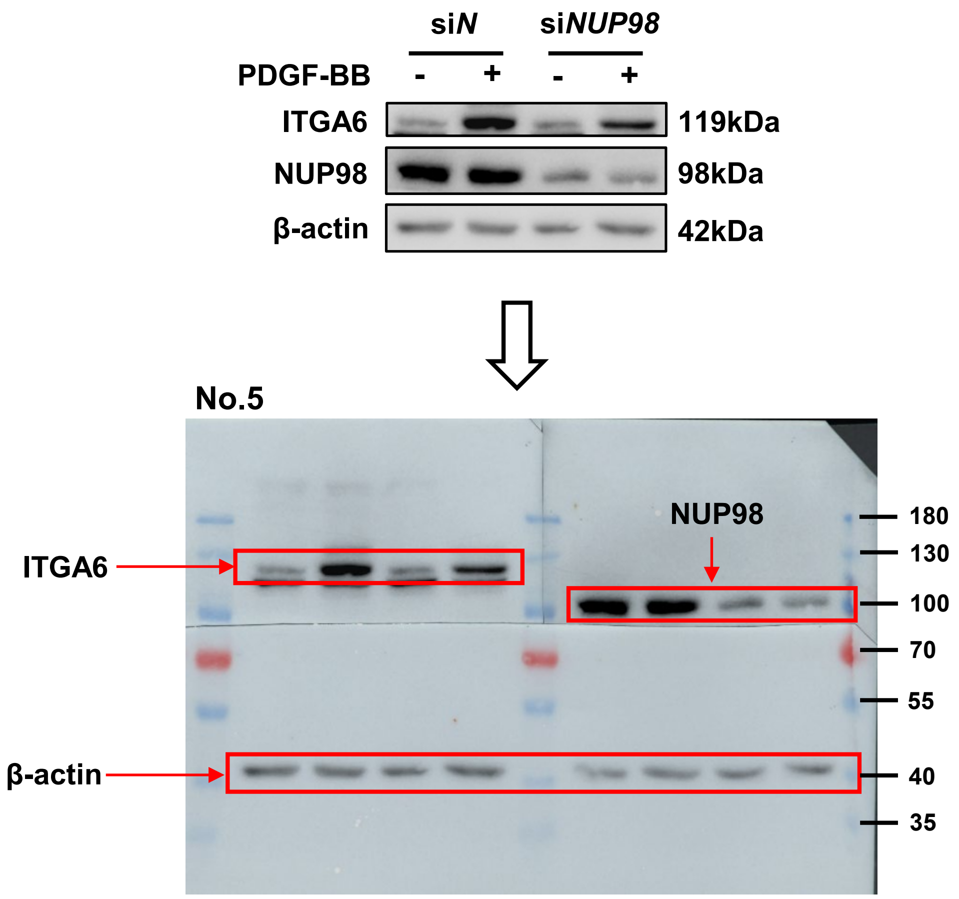
siN
siNUP98
PDGF-BB
- + - +
ITGA6
119kDa
NUP98
98kDa
β-actin
42kDa
No.5
ITGA6
NUP98
β-actin
180
130
100
70
55
40
35

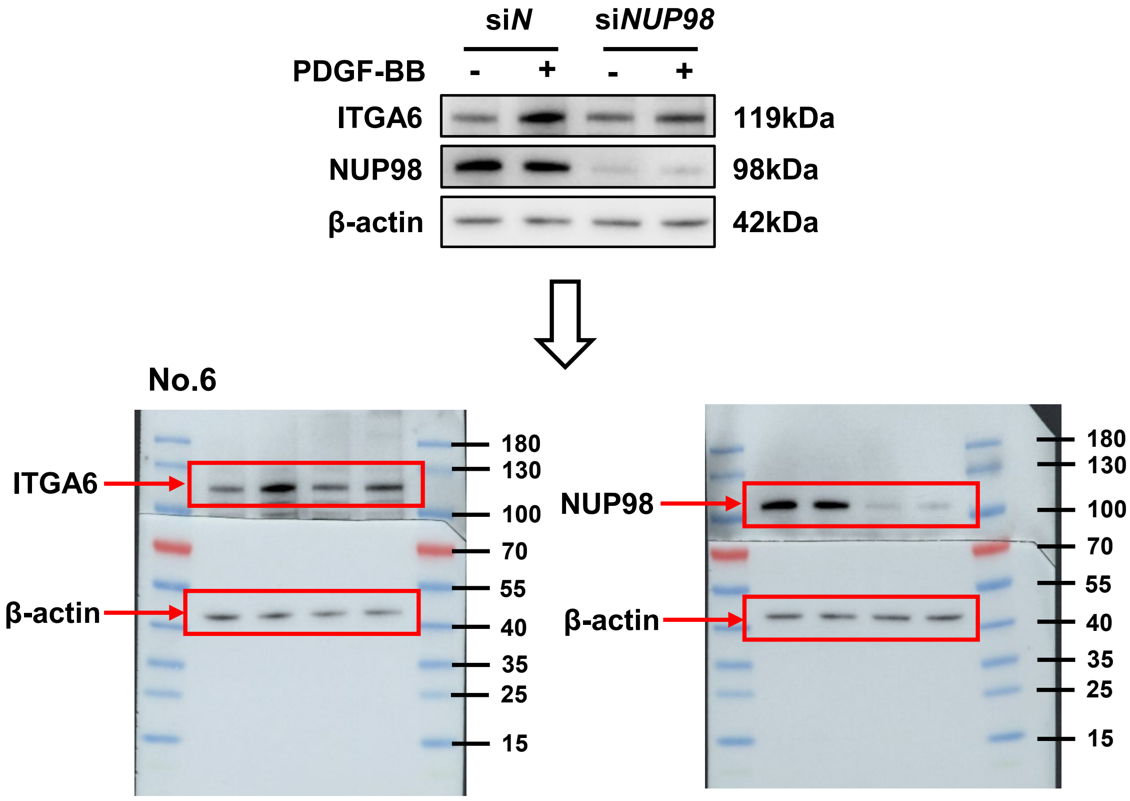
siN
siNUP98
PDGF-BB
- + - +
ITGA6
119kDa
NUP98
98kDa
β-actin
42kDa
No.6
ITGA6
β-actin
180
130
100
70
55
40
35
25
15
NUP98
β-actin
180
130
100
70
55
40
35
25
15

Full unedited gel for Supplemental Figure 10J n=5

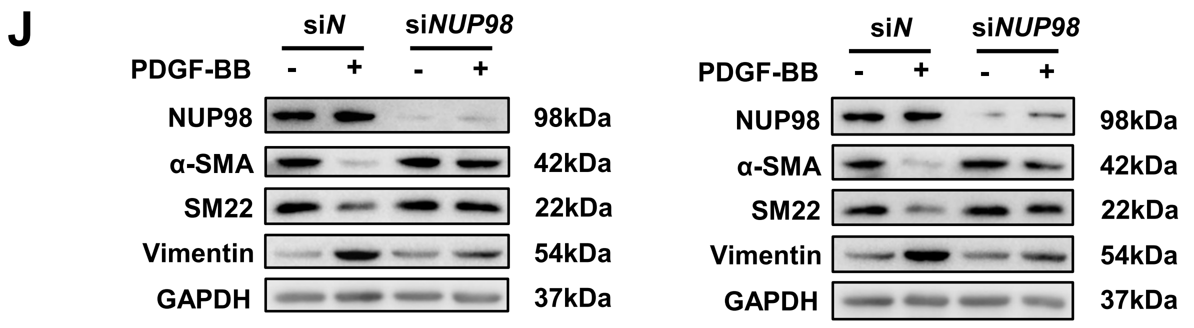

No.1-2

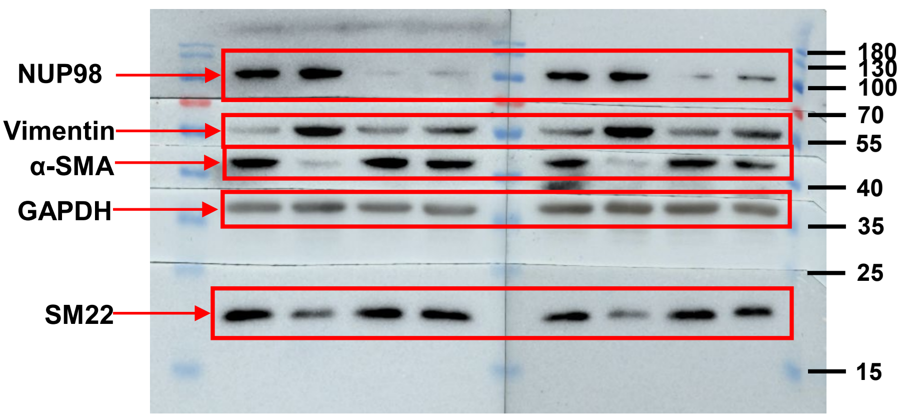

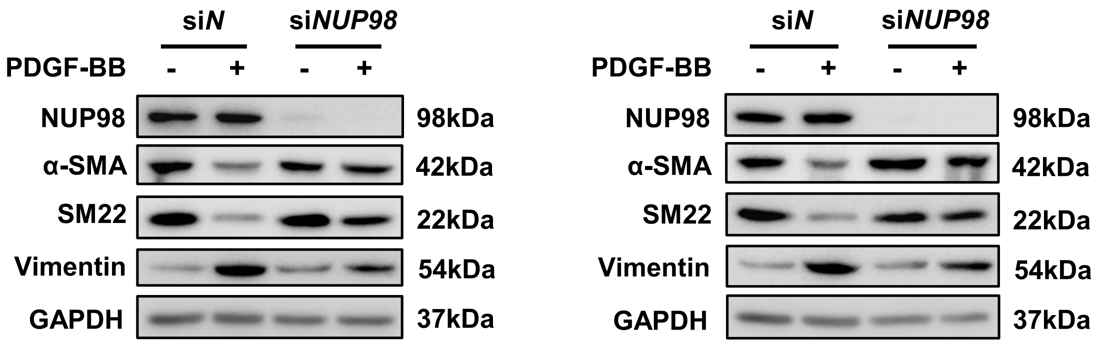

No.3-4

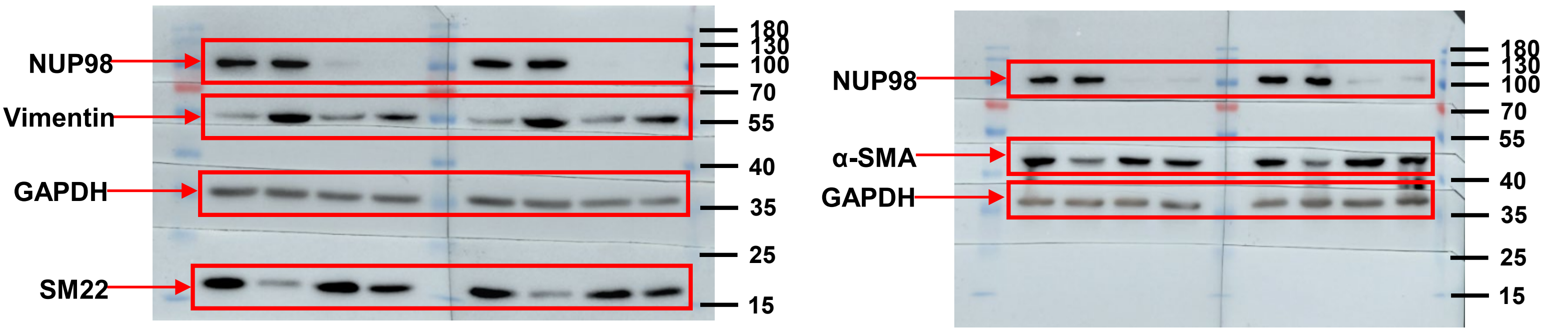

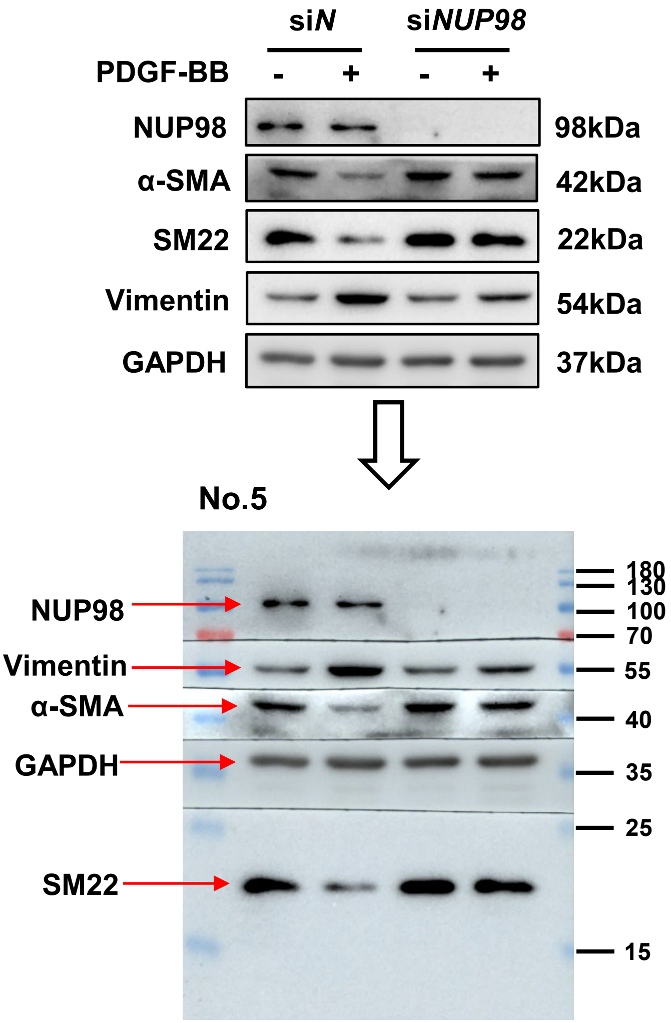
siN
siNUP98
PDGF-BB
- + - +
NUP98
α-SMA
SM22
Vimentin
GAPDH
98kDa
42kDa
22kDa
54kDa
37kDa
No.5
NUP98
Vimentin
α-SMA
GAPDH
SM22
180
130
100
70
55
40
35
25
15

Full unedited gel for Supplemental Figure 11B n=3

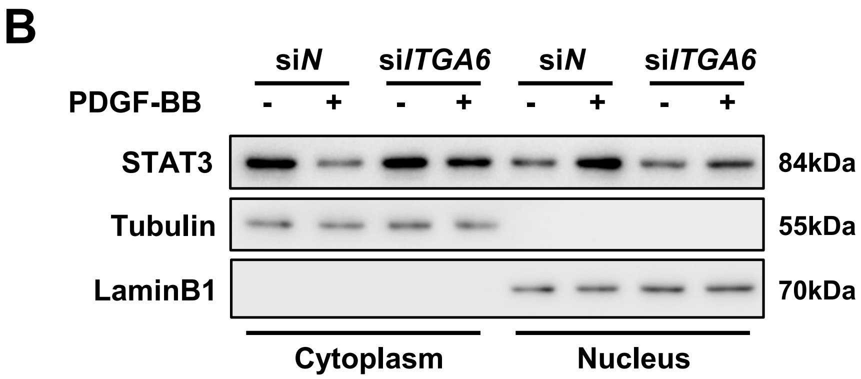

No.1

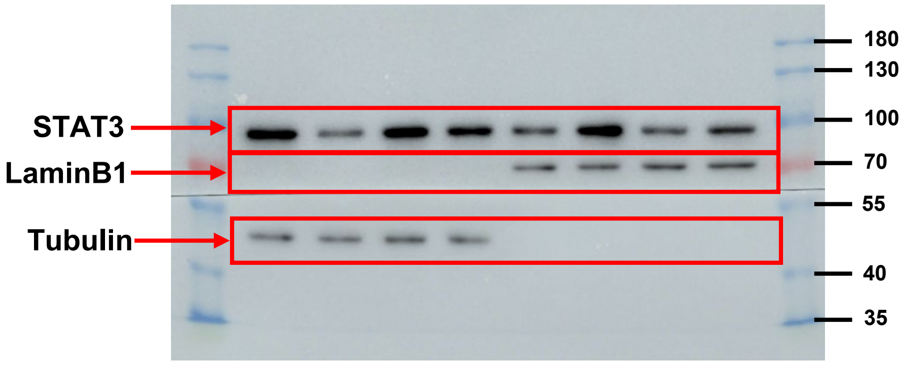

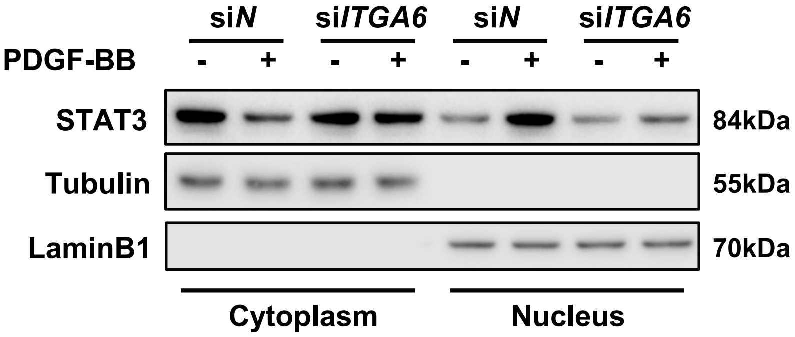

No.2

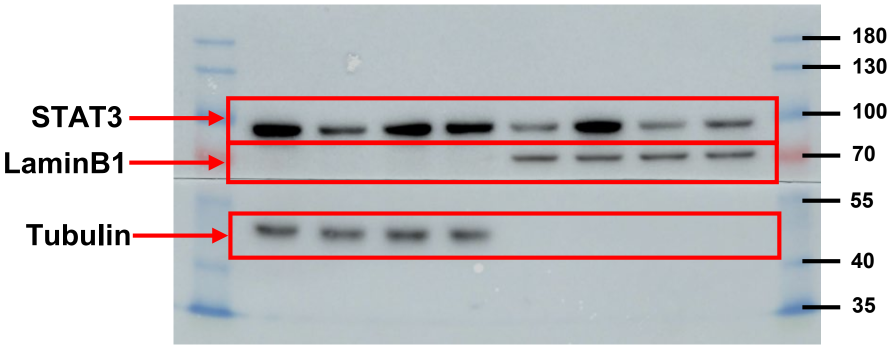

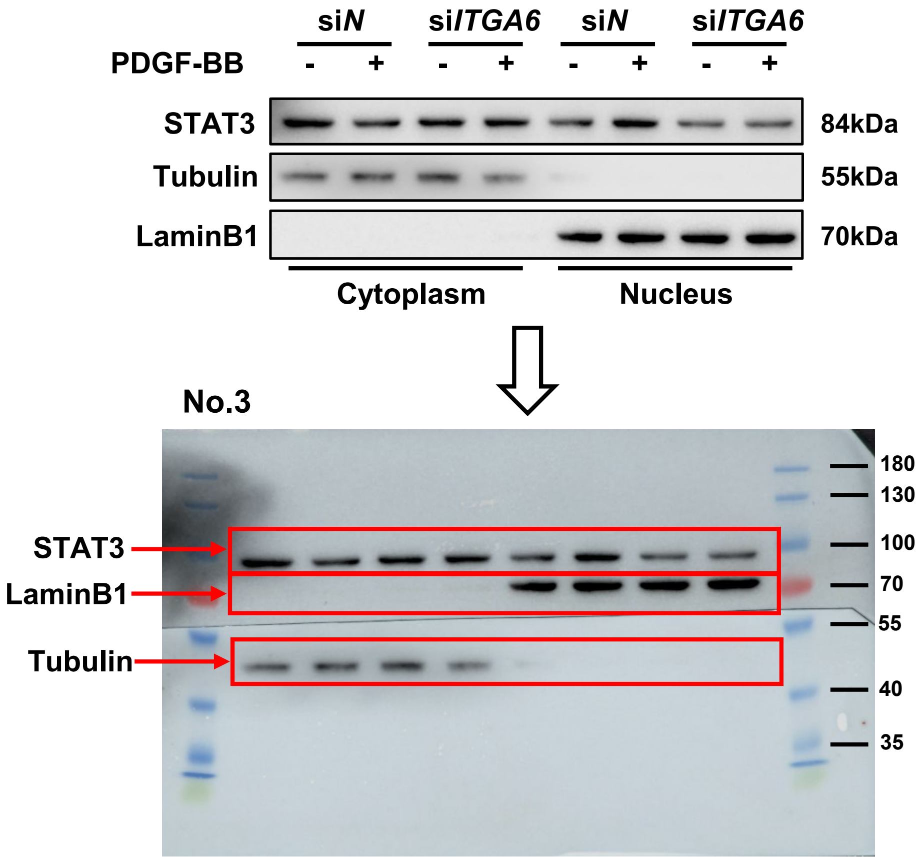

siN
siITGA6
siN
siITGA6
PDGF-BB
- + - + - + - +
STAT3
84kDa
Tubulin
55kDa
LaminB1
70kDa
Cytoplasm
Nucleus
No.3
STAT3
LaminB1
Tubulin
180
130
100
70
55
40
35

Full unedited gel for Supplemental Figure 11C n=6

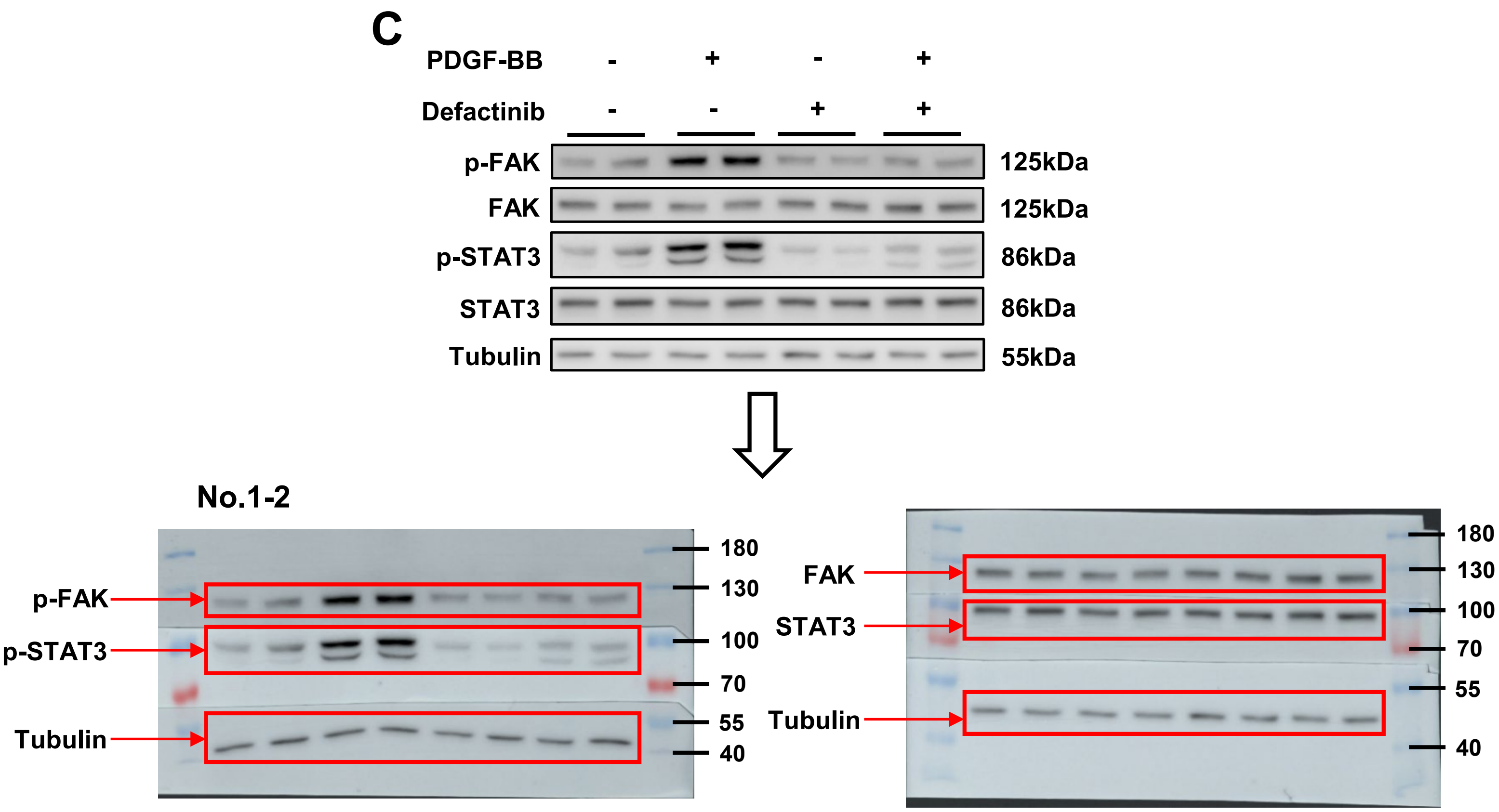

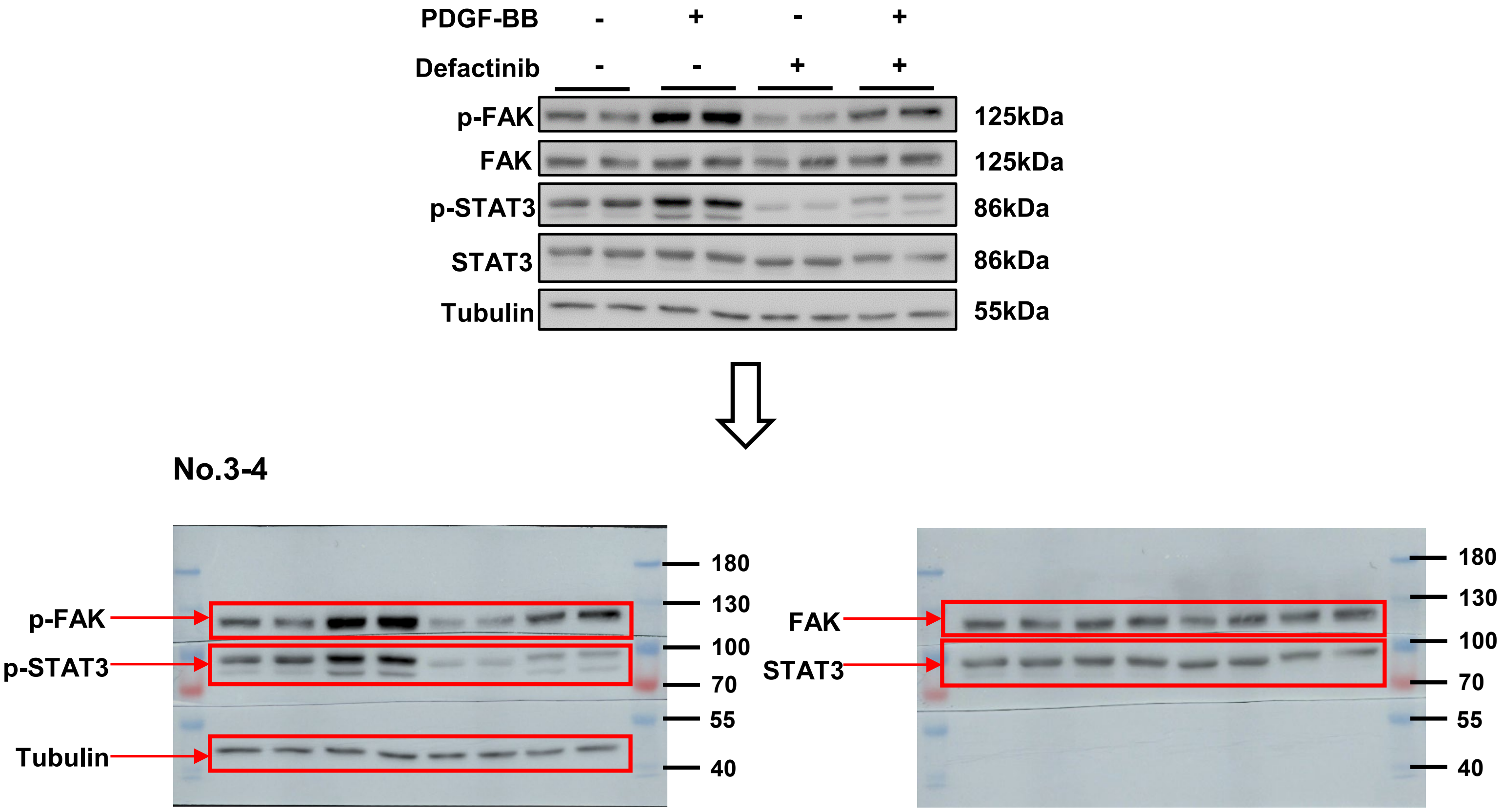

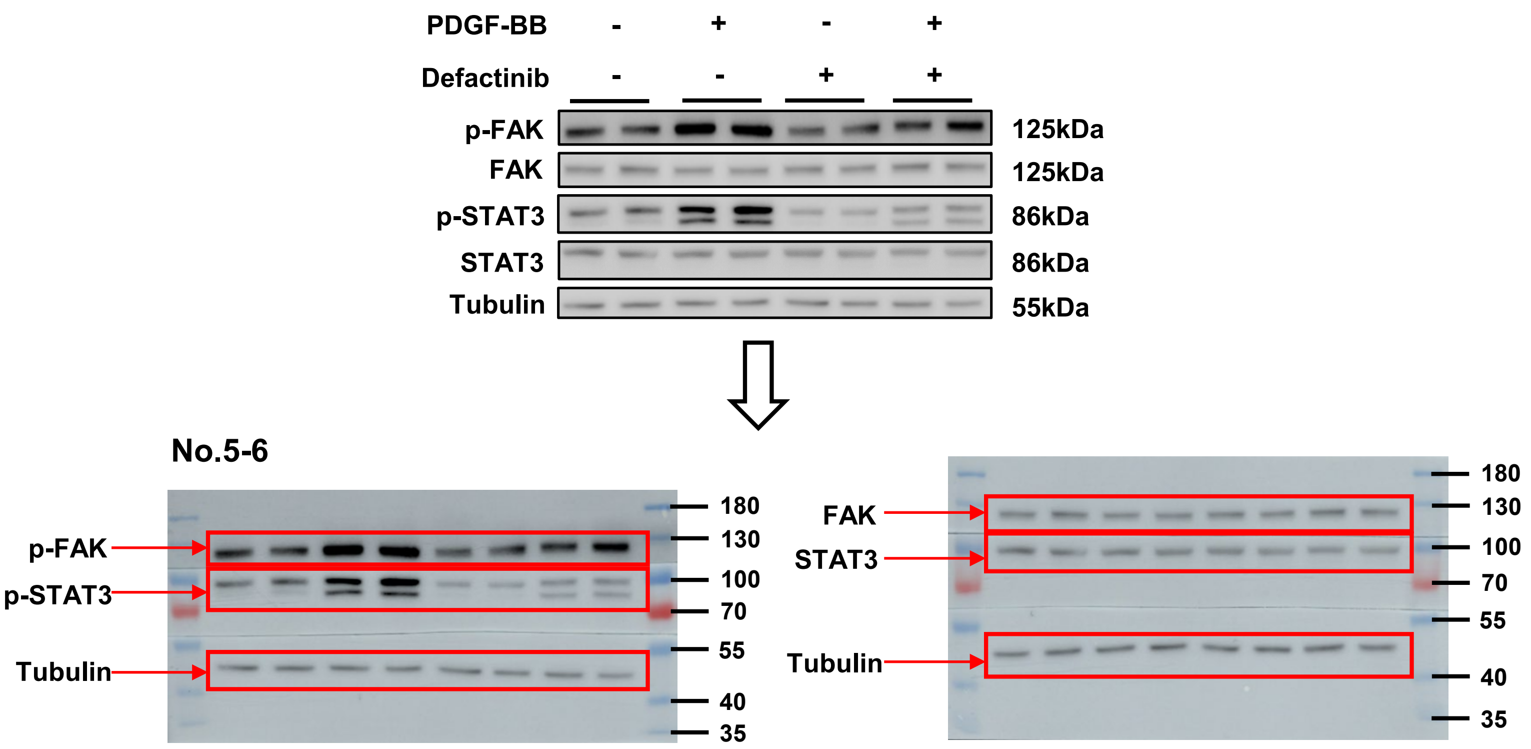
PDGF-BB - + - +
Defactinib - - + +
p-FAK
FAK
p-STAT3
STAT3
Tubulin
125kDa
125kDa
86kDa
86kDa
55kDa
No.5-6
p-FAK
p-STAT3
Tubulin
180
130
100
70
55
40
35
FAK
STAT3
Tubulin
180
130
100
70
55
40
35

Full unedited gel for Supplemental Figure 11E n=3

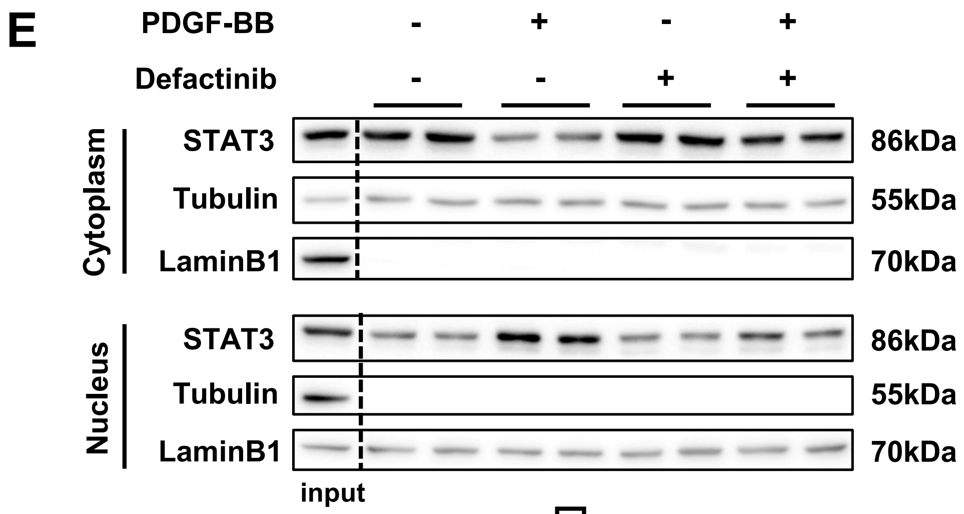

No.1-2

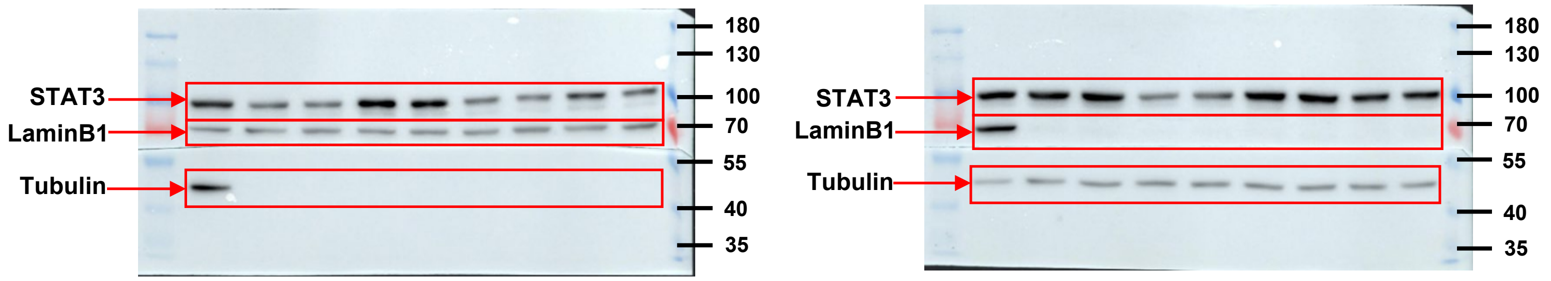

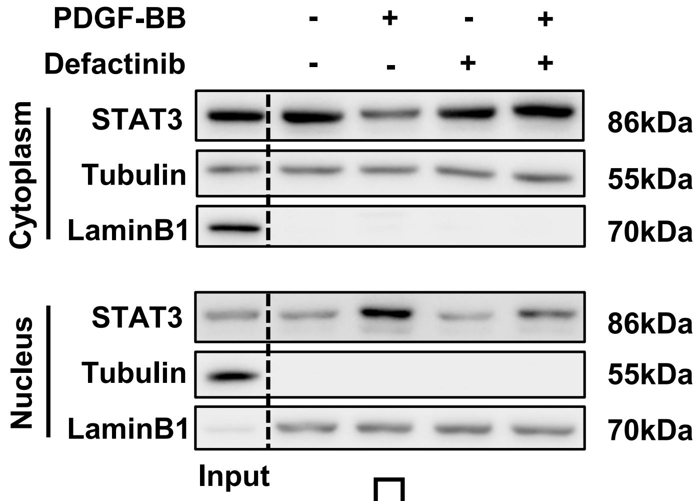

No.3

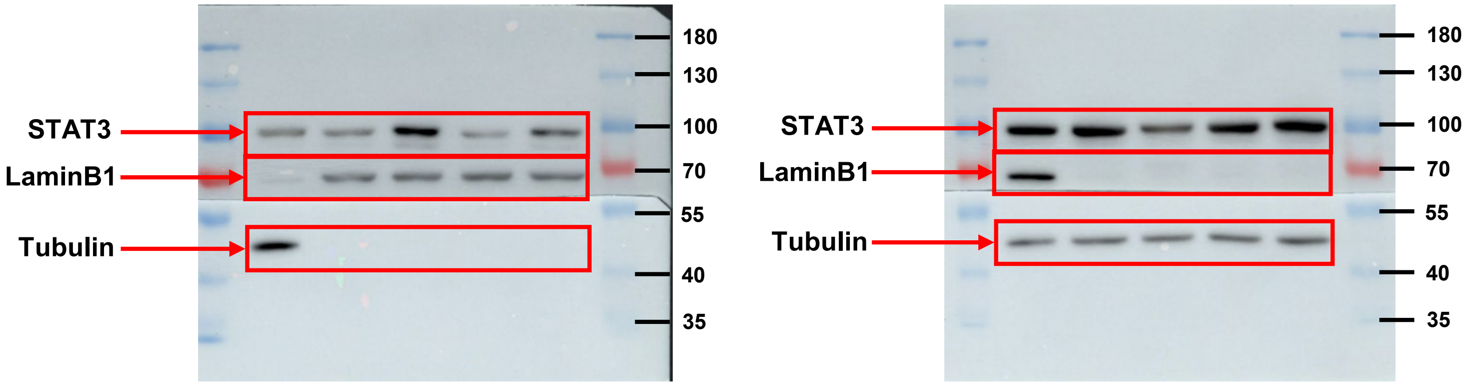

Full unedited gel for Supplemental Figure 11G n=6

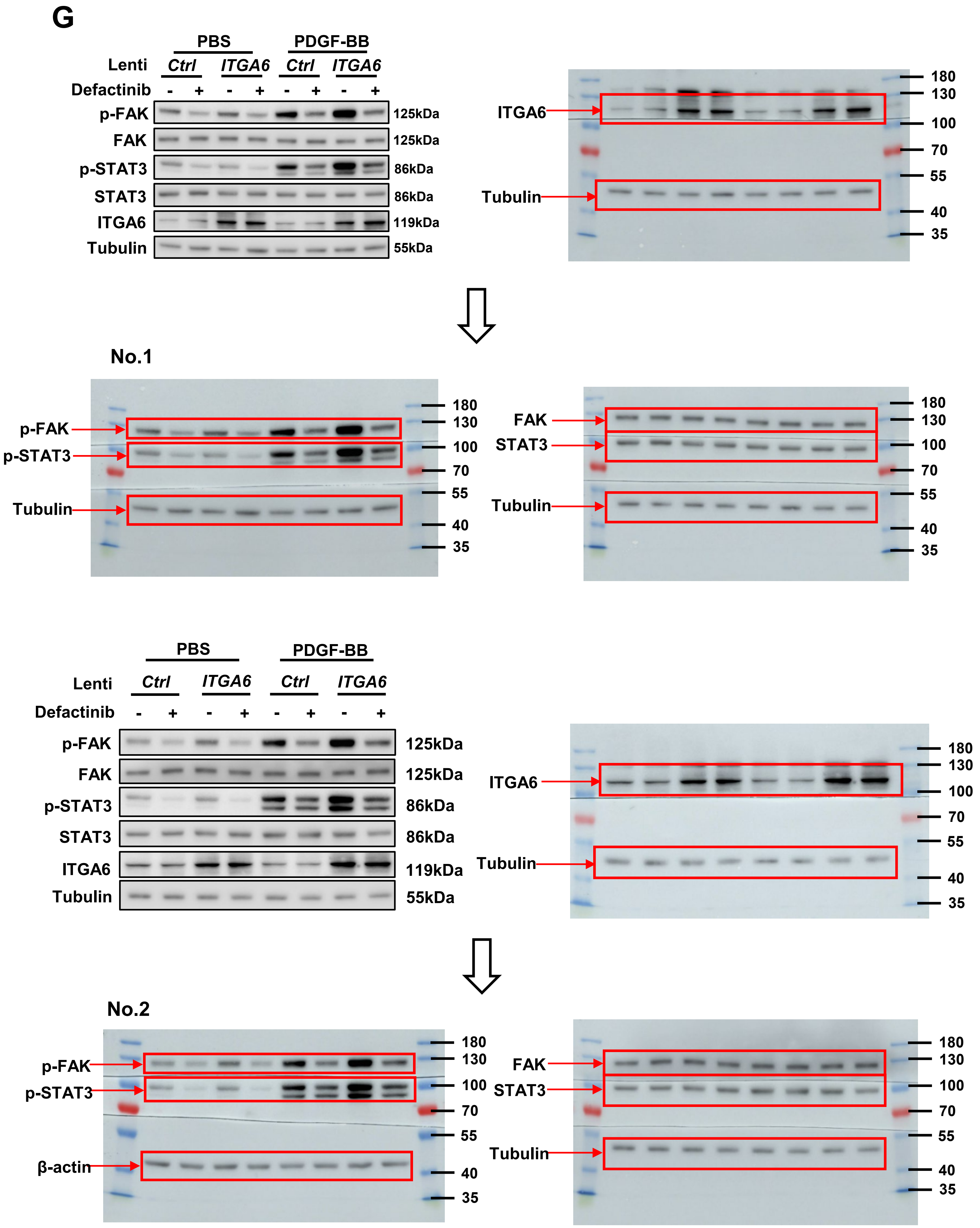

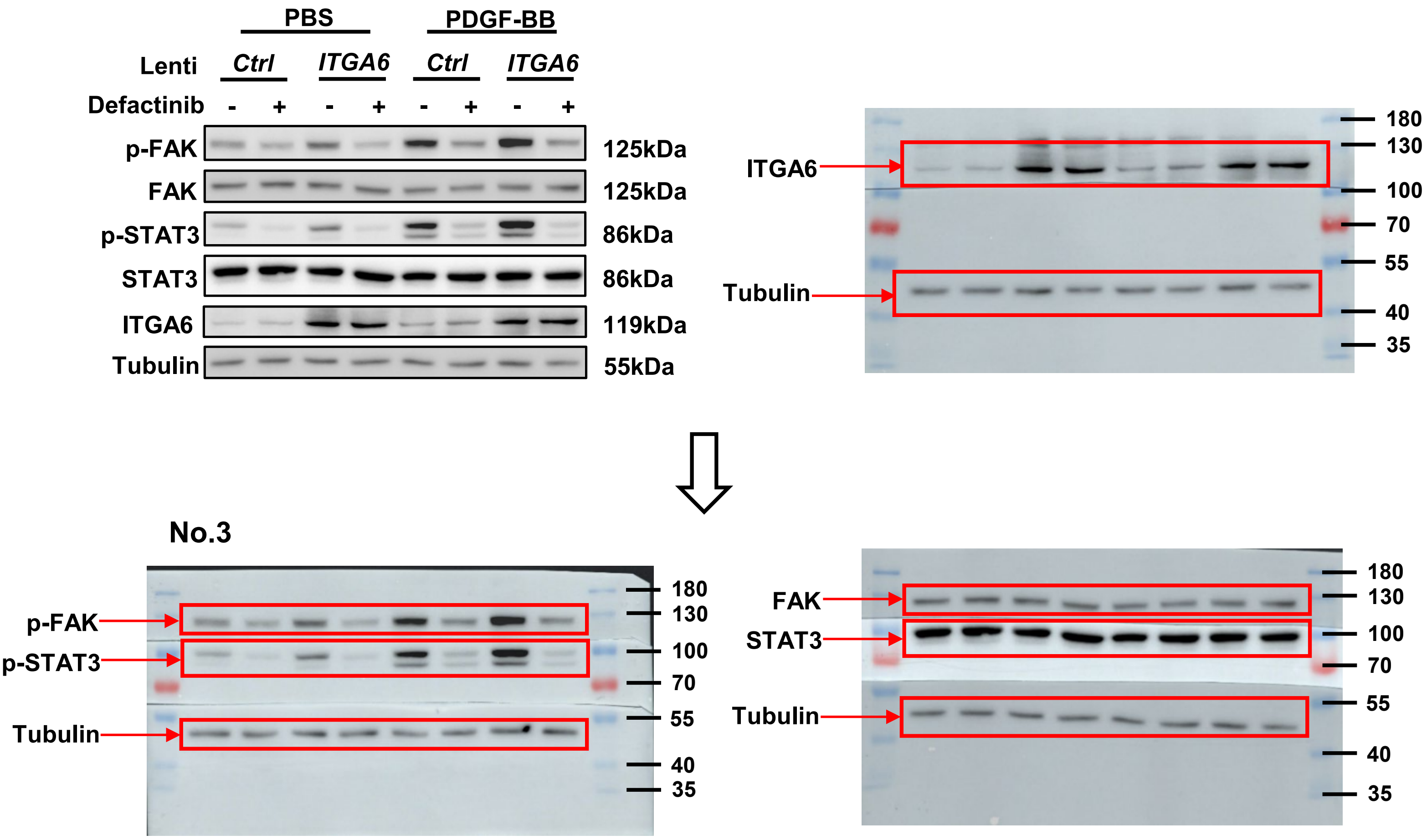
PBS
PDGF-BB
Lenti
Ctrl
ITGA6
Ctrl
ITGA6
Defactinib
- + - + - + - +
p-FAK
125kDa
FAK
125kDa
p-STAT3
86kDa
STAT3
86kDa
ITGA6
119kDa
Tubulin
55kDa
ITGA6
Tubulin
180
130
100
70
55
40
35
No.3
p-FAK
p-STAT3
Tubulin
180
130
100
70
55
40
35
FAK
STAT3
Tubulin
180
130
100
70
55
40
35

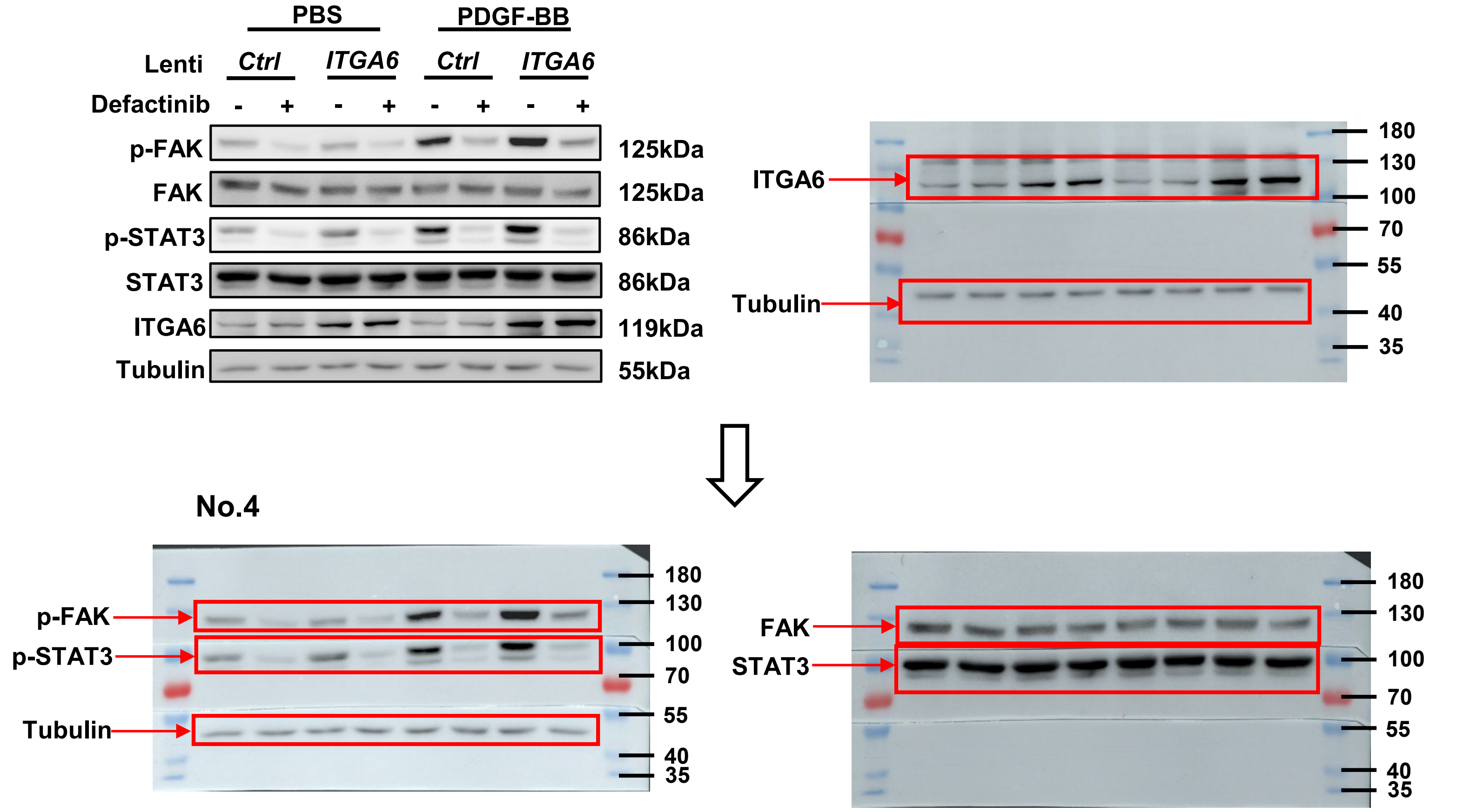
PBS
PDGF-BB
Lenti
Ctrl
ITGA6
Ctrl
ITGA6
Defactinib
- + - + - + - +
p-FAK
125kDa
FAK
125kDa
p-STAT3
86kDa
STAT3
86kDa
ITGA6
119kDa
Tubulin
55kDa
ITGA6
Tubulin
180
130
100
70
55
40
35
No.4
p-FAK
p-STAT3
Tubulin
180
130
100
70
55
40
35
FAK
STAT3
180
130
100
70
55
40
35

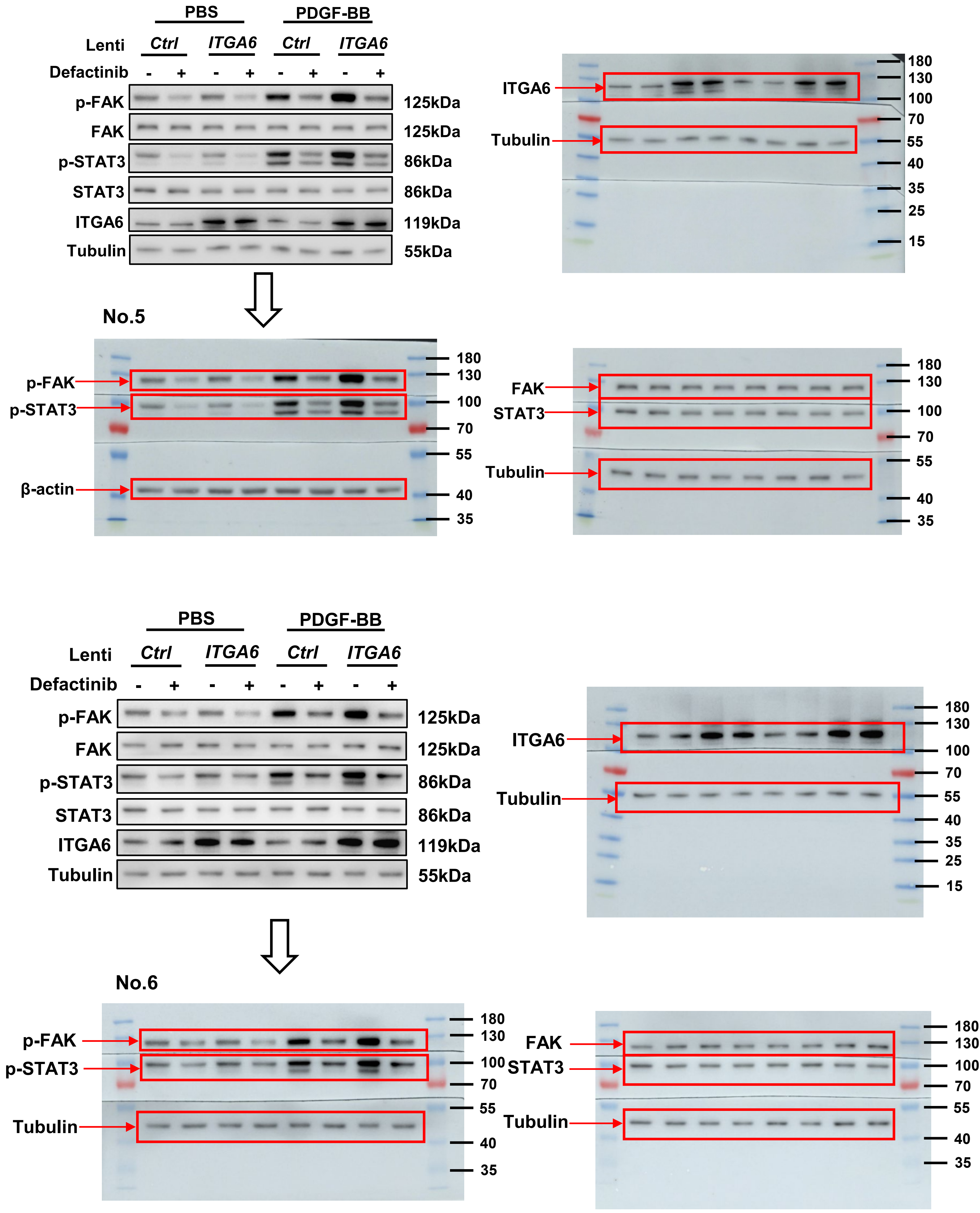

PBS
PDGF-BB
Lenti
Ctrl
ITGA6
Ctrl
ITGA6
Defactinib
- + - + - + - +
p-FAK
125kDa
FAK
125kDa
p-STAT3
86kDa
STAT3
86kDa
ITGA6
119kDa
Tubulin
55kDa
ITGA6
Tubulin
180
130
100
70
55
40
35
25
15
No.5
p-FAK
p-STAT3
β-actin
180
130
100
70
55
40
35
FAK
STAT3
Tubulin
180
130
100
70
55
40
35
PBS
PDGF-BB
Lenti
Ctrl
ITGA6
Ctrl
ITGA6
Defactinib
- + - + - + - +
p-FAK
125kDa
FAK
125kDa
p-STAT3
86kDa
STAT3
86kDa
ITGA6
119kDa
Tubulin
55kDa
ITGA6
Tubulin
180
130
100
70
55
40
35
25
15
No.6
p-FAK
p-STAT3
Tubulin
180
130
100
70
55
40
35
FAK
STAT3
Tubulin
180
130
100
70
55
40
35

Full unedited gel for Supplemental Figure 11H n=3

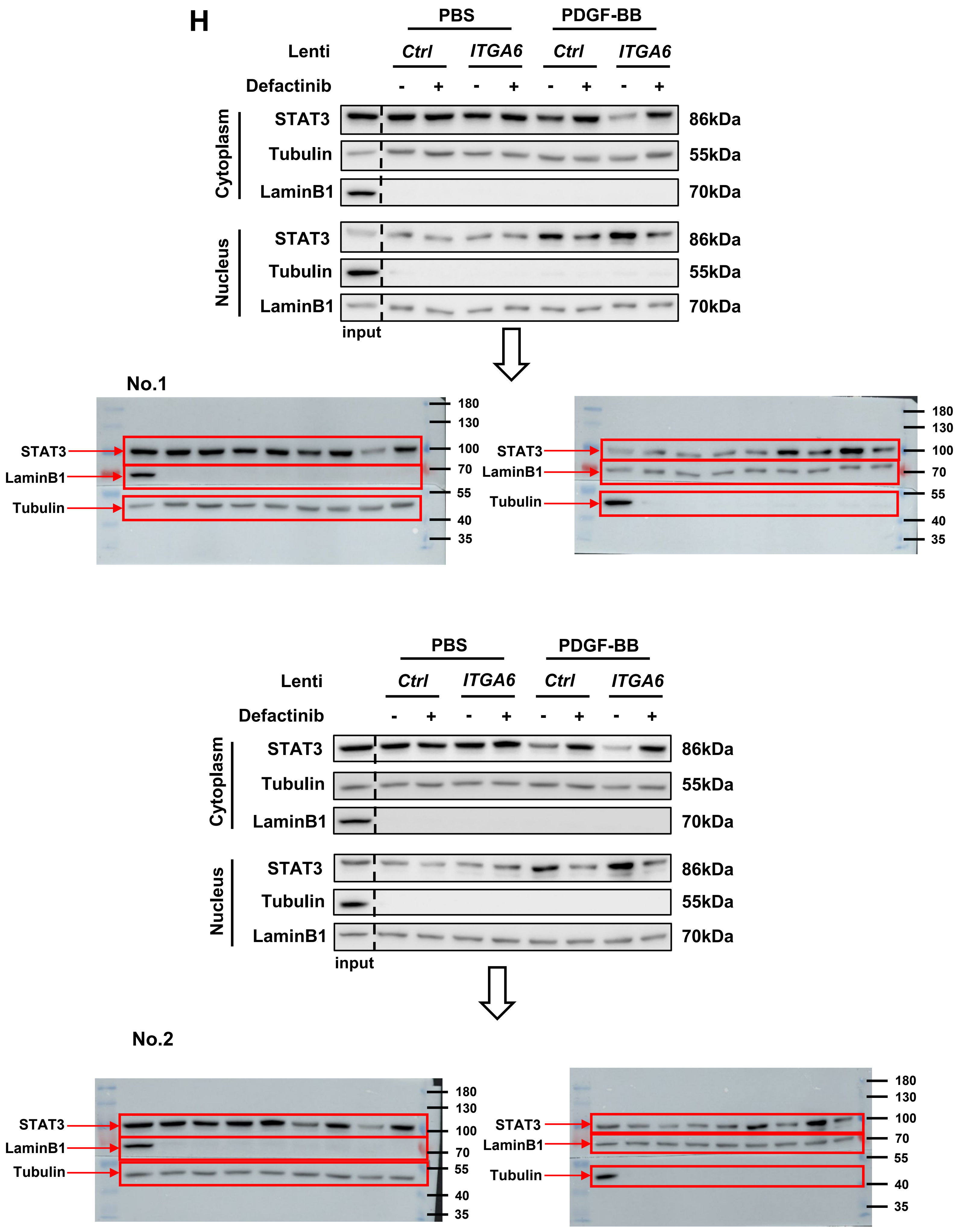

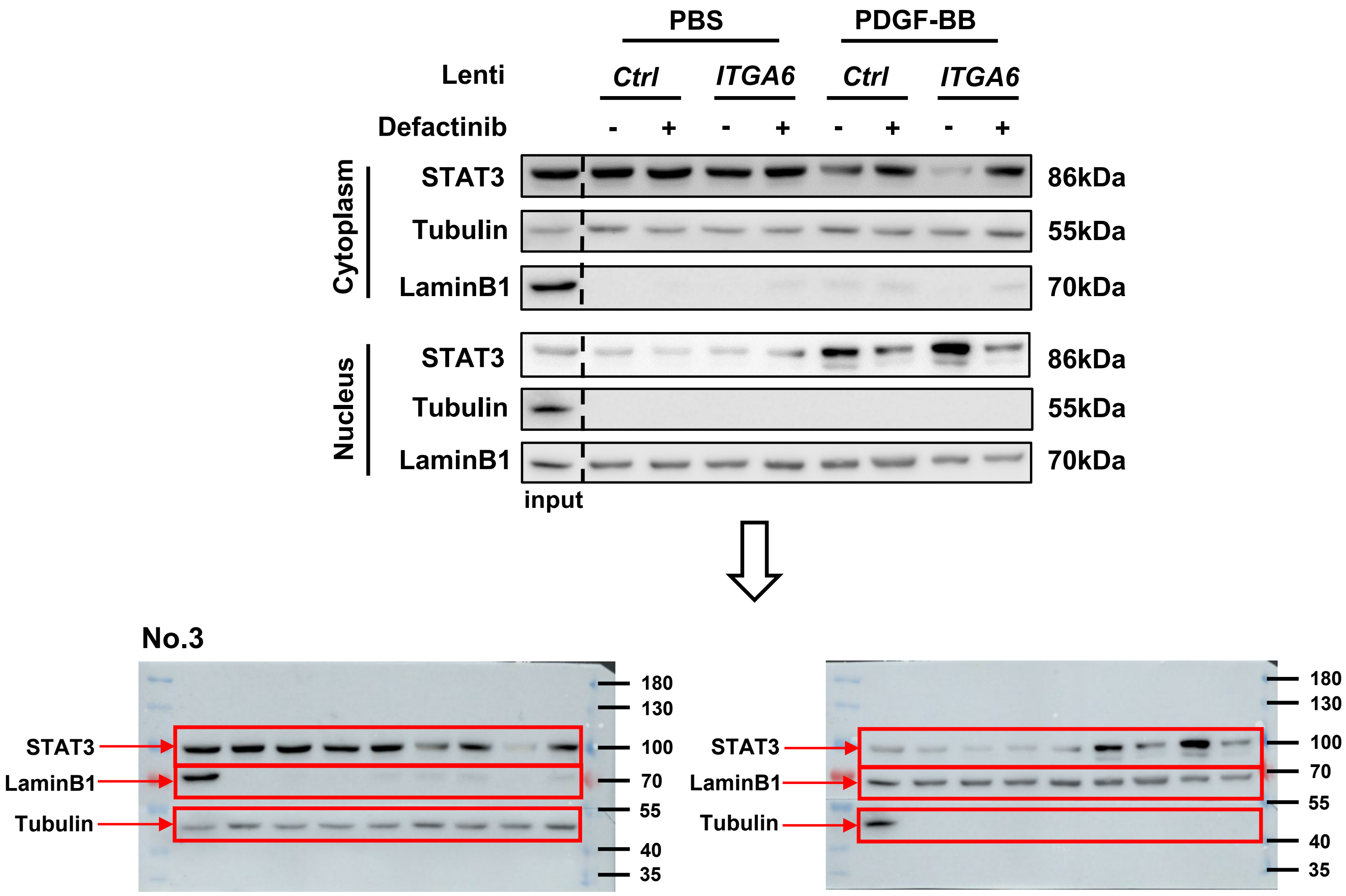
PBS
PDGF-BB
Lenti
Ctrl
ITGA6
Ctrl
ITGA6
Defactinib
- + - + - + - +
Cytoplasm
STAT3
Tubulin
LaminB1
86kDa
55kDa
70kDa
Nucleus
STAT3
Tubulin
LaminB1
86kDa
55kDa
70kDa
input
No.3
STAT3
LaminB1
Tubulin
180
130
100
70
55
40
35
STAT3
LaminB1
Tubulin
180
130
100
70
55
40
35
