# Supplementary material for: Corrigendum to HINT1 aggravates aortic aneurysm by targeting ITGA6/FAK axis in vascular smooth muscle cells
Source: J Clin Invest. 2026 Nov 16;136(22):e213937. doi: 10.1172/JCI213937 (PMC13767657; doi:10.1172/JCI213937)
Supplement: Supplementary file 1 [file jci-136-213937-s229.pdf]

Supplemental Material

HINT1 Aggravates Aortic Aneurysm by Targeting ITGA6/FAK Axis in Vascular Smooth Muscle Cells

<u>Supplemental Tables</u>

Supplemental table 1. Characteristics of aortic aneurysm patients

	Patient-1	Patient-2	Patient-3	Patient-4	Patient-5	Patient-6
Age (y)	84	60	59	75	64	59
Sex, Male (M) / Famale (F)	M	M	M	M	M	M
Smoking	Yes	No	Yes	Yes	Yes	Yes
Body mass index (kg/m^2)	22.86	23.15	22.49	20.15	25.06	27.02
Hypertension	Yes	No	Yes	Yes	Yes	Yes
Diabetes Mellitus	No	No	No	No	No	No
Total cholesterol (mmol/L)	2.53	5.38	3.64	3.26	2.65	3.57
HDL (mmol/L)	0.72	0.83	1.01	1.01	0.78	0.98
Triglyceride (mmol/L)	1.31	1.8	2.02	0.42	1.03	2.24
CCB	Yes	No	No	No	No	No
ACEI	No	No	No	No	Yes	No
ARB	No	No	Yes	No	No	No
Beta-blockers	No	No	No	Yes	Yes	Yes
Statins	Yes	No	Yes	No	No	No
Aspirin	Yes	No	No	No	No	No
Duration of the disease	3 days	2 months	2 weeks	1 months	5 years	6 days

HDL, high-density lipoprotein; CCB, calcium channel blocker; ACEI, angiotensin-converting enzyme inhibitor; ARB, angiotensin receptor blocker.

Supplemental table 2. The age and sex information of the controls.

	Control-1	Control-2	Control-3	Control-4	Control-5	Control-6
Age (y)	65	61	63	70	58	60
Sex, Male (M) / Famale (F)	F	M	M	M	M	M

Supplemental table 3. The primer sequences used for quantitative polymerase chain reaction (qPCR) analysis were described

Gene	Forward (5' to 3' sequence)	Reverse (5' to 3' sequence)	Species
Acta2	CGCCTCCAGTTCCTTTCCAA	AGAGGGGGCCACCCTATAAT	Mouse
Tagln	GGTGACATCACTGCCTA	GACTGCACTTCTCGGCTCAT	Mouse
Cnn1	GGGTTACGGTTTGGGGAGAT	AACTCAGTGCTTCCTTCGGG	Mouse
Opn	AATCTCCTTGCGCCACAGAA	GGACATCGACTGTAGGGACG	Mouse
Klf4	TGGCCATCGGACCTACTTATC	CATGTCAGACTCGCCAGGTG	Mouse
Myh10	GGAATCCTTTGGAAATGCGAAGA	GCCCCAACAATATAGCCAGTTAC	Mouse
Hint1	GCGACACGATCTTCGGCAA	GGTGCTTGAGGGGAAATGTCA	Mouse
Itga6	GGGATCGTCCGTGTAGAACAA	TCTCTCCACCAACTTCATAGGG	Mouse
Itga7	GGATTCCGAGGTGCGATTTTC	GCCGGTGGTAAGAACAGTCC	Mouse
Itga8	CGAAGCCGAACTCTTTGTTATCA	GGCCTCAGTCCCTTGTTGT	Mouse
Itgb8	TGCATGTTGTAACGTCAAGTGA	GATGCTGACACATCAACCAGATA	Mouse
HINT1	GATCATCCGCAAGGAAATACCA	TCACCACCATTCGATAACCCT	Human
ITGA6	ATGCACGCGGATCGAGTTT	TTCCTGCTTCGTATTAACATGCT	Human
ACTA2	AGGAAGGACCTCTATGCTAACAAT	AACACATAGGTAACGAGTCAGAGC	Human
TAGLN	AGTGCAGTCCAAAATCGAGAAG	CTTGCTCAGAATCACGCCAT	Human
CNN1	CTGTCAGCCGAGGTTAAGAAC	GAGGCCGTCCATGAAGTTGTT	Human
OPN	CTCCATTGACTCGAACGACTC	CAGGTCTGCGAAACTTCTTAGAT	Human
KLF4	CGGACATCAACGACGTGAG	GACGCCTTCAGCACGAACT	Human
MYH10	TGGTTTTGAGGCAGCTAGTATCA	AGTCCTGAATAGTAGCGATCCTT	Human

Supplemental Methods

Human Samples

Two types of human abdominal aortic tissues were used in this study: aorta samples from aortic aneurysm patients and normal aorta samples from organ donors. The diagnosis of aortic aneurysm was confirmed by computed tomographic angiography (Aortic aneurysm patients were recruited with abdominal aortic diameters ⩾55 mm in men). Abdominal aortic tissues from aortic aneurysm patients were freshly isolated during surgical repair for aortic aneurysm. The aortic tissue was placed in ice-cold physiological salt solution immediately upon removal, followed by stripped of the periaortic tissue and mural thrombus. The aortic tissue was divided into several segments, which were either fixed in 4% paraformaldehyde for histologic analyses or snap-frozen in liquid nitrogen followed by storage at -80°C for RNA or protein extraction. Control aortic tissues were collected from donors for kidney transplantation. To minimize the effect of aortic damage caused by poor circulation, we selected donors with cardiac arrest for less than 60 minutes and the aortic tissues were collected within 60 minutes of termination of life support. The aortic tissue was placed in ice-cold physiological salt solution immediately upon removal, followed by stripped of the periaortic tissue and mural thrombus. The aortic tissue was divided into several segments, which were either fixed in 4% paraformaldehyde for histologic analyses or snap-frozen in liquid nitrogen followed by storage at -80°C for RNA or protein extraction.

Reagents

Antibodys against HINT1 (ab124912), α-sma (ab5694), Tagln (ab14106), Vimentin (ab92547), TFAP2A (ab108311) for western blotting were obtained from Abcam (Cambridge, MA, UK). Antibodys against ITGA6 (27189-1-AP), Tubulin (11224-1-AP), β-actin (66009-1-lg), GAPDH (60004-1-Ig) for western blotting, HA-tag (51064-2-AP; 66006-2-lg), Flag-tag (20543-1-AP; 66008-4-Ig), GST-tag（66001-2-Ig）for western blotting and immunoprecipitation assay were obtained from Proteintech (Chicago, IL, USA); antibody against HINT1 (sc-271790) for western blotting, immunoprecipitation assay and immunofluorescence, α-sma (sc-53015), TFAP2A (sc-12726) for immunofluorescence were obtained from Santa Cruz Biotechnology (CA, USA). Antibody against Phospho-FAK (Tyr397) (44-624G) for western blotting was obtained from Thermo Fisher Scientific (Rockford, CA, USA). Antibodys against NUP98 (5246S), FAK (3285T), STAT3 (9139T), Phospho-STAT3 (Tyr705) (9145T) for western blotting were obtained from Cell Signaling Technology (Boston, MA, USA). Antibody against LaminB1 for western blotting was obtained from Beyotime (Shanghai, China). Normal mouse IgG (sc-2025) used as a negative control for Co-IP assay was obtained from Santa Cruz Biotechnology, Inc. (Dallas, USA). Normal rabbit IgG (A7016) used for Co-IP assay was obtained from Beyotime (Shanghai, China). Nuclei were stained with 4,6-diamidino-2-phenylindole (DAPI, #0100-20) from SouthernBiotech (AL, USA). Alexa Fluor™ 488 donkey anti-mouse (H+L) antibody (A21202), Alexa Fluor™ 594 donkey anti-Rabbit (H+L) antibody (A21207) for confocal fluorescence microscopy were purchased from Thermo Fisher Scientific

(Rochester, USA). Angiotensin II (Ang II, ab120183) was purchased from Abcam (Cambridge, MA, UK). Defactinib (S7654) was purchased from Selleck (Shanghai, China). PDGF-BB (100-14B-50) was purchased from PeproTech, Inc. (Rocky Hill, USA). Lipofectamine® 3000 Transfection Reagent (L3000015) used for siRNAs and plasmids transfection were purchased from Invitrogen (Carlsbad, USA). Firefly & Renilla Luciferase Reporter Assay Kit was purchased from meilunBio (Dalian, China). Chromatin Immunoprecipitation (ChIP) Assay Kit was purchased from Beyotime (Shanghai, China).

Aortic Aneurysm Animal Model

To establish an angiotensin II (Ang II)-induced Aortic Aneurysm model, a mini osmotic pump (Alzet model 2004, 28-day delivery; Durect Corporation, USA) loaded with Ang II (1000 ng/kg/min) or saline (0.9% NaCl) was implanted subcutaneously at the dorsum of the neck for 28 days. The aorta was considered to be aneurysmal if the abdominal aorta diameter increased by 50% or more.

Blood pressure measurement

Arterial blood pressure (BP) was measured by the mouse-tail cuff method without anesthesia using the automated BP-2000 Blood Pressure Analysis System (Visitech Systems, Apex, NC, USA).

siRNA transfection

RASMC or HASMC were transfected with 50 nM siRNA targeting *Hint1*, *Itga6*, *Tfap2a* or *Nup98* using Lipofectamine 3000 according to the manufacturer's protocol.

Scrambled siRNA was used as a negative control (NC). After 24 h, culture medium was changed to complete growth medium.

Quantitative polymerase chain reaction (qPCR)

Total RNA was extracted using Trizol (Takara, Japan), followed by synthesizing cDNA with HiScript® II Q RT SuperMix (Vazyme). Real-time PCR amplification involved the use of an ABI QuantStudio^{TM} 6 Real-Time PCR System (Rockford, CA, USA). The mRNA expression levels were normalized to 18S or GAPDH. The primer sequences for the target genes are listed in Supplementary material, *Table S1*.

Co-immunoprecipitation

Total proteins of RASMC, HASMC or HEK293T cells were extracted by the lysis buffer (40 mM 349 Hepes, pH 7.4, 2 mM EDTA, 10 mM pyrophosphate, 10 mM glycerophosphate, 0.5% Triton) supplemented with protease inhibitor cocktail. Supernatants were harvested after centrifugation at 12,000 g for 10 min. Cell lysates were mixed with IgG, HINT1, GST-tag, HA-tag, or Flag-Tag antibody at 4°C overnight, followed by precipitation with protein A/G beads for 4 h at 4°C. After washing, immunoprecipitated complex was immediately identified by SDS-PAGE and immunoblotting.

Extraction of cytosolic and nuclear fraction

For separation of cytosolic and nuclear fractions, HASMC were washed with cold PBS and scraped with lysis buffer (10 mmol/L HEPES, 0.1 mmol/L EDTA, 7 1 mmol/L KCl, 50 mmol/L NaF, 0.1 mmol/L EGTA, 1 mmol/L Na_3VO_4, 1 mmol/L DTT, and cocktail 100×). The homogenate was then oscillated and centrifuged for 10,000 rpm for 5

minutes at 4°C. The supernatant collected was the cytosolic fraction. Next, the pellet was further lysed with buffer containing 20 mmol/L HEPES, 1 mmol/L EDTA, 0.4 mol/L NaCl, 50 mmol/L NaF, 1 mmol/L EGTA, 25% Glycerol, 1 mmol/L DTT and cocktail and centrifuged at 10,000 rpm for 10 minutes at 4°C, the supernatant was reserved as the nuclear fraction.

In situ zymography

MMP activity was determined by in situ zymography, using an EnzChek Gelatinase/Collagenase assay kit. The suprarenal abdominal aortic tissue of mice was embedded into OCT solution and rapidly frozen with dry ice. Freshly cut frozen aortic sections were incubated with a fluorogenic gelatin substrate (DQ gelatin, D12054, Thermo Fisher Scientific) at a concentration of 25 μg/mL at 37°C for 24 hours, in a dark environment. MMP-catalyzed hydrolysis of DQ gelatin resulted in fluorescence, with the fluorescence intensity directly positively correlating to MMP activity. The samples were then fixed in 4% PFA and stained with DAPI. Proteolytic activity was detected as green fluorescence (at 495 nm absorption/515 nm emission) by confocal microscopy (Zeiss LSM 800). To eliminate the interference of vascular tissue autofluorescence, negative control was set up. As a negative control, tissue sections were pre-incubated with 10 mM 1,10-phenanthroline (an MMP inhibitor) for 30 minutes before adding DQ gelatin, to inhibit MMP activity in the tissue. This fluorescence produced in this case was identified as tissue autofluorescence, since MMP activity has been inhibited by phenanthroline. Anhydrous ethanol was used to

dissolve the 1,10-phenanthroline since it is sparingly soluble in water. Solvent control was set up to exclude the effect of anhydrous ethanol on MMP activity.

Dual luciferase reporter assay

Wild-type and mutant Itga6 promoters were cloned into pGL6 luciferase reporter vectors, respectively. Recombinant pGL6 dual luciferase reporter vectors were co transfected into HEK293T cells with or without TFAP2A or HINT1 encoding plasmid. Followed fluorescein-labeled reporter gene detection was carried out using a Firefly & Renilla Luciferase Reporter Assay Kit (meilunBio, Dalian, China), according to the manufacturer's instructions.

LC-MS/MS analysis

HINT1 was immunoprecipitated with anti-HINT1 antibody to identify proteins interacted with HINT1 in VSMC. Gel was cut into small pieces and in-gel digestion was performed. Extracted peptides were solubilised in 0.1% TFA, desalted by C18 StageTip (Thermofisher) and then lyophilized.

LC-MS/MS analyses were performed on an EkspertTM nano LC 415 equipped with a TripleTOF® 5600+ (AB Sciex) mass spectrometer. Obtained Peptides were loaded on a Chrom XP C18 trap column (3 μm, 120 Å, 350 μm 0.5 mm; Eksigent) at a flow rate of 3 μL/min for 10 min and eluted through a separation column (3 μm, 120 Å, 75 μm 150 mm; Eksigent) at a flow rate of 300 nL/min using 98% water/0.1% formic acid and 98% acetonitrile/0.1% formic acid as the mobile phases A and B, respectively. The component of mobile phase B was collected on the basis of the following scheme: 0-0.1 min, 5-9%; 0.1-35 min, 9-25%; 35-45 min, 25-50%; 45-45.1 min, 50-80%; 45.1-50

min, 80%; 50-50.5 min, 80-5%; 50.5-60 min, 5%. Raw files were processed by using the MaxQuant software (version: 1.5.2.8), and searched against the UniProt reference sequences for Rat proteome.

RNA sequencing analysis

Total RNA from PDGF-BB-treated MASMC isolated from WT or *Hint1*$^{-/-}$ mice was isolated using Trizol (Takara, Japan) according to manufacturer's instruction. RNA sequencing was performed using a BGISEQ-500 apparatus (BGI, Wuhan, China) with a read length of 50bp. Reads were mapped to the mm10 build of the Mus musculus genome with the Hisat2. DESEQ2 (version 1.4.5) identified the differentially expressed genes (DEG) with fold change>1.5 and FDR<0.05. Gene Ontology (GO) and Kyoto Encyclopaedia of Genes and Genomes (KEGG) enrichment analysis was conducted by using R packages "Clusterprofile".

Isolation of mouse aortic smooth muscle cells (MASMCs)

MASMCs were isolated from the whole aortas of mice. Mice were dissected and the thoracic cavity was exposed, and then perfused with cold sterile PBS. The whole aorta of mice was isolated, followed by rinsing the aorta in cold sterile PBS. Then adventitia and endothelium were gently removed, and the aorta was cut into 1-2 mm explants. The explants were digested in collagenase type II at 37°C with 5% CO_2 for 3-4 hours. Then fetal bovine serum (FBS) was used to stop the enzymatic reaction. Cell pellets were collected by centrifuging at 2,000g for 10 minutes. Cells were resuspended with DMEM supplemented with 10% FBS and 100 U/mL penicillin/streptomycin.

Supplemental Figure and Figure Legends

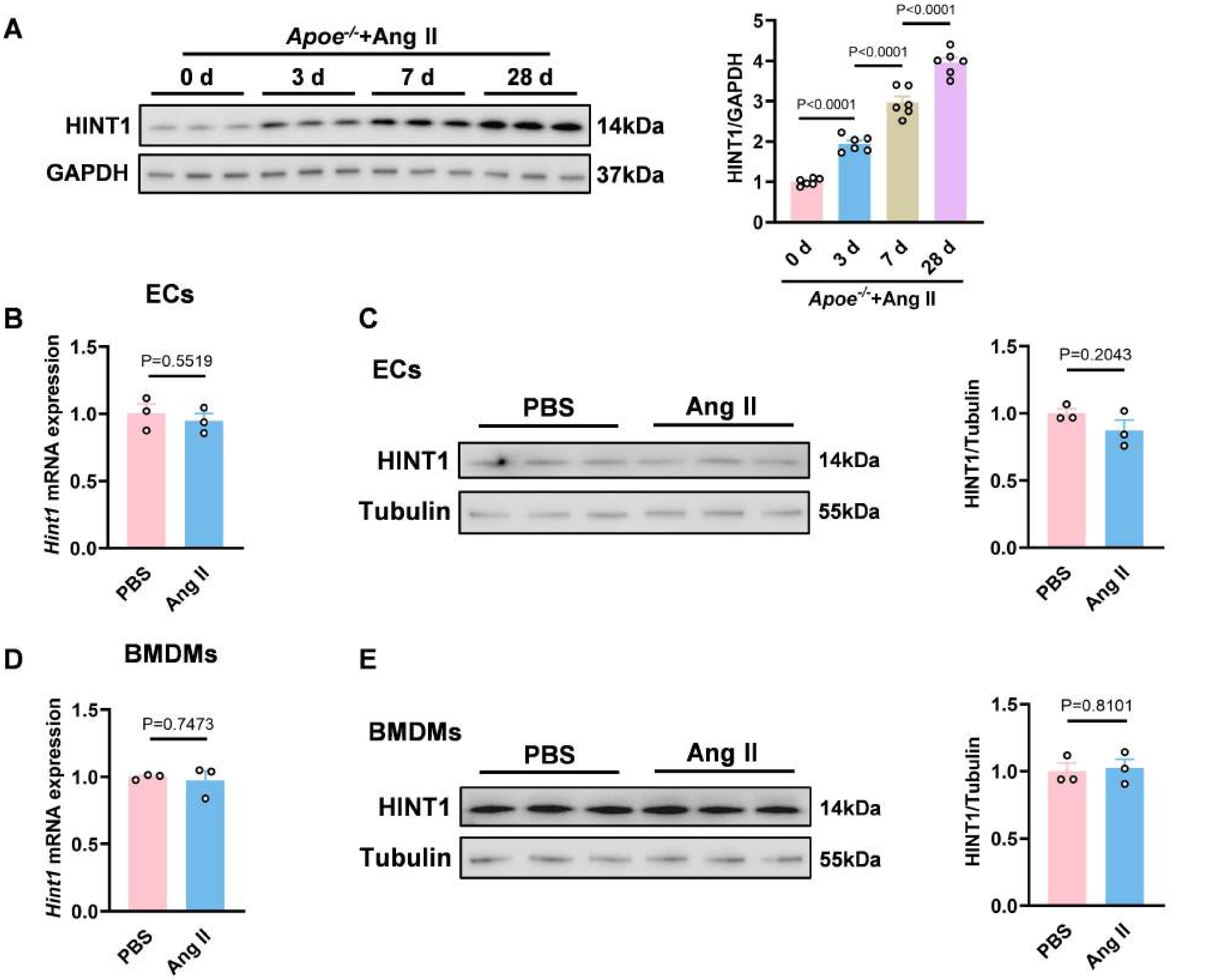

Supplemental Figure 1: HINT1 expression in mice aortic endothelial cells and bone marrow derived macrophages treated with Ang II.

(**A**), Western blotting analysis of *Hint1* in suprarenal abdominal aortas at various timepoints (0, 3, 7, 28 days) after Ang II infusion. n=6 per group. (**B and C**), qPCR (**B**) and Western blotting (**C**) analysis of *Hint1* in isolated mice aortic endothelial cells (ECs) treated with PBS or Ang II (10^{-6} M). n=3 per group. (**D and E**), qPCR (**D**) and Western blotting (**E**) analysis of *Hint1* in isolated mice bone marrow derived macrophages (BMDMs) treated with PBS or Ang II (10^{-6} M). n=3 per group. Statistical analysis was performed by One-way ANOVA for (**A**), Student *t* test for (**B through E**). For all statistical plots, the data are presented as mean ± SEM.

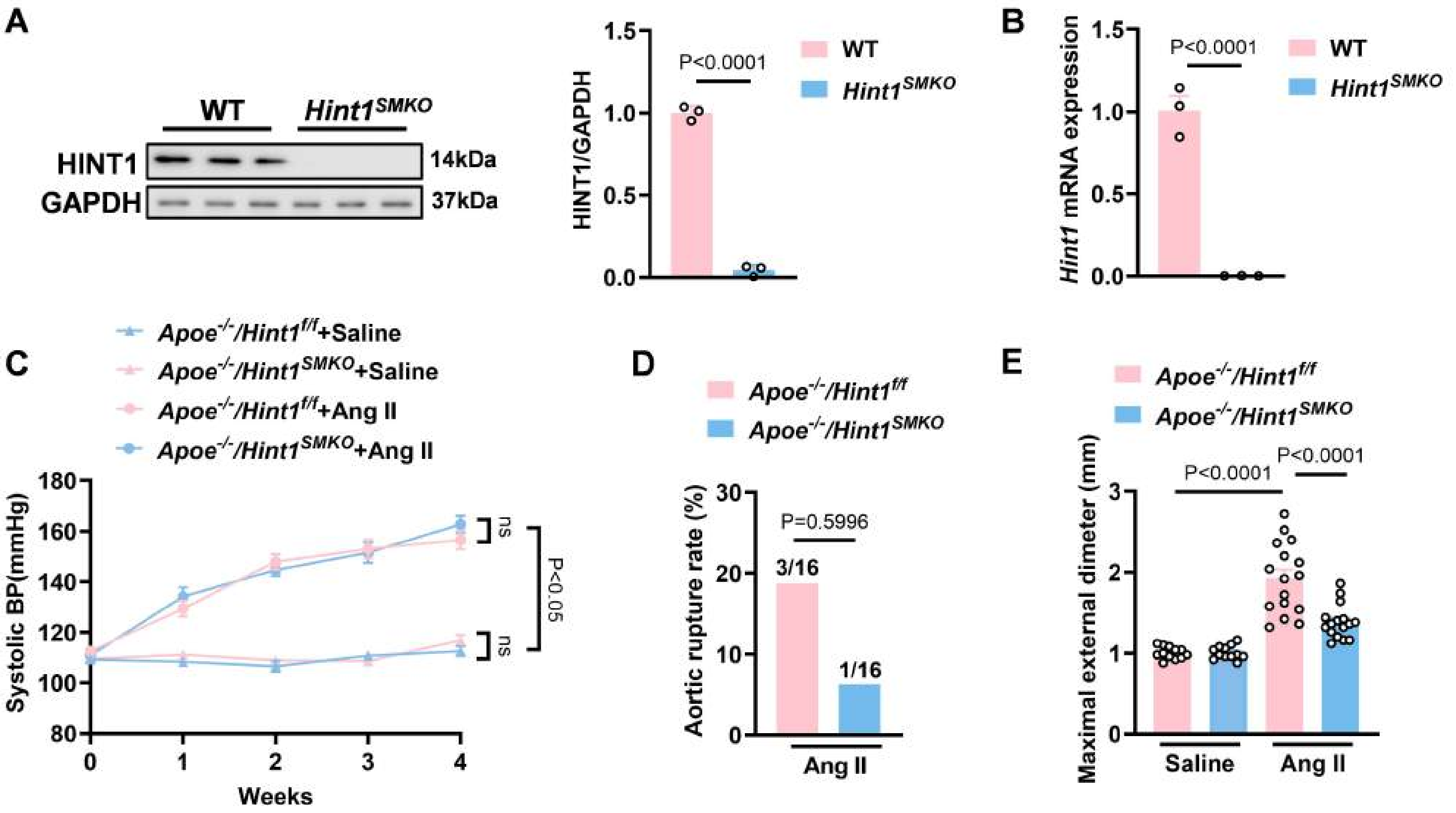

Supplemental Figure 2: *Hint1* deficiency in vascular smooth muscle cells mitigates aortic aneurysm.

(**A and B**), Western blotting (**A**) and qPCR (**B**) analysis of HINT1 expression in MASMCs isolated from aortas of WT and *Hint1^{SMKO}* mice. n=3 per group. (**C**), Systolic blood pressure at 0, 1, 2, 3 and 4 weeks for *Apoe$^{-/-}$/Hint1$^{f/f}$* and *Apoe$^{-/-}$/Hint1^{SMKO}* mice after saline or Ang II infusion. (**D**), The aortic rupture rate in Ang II-infused mice. (**E**), Maximum abdominal aortic diameters were assessed by measuring external aortic diameter from images. Statistical analysis was performed by Student *t* test for (**A and B**), Two-way ANOVA with mixed-effects analysis for (**C**), Fisher exact test for (**D**), One-way ANOVA for (**E**). For all statistical plots, the data are presented as mean ± SEM. ns, no significance.

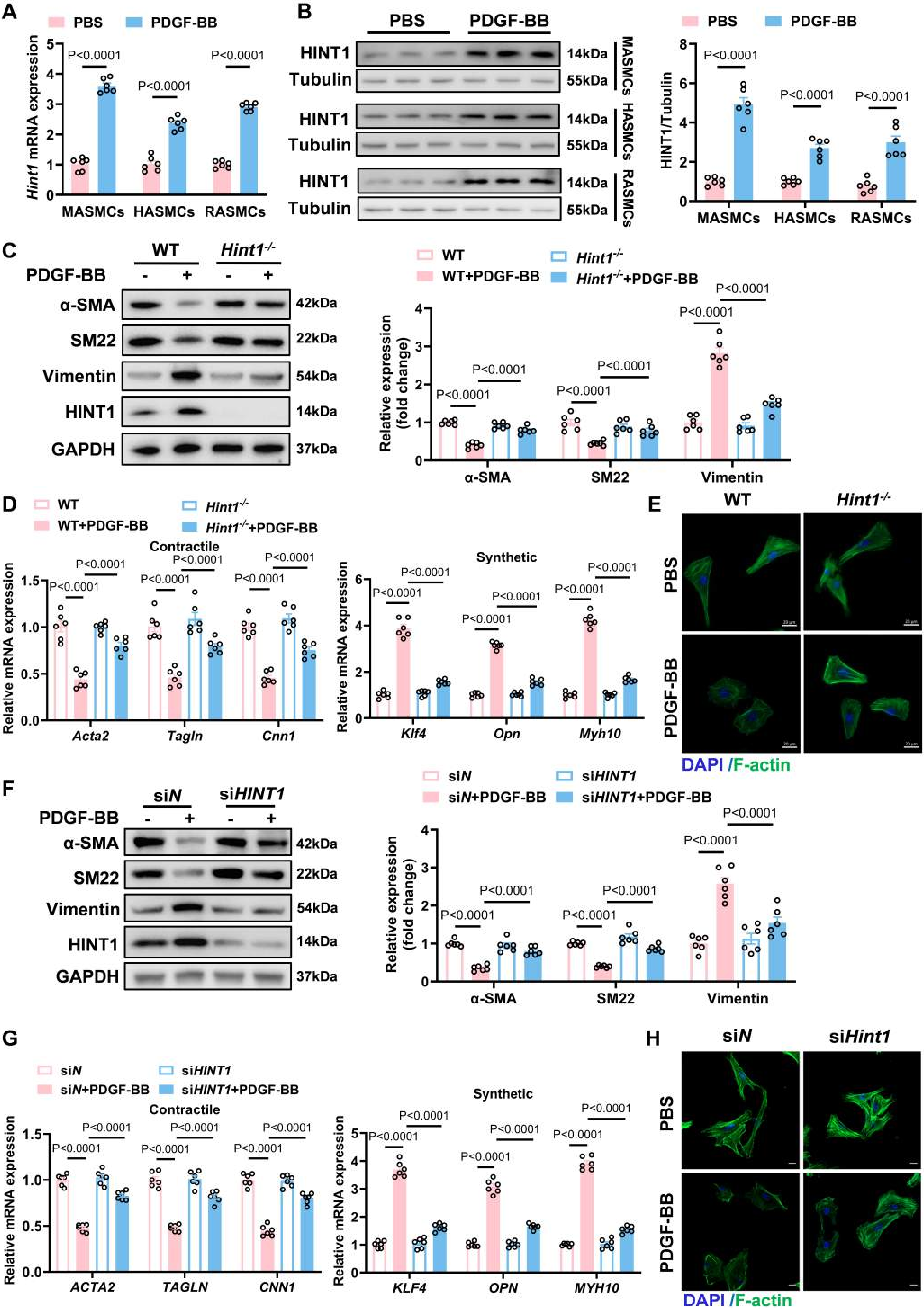
A
PBS
PDGF-BB
Hint1 mRNA expression
P<0.0001
MASMCs
HASMCs
RASMCs
B
PBS
PDGF-BB
HINT1
Tubulin
14kDa
55kDa
MASMCs
HASMCs
RASMCs
HINT1/Tubulin
P<0.0001
C
WT
Hint1-/-
PDGF-BB
α-SMA
SM22
Vimentin
HINT1
GAPDH
42kDa
22kDa
54kDa
14kDa
37kDa
WT
Hint1-/-
WT+PDGF-BB
Hint1-/-+PDGF-BB
Relative expression (fold change)
P<0.0001
α-SMA
SM22
Vimentin
D
WT
WT+PDGF-BB
Hint1-/-
Hint1-/-+PDGF-BB
Contractile
Synthetic
Relative mRNA expression
P<0.0001
Acta2
Tagln
Cnn1
Klf4
Opn
Myh10
E
WT
Hint1-/-
PBS
PDGF-BB
DAPI /F-actin
F
siN
siHINT1
PDGF-BB
α-SMA
SM22
Vimentin
HINT1
GAPDH
42kDa
22kDa
54kDa
14kDa
37kDa
siN
siN+PDGF-BB
siHINT1
siHINT1+PDGF-BB
Relative expression (fold change)
P<0.0001
α-SMA
SM22
Vimentin
G
siN
siN+PDGF-BB
siHINT1
siHINT1+PDGF-BB
Contractile
Synthetic
Relative mRNA expression
P<0.0001
ACTA2
TAGLN
CNN1
KLF4
OPN
MYH10
H
siN
siHint1
PBS
PDGF-BB
DAPI /F-actin

Supplemental Figure 3: HINT1 promotes vascular smooth muscle cell phenotypic switching.

(**A**), MASMCs were isolated from the whole aortas of mice. qPCR analysis of the mRNA levels of *Hint1* in MASMCs, HASMCs and RASMCs treated with PBS or platelet-derived growth factor BB (PDGF-BB; 20 ng/ml). n=6 per group. (**B**), MASMCs were isolated from the whole aortas of mice. Western blotting analysis of HINT1 in MASMCs, HASMCs or RASMCs with PBS or PDGF-BB (20 ng/ml). n=6 per group. (**C**), Western blotting analysis of VSMC contractile markers (α-SMA and SM22) and synthetic markers (Vimentin) in WT or *Hint1*$^{-/-}$ MASMCs treated with PBS or PDGF-BB (20 ng/ml). n=6 per group. (**D**), qPCR analysis of the mRNA levels of VSMC contractile markers (*Acta2, Tagln and Cnn1*) and synthetic markers (*Klf4, Opn and Myh10*) in MASMCs isolated from the whole aorta of WT and *Hint1*$^{-/-}$ mice and treated with PBS or PDGF-BB (20 ng/ml). n=6 per group. (**E**), Representative immunofluorescence images of F-actin (green) stained with phalloidin in MASMCs isolated from the whole aorta of WT and *Hint1*$^{-/-}$ mice and treated with PBS or PDGF-BB (20 ng/ml). (**F**), Western blotting analysis of VSMC contractile markers (αSMA and SM22) and synthetic markers (Vimentin) in HASMCs that transfected with siRNA expressing negative control (si*N*) or siRNA targeting *HINT1* (si*HINT1*) followed with PBS or PDGF-BB (20 ng/ml) stimulation. n=6 per group. (**G**), qPCR analysis of the mRNA levels of VSMC contractile markers (*ACTA2, TAGLN and CNN1*) and synthetic markers (*KLF4, OPN and MYH10*) in HASMCs transfected with si*N* or si*HINT1* followed by PBS or PDGF-BB (20 ng/ml) stimulation. n=6 per group. (**H**), Representative immunofluorescence images of F-actin (green) stained with phalloidin in RASMCs that transfected with si*N* or si*Hint1* followed with PBS or PDGF-BB (20 ng/ml) stimulation; scale bar = 20 μm. Statistical analysis was performed by Student *t* test for (**A and B**), One-way ANOVA for (**C, D, F and G**). For all statistical plots, the data are presented as mean ± SEM.

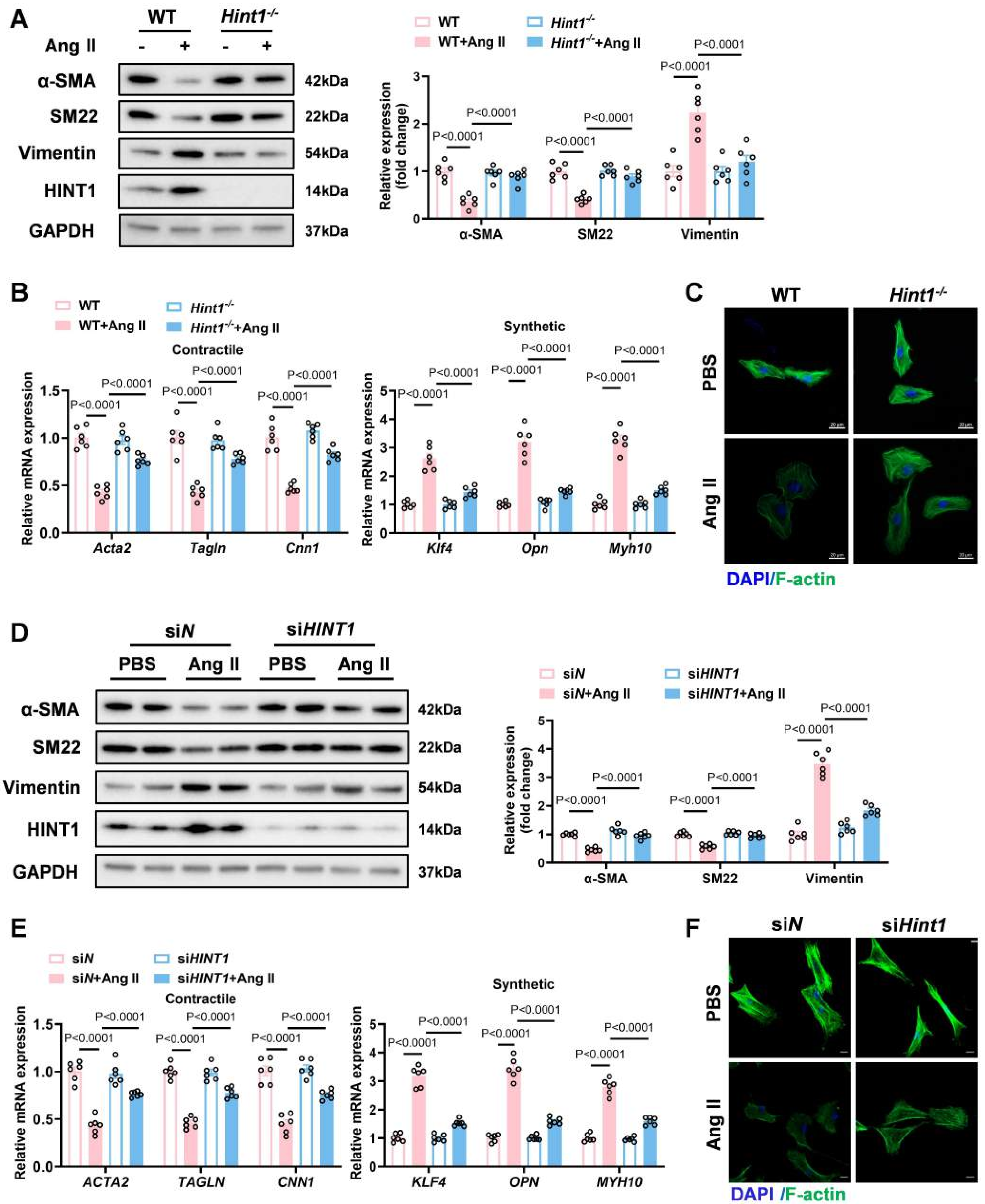
A
WT
Hint1-/-
Ang II
- + - +
α-SMA
SM22
Vimentin
HINT1
GAPDH
42kDa
22kDa
54kDa
14kDa
37kDa
WT
WT+Ang II
Hint1-/-
Hint1-/-+Ang II
Relative expression (fold change)
P<0.0001
α-SMA
SM22
Vimentin
B
Contractile
Synthetic
Relative mRNA expression
Acta2
Tagln
Cnn1
Klf4
Opn
Myh10
C
PBS
Ang II
DAPI/F-actin
D
siN
siHINT1
PBS
Ang II
siN+Ang II
siHINT1+Ang II
E
ACTA2
TAGLN
CNN1
KLF4
OPN
MYH10
F
siHint1
DAPI /F-actin

Supplemental Figure 4: HINT1 promotes Ang II-induced vascular smooth muscle cell phenotypic switching.

(**A**), Western blotting analysis of VSMC contractile markers (α-SMA and SM22) and synthetic markers (Vimentin) in MASMCs isolated from the whole aorta of WT and *Hint1*$^{-/-}$ mice and treated with PBS or Ang II (10^{-6} M). n=6 per group. (**B**), qPCR analysis of the mRNA levels of VSMC contractile markers (*Acta2*, *Cnn1*, and *Tagln*) and synthetic markers (*Klf4*, *Opn* and *Myh10*) in MASMCs isolated from the whole aorta of WT and *Hint1*$^{-/-}$ mice and treated with PBS or Ang II (10^{-6} M). n=6 per group. (**C**), Representative immunofluorescence images of F-actin (green) stained with phalloidin in MASMCs isolated from the whole aorta of WT and *Hint1*$^{-/-}$ mice and treated with PBS or Ang II (10^{-6} M). (**D**), Western blotting analysis of VSMC contractile markers (α-SMA and SM22) and synthetic markers (Vimentin) in HASMCs that transfected with siRNA expressing negative control (si*N*) or siRNA targeting HINT1 (si*HINT1*) followed with PBS or Ang II (10^{-6} M) stimulation. n=6 per group. (**E**), qPCR analysis of the mRNA levels of VSMC contractile markers (*ACTA2*, *CNN1* and *TAGLN*) and synthetic markers (*KLF4*, *OPN* and *MYH10*) in HASMCs transfected with si*N* or si*HINT1* followed by PBS or Ang II (10^{-6} M) stimulation. n=6 per group. (**F**), Representative immunofluorescence images of F-actin (green) stained with phalloidin in RASMCs that transfected with si*N* or si*Hint1* followed with PBS or Ang II (10^{-6} M) stimulation; scale bar=20 μm. Statistical analysis was performed by One-way ANOVA for (**A, B, D and E**). For all statistical plots, the data are presented as mean ± SEM.

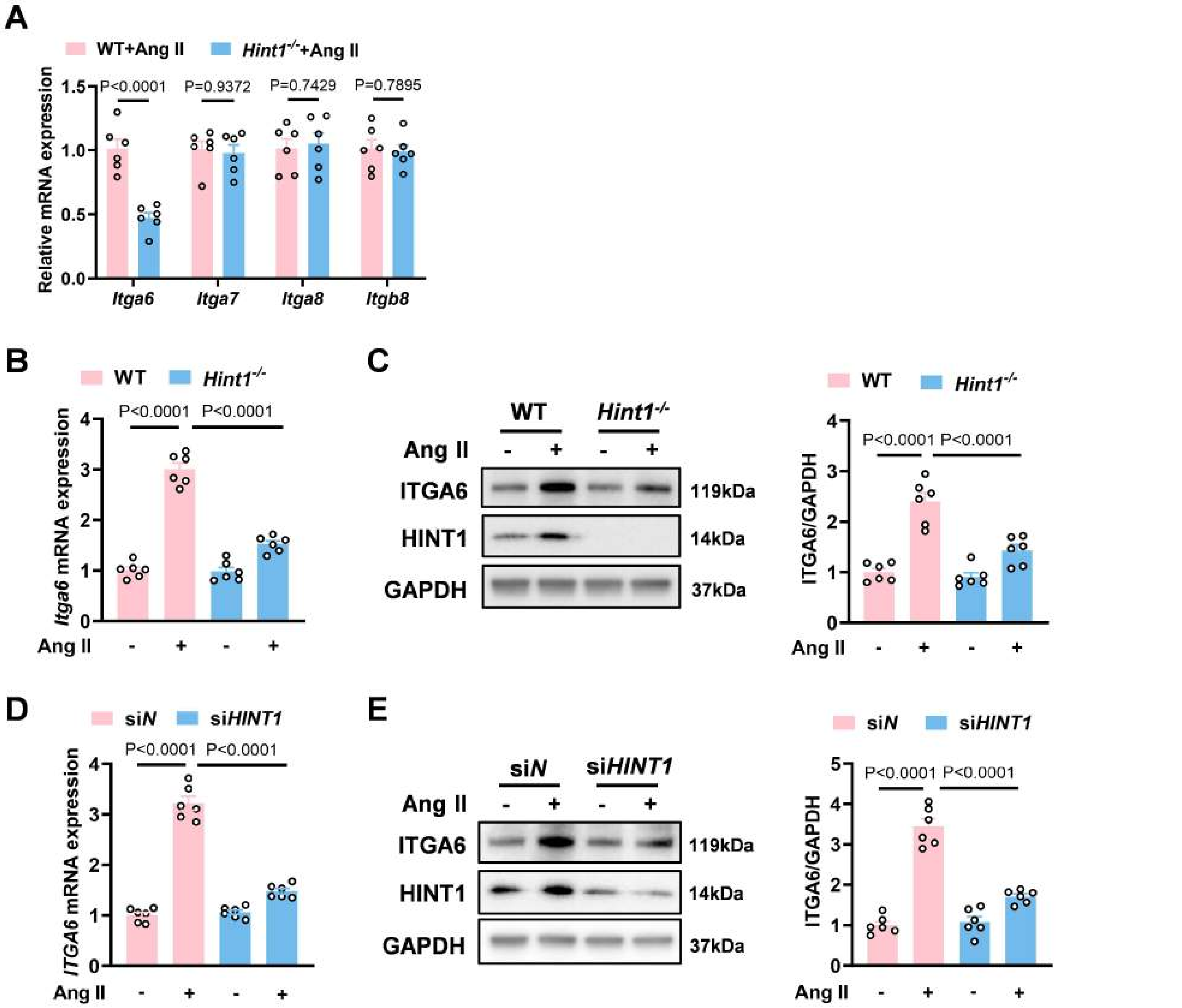

Supplemental Figure 5

(**A**), qPCR analysis of *Itga6*, *Itga7*, *Itga8*, *Itgb8* in MASMCs isolated from the whole aorta of WT and *Hint1*$^{-/-}$ mice and treated with Ang II (10^{-6} M). n=6 per group. (**B**), qPCR analysis of *Itga6* in MASMCs isolated from the whole aorta of WT and *Hint1*$^{-/-}$ mice and treated with PBS or Ang II (10^{-6} M). n=6 per group. (**C**), Western blotting analysis of ITGA6 in MASMCs isolated from the whole aorta of WT and *Hint1*$^{-/-}$ mice and treated with PBS or Ang II (10^{-6} M). n=6 per group. (**D**), qPCR analysis of *ITGA6* in HASMCs that transfected with si*N* or si*HINT1* followed by PBS or Ang II (10^{-6} M) stimulation. n=6 per group. (**E**), Western blotting analysis of ITGA6 in HASMCs that transfected with si*N* or si*HINT1* followed by PBS or Ang II (10^{-6} M) stimulation. n=6 per group. Statistical analysis was performed by Student t test for (**A**), One-way ANOVA for (**B through E**). For all statistical plots, the data are presented as mean ± SEM.

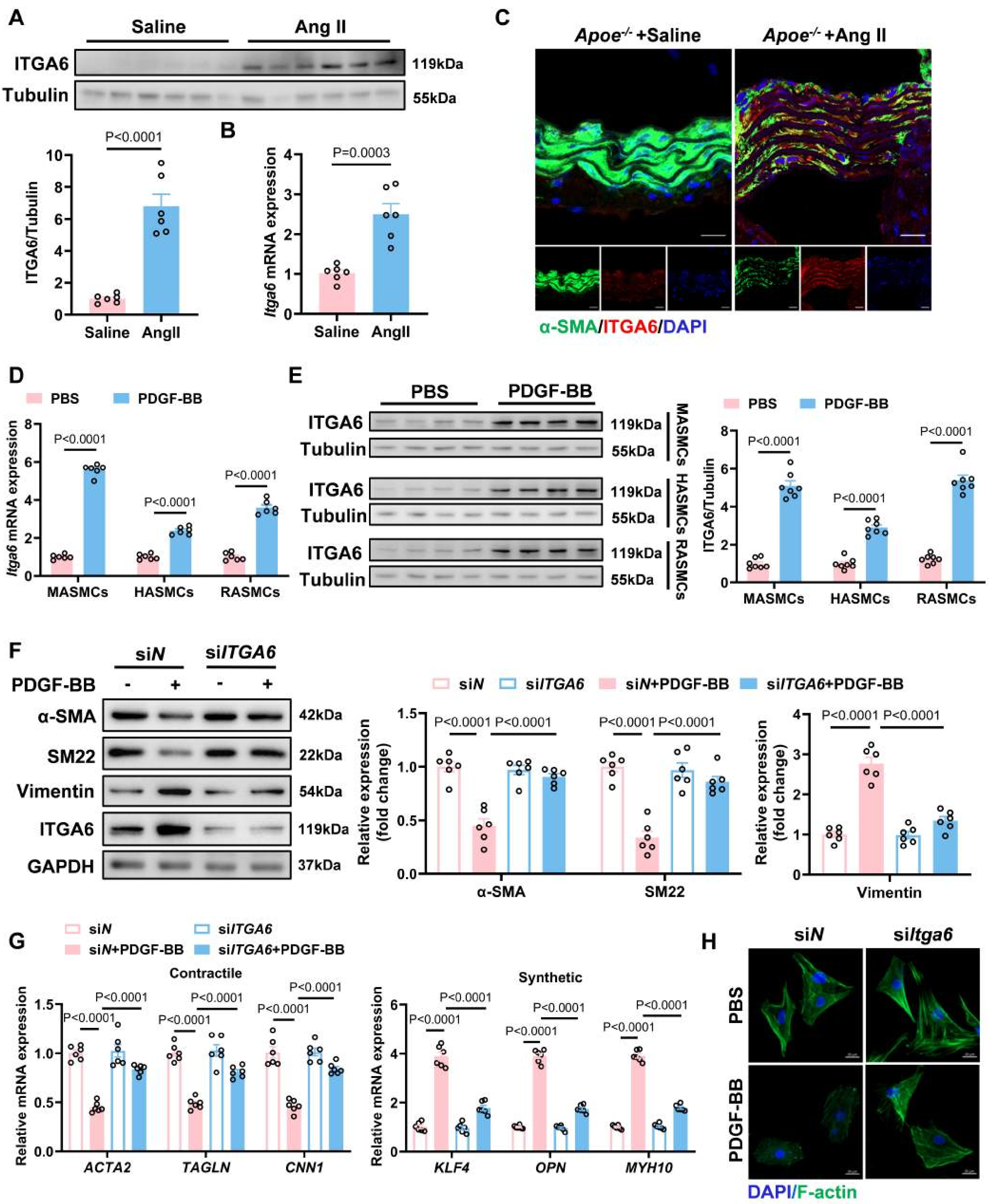

A
Saline
Ang II
ITGA6
Tubulin
119kDa
55kDa
P<0.0001
ITGA6/Tubulin
Saline
AngII
B
P=0.0003
Itga6 mRNA expression
Saline
AngII
C
Apoe-/- +Saline
Apoe-/- +Ang II
α-SMA/ITGA6/DAPI
D
PBS
PDGF-BB
Itga6 mRNA expression
P<0.0001
MASMCs
HASMCs
RASMCs
E
PBS
PDGF-BB
ITGA6
Tubulin
119kDa
55kDa
MASMCs
HASMCs
RASMCs
ITGA6/Tubulin
P<0.0001
F
siN
siITGA6
PDGF-BB
α-SMA
SM22
Vimentin
ITGA6
GAPDH
42kDa
22kDa
54kDa
119kDa
37kDa
siN
siITGA6
siN+PDGF-BB
siITGA6+PDGF-BB
Relative expression (fold change)
P<0.0001
α-SMA
SM22
Vimentin
G
siN
siITGA6
siN+PDGF-BB
siITGA6+PDGF-BB
Contractile
Synthetic
Relative mRNA expression
P<0.0001
ACTA2
TAGLN
CNN1
KLF4
OPN
MYH10
H
siN
siItga6
PBS
PDGF-BB
DAPI/F-actin

Supplemental Figure 6: ITGA6 promotes vascular smooth muscle cell phenotypic switching. (**A through C**), Eight-week-old male *Apoe*$^{-/-}$ mice were infused with saline or angiotensin II (Ang II; 1000 ng/kg/min) for 28 days. Western blotting (**A**) and qPCR (**B**) analysis of ITGA6 expression in suprarenal abdominal aortas. n=6 mice per group. (**C**), Representative immunofluorescence images of α-SMA and ITGA6 in suprarenal abdominal aortas; scale bar = 20 μm. (**D and E**), MASMCs were isolated from the whole aortas of mice. qPCR (**D**) and Western blotting (**E**) analysis of *Itga6* in MASMCs, HASMCs and RASMCs treated with PBS or PDGF-BB (20 ng/ml). n=6-7 per group. (**F**), Western blotting analysis of VSMC contractile markers (α-SMA and SM22) and synthetic markers (Vimentin) in HASMCs that transfected with siRNA expressing negative control (si*N*) or siRNA targeting *ITGA6* (si*ITGA6*) followed by PBS or PDGF-BB (20 ng/ml) stimulation. n=6 per group. (**G**), qPCR analysis of the mRNA levels of VSMC contractile markers (*ACTA2, TAGLN and CNN1*) and synthetic markers (*KLF4, OPN and MYH10*) in HASMCs that transfected with si*N* or si*ITGA6* followed by PBS or PDGF-BB (20 ng/ml) stimulation. n=6 per group. (**H**), Representative immunofluorescence images of F-actin (green) stained with phalloidin in RASMCs that transfected with si*N* or si*Itga6* followed by PBS or PDGF-BB (20 ng/ml) stimulation; scale bar = 20 μm. Statistical analysis was performed by Student *t* test for (**A, B, D and E**), One-way ANOVA for (**F and G**). For all statistical plots, the data are presented as mean ± SEM.

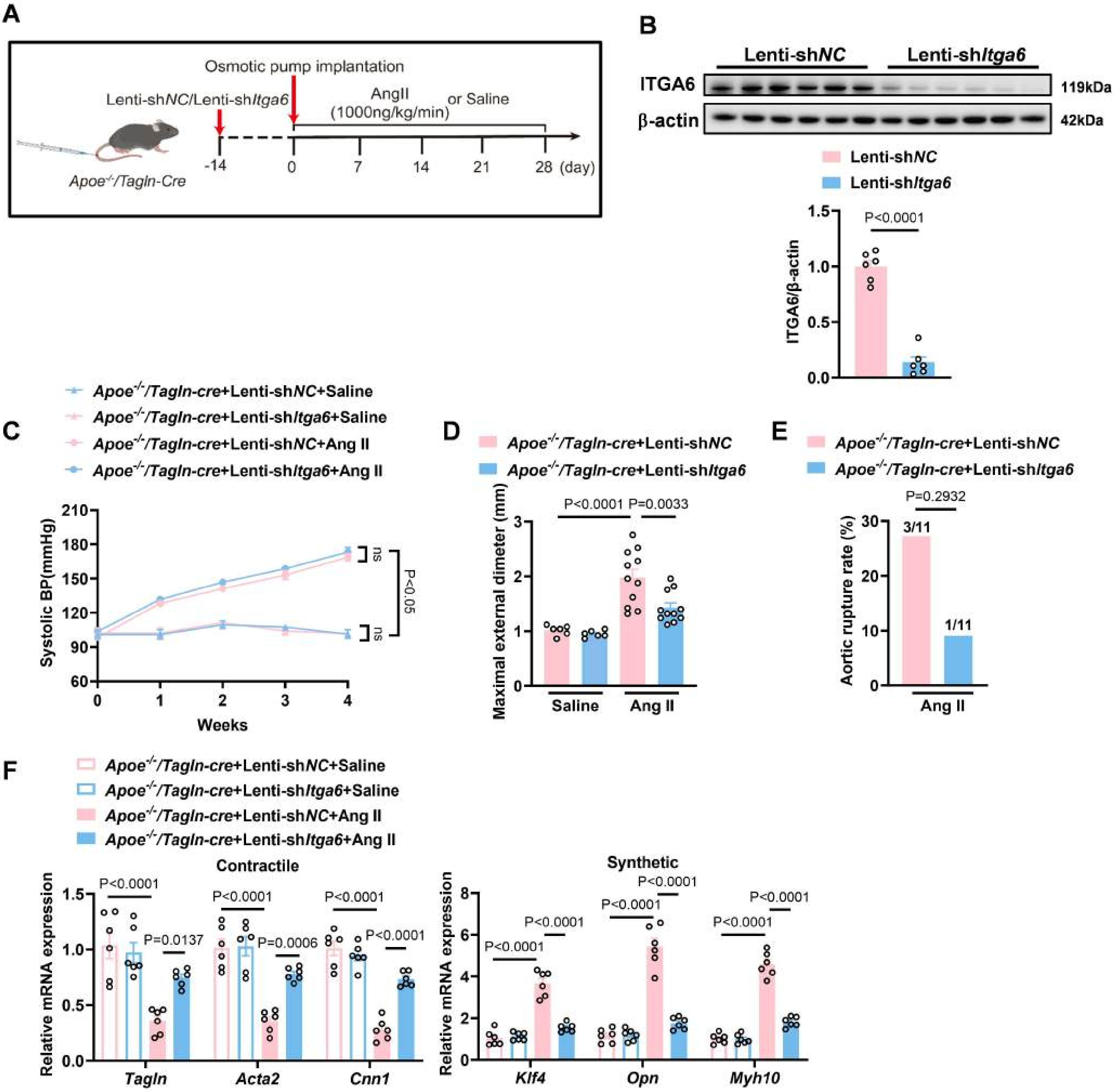
A
Osmotic pump implantation
Lenti-shNC/Lenti-shItga6
AngII
(1000ng/kg/min)
or Saline
Apoe-/-/Tagln-Cre
-14
0
7
14
21
28 (day)
B
Lenti-shNC
Lenti-shItga6
ITGA6
β-actin
119kDa
42kDa
ITGA6/β-actin
P<0.0001
C
Apoe-/-/Tagln-cre+Lenti-shNC+Saline
Apoe-/-/Tagln-cre+Lenti-shItga6+Saline
Apoe-/-/Tagln-cre+Lenti-shNC+Ang II
Apoe-/-/Tagln-cre+Lenti-shItga6+Ang II
Systolic BP(mmHg)
Weeks
ns
P<0.05
D
Apoe-/-/Tagln-cre+Lenti-shNC
Apoe-/-/Tagln-cre+Lenti-shItga6
Maximal external dimeter (mm)
P<0.0001
P=0.0033
Saline
Ang II
E
Aortic rupture rate (%)
P=0.2932
3/11
1/11
Ang II
F
Apoe-/-/Tagln-cre+Lenti-shNC+Saline
Apoe-/-/Tagln-cre+Lenti-shItga6+Saline
Apoe-/-/Tagln-cre+Lenti-shNC+Ang II
Apoe-/-/Tagln-cre+Lenti-shItga6+Ang II
Contractile
Synthetic
Relative mRNA expression
P=0.0137
P=0.0006
Tagln
Acta2
Cnn1
Klf4
Opn
Myh10

Supplemental Figure 7: Downregulation of *Itga6* in vascular smooth muscle cells mitigates aortic aneurysm.

(**A**), Experimental design. Six-week-old male *Apoe*$^{-/-}$/*Tagln-cre* mice were injected with lentivirus vector encoding negative shRNA control (Lenti-sh*NC*) or lentivirus vector encoding shRNA targeting *Itga6* (Lenti-*shItga6*) with 2 reverse loxP sites, which can be recognized by Cre recombinase. After injection for 14 days, mice were infused with saline or angiotensin II (Ang II; 1000 ng/kg/min) for 28 days. (**B**), Western blotting analysis of ITGA6 expression in aortas from saline-infused *Apoe*$^{-/-}$/*Tagln-cre* mice infected with Lenti-sh*NC* or Lenti-sh*Itga6*. n=6 per group. (**C**), Systolic blood pressure at 0, 1, 2, 3 and 4 weeks for saline or Ang II-infused *Apoe*$^{-/-}$/*Tagln-cre* mice infected with Lenti-sh*NC* or Lenti-sh*Itga6*. (**D**), Maximum abdominal aortic diameters were assessed by measuring external aortic diameter from images. (**E**), The aortic rupture rate in Ang II-infused mice. (**F**), qPCR analysis of the mRNA levels of VSMC contractile markers (*Acta2, Tagln and Cnn1*) and synthetic markers (*Klf4, Opn and Myh10*) in suprarenal abdominal aortas from saline or Ang II-infused *Apoe*$^{-/-}$/*Tagln cre* mice injected Lenti-sh*NC* or Lenti-sh*Itga6*. n=6 per group. Statistical analysis was performed by Student *t* test for (**B**), Two-way ANOVA with mixed-effects analysis for (**C**), One-way ANOVA for (**D and F**), Fisher exact test for (**E**). For all statistical plots, the data are presented as mean ± SEM. ns, no significance.

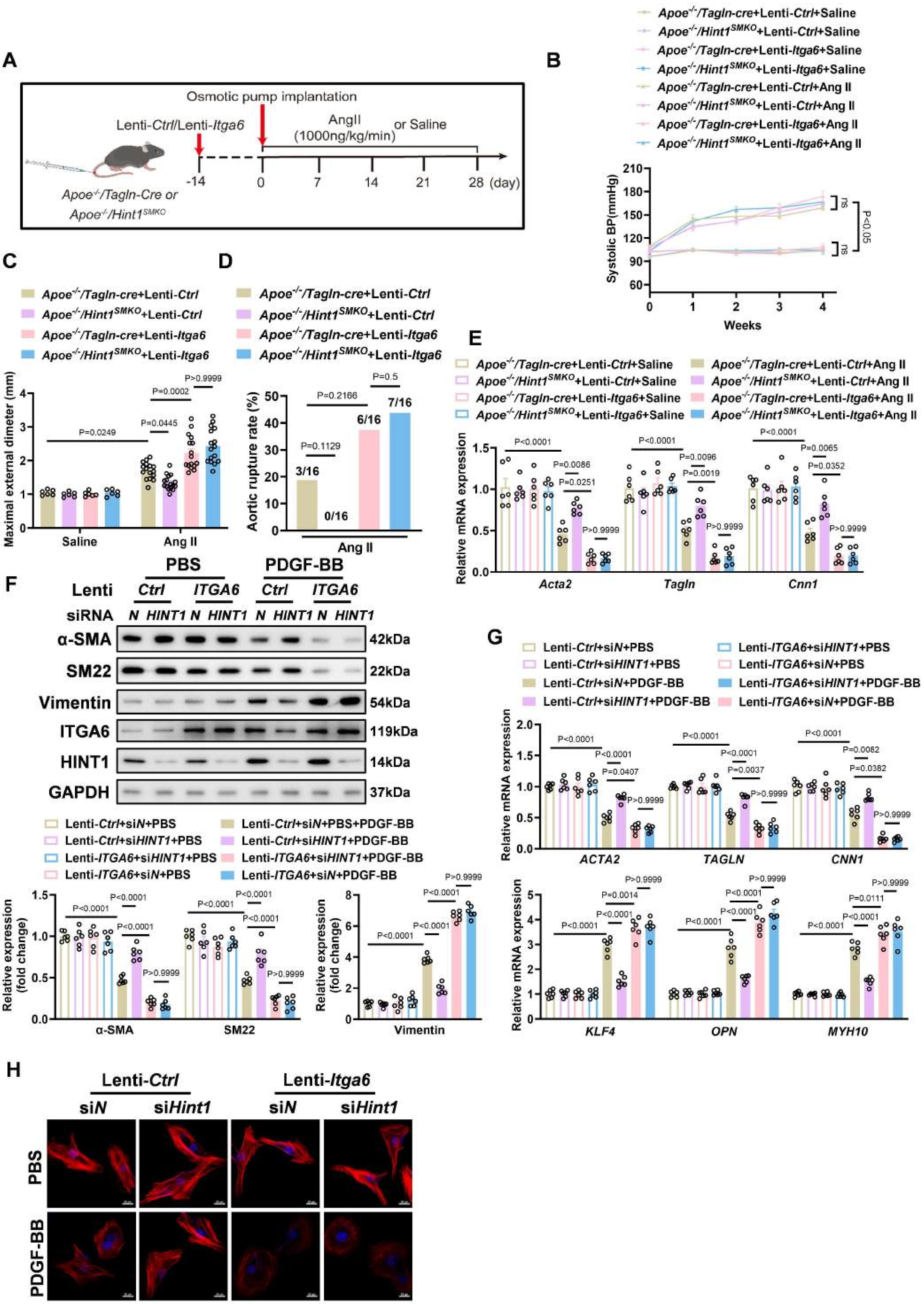
A
Osmotic pump implantation
Lenti-Ctrl/Lenti-Itga6
AngII (1000ng/kg/min) or Saline
-14 0 7 14 21 28 (day)
Apoe-/-/Tagln-Cre or Apoe-/-/Hint1SMKO
B
Apoe-/-/Tagln-cre+Lenti-Ctrl+Saline
Apoe-/-/Hint1SMKO+Lenti-Ctrl+Saline
Apoe-/-/Tagln-cre+Lenti-Itga6+Saline
Apoe-/-/Hint1SMKO+Lenti-Itga6+Saline
Apoe-/-/Tagln-cre+Lenti-Ctrl+Ang II
Apoe-/-/Hint1SMKO+Lenti-Ctrl+Ang II
Apoe-/-/Tagln-cre+Lenti-Itga6+Ang II
Apoe-/-/Hint1SMKO+Lenti-Itga6+Ang II
Systolic BP(mmHg)
Weeks
ns
P<0.05
C
Maximal external dimeter (mm)
Saline
Ang II
P=0.0249
P=0.0445
P=0.0002
P>0.9999
D
Aortic rupture rate (%)
3/16 0/16 6/16 7/16
P=0.1129
P=0.2166
P=0.5
E
Relative mRNA expression
Acta2 Tagln Cnn1
F
PBS PDGF-BB
Lenti Ctrl ITGA6 Ctrl ITGA6
siRNA N HINT1 N HINT1 N HINT1 N HINT1
α-SMA 42kDa
SM22 22kDa
Vimentin 54kDa
ITGA6 119kDa
HINT1 14kDa
GAPDH 37kDa
Lenti-Ctrl+siN+PBS
Lenti-Ctrl+siHINT1+PBS
Lenti-ITGA6+siHINT1+PBS
Lenti-ITGA6+siN+PBS
Lenti-Ctrl+siN+PBS+PDGF-BB
Lenti-Ctrl+siHINT1+PDGF-BB
Lenti-ITGA6+siHINT1+PDGF-BB
Lenti-ITGA6+siN+PDGF-BB
Relative expression (fold change)
α-SMA SM22 Vimentin
G
Lenti-Ctrl+siN+PBS
Lenti-Ctrl+siHINT1+PBS
Lenti-Ctrl+siN+PDGF-BB
Lenti-Ctrl+siHINT1+PDGF-BB
Lenti-ITGA6+siHINT1+PBS
Lenti-ITGA6+siN+PBS
Lenti-ITGA6+siHINT1+PDGF-BB
Lenti-ITGA6+siN+PDGF-BB
Relative mRNA expression
ACTA2 TAGLN CNN1
KLF4 OPN MYH10
H
Lenti-Ctrl Lenti-Itga6
siN siHint1 siN siHint1
PBS
PDGF-BB

Supplemental figure 8: Impact of HINT1 on aortic aneurysm depends on ITGA6.

(**A**), Experimental design. Six-week-old male *Apoe*$^{-/-}$/*Tagln-cre* and *Apoe*$^{-/-}$/*Hint1*^{SMKO} mice were injected with lentivirus vectors encoding control (Lenti-*Ctrl*) or *Itga6* (Lenti-*Itga6*) with 2 reverse loxP sites, which can be recognized by Cre recombinase. After injection for 14 days, mice were infused with saline or angiotensin II (Ang II; 1000 ng/kg/min) for 28 days. (**B**), Systolic blood pressure at 0, 1, 2, 3 and 4 weeks of saline or Ang II-infused *Apoe*$^{-/-}$/*Tagln-cre* or *Apoe*$^{-/-}$/*Hint1*^{SMKO} mice infected with Lenti-*Ctrl* or Lenti-*Itga6*. (**C**), Maximum abdominal aortic diameters were assessed by measuring external aortic diameter from images. (**D**), The aortic rupture rate in Ang II-infused mice. (**E**), qPCR analysis of the mRNA levels of VSMC contractile markers (*Acta2, Tagln and Cnn1*) in suprarenal abdominal aortas from saline or Ang II-infused *Apoe*$^{-/-}$/*Tagln-cre* or *Apoe*$^{-/-}$/*Hint1*^{SMKO} mice infected with Lenti-*Ctrl* or Lenti-*Itga6*. n=6 per group. (**F**), Western blotting analysis of VSMC contractile markers (α-SMA and SM22) and synthetic markers (Vimentin) in HASMCs that infected with Lenti-*Ctrl* or Lenti-*ITGA6* and transfected with si*N* or si*HINT1* followed by PBS or PDGF-BB (20 ng/ml) stimulation. n=6 per group. (**G**), qPCR analysis of the mRNA levels of VSMC contractile markers (*ACTA2, TAGLN and CNN1*) and synthetic markers (*KLF4, OPN and MYH10*) in HASMCs that infected with Lenti-*Ctrl* or Lenti-*ITGA6* and transfected with si*N* or si*HINT1* followed by PBS or PDGF-BB (20 ng/ml) stimulation. n=6 per group. (**H**), Representative immunofluorescence images of F-actin (red) stained with phalloidin in RASMCs that infected with Lenti-*Ctrl* or Lenti-*Itga6* and transfected with si*N* or si*Hint1* followed by PBS or PDGF-BB (20 ng/ml) stimulation; scale bar = 20μm. Statistical analysis was performed by Two-way ANOVA with mixed-effects analysis for (**B**), Fisher exact test for (**D**), Two-way ANOVA for (**C, E through G**). For all statistical plots, the data are presented as mean ± SEM. ns, no significance.

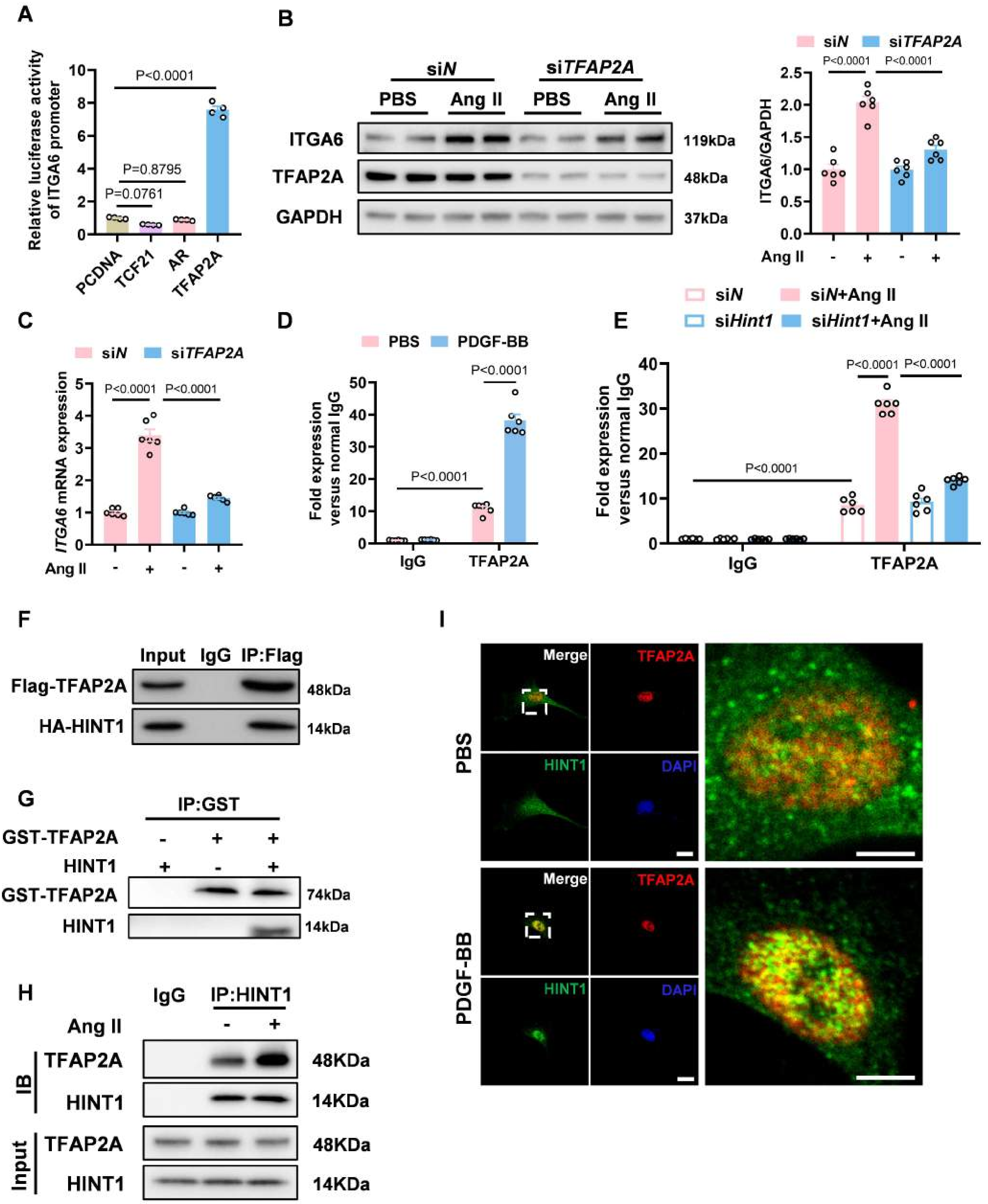
A
Relative luciferase activity of ITGA6 promoter
P<0.0001
P=0.8795
P=0.0761
PCDNA
TCF21
AR
TFAP2A
B
siN
siTFAP2A
PBS
Ang II
PBS
Ang II
ITGA6
TFAP2A
GAPDH
119kDa
48kDa
37kDa
siN
siTFAP2A
P<0.0001
P<0.0001
ITGA6/GAPDH
Ang II
C
siN
siTFAP2A
ITGA6 mRNA expression
P<0.0001
P<0.0001
Ang II
D
PBS
PDGF-BB
Fold expression versus normal IgG
P<0.0001
P<0.0001
IgG
TFAP2A
E
siN
siN+Ang II
siHint1
siHint1+Ang II
Fold expression versus normal IgG
P<0.0001
P<0.0001
P<0.0001
IgG
TFAP2A
F
Input
IgG
IP:Flag
Flag-TFAP2A
HA-HINT1
48kDa
14kDa
G
IP:GST
GST-TFAP2A
HINT1
GST-TFAP2A
HINT1
74kDa
14kDa
H
IgG
IP:HINT1
Ang II
IB
TFAP2A
HINT1
Input
TFAP2A
HINT1
48KDa
14KDa
48KDa
14KDa
I
PBS
PDGF-BB
Merge
TFAP2A
HINT1
DAPI

Supplemental Figure 9: HINT1 regulates ITGA6 expression via combining with TFAP2A.

(**A**), Luciferase reporter constructs with full-length *Itga6* promoter were co-transfected with plasmids of TFAP2A, TCF21, AR or PCDNA into HEK293T cells, and luciferase activity was evaluated and normalized to renilla luciferase. n=4 per group. (**B and C**), Western blotting (**B**) and qPCR (**C**) analysis of ITGA6 expression in HASMCs that were transfected with siRNA expressing negative control (si*N*) or siRNA targeting TFAP2A (si*TFAP2A)* followed by PBS or Ang II (10^{-6} M) stimulation. n=6 per group. (**D**), Chromatin immunoprecipitation assays of TFAP2A binding to the ITGA6 promoter in RASMCs with PBS or PDGF-BB (20 ng/ml) stimulation. n=6 per group. (**E**), Chromatin immunoprecipitation assays of TFAP2A binding to the ITGA6 promoter in RASMCs transfected with si*N* or si*Hint1* and treated with PBS or Ang II (10^{-6} M). n=6 per group. (**F**), HEK293T cells were co-transfected with Flag-TFAP2A and HA-HINT1 plasmids. Co-immunoprecipitation analysis of Flag-TFAP2A and HA-HINT1 interaction (immunoprecipitated by Flag antibody). (**G**), In vitro binding assay of purified HINT1 and GST-TFAP2A protein (immunoprecipitated by GST antibody). (**H**), Co-immunoprecipitation assay of HINT1 and TFAP2A interaction in HASMCs with PBS or Ang II (10^{-6} M) stimulation for 4h (immunoprecipitated by HINT1 antibody). (**I**), Confocal fluorescence microscopy of TFAP2A (red) and HINT1 (green) in HASMCs. DAPI, blue; scale bar=10 and 5μm. Statistical analysis was performed by One-way ANOVA for (**A through D**)), Two-way ANOVA for (**E**). For all statistical plots, the data are presented as mean ± SEM.

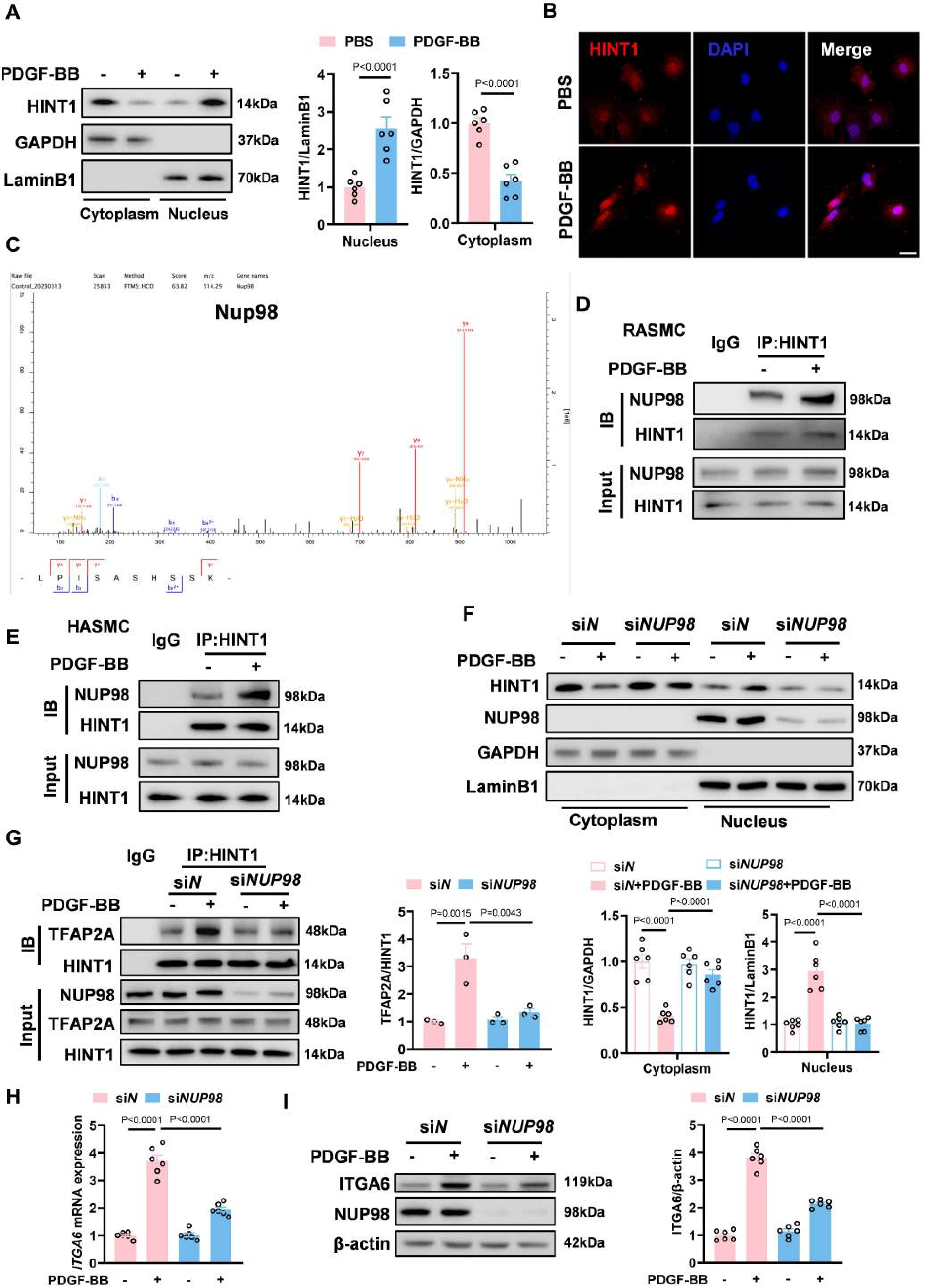

A
PDGF-BB - + - +
HINT1
GAPDH
LaminB1
14kDa
37kDa
70kDa
Cytoplasm Nucleus
PBS PDGF-BB
P<0.0001
HINT1/LaminB1
Nucleus
P<0.0001
HINT1/GAPDH
Cytoplasm
B
HINT1
DAPI
Merge
PBS
PDGF-BB
C
Nup98
D
RASMC
IgG
IP:HINT1
PDGF-BB - +
IB
Input
NUP98
HINT1
98kDa
14kDa
E
HASMC
IgG
IP:HINT1
PDGF-BB - +
IB
Input
NUP98
HINT1
98kDa
14kDa
F
siN siNUP98 siN siNUP98
PDGF-BB - + - + - + - +
HINT1
NUP98
GAPDH
LaminB1
14kDa
98kDa
37kDa
70kDa
Cytoplasm
Nucleus
G
IgG
IP:HINT1
siN siNUP98
PDGF-BB - + - +
IB
Input
TFAP2A
HINT1
NUP98
48kDa
14kDa
98kDa
siN siNUP98
P=0.0015 P=0.0043
TFAP2A/HINT1
PDGF-BB - + - +
siN siNUP98
siN+PDGF-BB siNUP98+PDGF-BB
P<0.0001 P<0.0001
HINT1/GAPDH
Cytoplasm
HINT1/LaminB1
Nucleus
H
siN siNUP98
P<0.0001 P<0.0001
ITGA6 mRNA expression
PDGF-BB - + - +
I
siN siNUP98
PDGF-BB - + - +
ITGA6
NUP98
β-actin
119kDa
98kDa
42kDa
siN siNUP98
P<0.0001 P<0.0001
ITGA6/β-actin
PDGF-BB - + - +

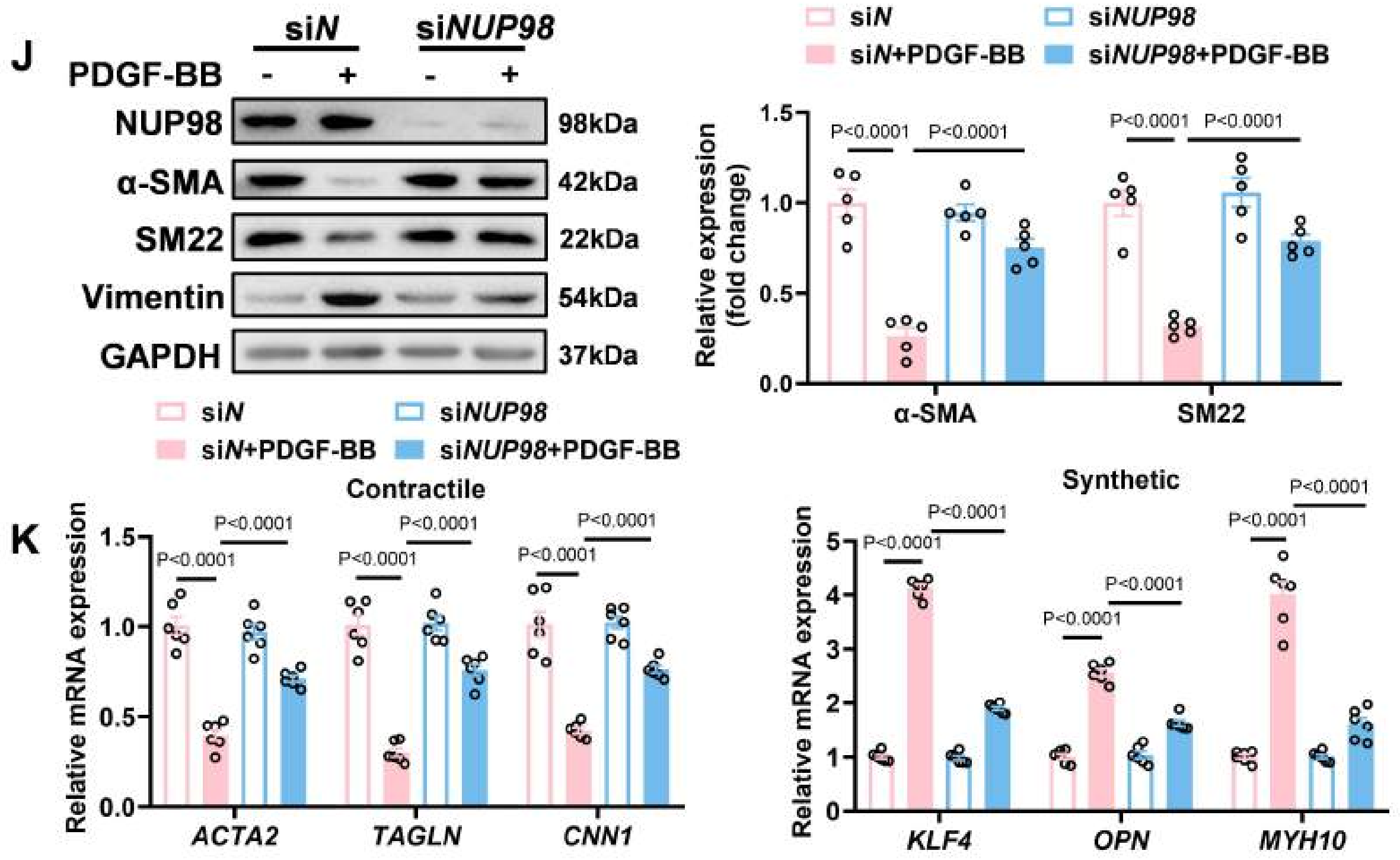

Supplemental Figure 10: HINT1 enhances the interaction with TFAP2A by its increased nuclear translocation under stimulation of PDGF-BB.

(**A**), Western blotting analysis of the nuclear translocation of HINT1 in HASMCs under PBS or PDGF-BB (20 ng/ml) stimulation. n=6 per group. (**B**), Representative confocal microscopy images showing the nuclear translocation of HINT1 (red) in HASMCs treated with PBS or PDGF-BB (20 ng/ml). DAPI, blue; scale bar = 20 μm. (**C**), NUP98 peptide fragmentation was detected by the liquid chromatography-tandem mass spectrometry (LC-MS/MS) analysis form the proteins immunoprecipitated with anti-HINT1 antibody. (**D**), Co-immunoprecipitation assay of HINT1 and TFAP2A interaction in RASMCs under PBS or PDGF-BB (20 ng/ml) stimulation (immunoprecipitated by HINT1 antibody). (**E**), Co-immunoprecipitation assay of HINT1 and TFAP2A interaction in HASMCs under PBS or PDGF-BB (20 ng/ml) stimulation (immunoprecipitated by HINT1 antibody). (**F**), Western blotting analysis of the nuclear translocation of HINT1 in HASMCs that transfected with si*N* or si*NUP98* followed by PBS or PDGF-BB (20 ng/ml) stimulation. n=6 per group. (**G**), Co-immunoprecipitation assay of HINT1 and TFAP2A interaction in HASMCs that transfected with si*N* or si*NUP98* followed by PBS or PDGF-BB (20 ng/ml) stimulation (immunoprecipitated by HINT1 antibody). n=3 per group. (**H and I**), qPCR (**H**) and western blotting (**I**) analysis of ITGA6 in HASMCs that transfected with si*N* or si*NUP98* followed by PBS or PDGF-BB (20 ng/ml) stimulation. n=6 per group. (**J**), Western blotting analysis of VSMC contractile markers (α-SMA and SM22) and synthetic markers (Vimentin) in HASMCs that transfected with si*N* or si*NUP98* followed by PBS or PDGF-BB (20 ng/ml) stimulation. n=5 per group. (**K**), qPCR analysis of the mRNA levels of VSMC contractile markers (*ACTA2, CNN1 and TAGLN*) and synthetic markers (*KLF4, OPN and MYH10*) in HASMCs that transfected with si*N* or si*NUP98* followed by PBS or PDGF-BB (20 ng/ml) stimulation. n=6 per group. Statistical analysis was performed by Student *t* test for (**A**), One-way ANOVA for (**F through K**). For all statistical plots, the data are presented as mean ± SEM.

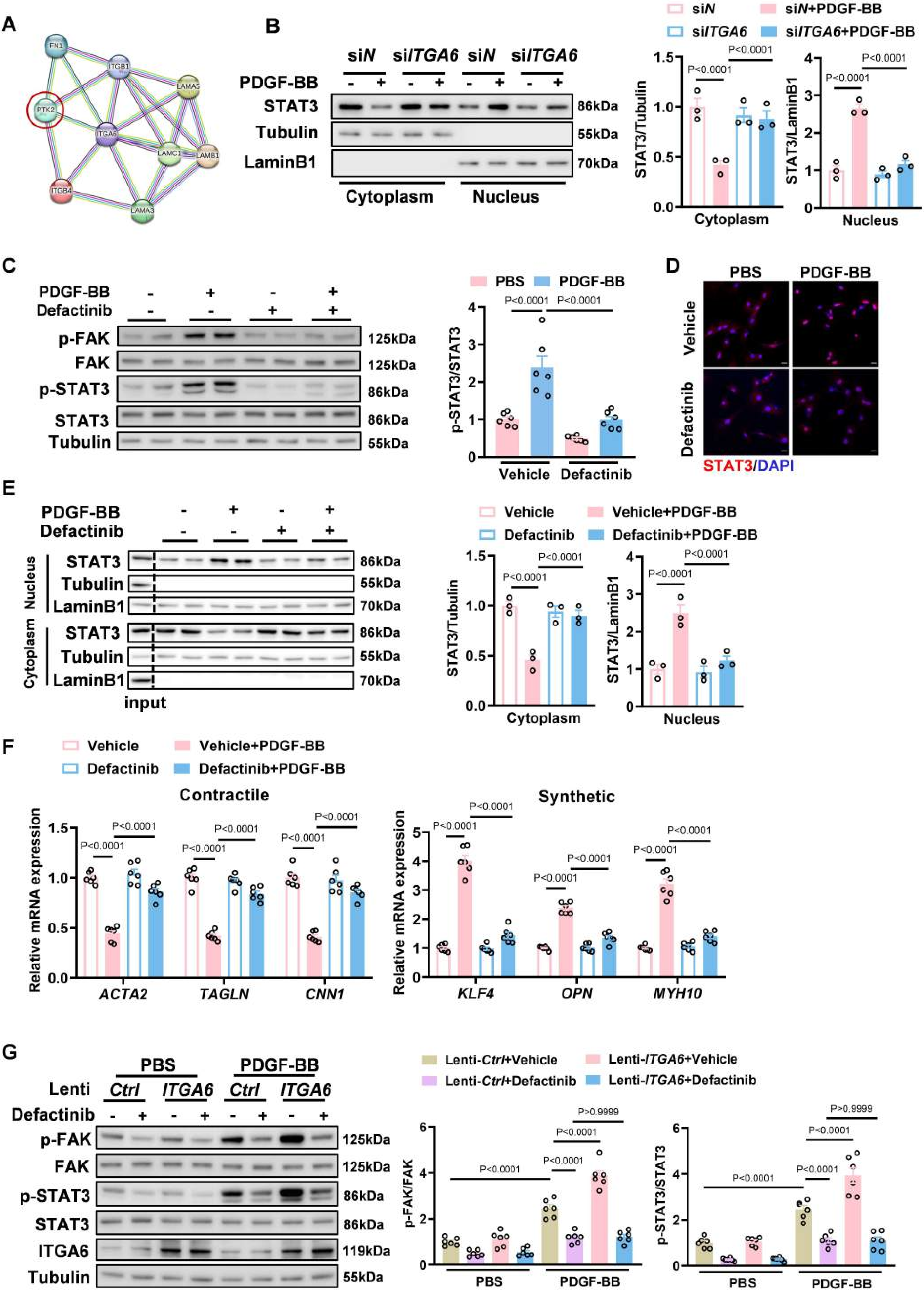
A
FN1
ITGB1
LAMA5
PTK2
ITGA6
LAMC1
LAMB1
ITGB4
LAMA3
B
siN siITGA6 siN siITGA6
PDGF-BB - + - + - + - +
STAT3 86kDa
Tubulin 55kDa
LaminB1 70kDa
Cytoplasm Nucleus
siN siN+PDGF-BB
siITGA6 siITGA6+PDGF-BB
STAT3/Tubulin
STAT3/LaminB1
P<0.0001
Cytoplasm
Nucleus
C
PDGF-BB - + - +
Defactinib - - + +
p-FAK 125kDa
FAK 125kDa
p-STAT3 86kDa
STAT3 86kDa
Tubulin 55kDa
PBS PDGF-BB
p-STAT3/STAT3
P<0.0001 P<0.0001
Vehicle Defactinib
D
PBS PDGF-BB
Vehicle
Defactinib
STAT3/DAPI
E
PDGF-BB - + - +
Defactinib - - + +
Nucleus
Cytoplasm
STAT3 86kDa
Tubulin 55kDa
LaminB1 70kDa
input
Vehicle Vehicle+PDGF-BB
Defactinib Defactinib+PDGF-BB
STAT3/Tubulin
STAT3/LaminB1
P<0.0001
Cytoplasm
Nucleus
F
Vehicle Vehicle+PDGF-BB
Defactinib Defactinib+PDGF-BB
Contractile
Synthetic
Relative mRNA expression
P<0.0001
ACTA2 TAGLN CNN1
KLF4 OPN MYH10
G
PBS PDGF-BB
Lenti Ctrl ITGA6 Ctrl ITGA6
Defactinib - + - + - + - +
p-FAK 125kDa
FAK 125kDa
p-STAT3 86kDa
STAT3 86kDa
ITGA6 119kDa
Tubulin 55kDa
Lenti-Ctrl+Vehicle Lenti-ITGA6+Vehicle
Lenti-Ctrl+Defactinib Lenti-ITGA6+Defactinib
p-FAK/FAK
p-STAT3/STAT3
P<0.0001
P>0.9999
PBS PDGF-BB

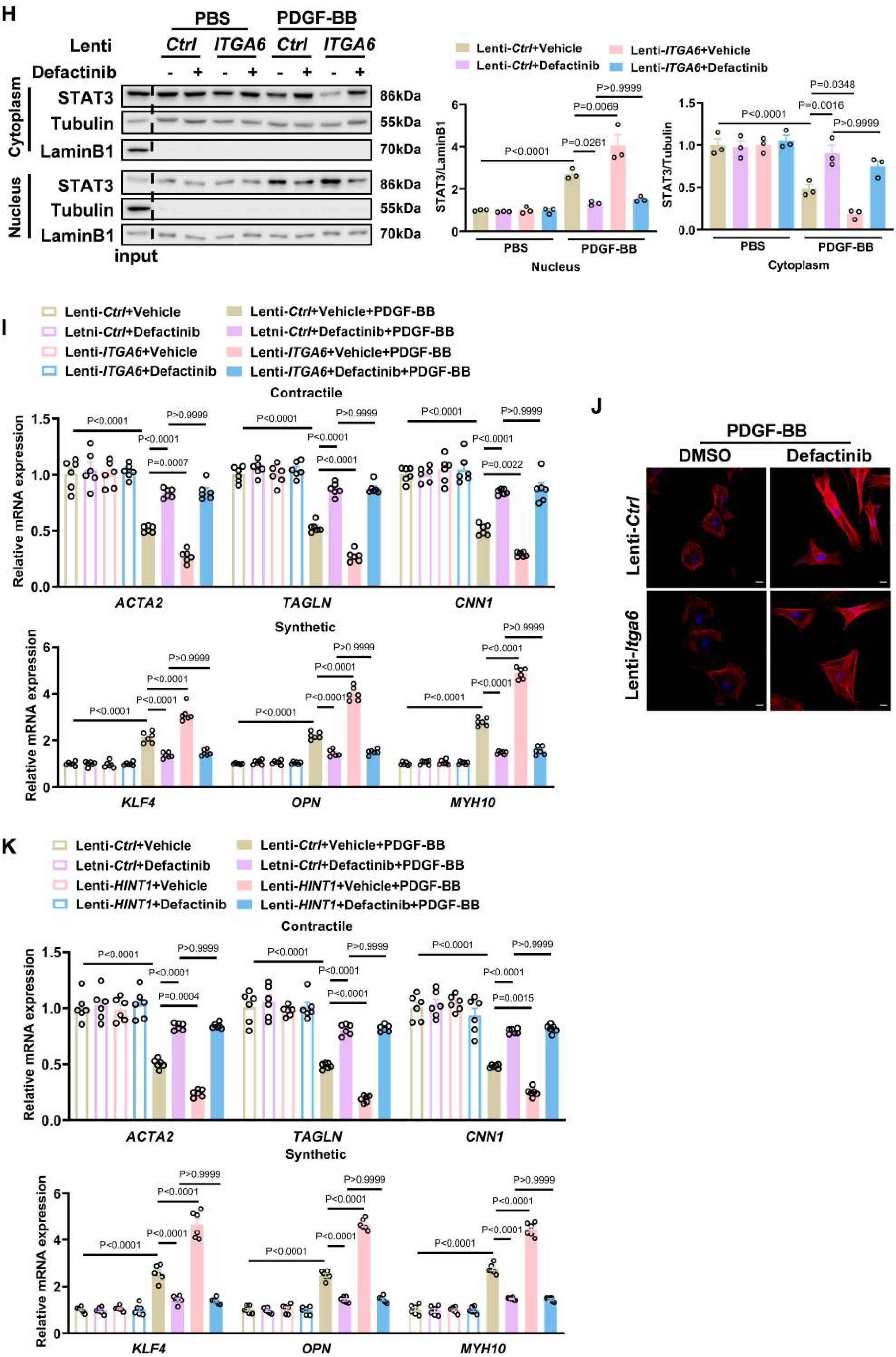
H
PBS
PDGF-BB
Lenti
Ctrl
ITGA6
Ctrl
ITGA6
Defactinib
Cytoplasm
STAT3
Tubulin
LaminB1
Nucleus
STAT3
Tubulin
LaminB1
input
86kDa
55kDa
70kDa
86kDa
55kDa
70kDa
Lenti-Ctrl+Vehicle
Lenti-Ctrl+Defactinib
Lenti-ITGA6+Vehicle
Lenti-ITGA6+Defactinib
STAT3/LaminB1
P<0.0001
P=0.0261
P=0.0069
P>0.9999
PBS
PDGF-BB
Nucleus
STAT3/Tubulin
P<0.0001
P=0.0016
P=0.0348
P>0.9999
PBS
PDGF-BB
Cytoplasm
I
Lenti-Ctrl+Vehicle
Letni-Ctrl+Defactinib
Lenti-ITGA6+Vehicle
Lenti-ITGA6+Defactinib
Lenti-Ctrl+Vehicle+PDGF-BB
Letni-Ctrl+Defactinib+PDGF-BB
Lenti-ITGA6+Vehicle+PDGF-BB
Lenti-ITGA6+Defactinib+PDGF-BB
Contractile
Relative mRNA expression
P<0.0001
P<0.0001
P=0.0007
P>0.9999
P=0.0022
ACTA2
TAGLN
CNN1
Synthetic
P<0.0001
P>0.9999
KLF4
OPN
MYH10
J
PDGF-BB
DMSO
Defactinib
Lenti-Ctrl
Lenti-Itga6
K
Lenti-Ctrl+Vehicle
Letni-Ctrl+Defactinib
Lenti-HINT1+Vehicle
Lenti-HINT1+Defactinib
Lenti-Ctrl+Vehicle+PDGF-BB
Letni-Ctrl+Defactinib+PDGF-BB
Lenti-HINT1+Vehicle+PDGF-BB
Lenti-HINT1+Defactinib+PDGF-BB
Contractile
Relative mRNA expression
P<0.0001
P=0.0004
P=0.0015
P>0.9999
ACTA2
TAGLN
CNN1
Synthetic
KLF4
OPN
MYH10

Supplemental Figure 11: ITGA6 promotes vascular smooth muscle cell phenotypic switching through activating FAK/STAT3 signal pathway.

(**A**), Proteins that interact with ITGA6 were identified to construct the protein-protein interactive (PPI) network using STRING. (**B**), Western blotting analysis of the nuclear translocation of STAT3 in HASMCs that transfected with si*N* or si*ITGA6* followed by PBS or PDGF-BB (20 ng/ml) stimulation. n=3 per group. (**C**), Western blotting analysis of phosphorylation levels of FAK and STAT3 in HASMCs pretreated with or without Defactinib (2.5 μM) followed by PBS or PDGF-BB (20 ng/ml) stimulation for 30 min. n=6 per group. (**D**), Representative confocal microscopy images showing the nuclear translocation of STAT3 (red) in HASMCs that pretreated with or without Defactinib (2.5 μM) followed by PBS or PDGF-BB (20 ng/ml) stimulation. DAPI, blue; scale bar = 20 μm. (**E**), Western blotting analysis of the nuclear translocation of STAT3 in HASMCs that pretreated with or without Defactinib (2.5 μM) followed by PBS or PDGF-BB (20 ng/ml) stimulation. n=3 per group. (**F**), qPCR analysis of the mRNA levels of VSMC contractile markers (*ACTA2, CNN1 and TAGLN*) and synthetic markers (*KLF4, OPN and MYH10*) in HASMCs that pretreated with or without Defactinib (2.5 μM) followed by PBS or PDGF-BB (20 ng/ml) stimulation. n=6 per group. (**G**), Western blotting analysis of phosphorylation levels of FAK and STAT3 in HASMCs infected with Lenti-*Ctrl* or Lenti-*ITGA6* and pretreated with or without Defactinib (2.5μM) followed by PBS or PDGF-BB (20 ng/ml) stimulation for 30 min. n=6 per group. (**H**), Western blotting analysis of the nuclear translocation of STAT3 in HASMCs infected with Lenti-*Ctrl* or Lenti-*ITGA6* and pretreated with or without Defactinib (2.5 μM), followed by PBS or PDGF-BB (20 ng/ml) stimulation. n=3 per group. (**I**), qPCR analysis of the mRNA levels of VSMC contractile markers (*ACTA2, CNN1 and TAGLN*) and synthetic markers (*KLF4, OPN and MYH10*) in HASMCs infected with Lenti-*Ctrl* or Lenti-*ITGA6* and pretreated with or without Defactinib (2.5 μM) followed by PBS or PDGF-BB (20 ng/ml) stimulation. n=6 per group. (**J**), Representative immunofluorescence images of F-actin (red) stained with phalloidin in RASMCs infected with Lenti-*Ctrl* or Lenti-*Itga6* and pretreated with or without Defactinib (2.5 μM) followed by PBS or PDGF-BB (20 ng/ml) stimulation. scale bar = 20 μm. (**K**), qPCR analysis of the mRNA levels of VSMC contractile markers (*ACTA2, CNN1 and TAGLN*) and synthetic markers (*KLF4, OPN and MYH10*) in HASMCs infected with Lenti-*Ctrl* or Lenti-*HINT1* and pretreated with or without Defactinib (2.5 μM) followed by PBS or PDGF-BB (20 ng/ml) stimulation. n=6 per group. Statistical analysis was performed by One-way ANOVA for (**B, C, E and F**), Two-way ANOVA for (**G, H, I and K**). For all statistical plots, the data are presented as mean ± SEM.

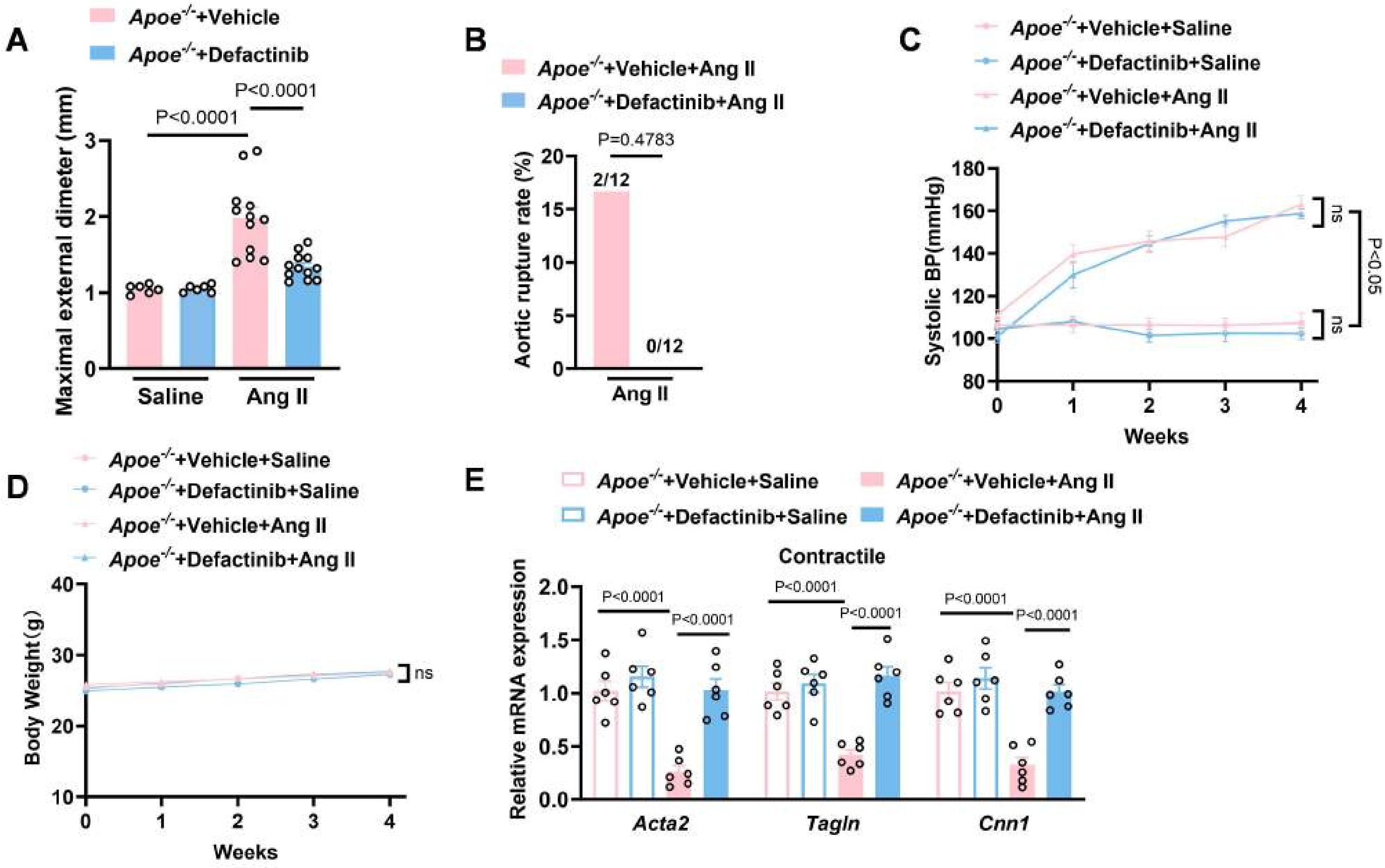

Supplemental Figure 12: Defactinib protects against aortic aneurysm.

(**A**), Maximum abdominal aortic diameters were assessed by measuring external aortic diameter from images. (**B**), The aortic rupture rate in Ang II-infused mice. (**C and D**), Systolic blood pressure (**C**) and Body weight (**D**) at 0, 1, 2, 3 and 4 weeks for saline or Ang II-infused *Apoe*$^{-/-}$ mice. (**E**), Severity stratification of Ang II-induced aortic aneurysm. (**F**), qPCR analysis of the mRNA levels of VSMC contractile markers (*Acta2, Tagln and Cnn1*) in suprarenal abdominal aortas from saline or Ang II-infused *Apoe*$^{-/-}$ mice. n=6 per group. Statistical analysis was performed by mixed-effects analysis followed by One-way ANOVA for (**A and E)**, Fisher exact test for (**B**), Two-way ANOVA with mixed-effects analysis for (**C and D)**,. For all statistical plots, the data are presented as mean ± SEM. ns, no significance.

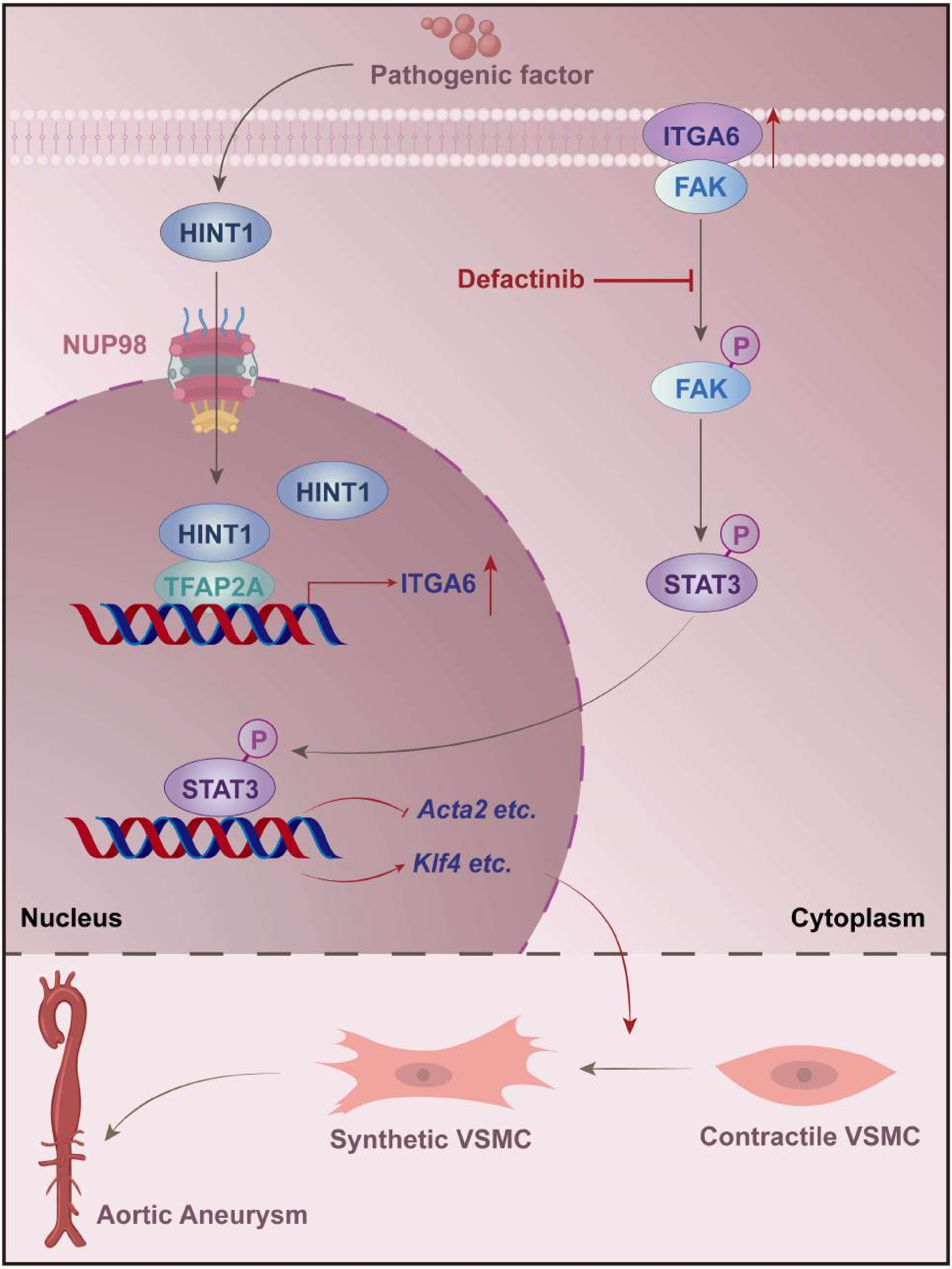

Supplemental Figure 13: Schematic illustration of the involvement of HINT1 in the progression of aortic aneurysm.
